# Identification of Key Components in Colon Adenocarcinoma Using Transcriptome to Interactome Multilayer Framework

**DOI:** 10.1038/s41598-020-59605-z

**Published:** 2020-03-19

**Authors:** Ehsan Pournoor, Zaynab Mousavian, Abbas Nowzari Dalini, Ali Masoudi-Nejad

**Affiliations:** 1 Laboratory of Systems Biology and Bioinformatics (LBB), Institute of Biochemistry and Biophysics, University of Tehran, Tehran, Iran; 2 School of Mathematics, Statistics, and Computer Science, College of Science, University of Tehran, Tehran, Iran

**Keywords:** Data integration, Data mining

## Abstract

Complexity of cascading interrelations between molecular cell components at different levels from genome to metabolome ordains a massive difficulty in comprehending biological happenings. However, considering these complications in the systematic modelings will result in realistic and reliable outputs. The multilayer networks approach is a relatively innovative concept that could be applied for multiple omics datasets as an integrative methodology to overcome heterogeneity difficulties. Herein, we employed the multilayer framework to rehabilitate colon adenocarcinoma network by observing co-expression correlations, regulatory relations, and physical binding interactions. Hub nodes in this three-layer network were selected using a heterogeneous random walk with random jump procedure. We exploited local composite modules around the hub nodes having high overlay with cancer-specific pathways, and investigated their genes showing a different expressional pattern in the tumor progression. These genes were examined for survival effects on the patient’s lifespan, and those with significant impacts were selected as potential candidate biomarkers. Results suggest that identified genes indicate noteworthy importance in the carcinogenesis of the colon.

## Introduction

One of the most prominent aspects of computational biology is network biology in which network science plays an essential role in discovering biological occurrences. Networks are reconstructed from different biological phenomena and deduced from mathematical and topological aspects. Protein-protein interaction (PPI), metabolic, and regulatory networks are examples of biological networks that have frequently been employed in recent years. However, nature comes with a lot of complexity. Inside a cell, there is a broad spectrum of entities contributing to create different cascading interactions that define the cell life and function. Considering these complexities in the modeling will gain comprehensive and valuable insights on biological events.

Currently, thanks to progress in high-throughput sequencing technologies and large projects such as TCGA^1^, there is a booming amount of omics data that could be used for wide-ranging analyses. Curated databases contain information for regulatory interactions between biomolecules and their targets, single nucleotide polymorphisms (SNPs), biological pathways, and gene expression profiles of various phenotypes. Moreover, tools and applications, developed by scientific societies, are increasingly accessible, resulted in an easier exploration of biological happenings^2^. However, these data are heterogeneous, inconsistent, and provider technologies may come with a bias^3^. To overcome these problems, data integration would be an asset. Recently, integrative systems biology has become a popular area in which more than just a single type of biologic data is incorporated^4–7^. Bearing these considerations in mind, the results will be more truthful and reliable. In an excellent review article written by Koyel *et al*.^8^, they described different approaches in integrative biological networks. Also, Peng *et al*.^9^ proposed a new multi-omics approach for bladder cancer-related genes discovery.

Systematically, multi-omics datasets could be regarded as multilayer networks. Based on scientific definitions^10,11^, a multilayer network contains multiple layers (different layers for different types of interactions) and considering the topology of layers, various kinds of multilayer networks are characterized. As Hmimida *et al*.^12^ mentioned, multilayer networks exploration can be executed in three ways. First, Layer Aggregation, in which all layers are aggregated to make a single network and traditional single layer (monoplex) analysis could be applied to explore it. Second, Ensemble (Consensus) approaches, in which each layer is individually evaluated; then, the results are combined to create the final consequence. Third, methods extended for multilayer networks (briefly called extended approaches), in which the analysis process is simultaneously conducted on all layers. Didier *et al*.^13^ compared these three approaches in terms of community detection and found that the extended modularity function has superiority over the other two methods.

The extension of topological attributes from monolayer to multilayer is a critical and challenging topic in this area^12,14–16^. Hmimida *et al*.^12^ have defined metrics (such as degree, shortest-path, neighbor set) for multiplex networks using an entropy-like aggregate function. Domenico *et al*.^16^ proposed reducibility methods for multilayer networks to eliminate redundant interactions and layers. In this context, community detection for multilayer networks is considered one of the most challenging topics. Given the topological perspective, a community is a cluster of densely connected nodes, which are far from other clusters. Communities may be either local or global and may have overlap with each other. Recently, various extended multilayer community detection algorithms have been proposed to seek modules in layers simultaneously^17–21^. A specific type of community detection method is based on seed-centric approach, in which communities are localized around predefined (manual or computational) seed nodes^12,22^.

Extended approaches for multilayer networks were recently used in biological and medical sciences. Berenstein *et al*.^23^ have taken benefit from these methods for the application of drug repositioning in neglected diseases. Furthermore, Rai *et al*.^24^ found a similar structure in the PPI network of seven types of cancers using spectral graph theory and the multilayer framework. Although these kinds of integrative methods were used recently in some contexts of biology, there is still a gap in their usage in prognosis and diagnosis of human disorders such as cancer even with the high availability of omics datasets. Also, because of the complexity of this kind of research with high dimensional data, previous multilayer-based works have relied mostly on the usage of two types of data and missed complementary interactions such as regulatory links in transcriptional and post-transcriptional phases.

Here, we utilize an extended approach to find functional communities in colon adenocarcinoma (COAD). To model such a multifaceted system in a realistic and comprehensive way, three levels of abstraction are declared. First, at the transcriptional level, gene correlations can be defined to represent the co-expression patterns among the genes. Second, at the post-transcriptional level, biomolecules such as RNAs and proteins have regulatory interrelations. Regulatory interactions indicate direct or indirect control of gene expression. Third, physical interactions show bindings of molecules such as proteins or RNAs to other molecules (such as protein-protein and RNA-protein bindings). In this arrangement, it is possible that a regulatory interaction may also be a physical binding interaction. Accordingly, we construct a three-layer network with co-expression, regulatory and physical interaction layers. One of the novelties addressed in this study is utilizing the most diverse types of interactions including co-expression, signaling, kinase-substrate, metabolic enzyme-coupled, nucleoproteins, protein complexes, RNA-RNA, and regulatory in a multi-layer framework to get a holistic view of the genes involved in carcinogenesis. The analysis program in this research contains the following steps to achieve potential biomarkers derived from raw datasets. We employ a local seed-centric community detection algorithm to explore modules in the multilayer network. In this process, to select seed items computationally, we propose an innovative multilayer heterogeneous random walker to score nodes. The top-ranked nodes are applied as seeds, and local communities around these seed nodes are computed as modules of interest. Out of the identified modules, those with a high overlap with COAD, based on validated databases, are selected as candidate modules for further steps. For every module, differential expression (DE) analysis is performed to extract differentially expressed genes (DEGs). We conduct the survival analysis for DEGs in the final step, to find genes that their differential expression influences the survival of patients with COAD. Modules containing a large amount of these genes are selected as final modules, and the functional enrichment was carried out on them. Finally, we discussed the upstream regulators of candidate genes to specify their role in the differential expression of target candidates and the related biological pathways. This kind of novel evaluation led us to identify a list of new potential targets in colorectal cancer^25^.

## Materials and methods

### Data collection and preprocessing

**Figure 1:**
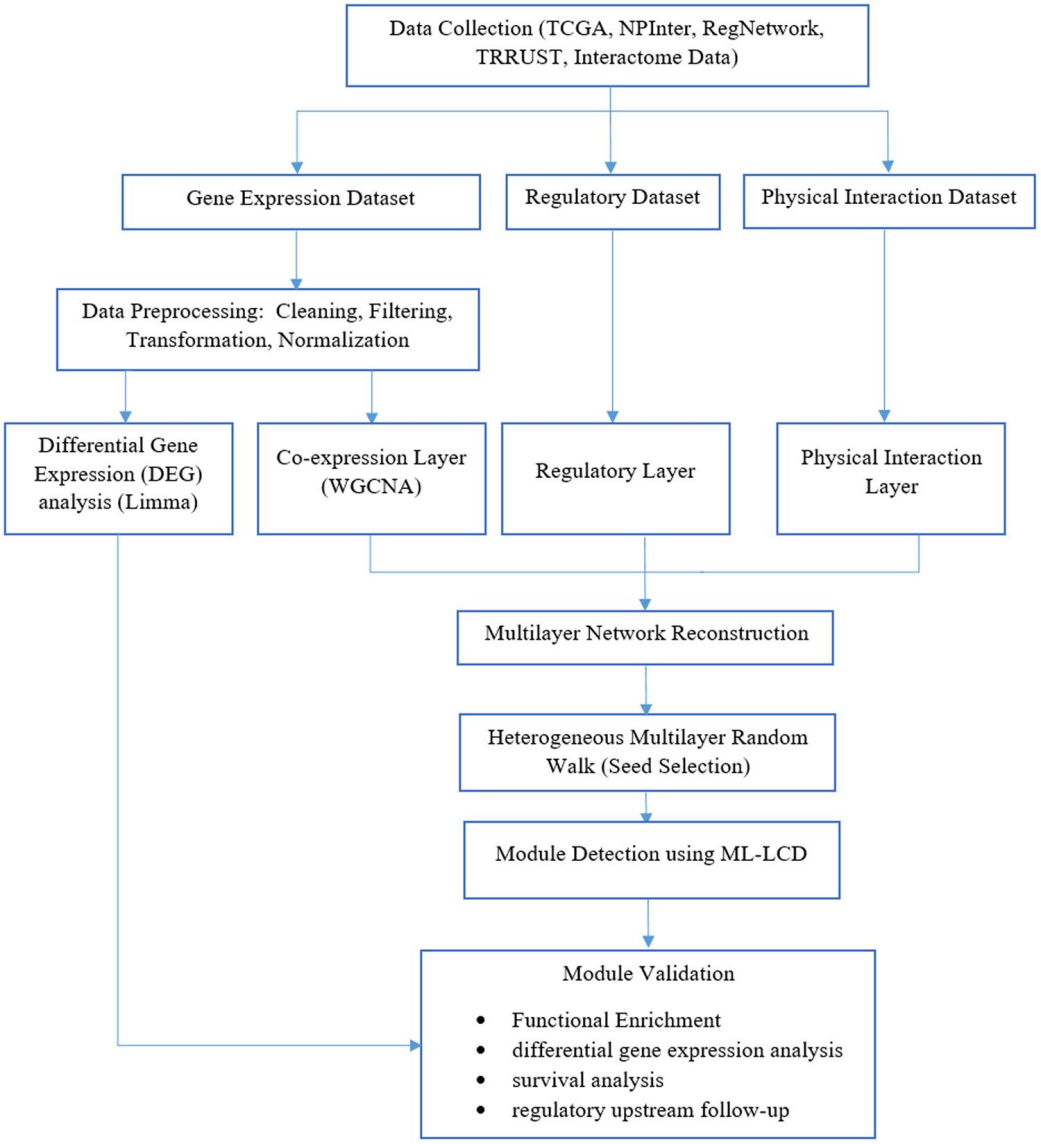
The Workflow of the research.

In the process of data collection, the information needed to build a multilayer network was gathered from multiple sources. As presented in the research workflow (Fig. 1), in this step, three types of data were used: gene expression profiles, regulatory relationships, and physically binding interactions. First, to build the co-expression network (layer 1), RNAseq data for COAD were exploited from the TCGA data portal^26^. In the preprocessing of expression data, we performed sample and gene filtering and then used the FPKM-UQ (Fragments Per Kilobase of transcript per Million mapped reads upper quartile) normalized data to generate the network. In the gene filtering step, genes with the following conditions were excluded: (1) missing values in any samples, (2) the expression count value of zero in more than 80% of all samples^27^, (3) genes that possessed the expression rates with zero standard deviation across all samples, and (4) genes with average CPM (count per million) lower than 1.

Second, for the generation of regulatory layer, we utilized the experimentally curated regulatory interactions using the NPInter (non-coding RNA interactions with biomolecules)^28^, the RegNetwork^29^ (Regulatory Network Repository of Transcription Factor and microRNA Mediated Gene Regulations), and the TRRUST (database of TF-target regulatory information)^30^ databases. Third, the network of physical interaction layer was assembled using the interactome data, as previously published^31^. These data comprise different interactions including (1) regulatory interaction of transcription factors, (2) yeast two-hybrid (Y2H) binary interactions, (3) metabolic enzyme-coupled interactions, (4) signaling interactions, (5) protein complexes, (6) kinase-substrate interactions, and (7) low-throughput manually curated interactions in the literature. In addition to the co-expression layer, in other layers (regulatory and physical interaction), genes possessing the average expression value of zero in transcriptomic data were also removed. Our reason is that genes with no expression will never be translated into proteins, and they have no regulatory function or physical binding interactions.

### Multilayer network construction

**Figure 2:**
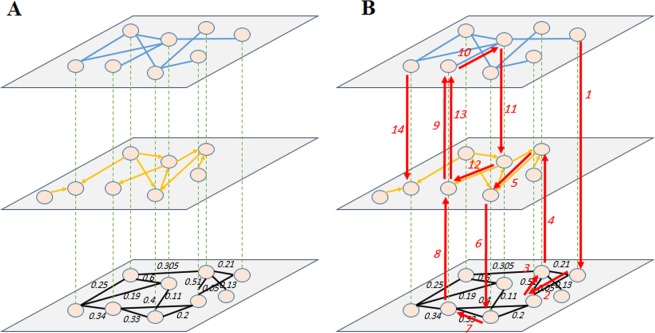
(**A**) A sample heterogeneous multilayer network of the transcriptome to interactome. (**B**) A typical random walk with the random jump.

Herein, we used FPKM-UQ normalized data and established the co-expression network using the WGCNA^27^ package in the R. In this layer; we selected those correlations having the values higher than 0.75 (considering scale-freeness of the network). Since the gene-set is made of both coding and non-coding RNAs and contains regulators and their targets, in the WGCNA “adjacency” function, we set the network type to “unsigned,” and correlations were calculated using the Pearson Correlation Coefficient (PCC) measure. The output is a weighted and undirected network in which the edge weights address correlations between the genes. We built other layers of the multilayer network (regulatory and physical binding layers) using interactions deduced by the experimentally curated databases. The regulatory network is directed, while the physical interaction network is undirected; however, both of them are unweighted networks. Figure 2A depicts such a heterogeneous multilayer network.

### Personalized seed selection

In most of the seed-centric community detection approaches, the seeds are either selected manually (such as predefined nodes) or based on their specific properties (such as being a hub). Here, to select seeds in a sophisticated way, nodes were ranked using a random walk with a random jump procedure. To attain this objective, we customized the random walker to walk in all three layers considering the heterogeneity. Since the network topology (node set, edge set, directedness, and edge weighting) is different in each layer (Fig. 2A), we proposed a walker algorithm (algorithm 1) to resolve this kind of ambiguity.

**Algorithm 1:**
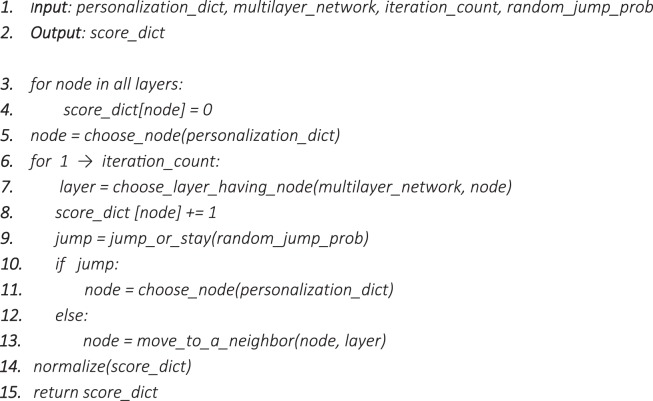
Proposed heterogeneous multilayer random walker.

The walker must be able to move in all three layers with the ability to change layers and nodes. Figure 2B shows a sample walk with node and layer exchange. In this procedure, moving to a neighbor follows the probability function ${P}_{l}(i,j)$ defined in Eq. (1) for unweighted and Eq. (2) for weighted networks.

As defined by Kivelä *et al*.^10^, given a set of nodes ${\mathcal{V}}$ and a set of layers ${\mathcal{L}}=\{{L}_{1},\,{L}_{2},\,\ldots ,\,{L}_{s}\}$, ${V}_{{\mathcal{L}}}\subseteq {\mathcal{V}}\times  {\mathcal L}$ specifies nodes and ${E}_{{\mathcal{L}}}\,\subseteq \,{V}_{{\mathcal{L}}}\times {V}_{{\mathcal{L}}}$ denotes the edge list (including inter-layer and intra-layer edges) in a multilayer framework. We indicated the multilayer network graph with notation ${G}_{{\mathcal{L}}}=({V}_{ {\mathcal L} },\,{E}_{{\mathcal{L}}},\,{\mathcal{V}},\,{\mathcal{L}})$. In this context, the probability of proceeding to a next neighbor in unweighted networks (regulatory and physical interaction layers) is defined as:

1 $${P}_{l}(i,j)=\frac{1}{{\sum }_{k{\epsilon }{m}_{l}}{A}_{l}(i,k)},\,{A}_{l}(i,j)=\{\begin{array}{cc}1 & if\,there\,is\,an\,edge\,from\,node\,i\,to\,j\\ 0 & else\end{array}$$

where ${P}_{l}(i,j)$ is the probability of proceeding from node *i* to any other neighbor (out-neighbors) j in layer *l*, ${m}_{l}$ indicates the neighbors (or out-neighbors if the network is directed) of node *i* in layer *l*, and ${A}_{l}$ demonstrates the adjacency matrix of the layer *l*. However, in the co-expression layer, we set the walk probabilities as:

2 $${P}_{l}(i,j)=\frac{{A}_{l}(i,j)}{{\sum }_{k{\epsilon }{m}_{l}}{A}_{l}(i,k)},\,{A}_{l}(i,j)=\{\begin{array}{cc}{Corr}_{l}(i,j) & if\,there\,is\,an\,edge\,from\,node\,i\,to\,j\\ 0 & else\end{array}$$

where $Corr(i,j)$ implies the co-expression correlation between nodes (genes) *i* and *j*. Also, the walker must be able to go from a node to its counterparts in other layers. For instance, when the walker is at node *x* inside layer $\,{L}_{1}$, for any layer ${L}_{r}\in {\mathcal{L}},\,r\ne 1$, if ${L}_{r}$contains node *x*, the walker can jump to node *x* in ${L}_{r}$, or stay in ${L}_{1}$ itself. In each step, the walker should choose to go to one of its counterparts in other layers (line 7 in algorithm 1) or move to one of its neighbors in the same layer (lines 13 in algorithm 1). We set the probability of moving to a neighbor in the same layer as same as moving to its counterpart in a different layer to enable the walker to score nodes based on shared properties of nodes in all layers in an unbiased manner. Moreover, to avoid tangle in traps, we set the walker to jump with a predefined probability ${\rm{\beta }}$or continue to walk with a probability of $1-{\rm{\beta }}$ (line 10 in algorithm 1). To be fair in the selection of jump destination and ascribe it to the biological gene expression, we set the personalized random jump probability as:

3 $$pr(i)=\frac{\exp (i)}{{\sum }_{k=1}^{N}\exp (k)}$$

where $pr(i)$ specifies the probability to jump to node *i*, $\exp (i)$ indicates the average expression of gene *i* across the samples of patients with cancer and *N* is the total number of genes (nodes) in the multilayer framework.

### Local seed-centric module detection

We used a local community detection approach to detect the functional composite modules around the seed nodes in our multilayer network. The idea underlying this approach is to find items that contribute to the development of carcinogenesis. Items are scattered in layers, and their nature might be a gene, RNA, or protein; however, we consider the equivalent gene names in the final module. Since currently there is not an applicable community detection algorithm about large heterogeneous multilayer networks compatible with our network; in this phase, we considered layers in an unweighted and undirected manner. We utilized ML-LCD local community detection method^22^ to discover modules for every seed node. It is a modularity expansion-based community detection method, which has better performance in large multilayer networks. Given a seed node ${v}_{0}\in {\mathcal{V}}$, ML-LCD algorithm attempts to find a subgraph ${G}_{{\mathcal{L}}}^{{v}_{0}}\subseteq \,{G}_{{\mathcal{L}}}$ that consisted of the seed node *v*_0_ and maximizes the local community function (LC) in Eq. (4). For simplifications, the letter *C* is used to denote local community subgraph. In this regard, ${E}^{C}\,\subseteq \,{E}_{{\mathcal{L}}}$ demonstrates the edge set of the subgraph.

4 $${G}_{{\mathcal{L}}}^{{v}_{0}}=\begin{array}{c}argmax\,LC(C)\\ C=(V,E,{\mathcal{V}},{\mathcal{L}})\subseteq {G}_{{\mathcal{L}}}\wedge {v}_{0}\in V\end{array}$$

where:

5 $$LC(C)=\frac{L{C}^{int}(C)}{L{C}^{ext}(C)}$$

In Eq. (5), $L{C}^{int}(C)/L{C}^{ext}(C)$ indicates the density of links inside *C*, over the density of links between nodes inside and outside of the *C*. To formulate local community function LC, terms *Shell Nodes* and *Boundary Nodes* should be defined. In this regard, to specialize the edges inside the community *C* in a layer ${L}_{i}$, symbol ${E}_{i}^{C}=\{(u,\,v)|\exists ((u,\,{L}_{i}),\,(v,\,{L}_{i}))\in {E}^{C}\}$ will be used. For a local community being constructed, the shell nodes refer to nodes outside of the community that is a neighbor of nodes inside the community (displayed by symbol S), and these within-community neighbors of shell nodes are also called boundary nodes (depicted by symbol B):

$$S=\{v\in {\mathcal{V}}\backslash C|\exists ((u,{L}_{i}),(v,{L}_{j}))\in {E}_{{\mathcal{L}}}\wedge u\in C\}$$

$$B=\{v\in C|\exists ((u,{L}_{i}),(v,{L}_{j}))\in {E}_{{\mathcal{L}}}\wedge v\in S\}$$

Furthermore, ${E}^{B}=\{(u,v)|\exists ((u,\,{L}_{i}),\,(v,{L}_{j}))\in {E}_{{\mathcal{L}}}\wedge u\in B\wedge v\in S\}$ indicates the set of edges outgoing from the *C*, and for any layer ${L}_{i}$, ${E}_{i}^{B}=\{(u,v)|\exists ((u,\,{L}_{i}),\,(v,{L}_{i}))\in {E}^{B}\}$ is the portion of ${E}^{B}$ corresponding to edges of layer ${L}_{i}$. As reported by^22^, $L{C}^{int}(C)$ and $L{C}^{ext}(C)$ are defined as:

6 $$L{C}^{int}(C)=\frac{1}{|C|}\sum _{v\,\in C}\sum _{{L}_{i}\,\in \,{\mathcal{L}}}{\omega }_{i}|{E}_{i}^{C}(v)|$$

7 $$L{C}^{ext}(C)=\frac{1}{|B|}\sum _{v\,\in B}\sum _{{L}_{i}\,\in \,{\mathcal{L}}}{\omega }_{i}|{E}_{i}^{B}(v)|$$

where ${\omega }_{i}$ (for every ${L}_{i}$ ∈ ${\mathcal{L}}$) is non-negative coefficient, with $\,\sum _{{L}_{i}\,\in \,{\mathcal{L}}}{\omega }_{{L}_{i}}=1$, demonstrating layer weights.

### Module validation and biomarker extraction

Communities detected by the mentioned approach should be validated by a biological viewpoint. Our goal was to seek modules (communities) that their genes were involved in the malignant tumor of the colon. We chose modules possessing considerable counts of genes associated with COAD according to the DisGeNet^32^ and containing a significant number of DEGs. For these disease-related modules, functional enrichment analysis is a strategy to check the role of module genes in pathways and processes. To enrich the discovered modules, we employed two popular tools, the Enrichr^33,34^ and ToppFun enrichment portal of the ToppGene^35^, and studied the presence of modules genes in tumor-associated pathways and biological processes. We selected the KEGG^36^ and Reactome^37^ as reference pathway databases.

For the genes inside the discovered modules, that were not previously reported as COAD-associated genes in the DisGeNet database, differential gene expression analysis was performed. Herein, we focused on genes that their expression pattern statistically differs in the malignant tumor. We did DE analysis using the Limma^38^ and the edgeR^39^ R packages based on workflow presented in^40^ and selected genes with larger fold changes ($|\log (FC)| > 1$) and smaller p-value (adj. p-value < 0.01).

To observe the effects of the expression level of discovered DEGs on the survival of patients, the survival analysis was carried out. This examination was applied to compare the lifespan of people when the expression of a gene differs from the normal. Kaplan-Meier (KM)^41^ curve is a worthy choice when the data are censored, and there is not complete information on subjects. It estimates the lifespan of a group of people having a low gene expression rate in comparison to another group with a high expression rate. The SurvExpress^42^ utility was used for the log-rank test and Kaplan-Meier survival analysis. In this phase, we chose those genes that a change in their expression rates influences the survival rate of patients with COAD and considered them as potential biomarkers. Although the impact of variations on the expression of upstream regulators is not the only reason for changes in the expression of downstream genes, it provides some explanations for these alterations. Finally, to validate changes in the transcription levels of biomarkers, in the regulatory layer, we performed a regulation follow-up on upstream items of biomarkers.

## Results

### Data preparation and network generation

The obtained dataset from the TCGA includes total RNAseq expression data for 60483 coding/non-coding RNAs in 424 samples that consisted of samples collected from healthy subjects and patients with colon cancer. Thus, we selected the paired samples that comprised of 49 samples obtained from healthy individuals and their cancer counterparts (49 normal specimens and 49 cancerous samples). Then, we carried out gene filtering as described in the “Materials and Methods” section. The cleaned data include the expression count value for 14515 genes in 98 samples. The 49-paired samples were used in the differential gene expression analysis. However, only the expression data of 49 cancerous samples were applied for the co-expression network construction. The generated co-expression layer comprises 5993 nodes and 75121 edges. On the other side, the regulatory and physical interaction layers were directly generated from source datasets. The physical interaction layer has 12751 nodes and 135712 edges, and the regulatory layer encompasses 17640 nodes with 133180 edges.

### Personalized seed selection

**Figure 3:**
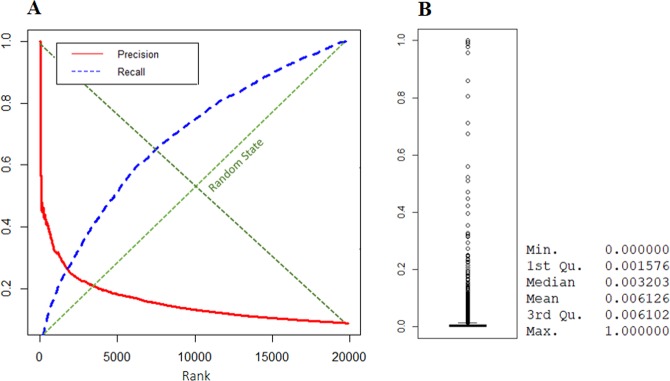
(**A**) Evaluation of walker in terms of precision and recall for COAD. (**B**) Distribution of nodes score.

We ranked nodes in the network using the proposed heterogeneous multilayer random walker. The walker was set to jump with a probability of ${\rm{\beta }}$ = 0.2 or continue to walk with a probability of 0.8. We ran the walker for three million moves and ranked nodes based on scores (Min. = 0.0, 1st Qu. = 0.0015, Median = 0.0032, Mean = 0.00612, 3rd Qu. = 0.00610, Max = 1.0). Although the goal of this project was far from the gene prioritization, to test the accuracy of our personalized seed selection algorithm against a random selection of genes, we examined the result of the walker in terms of precision and recall (Fig. 3). Precision (Eq. 8) measures the percentages of retrieved items that are relevant. However, recall (Eq. 9) evaluates the percentage of relevant items that have been retrieved. Results show that the proposed ranking system performs much better than the random assortment (diagonals of the chart). The complete result of random walker scores is provided in Supplementary File S1. We designated nodes with score larger than 0.5 (*rw_score* > 0.5) as seed nodes. As reported by the DisGeNET database, 11 out of 12 chosen seed nodes are involved in COAD, as previously mentioned, indicating that our computationally explored centroid seeds are biologically meaningful.

8 $$precision=\frac{|\{relevant\,items\}{\cap }^{}\{retrieved\,items\}|}{|\{retrieved\,items\}|}$$

9 $$recall=\frac{|\{relevant\,items\}{\cap }^{}\{retrieved\,items\}|}{|\{relevant\,items\}|}$$

### Module detection

For the selected seed nodes, we calculated local communities using the ML-LCD method. Since there is not any precedence on the three layers, we set layer weight ${\omega }_{{L}_{i}}=1/3$ for all layers equally. We selected modules whose genes (at least 25%) are known as predisposing genes in the development of COAD according to the reports by curated databases. Out of 12 modules found in this step, seven were related to colon cancer with an overlap more than the specified threshold (Table 1). The detailed information of 12 modules and their genes are available in Supplementary File S2.

**Table 1:** Explored modules around seed nodes with an overlap percentage higher than 0.25 in COAD.

#	Seed	Seed RW score	number of module genes	Involvement percentage	p-value	FDR B&H
1	SP1	0.977729191	239	0.414	2.667E-33	3.54E-30
2	EGR1	0.858748157	250	0.464	3.344E-44	3.31E-41
3	USF1	0.805867697	153	0.614	5.507E-48	2.97E-44
4	YY1	0.674073321	140	0.364	4.201E-15	7.65E-13
5	E2F1	0.559109168	156	0.25	6.14E-07	3.27E-05
6	MXI1	0.523567397	63	0.302	0.00003692	0.001946
7	JUN	0.511059135	140	0.457	2.984E-25	3.84E-22

Also, we checked random walk scores of module items separately to observe if there is a relationship between the module score and the percentage of its overlap with COAD. In all validated modules (seven modules), the minimum, maximum, median, and average scores were calculated (Table 2). It was observed that the average module score was highly associated with the involvement of module in COAD. For example, for the module with the seed gene *USF*1 (called module *USF1*), the average score of module items was the highest (avg. score = 0.05692) and it was the most related module to cancer (overlap = 61%). It emphasizes that COAD-related modules contain nodes with a high random walk score (hub nodes in the multilayer framework).

**Table 2:** Module relevance to COAD in terms of Random walk scores.

Module Seed	Min	Max	Avg.	Median	Involvement %
USF1	0.0061	0.80587	0.05692	0.03458	61%
YY1	0.00422	0.67407	0.03989	0.02677	46%
JUN	0.00158	0.51106	0.03635	0.0257	45%
EGR1	0.00219	0.85875	0.03331	0.02212	41%
SP1	0.00214	0.97773	0.03095	0.01978	36%
MXI1	0.00422	0.52357	0.02937	0.01693	30%
E2F1	0.00417	0.55911	0.01669	0.0091	25%
All Genes	0	1	0.00613	0.0032	—

### DEG analysis and module evaluation

**Figure 4:**
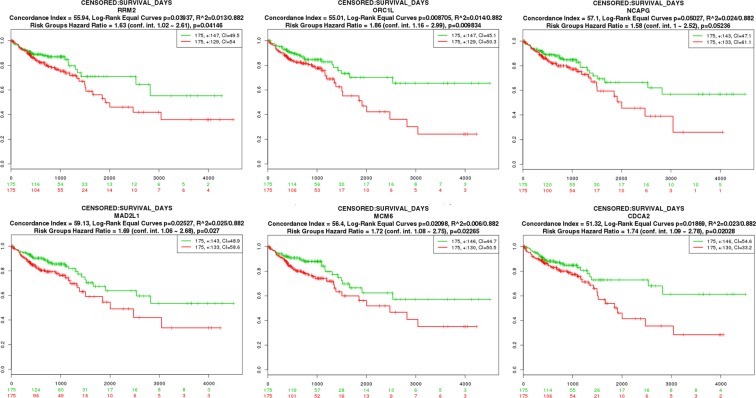
Kaplan-Mier curves (from SurvExpress tools) for candidate biomarkers *RRM2*, *ORC1*, *NCAPG*, *MAD2L1*, *MCM6*, and *CDCA2* selected with log-rank test p-Value < 0.05.

To assess each detected module, we applied differential gene expression analysis, with limitations adj. p-value < 0.01 and $|\log (FC)| > 1$. As presented in Table 3, the module *E*2*F1* has the highest value of differentially expressed genes (100 DEGs from 117 genes). We started with DEGs that their expression rate was changed in the transition from healthy to cancer state, and continued to survival analysis. Among the identified DEGs that were not reported in databases, 10 genes had different expression patterns (modules with seed genes *E2F1* and *USF1*), resulting in a different survival rate with log-rank test p-value < 0.05 (Table 3, Fig. 4). The genes *CDC6, RRM2, ORC1, NCAPG, MAD2L1, MCM6, CCNF, CDCA2, ECT2*, and *DEPDC1B*, are the output candidate genes identified in explored modules, which are differentially expressed between normal and cancer states, and their differential expression levels have noteworthy effects on the lifespan of patients. Since we looked for novel unreported genes, we ignored modules with low coverage of DEGs and survival biomarkers and proceed with two significant modules, the module *E2F1* (high DEG and survival rate) and the module *USF1* (high overlap) for functional enrichment analysis.

**Table 3:** Module comparison and evaluation.

	Module Seed	USF1	YY1	JUN	EGR1	SP1	MXI1	E2F1
1	# module genes	153	140	140	250	239	63	156
2	# COAD genes in 1	94 (61%)	51 (36%)	64 (45%)	116 (46%)	99 (41%)	19 (30%)	39 (25%)
3	# DEGs in 1	21 (13%)	8 (5%)	11 (7%)	40 (16%)	35 (14%)	1 (1%)	130 (83%)
4	# DEGs in (1–2)	3 (5%)	4 (4%)	3 (3%)	12 (8%)	14 (10%)	1 (2%)	100 (85%)
5	# Survival Candidate in 4	1	0	0	0	0	0	9

The first row demonstrates the number of genes inside each module. For each module, its overlap with COAD has been presented in the second row. Values in the third row are the count of DEGs inside each module. However, the fourth row shows the number of DEGs in module genes, which were not previously reported as COAD-related genes in databases. Additionally, the number of genes with significant effects on patients’ survival (according to the fourth row) are presented in the fifth row.

In another parallel process, we performed another tryout to validate our approach in finding differentially expressed genes inside detected modules. For this purpose, the proportional test was used to check the accuracy of our method in distinguishing genes that their expression varies between healthy samples and patients with COAD. In other words, this test was designed to answer the question of whether the proposed approach would be sufficiently precise in discovering DEGs inside the identified modules. For this aim, the “One-sample proportions test with continuity correction” method was applied. This was executed through the *prop.test* function in the R. The *prop.test* can be used for examination of the null hypothesis if the proportions (probabilities of success) in several groups are the same, or they have certain equal values. Pearson’s chi-square test statistic 16.87184 with degree freedom of 1 and a p-value of 3.99906e-05 demonstrates relatively high precision in the detection of DEGs in our proposed module detection approach.

### Functional enrichment

**Figure 5:**
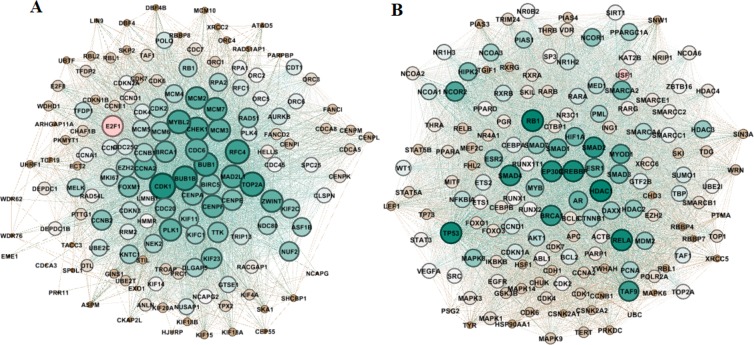
FLN subnetworks for modules with seed nodes (**A**) *E2F1* and (**B**) *USF1*. Nodes’ sizes are based on their degree.

The module *E2F1* has the highest number of differentially expressed genes that have survival impact; however, it has a low coverage with respect to the predefined COAD genes. On the side, the module *USF1* covers more percentage of COAD-related genes but contains fewer DEGs and effects on the survival rate. To study these two modules deeply, we utilized the human Functional Linkage Network (FLN), in which each node is a protein, and there is an edge between two nodes if there is evidence that nodes have a degree of the functional similarity. In this network, edges are weighted and predicted based on PPI interactions, gene expression profiles, literature mining, experimental techniques, and computational approaches^43,44^. A common methodology for predicting function based on FLNs applies a simple local threshold rule (mentioned as ‘guilt-by-association’)^45^. For the genes inside modules that were not specified as COAD related genes in databases, to explore their role in neoplastic cellular processes, we used functional linkage similarities of those genes with previously annotated genes. In the full FLN network, edge weights demonstrate the functional similarity between proteins. We extracted the subnetworks of the two modules from the human FLN network provided that the edge weights were higher than the average of the whole network. The *E2F1* FLN (Fig. 5A) is a dense subnetwork with an average path length of 1.18 and a density of 0.41. Out of 156 genes inside the module, 148 genes have functional similarities above the average values with respect to other module members. We surveyed the direct neighbors (containing 12111 nodes) of the module *E2F1* (module boundary); of them, 1496 were specified as cancer-related genes (coverage = 0.78). The module *USF1* (Fig. 5B) has a high intersection with the COAD; however, its boundary neighbors also have a tight coverage with cancer-related genes (coverage = 0.84). It is a dense subnetwork with a density of 0.59 and a network diameter of 1.4. Among genes belonging to the module *USF1*, 153 genes were in its FLN subnetwork, demonstrating the biological and chemical similarities between them. The functional linkage subnetworks are biological evidence on the modularity and resemblance of their genes and suggest a strong possibility to constitute a module and act together in biological pathways.

Additionally, to evaluate the relevance of selected modules to the development of carcinogenesis, we performed functional enrichment analysis. Results showed a significant correlation between discovered modules and pathways involved in the malignant tumors of the colon. Therefore, we set a threshold of 0.05 for enrichment adj. p-value. The extracted significant pathways for modules *USF1 and E2F1* are listed in Tables 4 and 5, respectively. The detailed results for functional enrichment of the two modules are accessible in Supplementary File S3. It was found that both modules were involved in pathways such as *Adipogenesis*, *Cell Cycle*, *Chromosomal and microsatellite instability in colorectal cancer*, and *TGF-beta Signaling Pathway* that they play crucial roles in colorectal cancer. However, the module *E2F1* seems to be involved in a specific way. Its genes have roles in key pathways involved in colon cancer such as *Wnt Signaling Pathway*, *Apoptosis*, *Tumor suppressor activity of SMARCB1*, *G1 to S cell cycle control*, *TGF-beta Signaling Pathway*, *H19 action Rb-E2F1 signaling, and CDK-Beta-catenin activity*, *Regulation of Wnt/B-catenin Signaling by Small Molecule Compounds* and *lncRNA involvement in canonical Wnt signaling and colorectal cancer*. Moreover, Gene Ontology (GO) that is associated with *E2F1* modules includes biological processes such as *mitotic cell cycle, cell division*, *chromosome organization*, *G1/S transition of the mitotic cell cycle*, *DNA metabolic process*, and *DNA repair* which are linked to cell proliferation (Supplementary File S3).

**Table 4:** Significant pathways for the module *USF1*.

Term	P-value	Adj. P-value	Z-score
Androgen receptor signaling pathway WP138	6.79E-58	2.17E-55	−1.38815
TGF-beta Signaling Pathway WP366	7.66E-38	1.23E-35	−1.2601
Nuclear Receptors WP170	6.06E-31	4.85E-29	−1.53161
Adipogenesis WP236	8.28E-30	5.3E-28	−1.04883
Cell Cycle WP179	1.46E-27	7.77E-26	−1.11229
AGE/RAGE pathway WP2324	2.48E-25	1.13E-23	−1.9584
Non-small cell lung cancer WP4255	1.44E-23	4.62E-22	−1.53125
RAC1/PAK1/p38/MMP2 Pathway WP3303	2.65E-23	7.06E-22	−1.39478
Oncostatin M Signaling Pathway WP2374	5.77E-22	1.35E-20	−1.85675
Circadian rhythm related genes WP3594	5.89E-22	1.35E-20	−0.88343
TGF-beta Receptor Signaling WP560	1.01E-21	2.16E-20	−2.33059
VEGFA-VEGFR2 Signaling Pathway WP3888	1.54E-21	3.09E-20	−0.79477
DNA Damage Response (only ATM dependent) WP710	1.04E-20	1.97E-19	−1.06194
TNF related weak inducer of apoptosis (TWEAK) Signaling Pathway WP2036	5.64E-20	9.49E-19	−2.38891
Brain-Derived Neurotrophic Factor (BDNF) signaling pathway WP2380	8.93E-20	1.43E-18	−0.86385
Chromosomal and microsatellite instability in colorectal cancer WP4216	2.24E-19	3.41E-18	−1.18043
Energy Metabolism WP1541	3.53E-19	5.13E-18	−1.95291
Leptin signaling pathway WP2034	4.51E-19	6.28E-18	−1.01867
IL-4 Signaling Pathway WP395	3.2E-18	4.1E-17	−1.68254
RANKL/RANK (Receptor activator of NFKB (ligand)) Signaling Pathway WP2018	4.26E-18	5.25E-17	−1.67297
ErbB Signaling Pathway WP673	9.84E-18	1.12E-16	−1.27747
Aryl Hydrocarbon Receptor WP2586	1.5E-17	1.65E-16	−1.90374
Thymic Stromal LymphoPoietin (TSLP) Signaling Pathway WP2203	2.06E-17	2.2E-16	−2.11
Wnt/beta-catenin Signaling Pathway in Leukemia WP3658	2.55E-17	2.64E-16	−2.43499
Nuclear Receptors Meta-Pathway WP2882	3.15E-17	3.15E-16	−0.67084
DNA Damage Response WP707	1.11E-16	1.08E-15	−1.09236
miRNA Regulation of DNA Damage Response WP1530	2.12E-16	1.94E-15	−1.33999
Non-genomic actions of 1,25 dihydroxyvitamin D3 WP4341	2.12E-16	1.94E-15	−0.88709
Vitamin D in inflammatory diseases WP4482	3.05E-16	2.71E-15	−2.36581
Corticotropin-releasing hormone signaling pathway WP2355	4.19E-16	3.62E-15	−1.07055
Integrated Cancer Pathway WP1971	4.43E-16	3.64E-15	−2.03412
Interleukin-11 Signaling Pathway WP2332	4.43E-16	3.64E-15	−2.02649

**Table 5:** Significant pathways for the module *E2F1*.

Term	P-value	Adj. P-value	Z-score
Cell Cycle WP179	1.97965E-63	2.71E-61	−1.15315
G1 to S cell cycle control WP45	3.94348E-47	1.8E-45	−1.95642
DNA Replication WP466	2.39913E-38	8.22E-37	−2.09373
miRNA Regulation of DNA Damage Response WP1530	1.76872E-24	4.85E-23	−1.72269
DNA Damage Response WP707	3.80714E-23	8.69E-22	−1.32103
DNA IR-damage and cellular response via ATR WP4016	9.81255E-22	1.92E-20	−1.27862
ATM Signaling Pathway WP2516	1.88381E-11	2.87E-10	−2.23923
Integrated Cancer Pathway WP1971	4.75612E-11	6.52E-10	−2.23108
Regulation of sister chromatid separation at the metaphase-anaphase transition WP4240	9.65678E-10	1.1E-08	−2.3819
DNA IR-Double Strand Breaks (DSBs) and cellular response via ATM WP3959	1.02091E-08	9.99E-08	−2.07864
H19 action Rb-E2F1 signaling and CDK-Beta-catenin activity WP3969	5.12131E-08	4.68E-07	−2.84809
ID signaling pathway WP53	1.10338E-07	8.89E-07	−2.87544
Human Thyroid Stimulating Hormone (TSH) signaling pathway WP2032	8.12668E-07	5.3E-06	−1.74211
Tumor suppressor activity of SMARCB1 WP4204	3.90517E-06	2.43E-05	−2.13364
Non-small cell lung cancer WP4255	1.2629E-05	6.92E-05	−1.38824
DNA Mismatch Repair WP531	3.77788E-05	0.000185	−2.89841
Photodynamic therapy-induced AP-1 survival signaling. WP3611	4.32184E-05	0.000197	−1.95428
Wnt Signaling Pathway WP363	5.23527E-05	0.000231	−2.18808
TGF-beta Signaling Pathway WP366	8.00653E-05	0.000343	−0.93161
LncRNA involvement in canonical Wnt signaling and colorectal cancer WP4258	9.45935E-05	0.000393	−1.14694
Chromosomal and microsatellite instability in colorectal cancer WP4216	0.000265291	0.001069	−1.07313
Regulation of Wnt/B-catenin Signaling by Small Molecule Compounds WP3664	0.000292112	0.001143	−1.89391
Aryl Hydrocarbon Receptor WP2586	0.000450398	0.001668	−1.83167
Androgen receptor signaling pathway WP138	0.000698091	0.002391	−1.02597
Wnt/beta-catenin Signaling Pathway in Leukemia WP3658	0.001060871	0.00338	−2.25473
PPAR Alpha Pathway WP2878	0.001060871	0.00338	−1.32492
Wnt Signaling Pathway and Pluripotency WP399	0.001227751	0.003823	−0.87218
Extracellular vesicle-mediated signaling in recipient cells WP2870	0.001619214	0.004822	−1.99363
Association Between Physico-Chemical Features and Toxicity Associated Pathways WP3680	0.001763243	0.00514	−1.61192
ATR Signaling WP3875	0.002099432	0.005981	−2.88868
Adipogenesis WP236	0.00354635	0.009717	−0.68844
Homologous recombination WP186	0.004456552	0.011972	−2.24022
Endoderm Differentiation WP2853	0.005003582	0.013183	−0.7774
PI3K-Akt Signaling Pathway WP4172	0.005302493	0.013706	−0.26886
Regulation of Microtubule Cytoskeleton WP2038	0.005527788	0.014024	−1.48329
Senescence and Autophagy in Cancer WP615	0.009259877	0.023066	−0.91906
Wnt Signaling WP428	0.012618554	0.029806	−0.8459
Vitamin D Receptor Pathway WP2877	0.014158639	0.032877	−0.63668
IL-7 Signaling Pathway WP205	0.016124042	0.036213	−2.13899
RAC1/PAK1/p38/MMP2 Pathway WP3303	0.016118811	0.036213	−0.70315
AMP-activated Protein Kinase (AMPK) Signaling WP1403	0.016757067	0.037028	−0.97432
Apoptosis WP254	0.028018802	0.04767	−0.75476

### Method evaluation

In order to evaluate the framework of this study against other community detection methods, we defined a two-step comparative analysis.

First, to assess our results in contrast with other multilayer methods, two well-known frameworks, Gene4x^46^ and mPageRank^19^, were selected and measures of central tendency were used to examine the distribution of each method overlap with disease-related genes (Table 6). We applied the mPageRank on the two layers of co-expression and PPI, by choosing random seeds from COAD-specific genes, as defined in the original article. However, the Gene4x is not a seed-centric approach, and from its multiple output modules, we selected top-ranked ones with considerable size for the valuation task. Here, due to the unavailability of differential expression data for the Gene4x method, evaluation has continued with the finding of the similarity of modules to gene sets involved in colorectal cancer. The results of this evaluation reveal the accuracy of our method in finding relevant communities.

**Table 6:** The overlap of communities resulted from multi-network methods with DisGeNet.

Method/Framework	Min.	1^st^ quart.	Med.	3^rd^ quart.	Mean	Max.
*Gene4x*	0.2580	0.2583	0.2586	0.2589	0.3183	0.3750
*mPageRank*	0.0	0.0016	0.0032	0.0048	0.1865	0.3838
*Our Approach*	0.2692	0.2697	0.2702	0.2706	0.4121	0.6143

**Figure 6:**
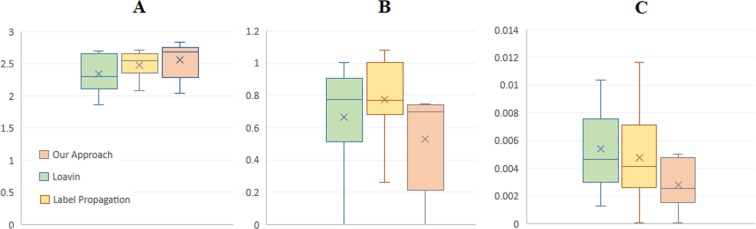
Comparison of single-layer methods, Loavin and Label-propagation, with our approach using DEG-based criteria defined by Cantini *et al*.^46^. (**A**) |mean _i∈C_ (log2 (fold change)_i_)|; (**B**) sd_i∈C_ (log2 (fold change)_i_); (**C**) Student’s t-test p-value. The framework employed here unfolds homogenous communities (low standard deviation of expression change) containing genes with higher changes in their expression and less p-value.

Second, to test the performance of the prepared framework with other single-layer methods, another validation on detected modules was accomplished using DEG-based criteria defined by Cantini *et al*.^46^. We selected two single-layer community detection algorithms, Loavin^47^ and Label-propagation^48^, which were applicable to large networks. In comparing the proposed multi-layer framework with the module identification methods in single-layer networks, our aim is to investigate whether adding more layers to the proposed method has led to the identification of better modules. The two methods were executed on the co-expression layer and their output modules having an acceptable size were selected to be assessed. Here, we examined the fold change and p-value of differentially expressed genes inside modules. Data distribution for the logarithm of fold change values, student’s t-test p-value and standard deviation of fold change values for the modules are depicted in Fig. 6. The comparison results denote that the approach presented here outperforms the other two methods in terms of unfolding homogenous modules containing genes with a higher change in their expression with low p-values.

## Discussion

In the previous sections, we investigated the relevance of the modules with COAD in terms of functional linkage networks and pathway analyses, and results suggest that the two modules were highly correlated with the disease causality. Therefore, we discussed the candidate biomarkers of the two modules extracted from the survival analysis: *CDC6, RRM2, ORC1, NCAPG, MAD2L1, MCM6, CCNF, CDCA2, ECT2*, and *DEPDC1B*.

**Figure 7:**
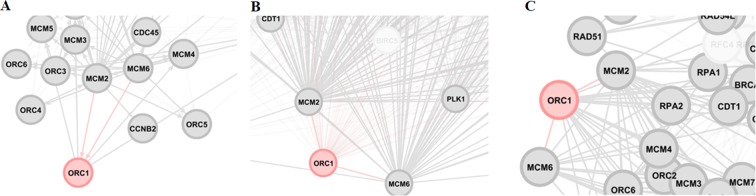
Three-layer overview of genes, namely *ORC1*, *MCM2*, and *MCM6*. (**A**) Regulatory layer. *MCM2* and *MCM6* (from *MCM* complex family) both regulate the transcription of the gene *ORC1*. (**B**) Co-expression layer. All three genes have expression correlations (negative and positive). (**C**) Physical binding layer.

*ORC1* is a protein-coding gene, which is overexpressed in colon cancer*. ORC1* is involved in some critical pathways: *Cell Cycle, DNA Replication, E2F transcription factor network, G1 to S cell cycle control*, and *Retinoblastoma (RB) in* cancer. Gene ontology annotations that are related to this gene include *chromatin binding*. *MCM2* and *MCM6* are the upstream transcription factors regulating the *ORC1* protein factor and are upregulated in the case of cancer; therefore, it could provide some explanations for the upregulation of *ORC1* (Fig. 7). *ORC1* is an important paralog of *CDC6*. The *CDC6* protein is essential for the initiation of DNA replication, which is a crucial phase during the cell division. This protein acts as a regulator at the early steps of DNA replication. It could be localized within the cell nucleus during cell cycle G1 but translocated to the cytoplasm at the initiation of S phase. Among its related pathways, namely *Cell cycle_Role of APC in cell cycle regulation* and *CDK-mediated phosphorylation and removal of CDC6* are more characterized compared with others. Gene ontology annotations that are associated with this gene comprise nucleotide and kinase binding. Among its regulators, *MCM*3*, E2F2, MCM2, E2F1, E2F7, FOXM1, ARID3A* and *MCM7* are all overexpressed in cancer. Correspondingly, *MYC* and *AR* are the other regulators, down-regulated when the cells are transformed into cancer cells.

*CCNF (cyclin F)* is another protein-coding gene, which encodes a member of the cyclin family. Cyclins are regulators of cell cycle transitions through their capability to bind and activate cyclin-dependent protein kinases. It is introduced as a key gene in carcinogenic pathways associated with colorectal adenoma-to-cancer progression^49^. In the same way, *MAD2L1 (Mitotic Arrest Deficient 2 like 1)* is a component of the mitotic spindle assembly checkpoint that prevents the onset of anaphase until all chromosomes are suitably aligned at the metaphase plate. Among its associated pathways, *Mitotic Metaphase and Anaphase* and *Cell cycle_Role of APC in cell cycle regulation* are well-defined and investigated. In a study conducted by Abal *et al*.^50^, they mentioned that *APC* inactivation is associated with abnormal mitosis and concomitant *BUB1B*/*MAD2L1* up-regulation. However, the overexpression of these two genes was correlated with tumor metastasis and poor prognosis of patients with breast cancer^51^. Its expression is amplified in colon cancer as well.

The *CDCA2* gene encodes a subunit of *protein phosphatase 1*, which is associated with cell cycle and has relatively higher expression in cancer conditions. *POU2F2* is a regulator of *CDCA2*, which is down-regulated in colon cancer, and it is a negative regulator of *CDCA2*. Conversely, *POU5F1* is another controller of *CDCA2, which* is overexpressed in cancer and a positive regulator.

Among the proposed gene list, *ECT2 (Epithelial Cell Transforming 2)* is another biomarker previously mentioned that it is involved in breast cancer^52^. Its expression is increased in tumor tissues of patients and involved in the following pathways *RET signaling* and *G-protein signaling_RhoA regulation*. Annotations that are associated with this gene include *protein homodimerization activity* and *GTPase activator activity*. Its role in colorectal cancer is discussed earlier by Chen *et al*.^53^ and Luo *et al*.^54^.

*Condensin* complex is responsible for the condensation and stabilization of chromosomes during mitosis and meiosis. *NCAPG (Non-SMC Condensin I Complex Subunit G)* encodes a subunit of *Condensin* complex, and phosphorylation of the encoded protein activates the complex. *Mitotic Prometaphase* and *Cell cycle Chromosome condensation in prometaphase* are biological pathways in which *NCAPG* plays a role in them. *RRM2 (Ribonucleotide Reductase Regulatory Subunit M2)* is another DEG that it has a crucial impact on the survival rate of patients with cancer. It has been indicated that *RRM2* is involved in the pathogenesis of pancreas adenocarcinoma. However, its role in colorectal cancer has been addressed in several studies^55,56^. *KRAS*-mediated upregulation of *RRM2* is vital for the proliferation of colorectal cancer cell lines^57^.

The protein encoded by *MCM6* is one of the extremely conserved mini-chromosome maintenance proteins (*MCM*) that are necessary for the beginning of eukaryotic genome replication. The MCM complex consisted of this protein, as well as *MCM2*, *MCM*4, and *MCM7*. This complex has been shown to have DNA helicase activity and act as a DNA unwinding enzyme. The phosphorylation of the complex by *CDC2* kinase decreases the helicase activity, signifying a role in the regulation of DNA replication. Huang *et al*. mentioned that the interaction between *RAD*5*1* and *MCM* complex is indispensable for the formation of *RAD51* foci in colon cancer *HCT116* cells^58^. Also, its significance has been shown in other types of cancers^59,60^. Accordingly, the protein *DEPDC1B* has some significant roles in pathways *p75 NTR receptor-mediated signaling* and *Signaling by Rho GTP*ases. An important paralog of this gene is *DEPDC1*, and it is overexpressed in the case of tumor progression. Our computations suggest that the genes mentioned above have a critical role in colon carcinoma; however, additional experimental validations are needed to approve these markers.

## Conclusion

In this research, we employed multilayer networks to model colon adenocarcinoma and investigated its functional core components. We used the transcriptome-to-interactome integrative approach, and different omics data sources to reconstruct disease network and study its foundations. Then, we utilized an extended community detection algorithm and achieved two modules with centroid genes *USF1* and *E2F1*, which are dense subnetworks, and their genes have functional linkage similarities. Both modules showed a significant overlay with COAD-related genes and pathways; however, the module *E2F1* contained statistically meaningful differentially expressed genes, and it had a marked effect on the survival of patients with COAD. We selected suitable DEGs as potential biomarkers and examined their regulatory cascade flow. Results of the literature mining suggest that the candidate genes play roles in critical pathways associated with cell cycle, apoptosis, and COAD progression; however, their role in the development and pathogenesis of cancer should be approved by experimental approaches in the future. The approach employed here is a general framework applicable to other problems in this context; however, the construction of the multilayer network is the core part of the procedure that must be constructed based on the phenotype-specific transcriptomic dataset. Modules extracted in this study are dedicated to colon adenocarcinoma, which should be confirmed experimentally.

## Supplementary information

supplementary information.

supplementary information 2.

supplementary information 3.
