# Supplementary material for: Identification of Key Components in Colon Adenocarcinoma Using Transcriptome to Interactome Multilayer Framework
Source: Sci Rep. 2020 Mar 19;10:4991. doi: 10.1038/s41598-020-59605-z (PMC7081269; doi:10.1038/s41598-020-59605-z)
Supplement: Supplementary file 1 — supplementary information. [file 41598_2020_59605_MOESM1_ESM.pdf]

Identification of key components in colon adenocarcinoma using transcriptome to interactome multilayer framework

Ehsan Pournoor[1], Zaynab Mousavian[2], Abbas Nowzari Dalini[2], Ali Masoudi-Nejad[1*]

[1] Laboratory of Systems Biology and Bioinformatics (LBB), Institute of Biochemistry and Biophysics, University of Tehran, Tehran, Iran

[2] School of Mathematics, Statistics, and Computer Science, College of Science, University of Tehran, Tehran, Iran

***Corresponding Author**

Ali Masoudi-Nejad, Ph.D.
Laboratory of Systems Biology and Bioinformatics (LBB)
Institute of Biochemistry and Biophysics
University of Tehran, Tehran, Iran
E-mail: amasoudin@ut.ac.ir
WWW: http://LBB.ut.ac.ir
Tel: +98-21-6695-9256
Fax: +98-21-6640-4680

Heterogeneous multilayer random walk scores

Genes (Red cell genes play role in COAD according to DisGeNET)	RW_score	all colon genes in DesGeNet
GABPA	1	NAT1
MAX	0.994457721	NAT2
CTCF	0.989474755	NCOA3
SP1	0.977729191	NRIP1
MYC	0.957543092	GATD3A
EGR1	0.858748157	ABCA1
USF1	0.805867697	ACACA
TFAP2A	0.711699802	APCDD1
YY1	0.674073321	MAT2A
E2F1	0.559109168	ACP1
MXI1	0.523567397	MAX
JUN	0.511059135	ACPP
NFYA	0.471297097	MBL2
TFAP2C	0.448314435	ACTB

SPI1	0.422992831	DACT2
HNF4A	0.395942442	MCAM
FOS	0.354604159	MCC
TP53	0.327960543	ACTG1
POU2F1	0.32262165	MMP21
CREB1	0.316875985	MCL1
ARNT	0.313825189	MCM2
PPARG	0.294910256	MCM7
MEF2A	0.273859765	METAP1D
CUX1	0.25092795	ACVR2A
REST	0.250165251	XRCC6P5
EP300	0.249809325	IL17D
NFKB1	0.248182234	MDK
SRF	0.247012762	EPX
PAX5	0.235928205	MDM2
CEBPA	0.23465704	MDM4
GATA1	0.227284283	HIST3H3
RELA	0.22509788	SGO1
ATF2	0.215843799	ME1
STAT1	0.206182946	NDRG2
ELK1	0.206081253	BIRC6
TBP	0.198301724	GATAD2B
POU3F2	0.197640718	PPM1H
AHR	0.191691666	AXIN1
ESR1	0.185996848	MAP3K5
TCF3	0.180302029	AXIN2
JUNB	0.178115625	SLC12A5
STAT3	0.175573295	FZD1
E2F4	0.175013983	FZD7
ZEB1	0.167946306	ADM
CREBBP	0.164132811	ADORA2B
RFX1	0.163370112	MEST
CEBPB	0.154827884	MET
HNF1A	0.154675344	PARP1
E2F2	0.153963492	SCGB2A1
EBF1	0.152031322	MGAT5
SREBF1	0.14816698	MGMT
SREBF2	0.144251792	AFP
AR	0.13876036	ACAN
MEIS1	0.13860782	HECW2
RORA	0.133218081	AGER

GRB2	0.132557075	JAG1
NR3C1	0.130929984	MIF
NFIC	0.130879138	MAD1L1
EGR3	0.126709717	MIP
STAT5A	0.126302944	ZC3H12D
ATF6	0.119896273	NR0B1
SMAD3	0.118167489	MITF
FOXA2	0.115726852	AHCY
EGR2	0.115726852	MLH1
CREB3	0.115523466	AHR
E2F3	0.114099761	AHSG
E2F7	0.11232013	KMT2A
GATA2	0.112218437	AFDN
REL	0.111557431	AKT1
E2F6	0.111404891	FOXO4
RXRA	0.111150658	PLA2G10
PAX6	0.110743886	AKT2
MZF1	0.110032033	NR3C2
ATF1	0.1097778	SPARCL1
MYB	0.108964255	SEMA6A
SMAD4	0.108811715	ALB
STAT2	0.10784563	ALCAM
HDAC1	0.104184675	MME
SP3	0.103981288	MMP1
POU3F1	0.103269436	ULK1
LMO2	0.101032186	MMP2
E2F5	0.100422027	MMP3
TGIF1	0.10037118	ALDH1B1
ATF4	0.100167794	TDRD6
SMAD2	0.098642396	MMP7
FOXJ2	0.097778004	MMP9
IRF1	0.097676311	MMP13
ABL1	0.096811918	MMP14
BRCA1	0.096252606	MMP17
TAL1	0.094778055	AKR1B1
RREB1	0.094676361	MIR675
ATF5	0.093456043	ALK
TFAP4	0.09335435	NR0B2
NFKB2	0.093150964	ALOX5
ATF3	0.092388265	RECK
TLX2	0.092235725	ALOX15

NFIL3	0.0911171	ALPP
PAX2	0.090761174	ALPG
JUND	0.09065948	MPG
KLF12	0.090100168	MPO
NR2F1	0.089947628	CUL4A
RB1	0.089795088	CUL3
FOXA1	0.088930696	LYPD4
NFE2L1	0.088473077	MPZ
HOXA9	0.088167997	MRC1
ETS1	0.08603244	MRE11
BACH1	0.085829054	ABCC1
TOPORS	0.084862968	AMPD3
FOXO1	0.083235877	BIN1
FOSB	0.083235877	STIM2
IKBKE	0.081812173	ANG
CTNNB1	0.080795241	ANGPT1
UBE2I	0.080642701	ANPEP
FOXO4	0.080185082	GIT1
ATF7	0.080083388	GEMIN2
CDK2	0.079930849	LIN28B
IRF7	0.079422383	MUC15
TCF4	0.078557991	ANXA2
NR2F2	0.078456297	RANBP3
BPTF	0.076219047	CIP2A
EWSR1	0.075812274	ANXA5
PRKACA	0.075100422	ABCB5
NFYC	0.074337723	ANXA6
DDIT3	0.072354706	RGCC
POU2F2	0.072151319	BMF
CSNK2A1	0.071592007	ENC1
FOSL1	0.071388621	APAF1
FOXF2	0.069914069	MMP23B
NFE2	0.068947984	MMP23A
MIF	0.068744598	APC
PBX1	0.067931052	HIPK2
GATA3	0.067880205	APEX1
SRC	0.067625972	BIRC3
YWHAZ	0.067168353	XIAP
SMAD1	0.06676158	BIRC5
ZBTB6	0.065541262	DGKZ
SUMO1	0.064117557	UPRT

HSF1	0.063965018	APOA1
BACH2	0.063761631	APOBEC1
HLF	0.063761631	MSH2
CDC5L	0.063710785	MSH3
IKBKG	0.063558245	MSI1
FOXC1	0.063253165	APOE
POU5F1	0.062643006	MAGEE1
YWHAG	0.062185387	APP
HIF1A	0.061931154	APRT
CDK1	0.061727767	FAS
STAT6	0.061422688	FASLG
SMAD9	0.061066762	LGR5
PATZ1	0.061066762	AQP1
MAPK1	0.060456602	CWC22
ZFP36	0.059948136	AQP5
VSX2	0.05959221	AR
FOXO3	0.058727818	AREG
BCL6	0.058676972	PDLIM4
MAPK14	0.058524432	MSN
BHLHE40	0.058524432	BCO1
NKX2-2	0.057812579	RHOA
ALX1	0.05766004	MST1
PAPOLA	0.057558346	MST1R
FOXD1	0.057304113	MT1F
NFYB	0.056083795	SETD2
ATXN1	0.054761784	ARNT
TRAF2	0.054253318	MT2A
HDAC2	0.053999085	ARSA
SOX9	0.053846545	STS
EGFR	0.053185539	RUVBL1
NKX6-1	0.052626227	MT-CO1
APC	0.052524533	MT-CO2
KRTAP10-3	0.052117761	GSTK1
SIN3A	0.051761835	MT-CO3
KAT2B	0.051710988	NUDT1
STAT4	0.051558448	MTHFD1
HMGB1	0.050897442	MTHFR
PARP1	0.050846596	ASCL2
SMEK1	0.050846596	TP63
UBC	0.050744903	PYCARD
MDFI	0.050744903	UNC5C

XRCC6	0.050744903	ASPH
HSF2	0.050439823	MYLIP
NFATC1	0.050439823	ZBTB10
NFATC2	0.049982204	MTR
VHL	0.049321198	AKR1C3
FYN	0.049321198	MTRR
GSK3B	0.049270351	UHRF1
MAPK3	0.049168658	SOCS1
CLMN	0.048253419	TNKS
EEF1A1	0.048202573	DDX53
RXRB	0.04794834	ATF3
STAT5B	0.047897493	IRS2
EIF3E	0.047846647	ATHS
NKX3-1	0.0477958	ATM
PRKCA	0.0477958	HAMP
WDR20	0.047541567	ATOH1
POU3F3	0.04749072	AOC4P
WT1	0.047338181	MTX1
ZIC2	0.047134794	MUC1
TRAF6	0.047134794	BECN1
ARID5B	0.046117862	MUC2
ALB	0.045761936	MUC3A
SETDB1	0.04571109	ATP2A3
HMGA1	0.045609396	MUC4
FOXO3B	0.04555855	MUC5AC
ETS2	0.045304317	ATP2B2
MAPK6	0.04525347	ATP2B3
NFKBIA	0.045050084	ATP2B4
RELB	0.044897544	MUTYH
WNK1	0.044897544	MYB
UBQLN4	0.044745004	MYBPC3
TAF12	0.044643311	MYC
SLC22A11	0.044592465	MYCN
ARRB1	0.044541618	ZNF410
EHMT2	0.044490771	MYD88
NPM1	0.044134845	GADD45B
GOLGA2	0.044083999	ABCC3
POLR2A	0.043829766	MIR133B
NOTCH2NL	0.043473839	MIR135B
FOXL1	0.043372146	TNFRSF25
KRT40	0.043270453	MIR324

COPS5	0.043270453	EED
YBX1	0.04316876	MIR345
RARA	0.04316876	MIR346
SOCS3	0.042965373	ATP7A
TRIM27	0.042914527	MIR342
NIFK	0.042914527	MIR335
RRS1	0.04286368	MYO1A
APP	0.042202675	ATR
SRY	0.042202675	RIPK1
KAT5	0.041846748	MIR374A
ATM	0.041795902	HCA1
CSNK2A2	0.041795902	TNFSF12
KRTAP10-7	0.041795902	AVP
HDAC4	0.041643362	TNFSF10
USF2	0.041236589	TNFSF9
AMY2B	0.041084049	MIR196B
NUP62	0.041084049	ADAM9
DIMT1	0.040372197	AZGP1
HTT	0.040168811	B2M
SOD2	0.039914578	BAAT
PPARA	0.039812885	BAD
FOXD3	0.039609498	BAG1
SMURF2	0.039558652	TNFRSF6B
HLA-B	0.039202725	BARD1
RIPK2	0.039202725	BAX
ATF7IP	0.039151879	MIR362
HCFC1	0.038948492	MIR363
HDAC3	0.038846799	CEACAM6
ZBTB16	0.037982407	NCAM1
SMARCA4	0.037982407	CCND1
GTF2B	0.037779021	BCL2
SFN	0.037575634	BCL2L1
CITED2	0.037524788	BCL2L2
CITED4	0.037372248	TNFRSF10D
TRIP13	0.037219708	TNFRSF10C
FHL2	0.037118015	TNFRSF10B
SKIL	0.036965475	TNFRSF10A
BMPR2	0.036863782	BCL9
VIM	0.036711242	SUCLA2
GTF2I	0.036711242	FGF18
RUNX1	0.036609549	BDNF

MAPK8	0.036558702	DEFB103A
CDKN1A	0.036101083	BGLAP
APPBP2	0.035948543	CES2
SMARCC2	0.035745157	BGN
PML	0.035643464	CEACAM1
RBPMS	0.035440077	IQGAP1
MEOX2	0.035440077	NRP2
AKT1	0.035236691	DRG1
FXR2	0.035084151	NRP1
EIF2AK2	0.034779072	NEDD4
DAXX	0.034728225	CXCR5
NCOA6	0.034626532	NEDD9
TERT	0.034575685	CFLAR
TRIP6	0.034473992	BMI1
MDM2	0.034473992	CCN4
PPP1CA	0.034219759	BMP1
CDC42	0.034067219	HDAC3
ARRB2	0.033863833	BMP2
NR4A1	0.033863833	PROM1
ACTB	0.033863833	BMP4
POGZ	0.0336096	BMPR1A
NCOA1	0.0336096	KAT2B
GFI1B	0.033507907	BMPR2
SUMO2	0.033101134	AKAP4
NCOR2	0.032999441	BNIP3
VEGFB	0.032948594	NEUROD1
RUVBL1	0.032846901	NR1I2
FOXI1	0.032745208	LOC101930123
IKBKB	0.032643515	BNIP3L
TGFBR1	0.032541821	NF1
PCNA	0.032440128	PER3
CTBP1	0.032338435	BRCA1
UBQLN1	0.032338435	PER2
APOA1	0.032236742	BRAF
SMAD7	0.032236742	BRCA2
FASLG	0.032185895	NFATC1
PLK1	0.032135049	NFATC2
HSP90AA1	0.032135049	IER5L
RGS1	0.032135049	BSG
HSPA8	0.032084202	ARHGEF7
PSEN1	0.031982509	NFE2L2

TCF12	0.031931662	SPHK1
RNF31	0.031779122	SQSTM1
FAM102B	0.031626583	KLF5
TLE1	0.031524889	NFKB1
CSNK2B	0.031423196	NFKB2
THRB	0.03137235	NFKBIA
RAC1	0.031321503	BUB1
TRAF1	0.03121981	TSPO
PRKACB	0.03106727	NGF
COPS6	0.030863884	PRPF4B
FHL3	0.030863884	JAZF1
SLX4	0.03076219	NID1
LMNA	0.030711344	C3
MITF	0.030558804	BCL10
CALCOCO2	0.030507957	B4GALNT3
CITED1	0.030507957	NME1
ARRDC2	0.030355418	NME2
PSMA3	0.030304571	NME4
ESR2	0.030253725	MBD4
SIRT1	0.030202878	MBD2
PLIN1	0.030202878	NOS2
MCC	0.030050338	NOTCH1
YWHAB	0.030050338	NOTCH2
VDR	0.030050338	CA1
HMGB2	0.029999492	CA2
CASP3	0.029999492	CA8
PIN1	0.029999492	CA9
PIAS1	0.029796105	NPHS1
XRCC5	0.029694412	NPM1
RPS27A	0.029643565	SLC25A20
RUVBL2	0.029541872	CALB2
PDHX	0.029541872	HSPB3
SIRT5	0.029541872	NRAS
RBBP4	0.029491026	ZBTB7C
SLC35E4	0.029389332	CALCR
TOP2A	0.029338486	CALD1
SET	0.029287639	NRCAM
AC074212.6	0.029287639	YBX1
RBL1	0.029185946	F2RL3
NCOR1	0.029185946	CALR
NFATC4	0.029135099	NT5E

PRKDC	0.029135099	CALU
MEF2C	0.028880866	NGB
LHX3	0.02883002	NTRK1
YWHAH	0.028779173	NTRK2
EZH2	0.028728327	ROR2
SMC1A	0.028728327	NTS
DEFB1	0.028728327	NTSR1
KIF7	0.028575787	SOCS3
SKP1	0.02852494	CAST
LCK	0.028474094	NR4A2
HSPA1A	0.028474094	SLC2A14
PGR	0.028423247	CASP2
NCK1	0.0283724	CASP3
POU3F4	0.028118167	CASP6
TSC22D1	0.028016474	CA13
MED1	0.027965628	CASP8
RP11-279F6.1	0.027863935	PKD2L1
ITGB2	0.027863935	CASP9
MED31	0.027863935	OAS1
DAZAP2	0.027813088	CASP10
IRF4	0.027762241	OAS3
MYOD1	0.027711395	CASR
TUBA1A	0.027660548	FAM3B
OAS3	0.027406315	SPAG9
TRIM23	0.027406315	CAV1
AES	0.027304622	ODC1
CFTR	0.027304622	TLR9
UBA52	0.027304622	RUNX2
ID2	0.027152082	GPRC5A
CEP70	0.027152082	RUNX1
TAF10	0.027101236	RUNX1T1
GATAD2B	0.027101236	PRC1
PIK3R1	0.027101236	RUNX3
TSG101	0.027050389	PIAS2
SMC3	0.027050389	OGG1
RP11-397G17.1	0.026999542	CBR1
GABPB1	0.026999542	CBS
CALM1	0.026948696	CLDN2
MTUS2	0.026897849	CLDN1
ITGB1	0.026897849	CCK
BRCA2	0.026745309	CCKBR

KPNA1	0.026694463	CCNA2
POLR2L	0.026643616	CCNB1
MAPK9	0.026643616	LOC107984148
FLI1	0.02659277	CCNC
FN1	0.026541923	CERS5
NCOA3	0.02644023	MIR551A
EXOSC4	0.026338537	CCNH
KRTAP10-9	0.02628769	TBX18
CRK	0.02628769	USP14
UPF1	0.026185997	USP10
SDCBP	0.02613515	OSM
TFCP2	0.026084304	CLDN11
HNRNPK	0.026084304	CD9
HSP90AB1	0.026033457	P2RX5
GLI1	0.026033457	MIR570
RBL2	0.025931764	CD28
CCDC85B	0.025880917	CD80
ATN1	0.025880917	PEBP1
PBX2	0.025830071	CCNE2
APOB	0.025830071	CBFA2T2
UBB	0.025779224	CD36
TAF1	0.025779224	PAEP
CCND1	0.025779224	NEMF
TP73	0.025626684	PRDX1
TAF7	0.025626684	DYRK1B
ID3	0.025575838	CD40
YWHAE	0.025575838	SERPINE1
OLIG2	0.025524991	CD40LG
PRKCD	0.025423298	CD44
NFATC3	0.025423298	PAK1
TAF9	0.025321605	MIR451A
NCOA2	0.025321605	MIR608
BCL2	0.025270758	MIR612
USP22	0.025169065	CD68
SOD1	0.025016525	REG3A
ERBB3	0.024914832	PRKN
CDKN2A	0.024863985	CD151
KPNA2	0.024813139	SCAF11
NFKBIB	0.024762292	PAWR
CDH1	0.024660599	LPAR2
DNMT1	0.024609752	PAX2

CCNDBP1	0.024558906	CDK1
MLH1	0.024508059	SLC5A8
ACSL3	0.024406366	OSMR
PTK2	0.024406366	CDC25A
PCBD1	0.024304673	CDC25C
MAP3K3	0.024304673	MIR490
STAG1	0.024253826	DDX21
KPNB1	0.02420298	CDC42
TTYH1	0.02420298	CDH1
LZTS2	0.024152133	MIR146B
NRIP1	0.024152133	CDH2
GADD45A	0.024101286	HACD1
AGTRAP	0.024101286	CDH11
TEAD1	0.024101286	DCLK1
CTBP2	0.02405044	CDH13
DDB1	0.02405044	MIR498
MME	0.023999593	PCNA
RSF1	0.023948747	MIR202
AURKA	0.02374536	CDK2
ETV4	0.02374536	MIR492
PLCG1	0.023694514	MIR493
SMAD5	0.023643667	CDK4
GABRD	0.023643667	CDK5
ELF1	0.023643667	AURKB
RBBP7	0.02359282	MIR494
IFIH1	0.02359282	CDK6
APOH	0.02359282	XPR1
CLTC	0.023541974	FCMR
F2	0.023541974	CDK8
TK1	0.023491127	CDKN1A
MCM7	0.023389434	CDKN1B
DYNLL1	0.023338587	MTA2
CIITA	0.023287741	CDKN1C
MCM2	0.023236894	CDKN2A
EPAS1	0.023236894	COMMD3-BMI1
PRKACG	0.023236894	CDKN2B
TOP1	0.023186048	CDKN3
ZHX1	0.023135201	PDCD1
PRNP	0.023033508	PTTG1
RPRD2	0.022982661	IL32
PSMC5	0.022982661	CDX1

MYBL2	0.022931815	PDE3B
PQBP1	0.022880968	CDX2
IGHG1	0.022830122	CEACAM5
HIPK2	0.022779275	MSC
TRRAP	0.022779275	CEBPB
CHD8	0.022779275	GCNT3
SNRPB	0.022677582	NAPEPLD
PSMB3	0.022677582	MIR502
IRF3	0.022677582	MIR503
CSRNP1	0.022626735	RPS6KA5
HSPA5	0.022575889	PDGFRB
ST5	0.022525042	AIMP1
PRKCZ	0.022525042	MIR506
IL2	0.022474195	MIR483
NR2C2	0.022423349	CETN1
BLOC1S1	0.022423349	PDLIM7
SQSTM1	0.022372502	CFL1
APOC3	0.022372502	ENPP2
NR5A1	0.022372502	PDPK1
ZEB2	0.022321656	CFTR
HDAC5	0.022321656	SERPINF1
DNAJC14	0.022270809	CEACAM3
BARD1	0.022219962	CEACAM7
HGS	0.022219962	BLNK
IL2RB	0.022118269	CEACAM4
KRTAP10-8	0.022118269	KLF14
LNX1	0.022118269	PER1
RNPS1	0.022016576	GPR55
THPO	0.021965729	PF4
HRAS	0.021965729	CHAT
ONECUT1	0.021965729	CFP
COIL	0.021914883	CHEK1
TNF	0.021914883	CHI3L1
SIN3B	0.021914883	CHKA
KPNA3	0.021914883	KLF4
RING1	0.021864036	TICAM2
CHD3	0.02181319	CHRM3
PTPN6	0.02181319	SRPRB
PPP2R1A	0.02181319	PGK1
RXRG	0.02181319	CHRNA4
PRPF40A	0.021762343	TERF2IP

SOX8	0.021609803	ABCB1
JAK2	0.021609803	GLP2R
SLC9A3R1	0.021609803	CKB
IKZF1	0.021609803	CISH
ACTA1	0.021558957	SLC9A3R2
SNRPA	0.021558957	SCAF1
NCL	0.021558957	HNRNPA1P10
PLEKHF2	0.02150811	SENP1
SF3B1	0.021457263	SERPINB5
MTIF2	0.021406417	SERPINB6
FLNA	0.021406417	KL
RIF1	0.021406417	NOXO1
TCEB1	0.02135557	ADIPOQ
FAM9B	0.02135557	ICOS
GOPC	0.02135557	PLAA
UTRN	0.02135557	CUEDC2
TOP2B	0.021304724	CLCN4
PUF60	0.021304724	COX5A
SIAH1	0.021253877	PIGR
ATR	0.021253877	CLU
APAF1	0.021253877	CLIC1
DRAP1	0.021101337	SASH3
KRT31	0.021101337	PIK3CA
CBS	0.021050491	PIK3CB
SRSF1	0.021050491	PIK3CD
PRKCH	0.021050491	PIK3CG
NFE2L2	0.021050491	PIK3R1
PPP2CA	0.021050491	PIK3R2
RAPGEF2	0.021050491	CLN3
EPB41	0.021050491	PIN1
PROX1	0.020999644	PIP
MTA1	0.020897951	GRAP2
E2F8	0.020847104	SELENOF
AIP	0.020847104	RAD54B
SMARCC1	0.020847104	PKD2
FBXO11	0.020847104	RETREG1
CALM2	0.020745411	PKM
RAD51	0.020745411	CTHRC1
HSPD1	0.020694564	PLA2G1B
TCEB2	0.020643718	PLA2G2A
PIAS4	0.020592871	PLA2G4A

RUNX1T1	0.020592871	KRT20
ZMYM4	0.020542025	SPDEF
ZNF24	0.020542025	HAND1
EDF1	0.020542025	BAMBI
PDZK1	0.020491178	SAC3D1
TAF4	0.020440332	CCR1
KHDRBS1	0.020440332	PLAT
GNB2L1	0.020440332	NTN1
USP7	0.020440332	PLAU
PAK1	0.020389485	EEF2K
TF	0.020389485	PLAUR
UBXN1	0.020389485	CCR5
PTPRC	0.020389485	CCR7
ASCC2	0.020287792	ABCG2
MCM4	0.020287792	PLCG1
MED14	0.020287792	TNFAIP8
YWHAQ	0.020236945	LTB4R
PRKCB	0.020236945	PLD1
PRICKLE3	0.020236945	PLD2
KRTAP10-5	0.020186099	PLG
NFAT5	0.020186099	RMC1
SOX13	0.020084405	PRDX5
TSC22D4	0.020084405	BACE2
MATR3	0.020084405	NME7
TP53BP1	0.020084405	PLK1
TGFBR2	0.020084405	AIM2
POLR2H	0.020084405	MIR429
COMMD5	0.020033559	EIF2AK3
HPSE	0.020033559	PLS3
LYN	0.019931866	CNR1
PTPN11	0.019931866	ARMC8
GTF2F2	0.019881019	SERTAD1
CREB3L1	0.019881019	COL2A1
TFDP1	0.019881019	PMP22
CBL	0.019881019	PMS1
MAPK13	0.019881019	WHRN
CDK4	0.019779326	COL4A6
LEF1	0.019779326	PMS2
IGHA1	0.019779326	COL11A2
C3	0.019779326	COMT
RBX1	0.019779326	OBP2A

ERG	0.019779326	DLL4
LYAR	0.019779326	RNF19A
DHX9	0.019728479	MAP3K8
WWP1	0.019728479	GDF15
SMARCB1	0.019677633	POLB
SNRPD2	0.019677633	TBPL1
PIAS3	0.019626786	POLD1
TFIP11	0.019626786	UGT1A7
XPO1	0.019626786	UGT1A6
PLG	0.019575939	RRM2B
NRP1	0.019575939	DEFB104A
BANF1	0.019575939	PLA2G3
GABPB2	0.019525093	EGLN1
CREM	0.019525093	BAG3
RPS9	0.019525093	PTGES
CREB3L3	0.019525093	COX8A
EIF1B	0.019474246	SOSTDC1
RPL7	0.019474246	UGT1A9
SNAI1	0.019474246	NOX4
MMP2	0.0194234	WWTR1
ADAMTSL4	0.0194234	FSD1
ARF6	0.019321706	H2AFY
CAV1	0.019321706	SAMHD1
RARB	0.01927086	POU5F1
PPARGC1A	0.019220013	CLDN7
SNW1	0.019220013	GATA5
GPATCH8	0.019169167	PPARA
SMARCA2	0.01911832	CPOX
BANP	0.01911832	PPARD
PLEKHA5	0.01911832	PPARG
SLC22A7	0.01911832	BCAR1
RHOG	0.019067473	MED1
AMBP	0.019016627	PPBP
RPL8	0.019016627	CRABP1
BTK	0.01896578	PPIB
MAP3K7	0.01896578	CLOCK
ERBB2	0.01896578	CRAT
TET2	0.018914934	CREBBP
CEBPD	0.018914934	CREM
DDC	0.018914934	MIR106B
NPC2	0.018914934	MIR107

CCDC34	0.018864087	MIR106A
RPL35	0.01881324	CRK
RAN	0.01881324	TIPARP
SPIB	0.01881324	CRP
H2AFZ	0.01881324	PPOX
PRKAB1	0.01881324	HAPLN1
RPS6KA1	0.018762394	PPP1R1A
NMI	0.018762394	CRY1
FLOT1	0.018711547	CRY2
TFAM	0.018711547	KRT23
SHC1	0.018609854	MIR130A
BRF1	0.018609854	UGT1A1
XRN1	0.018609854	RNF14
DDB2	0.018609854	MIR126
NT5C2	0.018609854	SNED1
RPLP1	0.018559007	RIN1
ATG5	0.018559007	PIM3
PSMD13	0.018559007	NCOR2
RPS3A	0.018559007	PPP2R1B
PSTPIP1	0.018508161	MIR142
DDX5	0.018508161	MIR143
PNMA1	0.018457314	MIR140
RPL23	0.018457314	MIR141
RHOXF2	0.018457314	PTPA
SCAND1	0.018457314	CRYZ
TLE4	0.018406468	KLK4
THRA	0.018406468	MIR137
HSPB1	0.018406468	MAPK14
MCM6	0.018355621	UBXN11
SMARCE1	0.018355621	CSE1L
ESRRG	0.018355621	CSF1
PABPC1	0.018355621	CSF1R
PHB	0.018355621	CSF2
C4BPA	0.018304774	MIR147A
ROCK1	0.018253928	MIR145
DISC1	0.018253928	CSF3
KLC1	0.018253928	CYP2R1
USP34	0.018253928	CSK
BAI2	0.018203081	MIR155
SDHAF2	0.018152235	PRB1
WDYHV1	0.018152235	MIR182

CDK7	0.018152235	CSNK1A1
FKBP2	0.018101388	MIR17
RAD21	0.018101388	MIR18A
CDK9	0.018101388	CSNK2A1
GAPDH	0.018050542	ATRNL1
PRKCI	0.018050542	MIR192
TOM1L1	0.018050542	PRH1
KAT7	0.017999695	PRH2
UQCRQ	0.017999695	IL22
CEBPG	0.017999695	PRKAA1
HNRNPU	0.017999695	PRKAA2
FTSJ1	0.017948848	PRKAB1
CASP8	0.017948848	CST1
CBX3	0.017948848	MIR195
SMARCD1	0.017948848	MIR20A
TOLLIP	0.017898002	CST5
MYO18A	0.017898002	MIR200A
TRIB3	0.017847155	MIR19B1
PRAP1	0.017847155	PRKAR1A
GLIPR2	0.017847155	MIR19A
CXCR4	0.017847155	MIR21
RANBP2	0.017847155	PRKCA
NME1	0.017847155	LOC100507703
SGTA	0.017796309	CTAA1
SMURF1	0.017796309	PRKCB
RUNX3	0.017745462	PRKCD
MCM3	0.017745462	MIR203A
SNIP1	0.017745462	UBXN2A
PTTG1	0.017745462	MIR200B
MED23	0.017694615	MBL3P
PTP4A3	0.017694615	MIR200C
EXOSC8	0.017643769	KDM4A
PPP1R15A	0.017643769	MIR214
PLSCR1	0.017643769	CTH
HAX1	0.017643769	PRKD1
SHPRH	0.017643769	XAF1
CUL1	0.017592922	MIR215
NOTCH1	0.017592922	PRKCZ
TADA3	0.017592922	MIR210
RCOR1	0.017592922	CTNNA1
SUMO3	0.017592922	PRKDC

GABARAPL2	0.017542076	POLDIP2
PPP1CC	0.017542076	MAPK1
MT2A	0.017542076	MIR22
PPARD	0.017491229	CTNNB1
MED8	0.017491229	MAPK3
NAA10	0.017491229	CTNND1
VPS28	0.017440382	MAPK7
CCT3	0.017440382	MAPK8
BIN1	0.017440382	MIR25
CSNK1A1	0.017440382	MAPK9
FATE1	0.017440382	MIR26A1
LDOC1	0.017440382	RIMS2
PPP4C	0.017440382	CTSB
GATA4	0.017440382	MAP2K1
WDR48	0.017389536	MIR23A
RPS19	0.017389536	MIR23B
HBP1	0.017338689	CTSE
SAP30	0.017338689	MAP2K5
RPL28	0.017287843	MIR224
TNFAIP3	0.017287843	MAP2K7
UBXN7	0.017287843	MIR27A
AC133528.2	0.017287843	MIR27B
GEM	0.017287843	MIR26B
HDAC9	0.017236996	MIR30C1
CDC25A	0.017236996	MIR302A
RNF2	0.017236996	CUX1
MAPK10	0.017186149	MIR30A
FBL	0.017186149	CXADR
PTN	0.017186149	MIR29B1
RPL36	0.017186149	MIR29B2
RPS20	0.017186149	CYB5A
TUBB	0.017186149	PPP1R12C
SOX4	0.017135303	PRODH
MAPK11	0.017135303	MIR320A
HCST	0.017135303	MIR30E
INSR	0.017135303	PROX1
NDUFA3	0.017135303	MIR31
OTUB1	0.01703361	MIR30C2
KIR3DL1	0.01703361	CYBB
RABAC1	0.01703361	DCAF1
HMG20A	0.01703361	MIR34C

SPEN	0.01703361	SART3
ALMS1	0.016982763	HDAC9
SMARCA5	0.016982763	MIR34A
UBE2M	0.016931916	CYP1A1
MTA2	0.016931916	MIR34B
PRPF4B	0.016931916	CYP1A2
RPS6KA3	0.01688107	SETD1A
CHUK	0.01688107	CYP2A6
NUTF2	0.01688107	PRSS1
BTRC	0.01688107	MIR93
SMG1	0.01688107	NLRP12
CCNA2	0.01688107	KLK7
PRKCE	0.01688107	KLK6
UBE2D2	0.016830223	ARHGAP24
MFAP3	0.016830223	SAMD9
RPS13	0.016779377	CYP2D6
EIF6	0.016779377	CYP2E1
TCERG1	0.016779377	DACOR1
LDB1	0.016779377	PSG2
VEGFA	0.016779377	CYP3A4
RPL24	0.016779377	PCLAF
PTMA	0.01672853	CDHR2
MAGEA11	0.01672853	NOC2L
TKT	0.01672853	CYP7A1
MMP9	0.01672853	BCLAF1
RANBP9	0.016677683	CYP24A1
ACVR1	0.016677683	COLCA2
NR2F1-AS1	0.016677683	CYP27B1
UBE2D3	0.016677683	ESRP1
USP37	0.016677683	LAMA1
RPS8	0.016626837	PSMB6
MRPL28	0.016626837	PSMB8
DYNC1LI2	0.016626837	CD55
ARHGDIB	0.016626837	DAG1
PPP1R12A	0.01657599	MS4A12
PRDM1	0.01657599	SCRN1
KAT2A	0.01657599	SNRK
SYK	0.01657599	PSMD9
NR0B2	0.01657599	PSMD10
CCDC155	0.01657599	BIRC7
RYBP	0.016525144	KEAP1

PIK3CA	0.016525144	PTBP1
RARG	0.016525144	DCC
BCL3	0.016525144	PTEN
CD2BP2	0.016525144	ARHGAP11A
LSM10	0.016525144	DCN
DDX20	0.016474297	PTGDS
SMN1	0.016474297	PTGER1
UNC119	0.016474297	ACE
LRIF1	0.016474297	ARHGEF17
CBX8	0.016474297	PTGER3
GSK3A	0.016474297	PTGER4
BCL9L	0.01642345	NSUN2
HIST3H3	0.01642345	DDB1
DLG4	0.01642345	DDB2
LAMA1	0.01642345	PTGIS
MCM5	0.01642345	AKR1C1
PROS1	0.01642345	PTGS1
ILF3	0.016372604	RNF43
BABAM1	0.016372604	GADD45A
PIAS2	0.016372604	PTGS2
HCLS1	0.016372604	ZEB2
ANXA1	0.016372604	PTHLH
SF3B3	0.016321757	DDIT3
NOP16	0.016321757	PTK2
TMEM173	0.016321757	DDX5
RPL18	0.016321757	PTK6
MAP1LC3B	0.016220064	CYP2W1
CDK5	0.016220064	CABLES1
NAA38	0.016220064	PTK7
PHLDA3	0.016220064	DECR1
KEAP1	0.016220064	DEFB4A
SNRPD3	0.016220064	PTPN1
FOXN3	0.016169217	BCL2L12
PSMD4	0.016169217	TIMM8A
THAP1	0.016169217	PTPN3
HSPA4	0.016169217	PTPN6
RPL13	0.016169217	PTPN11
HSPB9	0.016169217	PTPN12
EXOSC10	0.016118371	GSDME
SATB1	0.016118371	PTPN13
EEF1G	0.016118371	RHOBTB1

TNFRSF1A	0.016118371	PTPRG
MSH2	0.016118371	PTPRJ
TP63	0.016118371	NUAK1
MEF2D	0.016118371	PTPRM
EEF1D	0.016118371	PLA2G2D
LST1	0.016067524	CXCL17
WTAP	0.016067524	DHCR7
GMFG	0.016067524	DHFR
TMA7	0.016067524	WDR20
LIN9	0.016067524	IL21
BAG6	0.016067524	PVT1
SSRP1	0.016016678	CYB5R3
TRIM29	0.016016678	NQO1
DVL2	0.016016678	DIAPH1
CEBPE	0.016016678	PXN
NPAT	0.016016678	DLD
SMG6	0.016016678	SESN2
PLEKHO2	0.016016678	DLEC1
DLG1	0.016016678	DMBT1
RPS14	0.015965831	SLC52A2
ARHGAP15	0.015965831	DNAH8
RPL11	0.015965831	MVP
RPL27	0.015965831	MUL1
MAP2K2	0.015965831	DNASE1
ERH	0.015914984	FGF19
DICER1	0.015914984	MED12
ASB2	0.015914984	TRAPPC9
ATXN7	0.015813291	NR1I3
RBPJ	0.015813291	CERS4
SENP1	0.015813291	NR1H4
HABP4	0.015813291	ADIPOR2
CCNT1	0.015813291	IL23R
DAB2	0.015762445	RAC1
NFIX	0.015762445	RAC2
RPS3	0.015762445	DNM2
ILK	0.015762445	DNMT1
RPL30	0.015762445	FERMT3
ARID2	0.015762445	DNMT3B
UBE2D1	0.015762445	RAD21
MCRS1	0.015660752	FOXP3
USHBP1	0.015609905	RAD51

APOA2	0.015609905	HNRNPDL
APEX1	0.015609905	CRISPLD2
SPTLC1	0.015609905	RAF1
CCS	0.015609905	YPEL3
TUBA4A	0.015559058	DPEP1
ANK3	0.015559058	RAG1
PIK3C3	0.015559058	CASP8AP2
MYCN	0.015559058	DPP4
RPS7	0.015559058	MIR7-3HG
RASA1	0.015559058	RAN
FAU	0.015559058	DPYD
ACTL6A	0.015508212	EGLN3
FAM78A	0.015508212	DPYS
PINX1	0.015508212	RANBP2
RPS10	0.015508212	INHBE
VAC14	0.015508212	RAP1A
ANKRD18EP	0.015457365	SLC26A3
DAB1	0.015457365	RAP1GAP
ZNF581	0.015457365	RIOK1
RNF111	0.015457365	SLC52A1
CEP76	0.015457365	RARB
NIF3L1	0.015406519	ARID3A
A2M	0.015406519	HDAC6
SYNCRIP	0.015406519	ATN1
CHMP2A	0.015406519	RARRES1
RPL14	0.015406519	DSC2
SUFU	0.015406519	PDCD6
PRKAB2	0.015406519	RASA1
RPLP2	0.015355672	BCL2L11
HCK	0.015355672	DSG2
CLTB	0.015304825	RB1
ZNF770	0.015304825	RBBP6
NRF1	0.015304825	SLC26A2
PFDN5	0.015304825	RBBP8
RNF169	0.015304825	HBEGF
DST	0.015304825	RBM3
POLR2J	0.015304825	DTYMK
PRR12	0.015304825	CHAF1A
RPL29	0.015304825	NLRX1
RUNX2	0.015253979	DVL2
CYP3A4	0.015253979	ASCC1

ZNF446	0.015253979	RTL10
MDM4	0.015203132	RCN1
NDUFA13	0.015203132	SLFN12
DUSP6	0.015203132	OPN1LW
BOD1L1	0.015203132	AGGF1
CYHR1	0.015203132	TMED7
ERBB2IP	0.015152286	RDX
TMF1	0.015152286	ABCB6
GLI3	0.015152286	E2F1
ENTPD5	0.015101439	GALNT12
KANSL1	0.015101439	E2F4
BCL2L1	0.015101439	REN
UBR1	0.015101439	PIWIL2
CBX5	0.015101439	RET
MRPL52	0.015101439	DNAJB8
HNRNPF	0.015101439	PRRT2
TRIM28	0.015050592	TYMP
PRKG1	0.015050592	TRIM27
NFKBIE	0.015050592	USH1C
PSMD7	0.015050592	EDIL3
POLA1	0.015050592	AAGAB
RGS2	0.014999746	GMNN
MED19	0.014999746	LPAR1
ZNF587	0.014999746	LIN28A
CARD16	0.014999746	RGS10
GIT1	0.014999746	EDN1
ZFR	0.014999746	EDN2
RPS6KB1	0.014999746	EDN3
EGLN3	0.014999746	EEF1A1
YTHDC1	0.014948899	EEF1A2
EEF1A1P5	0.014948899	RAD50
GTF2H1	0.014948899	LOC102723996
RAPH1	0.014948899	FEM1B
EXOSC5	0.014948899	CNNM4
AQR	0.014948899	RMDN3
MAFK	0.014948899	GAL
MARK3	0.014898053	EEF1B2P2
RPS2	0.014898053	FSD1L
GTF2F1	0.014898053	RNASE3
CCNB1	0.014898053	ADIPOR1
KDM5B	0.014898053	RNASEL

CDK16	0.014898053	EFNB2
CHEK1	0.014898053	EGF
CBX4	0.014898053	MEGF8
TSSC4	0.014898053	EGFR
DEK	0.014898053	EGR1
MAP3K8	0.014898053	GATD3B
PRPF31	0.014847206	CEBPZ
CCNE1	0.014847206	ATAD3A
PRMT1	0.014847206	RMDN1
GFAP	0.014847206	DPPA4
ATF6B	0.014847206	SIGIRR
AKT3	0.014847206	RNU1-4
CPSF2	0.014796359	EIF2S1
CDX2	0.014796359	PDIK1L
ATP5J2	0.014796359	EPHA2
RHOA	0.014796359	EIF4E
PHC3	0.014796359	EIF4EBP1
RPL7A	0.014745513	NAT10
CNOT1	0.014745513	EIF5A
FGFR4	0.014745513	MOB1A
GNAI1	0.014745513	TENM1
SARDH	0.014745513	INSIG2
CFL1	0.014745513	ELANE
KNG1	0.014694666	TLE6
SLC19A1	0.014694666	ELAVL1
DDX3X	0.014694666	ING4
NDUFA7	0.014694666	SDF4
NHP2L1	0.014694666	RORA
SAT1	0.014694666	YY1AP1
NOTCH3	0.014694666	ELK1
SHBG	0.014694666	ROS1
TRIM24	0.01464382	MYEOV
SKP2	0.01464382	NAA40
RASSF1	0.01464382	LGR6
AKT2	0.01464382	NME6
HNRNPD	0.01464382	GREM1
PCBP1	0.01464382	MARK2
SMC4	0.01464382	CTTN
SUV39H1	0.01464382	ENO1
WDR5	0.01464382	LEF1
JMJD1C	0.014592973	RPE65

ELF3	0.014592973	CTDSPL
MAGED1	0.014592973	MTDH
BRD7	0.014592973	QRSL1
GLI2	0.014592973	GPA33
BRMS1	0.014592973	EP300
RAF1	0.014592973	EPAS1
POU2AF1	0.014592973	MSLN
MAFG	0.014542126	EPHA1
PSMB4	0.014542126	EPHA3
GSTP1	0.014542126	EPHA7
PRDX5	0.014542126	EPHA8
IMMT	0.014542126	FBXW7
ISY1	0.014542126	EPHB2
KDM1A	0.014542126	EPHB4
UXT	0.014542126	EPHX1
CREB5	0.01449128	GLYAT
KIT	0.01449128	EPS8
EHMT1	0.01449128	MTG1
GEMIN4	0.01449128	SPRY2
EGLN2	0.01449128	NR2F6
CCND2	0.01449128	RPL29
ALDOA	0.014440433	ERBB2
GJB1	0.014440433	ERBB3
BIRC6	0.014440433	ERBB4
SNRPD1	0.014440433	ERCC1
ITGA4	0.014440433	EREG
CTNND1	0.014440433	EYA4
ZNF408	0.014389587	RPL37
AATF	0.014389587	BRIP1
PHC2	0.014389587	IRX5
REV1	0.014389587	PHF20
ZNF512B	0.014389587	REG4
NDUFA1	0.014389587	RPLP1
PEX19	0.014389587	RGS19
ADAM17	0.01433874	TMEM69
COMMD4	0.01433874	NANOG
MBD3	0.014287893	ESR1
COX17	0.014287893	RPS6KA1
NF1	0.014287893	ESR2
CLU	0.014287893	RPS6KA2
POLR2I	0.014287893	ESRRA

KIAA1377	0.014287893	RPS6KA3
LSM7	0.014287893	CHDH
MAP2K1	0.014287893	RPS6KB1
SOS1	0.014287893	ESRRB
CASP1	0.014287893	APC2
ZNHIT1	0.014237047	LAPTM4B
TCEA2	0.014237047	PAK4
RPL38	0.014237047	MIR1290
CCL5	0.014237047	MALAT1
KRT15	0.014237047	ETS1
RPL6	0.014237047	ETS2
HELZ	0.0141862	ETV4
IKZF3	0.0141862	MECOM
SSB	0.0141862	DHDDS
HIPK3	0.0141862	CCAT1
ZC3H14	0.0141862	RPS18
APOE	0.0141862	RPS19
CALM3	0.0141862	RPS20
RPS16	0.0141862	LRRC59
IQGAP1	0.0141862	RPS27A
ZMIZ1	0.0141862	RRAD
NOTCH2	0.0141862	RRM1
FOSL2	0.0141862	EZH2
SS18L1	0.0141862	WLS
EMILIN1	0.014135354	F2R
RPS6KA5	0.014135354	F2RL1
BMI1	0.014135354	F3
FUS	0.014135354	IL17F
SECISBP2L	0.014135354	F7
UQCC3	0.014135354	RXRA
IGFBP1	0.014135354	SLC25A37
FANCD2	0.014135354	RXRG
TRIM54	0.014135354	PLAC8
NONO	0.014135354	FABP6
SLC9A3R2	0.014084507	ZNF331
TMSB10	0.014084507	B3GNT8
CIC	0.014084507	CITED2
ALAS1	0.014084507	S100A4
FAM208B	0.014084507	ACSL4
LSM2	0.014084507	TUBA1B
IRS1	0.014084507	PCBD2

MIEN1	0.014084507	PTK2B
PSMA1	0.014084507	IRF9
CDC37	0.014084507	DACT1
PNN	0.01403366	ZBTB7A
IRF9	0.01403366	S100P
ACTN2	0.01403366	FAP
UQCRB	0.01403366	FZR1
GATA6	0.01403366	FBLN1
LGALS2	0.01403366	SAA1
NDUFS6	0.01403366	FASN
ZBTB17	0.013982814	FBN2
SP4	0.013982814	FBP1
PAX3	0.013982814	SALL1
CDC6	0.013982814	DLC1
NOTCH4	0.013982814	MAPK12
TNFAIP8L2	0.013982814	NDRG1
ARID3A	0.013982814	SAT1
OGT	0.013982814	RACK1
MED12	0.013982814	CPQ
GTF2A1	0.013982814	FCGR3A
MRPL21	0.013931967	IFITM3
COL1A1	0.013931967	SERPINB3
PCYT2	0.013931967	YAP1
H2AFX	0.013931967	SCD
SOCS1	0.013931967	SPON2
POLR2C	0.013931967	FDXR
GABBR1	0.013931967	FECH
CHCHD5	0.013931967	SCN5A
TUSC2	0.013931967	ASCC2
KARS	0.013931967	FGF1
FOXM1	0.013931967	FGF2
IQGAP2	0.013931967	FGF3
MYL6	0.013881121	ANTXR1
MRPL11	0.013881121	CCL2
CYR61	0.013881121	FGF7
NDUFA2	0.013881121	FGF9
POLR2B	0.013881121	TRPV6
TWIST1	0.013881121	CCL5
GPATCH4	0.013881121	CCL7
TGFB1	0.013881121	FGFR3
JAK1	0.013881121	MIR1288

KLF4	0.013881121	FGFR2
NDUFS8	0.013881121	FGFR4
RPS18	0.013881121	PWAR1
ARPC4	0.013830274	MAD2L2
PSMC3	0.013830274	AMOTL2
CHD6	0.013830274	CCL21
TNFRSF1B	0.013830274	FHIT
OLA1	0.013830274	FHL1
HMG20B	0.013830274	FHL2
RPL27A	0.013830274	SNX9
NDUFB7	0.013830274	SDC1
FBXL14	0.013830274	SDC2
ELAC1	0.013779427	EIF3M
SETD2	0.013779427	SDC4
TOPBP1	0.013779427	HOXB13
BCAR1	0.013779427	CXCL12
PTGS2	0.013779427	PHF21B
NDC80	0.013779427	CAP1
GMCL1	0.013779427	RBM38
MMP14	0.013728581	SEA
MAPK7	0.013728581	SYNCRIP
GEMIN7	0.013728581	FOXM1
SCAF11	0.013728581	SELE
RPSA	0.013728581	TBPL2
VAV1	0.013728581	FOXO1
TAOK1	0.013728581	FOXO3
PLAUR	0.013728581	SEMA3F
CCND3	0.013728581	FLI1
SLMAP	0.013728581	SELENOP
IRF2	0.013677734	MACC1
MYO5C	0.013677734	FLT1
SLC25A6	0.013677734	FLT3LG
LMO4	0.013677734	FLT4
SART1	0.013677734	SFPQ
HES1	0.013677734	ISYNA1
TPM2	0.013677734	SFRP1
JAK3	0.013677734	HSD17B7
CDC73	0.013626888	CIB1
TROVE2	0.013626888	ASRGL1
SYNJ1	0.013626888	DDX17
CLIC1	0.013626888	SRSF3

MRPS15	0.013626888	KAT5
NAGK	0.013626888	FN1
FBXW11	0.013626888	SRSF6
PPP2R2B	0.013626888	TRA2B
LZTR1	0.013626888	TCHP
YAF2	0.013626888	SSSCA1
SRSF2	0.013626888	PHLDA1
CDKN1B	0.013576041	UBD
TIMM13	0.013576041	GLRX3
NEDD4	0.013576041	PUF60
ZBTB11	0.013576041	SGCG
C19orf70	0.013576041	FOS
MRPL24	0.013576041	FOSB
COL2A1	0.013576041	FPGS
TFDP2	0.013525194	FPR2
RPA1	0.013525194	DTL
BCOR	0.013525194	FERMT1
ORC2	0.013525194	VASH1
TALDO1	0.013525194	PRSS55
SAP18	0.013525194	OLFM4
DDX56	0.013525194	CXCL13
IPO13	0.013525194	SHH
CUL4B	0.013525194	SHMT1
USP20	0.013525194	NTNG1
CSNK1D	0.013525194	MUS81
RPS21	0.013525194	SLU7
SNRPC	0.013525194	ICK
CASP8AP2	0.013525194	MAGI3
NDUFA11	0.013525194	SI
RPS15A	0.013525194	SIVA1
TYROBP	0.013474348	SIAH1
POM121	0.013474348	NLRP1
F3	0.013474348	VTA1
MRPS5	0.013474348	HBP1
TJP1	0.013474348	ST6GAL1
BRD3	0.013474348	SORBS1
CDC20	0.013474348	CHD7
HNRNPA2B1	0.013474348	SYBU
SERF2	0.013423501	PMEL
WARS	0.013423501	FXN
LAMTOR4	0.013423501	SIRT6

ZNF57	0.013423501	SIM2
RIPK3	0.013423501	MLXIP
NOS3	0.013423501	ZHX2
MRPS21	0.013423501	SKP2
ENO2	0.013423501	AHSA1
PCK2	0.013423501	SLCO1B1
TRBV28	0.013423501	SLCO6A1
TNFRSF14	0.013423501	IL23A
ZNF384	0.013423501	PRPF40A
KLHL20	0.013423501	SLC2A1
SALL1	0.013423501	SLC2A2
PTGDS	0.013372655	SLC2A3
SETD5	0.013372655	PDLIM5
RPL35A	0.013372655	LIMS3
NUP153	0.013372655	CARD8
BAZ2A	0.013372655	SLC2A4
UBE2H	0.013372655	GDE1
SLC31A1	0.013372655	CYFIP2
NR2F6	0.013372655	SLC4A2
MAGI1	0.013321808	FAN1
SCMH1	0.013321808	LIMS2
GOT2	0.013321808	SLC5A5
PAK2	0.013321808	KLRK1
CCDC12	0.013321808	MED15
HAP1	0.013321808	MMRN1
RPL32	0.013321808	PIAS4
ALDH6A1	0.013270962	SLC6A8
CAMK2A	0.013270962	SLC7A1
CAPNS1	0.013270962	KDM4D
MAOB	0.013270962	LSR
NDUFB9	0.013270962	PPARGC1B
TSHZ3	0.013270962	IGF2BP1
THBS1	0.013220115	SIRT2
NR1I3	0.013220115	MLH3
TTR	0.013220115	CDK5RAP3
MXD1	0.013220115	CCDC88A
MON2	0.013220115	SLC10A2
MYOG	0.013220115	NOX1
LRP1	0.013220115	SIGLEC7
MAP7D1	0.013220115	FRA16D
STARD13	0.013220115	DKK1

PRKCQ	0.013169268	KHDRBS1
SOX11	0.013169268	MIR17HG
DCXR	0.013169268	TENM3
MGA	0.013169268	CELF2
MEN1	0.013169268	LBX1
NDUFS4	0.013169268	SND1
UBXN4	0.013169268	CTCF
UQCR11	0.013169268	MTOR
MAT1A	0.013169268	DCTN6
AXL	0.013169268	TNFSF12-TNFSF13
MAFF	0.013169268	FER1L4
YAP1	0.013169268	SLC22A3
RANBP1	0.013169268	GNB5
DDX6	0.013118422	TET1
ATRX	0.013118422	EBP
HINT2	0.013118422	ZKSCAN3
ASH2L	0.013118422	PPIL1
APBB1	0.013118422	PPP1R2C
C8A	0.013118422	NR5A2
VCP	0.013118422	SLPI
GTF3C1	0.013118422	TPX2
BATF	0.013118422	FTH1
FIS1	0.013067575	SNAI2
FTH1	0.013067575	CHFR
AURKB	0.013067575	SMARCA1
BAX	0.013067575	NT5C2
RFC4	0.013067575	HLTF
MAML1	0.013016729	CCAR1
NDUFAB1	0.013016729	ULBP1
NADK2	0.013016729	DHX32
PGM3	0.013016729	SMPD1
ZNF174	0.013016729	SMPD2
COMMD1	0.013016729	SNAI1
HNRNPUL1	0.013016729	HSR
HP	0.012965882	GAST
CALR	0.012965882	FUS
NR1H4	0.012965882	POLD3
PRMT5	0.012965882	FUT1
RPL5	0.012965882	CACYBP
MAP4K4	0.012965882	FUT4
STX4	0.012965882	FUT5

NEDD8	0.012965882	FUT6
PRRC2A	0.012915035	FUT7
NR1I2	0.012915035	MAP3K21
GSTK1	0.012915035	OGA
EIF3K	0.012915035	BBC3
PRKCG	0.012915035	PLK4
PLAC9	0.012915035	PALLD
EFEMP2	0.012915035	CSGALNACT1
ALDH2	0.012915035	DKK4
CCDC117	0.012915035	SNX1
ATAD2B	0.012915035	DKK3
SLC52A2	0.012864189	XRCC6
SHANK2	0.012864189	KDM1A
RPL31	0.012864189	NLK
ING1	0.012864189	SOAT1
DDX39B	0.012864189	KDM4B
PRKAA2	0.012864189	GABPA
ADAM10	0.012864189	SOD2
HDAC7	0.012864189	GABRA1
ARL6IP1	0.012813342	CD276
ZC3H13	0.012813342	WDTC1
CYP3A7	0.012813342	CHL1
ACTG1	0.012813342	SOX2
NDUFV2	0.012813342	SOX9
GADD45B	0.012813342	PALD1
SEC24C	0.012813342	SMG1
DEDD	0.012813342	SOX12
ACOX2	0.012813342	SP1
RPS15	0.012813342	TUSC7
PLEKHO1	0.012813342	SP4
BUB1B	0.012762496	VPS11
VTA1	0.012762496	HCAR2
KMT2E	0.012762496	PAG1
CDK8	0.012762496	CLUAP1
IGHM	0.012762496	SRSF10
SUMO4	0.012762496	SPARC
MFSD10	0.012762496	TCERG1L
HOXA10	0.012762496	GLS2
CCR5	0.012762496	WWOX
SRRM2	0.012762496	SPINK1
EHHADH	0.012762496	SPINT1

ITGAV	0.012762496	GAPDH
ORC1	0.012762496	SPN
TET3	0.012711649	ACRBP
SOX3	0.012711649	SPP1
CEACAM1	0.012711649	KDM4C
NDUFS5	0.012711649	TMEM8B
NDUFV1	0.012711649	USE1
PURA	0.012711649	CYSLTR1
CCDC167	0.012711649	43352
FEN1	0.012711649	SPTAN1
LAMTOR2	0.012711649	IL17C
ARID1A	0.012711649	SDCCAG8
CDT1	0.012711649	SPTBN1
FBXW7	0.012711649	ENTR1
ASH1L	0.012711649	SRC
ZNF318	0.012711649	WASF3
PAXIP1	0.012660802	NOXA1
NR3C2	0.012660802	RSF1
TIMM8B	0.012660802	HDAC8
FBP1	0.012660802	PRSS22
SNRPE	0.012660802	MAP3K20
TSTD2	0.012660802	PBK
RBBP5	0.012660802	GATA4
ZRANB1	0.012660802	GATA6
FTL	0.012660802	MAK16
NAP1L1	0.012609956	NEU3
CASP2	0.012609956	COLCA1
MAP3K1	0.012609956	GC
WBP11	0.012609956	GCG
AKAP13	0.012609956	TRIM21
PITX2	0.012609956	DEFB103B
CDC45	0.012559109	KLRC4-KLRK1
NKTR	0.012559109	SST
VASP	0.012559109	SMOC2
NR6A1	0.012559109	KDM6B
NDN	0.012559109	SSTR2
RFXANK	0.012559109	CCL27
HSD17B4	0.012559109	SSX2
PIP5K1C	0.012559109	HPSE
CYC1	0.012508263	RUVBL2
DAO	0.012508263	HPP1

MOB2	0.012508263	TMED7-TICAM2
SGK1	0.012508263	LILRB1
ELF4	0.012508263	GDNF
REV3L	0.012508263	ST13
LAPTM5	0.012508263	ST14
INPP5D	0.012508263	PDCD4
FANCG	0.012508263	STAT1
TUFM	0.012508263	STAT2
EPC2	0.012508263	STAT3
KATNA1	0.012457416	TBC1D9
RPL23A	0.012457416	STAT5A
ABR	0.012457416	STAT5B
MRPL36	0.012457416	STAT6
POLA2	0.012457416	STC1
DHX38	0.012457416	SULT1E1
CERS2	0.012457416	SMR3B
UBE2U	0.012457416	NOD2
RPS23	0.012457416	GH1
ZFHX3	0.012457416	GHR
KLHL12	0.012457416	STK3
WAS	0.012457416	GHSR
LTN1	0.012457416	AURKA
VTN	0.012457416	GJA1
TYMS	0.012457416	STK11
IL2RG	0.012457416	PPARGC1A
UQCR10	0.012457416	RFTN1
EIF3A	0.012457416	GJB1
RPLP0	0.012457416	RCOR1
TBK1	0.012457416	KISS1R
CANX	0.012457416	UTS2
PRKAA1	0.012406569	PDLIM3
WIPF1	0.012406569	GLB1
INTS2	0.012406569	SULT1A1
CDK3	0.012406569	RBMS3
RBM38	0.012406569	GCLC
PDGFRB	0.012406569	REEP6
SOWAHB	0.012406569	GCLM
RPL26	0.012406569	JMJD6
BCL7C	0.012406569	HPGDS
STK16	0.012406569	SUPT5H
MCTS1	0.012406569	GLI1

HAAO	0.012406569	SPZ1
ATP5E	0.012355723	GLI2
DPM3	0.012355723	RALBP1
ECM1	0.012355723	GLI3
THRAP3	0.012355723	GLO1
HTATSF1	0.012355723	PRDX3
PIK3R5	0.012355723	BHLHE22
XPC	0.012355723	GLS
RALGAPB	0.012355723	FAM126A
DVL3	0.012355723	SIK2
MED13L	0.012355723	ADAMTS15
ZFYVE9	0.012355723	ARC
PLK3	0.012355723	CBX1
CDC23	0.012355723	PHLPP1
MED21	0.012355723	GMDS
GUK1	0.012355723	RABGEF1
ASF1B	0.012355723	MICA
FAF1	0.012355723	GNAI2
HSPE1	0.012355723	ADAM17
ATXN1L	0.012355723	SGSM3
GORASP2	0.012304876	GNAS
NCK2	0.012304876	CKAP4
ZNF524	0.012304876	TAGLN
PIGR	0.012304876	FERMT2
ZNF148	0.012304876	COPS6
SCNM1	0.012304876	MAP3K7
ABAT	0.012304876	RAB32
RPS17	0.012304876	TAP1
A1CF	0.012304876	SDS
SKI	0.012304876	MIR375
NECAP2	0.012304876	TAZ
NDEL1	0.012304876	GOT2
SFPQ	0.012304876	FBXW11
RPL19	0.012304876	KIF2C
BAD	0.012304876	TBX5
LIN54	0.012304876	IL24
RERE	0.012304876	GPI
HEATR5B	0.01225403	MIR424
NCBP1	0.01225403	ICOSLG
CUL4A	0.01225403	TCF4
CHEK2	0.01225403	HNF1A

FMR1	0.01225403	CXCR3
SERPINE1	0.01225403	SATB2
CDK6	0.01225403	TCF7
CTDSPL2	0.01225403	UTS2R
HOXA1	0.01225403	TCF7L2
FGFR1	0.01225403	SSX2B
ARPC5	0.01225403	ZEB1
PRMT2	0.012203183	DSTN
RPL37	0.012203183	RIPK3
HLA-A	0.012203183	RHBDF1
RP11-274B21.2	0.012203183	USP22
SEC14L2	0.012203183	PIM2
ARID1B	0.012203183	SASH1
FBXO34	0.012203183	CBLL2
BRPF3	0.012203183	TRBV20OR9-2
MYBBP1A	0.012203183	GRK5
ZMYM2	0.012203183	PCDHA@
UBE2D4	0.012203183	GPX1
PTGES3	0.012203183	GPX2
FAM50A	0.012203183	GPX3
ESRRA	0.012203183	C19orf48
SELENBP1	0.012152336	SOX17
CUL2	0.012152336	PSD3
MAGEA6	0.012152336	KLK14
VPS52	0.012152336	PPP1R13B
FBXL7	0.012152336	RAPGEF1
NEMF	0.012152336	HBD
NDUFB11	0.012152336	MUC5B
C16orf45	0.012152336	B3GNT7
PSMB6	0.012152336	TDG
SURF2	0.01210149	TDGF1
NHP2	0.01210149	TDGF1P3
WWC2	0.01210149	GRIN2A
SSSCA1	0.01210149	PRDX2
RPS25	0.01210149	NR3C1
KIAA1109	0.01210149	TEK
ECI1	0.01210149	PRDM5
IGF1R	0.01210149	STK31
TRBC2	0.01210149	TEKT5
BTF3	0.01210149	TERF2
HIPK1	0.01210149	CXCL1

ERCC2	0.01210149	TERT
PDIA3	0.01210149	GRP
RET	0.01210149	TFAP2A
SF3A1	0.01210149	GRPR
NOP10	0.01210149	DICER1
MYLK	0.01210149	NR2F2
USP36	0.01210149	SIRT3
GPD2	0.01210149	SIRT1
C20orf24	0.01210149	GSK3B
MYL9	0.01210149	GSN
CHAF1A	0.012050643	TFF1
SNTA1	0.012050643	TFF2
OTUD4	0.012050643	GSS
ABCG5	0.012050643	TFF3
PRAF2	0.012050643	TFPI
MID2	0.012050643	TFRC
INO80B	0.012050643	TGFA
RAB23	0.012050643	GSTM1
TADA2B	0.011999797	TGFB1
DDA1	0.011999797	MIR938
SIRT6	0.011999797	RIOX2
U91319.1	0.011999797	GSTM2
MET	0.011999797	TGFB2
TAF6	0.011999797	MMP25
PRR3	0.011999797	TGFB3
PPIA	0.011999797	CDC37
ADRM1	0.011999797	TGFBI
NME2	0.011999797	GSTP1
ANKRD26	0.011999797	TGFBR1
POLR2G	0.011999797	LIMD2
MAP3K14	0.011999797	TGFBR2
SYNJ2BP	0.011999797	GSTT1
RAE1	0.011999797	TGFBR3
BECN1	0.01194895	ZMAT3
PTAR1	0.01194895	MIR885
SUV39H2	0.01194895	MSH6
LPP	0.01194895	TGM2
EED	0.01194895	COL18A1
PXN	0.01194895	CCDC83
TUBG1	0.01194895	BVES
TRIM39	0.01194895	CORO1A

TRMT112	0.01194895	HHIP
TMEM159	0.01194895	THBS1
GTF3C4	0.011898103	THBS2
MAF1	0.011898103	SH3PXD2B
POMP	0.011898103	PTP4A3
USP11	0.011898103	GTF2H3
C8orf76	0.011898103	SF3B1
NUP98	0.011898103	THY1
ZBTB1	0.011898103	FSTL1
HEPH	0.011898103	TIA1
C19orf24	0.011898103	TIAL1
PSMA2	0.011847257	CCAT2
GADD45GIP1	0.011847257	SERPINA13P
ITSN2	0.011847257	GUCA2A
ZNF593	0.011847257	TIMP1
CBX1	0.011847257	GUCA2B
BRD2	0.011847257	TIMP2
RBFOX2	0.011847257	TIMP3
ZNF184	0.011847257	GUCY2C
POLR2E	0.011847257	NKX2-1
MRPL20	0.011847257	ZNF277
ALDOB	0.011847257	CBX5
PEX5	0.011847257	BRD4
MAP3K5	0.01179641	TLR2
SHCBP1	0.01179641	PES1
TADA2A	0.01179641	TLR3
DENND4C	0.01179641	TLR4
C1QBP	0.01179641	TLR5
RPS6	0.01179641	WIF1
LAMTOR1	0.01179641	ANXA10
UBE2Z	0.01179641	CHEK2
DMGDH	0.01179641	TM7SF2
BRIP1	0.01179641	CBX7
ANXA2	0.01179641	H2AFX
APOC4-APOC2	0.01179641	TMSB4X
SRA1	0.01179641	AKAP10
RPS5	0.01179641	CLEC3B
POLR2K	0.01179641	TNF
CCNA1	0.01179641	TNS4
MAPRE1	0.01179641	SUZ12
SMIM12	0.01179641	HAS1

MASP1	0.011745564	TNFRSF1A
PHB2	0.011745564	TNFRSF1B
PPP1R16A	0.011745564	HAS3
RFC1	0.011745564	PRRT1
KMT2D	0.011745564	SRRM2
STUB1	0.011745564	HCFC1
CMTM5	0.011745564	TOP1
DXO	0.011745564	TOP2A
RPL18A	0.011745564	HCRT
EIF3D	0.011745564	TP53
FAS	0.011745564	HCRTR1
ME2	0.011745564	SEC14L2
CCNC	0.011745564	HDAC1
KIF23	0.011694717	TP73
HIST2H2BE	0.011694717	HDAC2
HNRNPC	0.011694717	TPBG
GABARAP	0.011694717	HDGF
NDUFB8	0.011694717	TPM2
C14orf169	0.011694717	HEXB
KLHL28	0.011694717	TPM3
ETV6	0.011694717	CFH
C9orf16	0.011694717	HFE
PRDX1	0.011694717	HGF
ZNF580	0.011694717	TPT1
CD69	0.011694717	CRISP2
SLC27A5	0.011694717	NR2C2
TSR3	0.011694717	LPAR3
DMAP1	0.01164387	HSP90B1
HINT1	0.01164387	TRAF1
ZDHHC17	0.01164387	HIC1
IRAK1	0.01164387	TRAF2
STX1A	0.01164387	HIF1A
SOX2	0.01164387	HIP1
BAZ1B	0.01164387	TRAF6
HSF4	0.01164387	NME1-NME2
UQCRC1	0.01164387	HINT1
KIAA2026	0.01164387	HK2
EIF4A3	0.01164387	CASP14
CTDP1	0.01164387	CCNDBP1
ATP5C1	0.01164387	SMUG1
RPL10	0.01164387	HLA-A

SNRNP70	0.01164387	HLA-B
RPL37A	0.01164387	HLA-C
ANAPC15	0.01164387	ORC6
OST4	0.01164387	TRP-AGG2-5
ASPSCR1	0.01164387	HLA-DRB1
RPL34	0.01164387	TRPC1
MON1B	0.011593024	HLA-E
SPG11	0.011593024	HLA-G
RP11-817O13.8	0.011593024	HMGB1
RNF4	0.011593024	MGLL
EIF5A	0.011593024	TSC1
MRPL12	0.011593024	HMGCR
KHK	0.011593024	BRCA3
TFPT	0.011593024	HMGCS2
EPHX1	0.011593024	HMGA1
KLF6	0.011593024	HMMR
PFN1	0.011593024	HMOX1
QSER1	0.011593024	NR4A1
ZFPM2	0.011593024	HNF4A
MRPL18	0.011593024	HNRNPA1
VGLL4	0.011593024	HNRNPC
DZIP1	0.011593024	HNRNPF
KIDINS220	0.011593024	ALX4
AP2M1	0.011542177	TMEFF2
PA2G4	0.011542177	TWIST1
MED9	0.011542177	HOXA@
AURKAIP1	0.011542177	TXN
CYP8B1	0.011542177	TXNRD1
TGFB1I1	0.011542177	TYK2
RNF144A	0.011542177	TYMS
CD63	0.011542177	HOXB@
BMPR1B	0.011542177	TYRP1
ABCB4	0.011542177	CSRNP1
MAPK8IP1	0.011542177	CLPTM1L
TBC1D9	0.011542177	UBA52
PHF2	0.011542177	HOXB7
EEF2	0.011542177	UBE2I
RPS11	0.011542177	HPGD
PDE9A	0.011542177	C17orf97
KIAA1549	0.011491331	LINC01194
ITGA3	0.011491331	HPRT1

RCOR3	0.011491331	UCN
TXNL4A	0.011491331	UCP1
FNTB	0.011491331	UCP2
PRKAR1A	0.011491331	SLC35A2
ENO1	0.011491331	UGCG
NR1H3	0.011491331	HRAS
CCR1	0.011491331	UGT1A
DOCK1	0.011491331	UGT2B17
FBXO5	0.011491331	PRMT1
SEC23A	0.011491331	COL14A1
MNAT1	0.011491331	UNG
FBF1	0.011491331	USP4
ID4	0.011491331	HES1
CWC15	0.011491331	NR1H2
DCP1A	0.011440484	UQCRFS1
CEP192	0.011440484	HSD11B2
EZR	0.011440484	HSD17B2
ESRP2	0.011440484	USF1
PIPOX	0.011440484	HSF1
IMPDH2	0.011440484	HSPA1A
RPS27	0.011440484	HSPA1B
DNMT3B	0.011440484	HSPA4
BIRC5	0.011440484	HSPA5
OPTN	0.011440484	UVRAG
HHEX	0.011440484	HSPA8
HAGH	0.011440484	HSPA9
DYNLRB1	0.011389637	HSPB1
KRT18	0.011389637	HSPB2
GCH1	0.011389637	VCAM1
MORF4L2	0.011389637	HSP90AA1
DNMT3A	0.011389637	HSP90AA2P
CD3E	0.011389637	VDR
CDC34	0.011389637	VEGFA
TNIK	0.011389637	VEGFB
PIK3C2A	0.011389637	VEGFC
TIMM50	0.011338791	EZR
HNRNPM	0.011338791	VIM
UBE3A	0.011338791	VIP
NOS1	0.011338791	VIPR1
COPS2	0.011338791	HTC2
PTDSS2	0.011338791	ENPP7

CCT7	0.011338791	B3GNTL1
IGHA2	0.011338791	WAS
CCDC102B	0.011338791	EIF4H
KIF2A	0.011338791	DIABLO
AQP1	0.011338791	TNC
PACS1	0.011338791	WNT1
PDCD6IP	0.011287944	WNT3
PLS1	0.011287944	POTEF
SRPK1	0.011287944	WNT5A
NUMA1	0.011287944	ICAM1
INCENP	0.011287944	WNT11
43715	0.011287944	WRN
AMDHD2	0.011287944	WT1
JTB	0.011287944	TMPRSS4
SERTAD3	0.011287944	XDH
CNPY2	0.011287944	LIMS4
INO80	0.011287944	YTHDC2
COX7A1	0.011237098	XPC
GPX1	0.011237098	USP46
NR1H2	0.011237098	IDH1
SON	0.011237098	XPO1
STK17B	0.011237098	XRCC1
BEST1	0.011237098	MUC13
CPN2	0.011237098	XRCC2
PEBP1	0.011237098	XRCC3
PRKD1	0.011237098	XRCC5
MARK2	0.011237098	CDCP1
FURIN	0.011237098	IFI27
USP9X	0.011237098	YES1
SCAF8	0.011237098	YY1
GIGYF2	0.011237098	LOC100128922
U2AF2	0.011237098	YWHAZ
B3GAT3	0.011237098	IFNA1
ID1	0.011186251	ZFP36
SERTAD1	0.011186251	IFNA13
PPIH	0.011186251	ZIC1
TBC1D4	0.011186251	IFNB1
CCT4	0.011186251	IFNG
RALY	0.011186251	IFNGR1
THAP7	0.011186251	CNBP
RPL12	0.011186251	IGF1

HAO1	0.011186251	BCL11B
MED15	0.011186251	TP53COR1
S100A6	0.011186251	IGF1R
ACSM2B	0.011186251	IGF2
SERPIND1	0.011186251	IGFBP2
DHX15	0.011186251	IGFBP3
APRT	0.011135404	IGFBP4
CIAO1	0.011135404	IGFBP5
BCR	0.011135404	IGFBP6
LRPPRC	0.011135404	IGFBP7
UPF2	0.011135404	CCN1
TST	0.011135404	DIXDC1
POU5F1P3	0.011135404	IHH
ITGB5	0.011135404	IL1A
TRADD	0.011135404	IL1B
BRF2	0.011135404	IL2
BCAP31	0.011135404	IL2RG
RP5-849H19.3	0.011135404	HOTAIR
VKORC1	0.011135404	IL4
AKAP9	0.011135404	IL6
CD82	0.011135404	CXCL8
SF3B5	0.011135404	CXCR1
PSMB7	0.011135404	CXCR2
SART3	0.011135404	IL10
PRLR	0.011135404	IL13
NR5A2	0.011135404	EME1
TCP1	0.011135404	IL15
CRMP1	0.011135404	PLB1
CAT	0.011135404	IL17A
GABARAPL1	0.011135404	IL18
TAB1	0.011084558	ZNF143
SHARPIN	0.011084558	IMPA1
PMVK	0.011084558	ZBTB17
COX4I1	0.011084558	IDO1
PDX1	0.011084558	ING2
DHDDS	0.011084558	CXCL10
ALAD	0.011084558	INPPL1
MORF4L1P1	0.011084558	KCMF1
CHD4	0.011084558	TCIM
CD247	0.011084558	IPP
MYEOV2	0.011084558	PNO1

PSMD8	0.011084558	SLCO1B3
RNF40	0.011084558	IRF1
NCAPG	0.011084558	IRF2
COL1A2	0.011033711	DEFB4B
ACAT2	0.011033711	IRF3
SERPINA1	0.011033711	IRF5
TPM3	0.011033711	C1GALT1
HMOX2	0.011033711	IRS1
PFKFB1	0.011033711	FJX1
MTHFD1	0.011033711	ZNF217
MAD1L1	0.011033711	PRPF6
TRAPPC8	0.011033711	MARCKSL1
DOK3	0.011033711	ITGA3
MRE11A	0.011033711	ITGA4
ROMO1	0.011033711	NME9
ANXA7	0.011033711	ITGA5
OAZ1	0.011033711	HM13
TERF1	0.011033711	ITGAM
PTPN13	0.011033711	PWAR4
MRPL55	0.011033711	ITGAX
MRPL17	0.010982865	PMEPA1
MECP2	0.010982865	MUC3
NDUFB2	0.010982865	ITGB3
FEZ1	0.010982865	ARNTL2
UBE2E1	0.010982865	ITGB4
ADRBK2	0.010982865	ITGB6
PFDN1	0.010982865	ITK
CD40	0.010982865	PRDM2
C11orf30	0.010982865	JAK2
RNF11	0.010982865	JAK3
EPRS	0.010982865	JARID2
HNRNPA1	0.010982865	JUN
MST1	0.010982865	JUNB
VCL	0.010982865	JUND
TAX1BP3	0.010982865	ANOS1
MAPKAPK2	0.010982865	CD82
MRPS34	0.010932018	KCNA3
ELL2	0.010932018	CHPT1
TTF2	0.010932018	SLC12A9
CUL3	0.010932018	PDRG1
PRSS23	0.010932018	KCND3

ACTA2	0.010932018	IL1R2
AFF1	0.010932018	MALL
NINL	0.010932018	CXCR4
GNAI2	0.010932018	KCNH2
C12orf10	0.010932018	FZD5
MAF	0.010932018	ACKR3
LMNB1	0.010932018	WNT3A
RRM2	0.010932018	AKR1B10
UBE2G2	0.010932018	USP7
SRRM1	0.010932018	PDXP
DTL	0.010881172	NAV2
SLIRP	0.010881172	IMMP1L
NAB2	0.010881172	NECTIN4
SDC3	0.010881172	PRAP1
WWP2	0.010881172	KDR
MLLT10	0.010881172	TWSG1
TIMM10	0.010881172	REEP5
CYB5A	0.010881172	PCBP4
PSME3	0.010881172	KISS1
PIK3R4	0.010881172	KIT
PPAN	0.010881172	DEK
COX14	0.010881172	KIF22
PSENEN	0.010881172	KPNA2
C19orf43	0.010881172	NELFE
G6PD	0.010881172	KRAS
PHF21A	0.010881172	PLA2G7
CLN3	0.010830325	TRIM15
TSC22D3	0.010830325	KRT8
SERPINC1	0.010830325	CYSLTR2
RPS19BP1	0.010830325	RAB25
TXNDC5	0.010830325	AIMP2
TWIST2	0.010830325	CD248
NEK1	0.010830325	PLXDC1
UBE2W	0.010830325	KRT19
STRN4	0.010830325	TFPI2
EIF4A2	0.010830325	ST7
B4GALNT1	0.010830325	43167
IGF2R	0.010830325	PAK5
EIF4G1	0.010830325	FAM84A
DLG3	0.010779478	L1CAM
NFIB	0.010779478	AHSA2P

HIVEP1	0.010779478	TMSB15B
HSPBP1	0.010779478	TTYH2
RENBP	0.010779478	DEFB104B
MAP3K2	0.010779478	RPSA
RNF5	0.010779478	NUP214
NR4A2	0.010779478	CERS6
DMWD	0.010779478	LBR
TUG1	0.010779478	ZMIZ1
SSR4	0.010779478	LCK
UROD	0.010779478	LCN2
MRPL54	0.010779478	LCP1
BAK1	0.010779478	DLL1
CRX	0.010779478	LDHA
LATS1	0.010779478	RMDN2
ADORA2A	0.010779478	ADAMTSL3
PSMD3	0.010779478	LDHB
MCM10	0.010779478	LDLR
FAM214A	0.010728632	LEP
SRPRB	0.010728632	LEPR
LUC7L2	0.010728632	LGALS1
RBMX	0.010728632	LGALS3
CHCHD1	0.010728632	LGALS3BP
CD79A	0.010728632	LGALS9
HLA-C	0.010728632	FOSL1
HIVEP2	0.010728632	CEMIP
F11	0.010728632	THAP11
RRP1B	0.010728632	ADAMTS12
PPP2CB	0.010728632	LIFR
ONECUT2	0.010728632	LIG4
CALCOCO1	0.010728632	SLC9A4
TAF11	0.010728632	LIMS1
ACSM2A	0.010677785	HMGA2
UBTF	0.010677785	LLGL1
PRPF4	0.010677785	LMNA
FRRS1	0.010677785	XRS
ZNF776	0.010677785	LNPEP
CAPN1	0.010677785	VANGL1
CSNK1E	0.010677785	LOX
RPL4	0.010677785	LOXL2
ZNF609	0.010677785	LPA
SMC2	0.010677785	CYP4F3

RBBP8	0.010677785	BCAM
IKZF4	0.010677785	LUM
PRIM1	0.010677785	QTRT1
C14orf1	0.010677785	TACSTD2
RPL21	0.010677785	TM4SF1
METTL7A	0.010677785	EPCAM
PLEKHB2	0.010677785	SLC14A2
UBL5	0.010677785	MARCKS
LCP2	0.010677785	MXD1
LMO3	0.010677785	SMAD2
KBTBD4	0.010626939	SMAD3
FKBP1A	0.010626939	SMAD4
USP49	0.010626939	SMAD7
CASP7	0.010626939	SMAD9
ARID4A	0.010626939	MIA
PTGES	0.010626939	
HIP1	0.010626939	
KIAA2018	0.010626939	
CDC25C	0.010626939	
CPB2	0.010626939	
TRERF1	0.010626939	
CD44	0.010626939	
RACGAP1	0.010626939	
MAD2L1	0.010626939	
CENPK	0.010626939	
CDK12	0.010626939	
NCOA4	0.010626939	
TBL3	0.010626939	
GTF2E1	0.010626939	
MDC1	0.010626939	
DNAJC7	0.010626939	
TNIP1	0.010626939	
RNF181	0.010626939	
PLAU	0.010626939	
RP11-337C18.9	0.010576092	
FGA	0.010576092	
UQCRFS1	0.010576092	
AP2B1	0.010576092	
SREK1	0.010576092	
CYTH2	0.010576092	
ADRB2	0.010576092	

HAND2	0.010576092	
XYLT1	0.010576092	
TXNRD1	0.010576092	
ZNF41	0.010525245	
GLUL	0.010525245	
ADORA2A-AS1	0.010525245	
NSMCE1	0.010525245	
ADH6	0.010525245	
SPARC	0.010525245	
VPRBP	0.010525245	
MAP4K5	0.010525245	
TCEA1P2	0.010525245	
DUSP1	0.010525245	
FAM96B	0.010525245	
S100A4	0.010525245	
IL3	0.010525245	
PITX1	0.010525245	
CCDC101	0.010525245	
UBE2N	0.010525245	
RPS24	0.010525245	
SRGN	0.010525245	
PDPK1	0.010474399	
UQCRH	0.010474399	
CBFA2T2	0.010474399	
EIF4G2	0.010474399	
CDC40	0.010474399	
ASAP2	0.010474399	
PIK3CG	0.010474399	
KRTAP5-9	0.010474399	
CUL5	0.010474399	
KAT6A	0.010474399	
MED10	0.010474399	
PC	0.010474399	
SNHG6	0.010474399	
ABCA2	0.010474399	
EFTUD2	0.010423552	
FILIP1L	0.010423552	
GADD45G	0.010423552	
SF3B4	0.010423552	
ATG12	0.010423552	
SEC61G	0.010423552	

HIGD2A	0.010423552	
NCAPH2	0.010423552	
PHF1	0.010423552	
SULT2A1	0.010423552	
SLC22A25	0.010423552	
SPINT2	0.010423552	
TUBB6	0.010423552	
GNA15	0.010423552	
MRPL13	0.010423552	
C11orf96	0.010423552	
UBE2S	0.010423552	
LSM1	0.010423552	
RINT1	0.010423552	
A4GALT	0.010423552	
TAB2	0.010423552	
AGXT	0.010423552	
WDR83OS	0.010423552	
AQP9	0.010423552	
HSP90B1	0.010423552	
NEK2	0.010423552	
MRPL27	0.010372706	
PSMB1	0.010372706	
RP11-384K6.6	0.010372706	
RASSF2	0.010372706	
EIF3H	0.010372706	
LIMK1	0.010372706	
ZNRD1	0.010372706	
RPL10A	0.010372706	
EEF1A2	0.010372706	
RPL36A	0.010372706	
EPC1	0.010372706	
COX5B	0.010372706	
PACSIN1	0.010372706	
LCAT	0.010372706	
FIBP	0.010372706	
SMARCD3	0.010372706	
CD74	0.010372706	
HNRNPH1	0.010372706	
TTC1	0.010372706	
EP400	0.010372706	
PIK3CD	0.010372706	

ZNF417	0.010372706	
MPP3	0.010372706	
KMT2A	0.010372706	
BNIP3	0.010372706	
SPRY2	0.010372706	
CNOT6	0.010372706	
SKIV2L2	0.010321859	
ZFP91	0.010321859	
DNAJB14	0.010321859	
BAZ2B	0.010321859	
TCF7L2	0.010321859	
FAN1	0.010321859	
MAPK12	0.010321859	
LAGE3	0.010321859	
SAC3D1	0.010321859	
TYK2	0.010321859	
DSP	0.010321859	
MRPL23	0.010321859	
H3F3A	0.010321859	
PDRG1	0.010321859	
RBM39	0.010321859	
PRKAR2A	0.010321859	
TUBB4A	0.010321859	
RFC3	0.010321859	
SRCAP	0.010321859	
VPS13C	0.010321859	
MASP2	0.010321859	
FUNDC2	0.010321859	
ORC6	0.010321859	
KIAA0100	0.010271012	
CEP295	0.010271012	
MYOF	0.010271012	
TXN2	0.010271012	
PSMA5	0.010271012	
TAF2	0.010271012	
PIK3R3	0.010271012	
RBKS	0.010271012	
GH1	0.010271012	
PLA2G4A	0.010271012	
ALDOC	0.010271012	
LENG1	0.010271012	

ADA	0.010271012	
UBE2L6	0.010271012	
BLZF1	0.010271012	
GNAQ	0.010220166	
XIAP	0.010220166	
NOSIP	0.010220166	
CD81	0.010220166	
ZAP70	0.010220166	
SUPT5H	0.010220166	
RPS28	0.010220166	
RAB11FIP1	0.010220166	
PSMA4	0.010220166	
MTOR	0.010220166	
FGFR3	0.010220166	
MXD3	0.010220166	
MRPS2	0.010220166	
COX6B1	0.010220166	
LRRC37A16P	0.010169319	
FAM161A	0.010169319	
PDZRN3	0.010169319	
MCL1	0.010169319	
ILF2	0.010169319	
KRTCAP2	0.010169319	
SUOX	0.010169319	
PPIL4	0.010169319	
MSX1	0.010169319	
CTR9	0.010169319	
VWA7	0.010169319	
BMP4	0.010169319	
MEGF10	0.010169319	
PIM1	0.010169319	
APOC2	0.010169319	
SPTBN1	0.010169319	
HIC2	0.010169319	
USP39	0.010169319	
NCF4	0.010169319	
RBM12	0.010169319	
PPP4R2	0.010169319	
PCM1	0.010169319	
APOC1	0.010118473	
TAF1D	0.010118473	

PDCD2	0.010118473	
PFDN2	0.010118473	
PPP1R14B	0.010118473	
TRIM37	0.010118473	
OXSR1	0.010118473	
MAML2	0.010118473	
NAV2	0.010118473	
NEO1	0.010118473	
GGA3	0.010118473	
PARK7	0.010118473	
FANCA	0.010118473	
NCF1	0.010118473	
C8B	0.010118473	
GMPPA	0.010118473	
NME3	0.010118473	
SNX2	0.010118473	
ACE	0.010118473	
MIB1	0.010118473	
TMEM208	0.010118473	
NDUFC2	0.010118473	
MAZ	0.010067626	
ITGB4	0.010067626	
TFE3	0.010067626	
KRT8	0.010067626	
PTPLAD2	0.010067626	
B2M	0.010067626	
TAF5L	0.010067626	
EMG1	0.010067626	
CISD3	0.010067626	
ITGB3	0.010067626	
TRAPPC10	0.010067626	
UBE2B	0.010067626	
SLC6A9	0.010067626	
F10	0.010067626	
NOL7	0.010067626	
CENPA	0.010067626	
HLA-DMA	0.010067626	
CD53	0.010067626	
IGF2	0.010067626	
RPS4X	0.010016779	
TNRC6A	0.010016779	

GON4L	0.010016779	
LSM4	0.010016779	
LRCH4	0.010016779	
DTNBP1	0.010016779	
PNRC2	0.010016779	
COX6C	0.010016779	
CD34	0.010016779	
AXIN1	0.010016779	
NDUFB1	0.010016779	
RPL13A	0.010016779	
UIMC1	0.010016779	
TRIP10	0.010016779	
HEY1	0.010016779	
LCOR	0.010016779	
MYBL1	0.010016779	
CCL15	0.010016779	
ITPA	0.010016779	
MGST1	0.010016779	
FAM192A	0.010016779	
BOP1	0.009965933	
NUDT21	0.009965933	
SLC12A6	0.009965933	
LIN37	0.009965933	
PAICS	0.009965933	
SSNA1	0.009965933	
SLC9A1	0.009965933	
FZD5	0.009965933	
EXOSC7	0.009965933	
TNFRSF10A	0.009965933	
XAB2	0.009965933	
CORO7	0.009965933	
ENY2	0.009965933	
MED13	0.009965933	
ZW10	0.009965933	
NUBP2	0.009965933	
MANF	0.009965933	
HMBS	0.009965933	
GNG2	0.009915086	
NR2E3	0.009915086	
PBX3	0.009915086	
ZNF765	0.009915086	

HEY2	0.009915086	
BYSL	0.009915086	
SNCA	0.009915086	
MBD2	0.009915086	
G3BP2	0.009915086	
NID1	0.009915086	
STRAP	0.009915086	
ITGAM	0.009915086	
MRPL51	0.009915086	
PFKP	0.009915086	
DLST	0.009915086	
EPHB2	0.009915086	
CD86	0.009915086	
SF3A2	0.009915086	
PKM	0.009915086	
GOLGB1	0.009915086	
TIMP2	0.009915086	
SSX2IP	0.009915086	
ST13	0.009915086	
SMAD6	0.009915086	
ACAD8	0.00986424	
STRN	0.00986424	
PTK2B	0.00986424	
TM6SF2	0.00986424	
GLRX3	0.00986424	
LYL1	0.00986424	
PNMA2	0.00986424	
TUBGCP4	0.00986424	
HOXB2	0.00986424	
CELF2	0.00986424	
CASP4	0.00986424	
S100A11	0.00986424	
CAND1	0.00986424	
PLAT	0.00986424	
ITGB3BP	0.00986424	
SNRNP200	0.00986424	
RAB13	0.00986424	
ZNF281	0.00986424	
RBP4	0.00986424	
OXTR	0.00986424	
GTF2E2	0.00986424	

PLOD3	0.009813393	
PGLS	0.009813393	
UPB1	0.009813393	
VPS72	0.009813393	
NGFR	0.009813393	
MAP1B	0.009813393	
CRKL	0.009813393	
ATOX1	0.009813393	
NGEF	0.009813393	
RPS12	0.009813393	
PET100	0.009813393	
NDUFS3	0.009813393	
F2R	0.009813393	
RP11-297L17.2	0.009813393	
TM7SF2	0.009813393	
PSMB2	0.009813393	
CENPH	0.009813393	
ABI3	0.009813393	
USP15	0.009813393	
ATG2B	0.009813393	
BLM	0.009813393	
IST1	0.009813393	
NT5C	0.009762546	
VDAC1	0.009762546	
CENPM	0.009762546	
SHFM1	0.009762546	
PKN2	0.009762546	
PPFIA1	0.009762546	
SPP1	0.009762546	
C11orf31	0.009762546	
STK4	0.009762546	
AGMO	0.009762546	
ZNF654	0.009762546	
ELK4	0.009762546	
CAMK2B	0.009762546	
SPTAN1	0.009762546	
YEATS4	0.009762546	
PPP1R35	0.009762546	
TRAPPC1	0.009762546	
NOS1AP	0.009762546	
ERCC1	0.0097117	

F13B	0.0097117	
DHX30	0.0097117	
MSC	0.0097117	
SLC2A3	0.0097117	
TNNT1	0.0097117	
PKNOX1	0.0097117	
ISL1	0.0097117	
MFSD5	0.0097117	
WRN	0.0097117	
KLF5	0.0097117	
HES4	0.0097117	
PICK1	0.0097117	
TTBK2	0.0097117	
ZBTB7A	0.0097117	
POLR2D	0.0097117	
NDUFB10	0.0097117	
CDCA5	0.009660853	
MEAF6	0.009660853	
CEP55	0.009660853	
PRDX3	0.009660853	
STMN1	0.009660853	
LMAN2	0.009660853	
NDRG1	0.009660853	
RMI2	0.009660853	
RPN1	0.009660853	
PAX8	0.009660853	
CARD9	0.009660853	
SOS2	0.009660853	
PLCB2	0.009660853	
CLTA	0.009660853	
FDX1L	0.009660853	
CXCL16	0.009660853	
BCL2L13	0.009660853	
ANAPC11	0.009660853	
DYNC1I1	0.009660853	
CDO1	0.009660853	
GNG11	0.009660853	
FBXO31	0.009660853	
LINC00152	0.009660853	
EBPL	0.009660853	
ITGA5	0.009660853	

GOT1	0.009660853	
C5	0.009660853	
YLPM1	0.009660853	
HDAC6	0.009660853	
GCC2	0.009660853	
ERCC3	0.009660853	
CDH6	0.009660853	
CIB1	0.009660853	
IQCJ-SCHIP1	0.009610007	
LARP4	0.009610007	
ACTN1	0.009610007	
BTAF1	0.009610007	
BIRC2	0.009610007	
RBCK1	0.009610007	
SF1	0.009610007	
MAP3K12	0.009610007	
EBNA1BP2	0.009610007	
MPDZ	0.009610007	
ORAI2	0.009610007	
C1QA	0.009610007	
DNAJC22	0.009610007	
ATP5O	0.009610007	
MRPL35	0.009610007	
RAVER1	0.009610007	
FHOD1	0.009610007	
NDUFA4	0.009610007	
TMEM132A	0.009610007	
ZNHIT2	0.009610007	
ARAP2	0.009610007	
PRR5L	0.009610007	
NASP	0.009610007	
DAG1	0.00955916	
ELN	0.00955916	
DAP3	0.00955916	
SCP2	0.00955916	
CPSF6	0.00955916	
RRP36	0.00955916	
RP11-6N17.4	0.00955916	
STIP1	0.00955916	
FAM89B	0.00955916	
PAWR	0.00955916	

ARHGAP21	0.00955916	
INO80D	0.00955916	
KIF4A	0.00955916	
DYNC1H1	0.00955916	
PPP1R14BP3	0.00955916	
MORC3	0.00955916	
UGT2B4	0.00955916	
C16orf13	0.00955916	
HLA-DRA	0.00955916	
GPAA1	0.00955916	
SERPINF1	0.00955916	
SYT1	0.00955916	
SUCLA2	0.00955916	
TEAD3	0.00955916	
RPS29	0.00955916	
STON1-GTF2A1L	0.00955916	
ALDH9A1	0.00955916	
B4GALT7	0.00955916	
MAP2K7	0.00955916	
ACAP2	0.00955916	
DGAT2	0.009508313	
CASP6	0.009508313	
ARNTL	0.009508313	
CD19	0.009508313	
SDF2L1	0.009508313	
PPP2R1B	0.009508313	
IRF8	0.009508313	
PPP1R12B	0.009508313	
CDH2	0.009508313	
ESPL1	0.009508313	
TMSB4X	0.009508313	
TMEM258	0.009508313	
MLST8	0.009508313	
NUF2	0.009508313	
CCDC124	0.009508313	
MOB4	0.009508313	
SLC6A1	0.009508313	
MPV17	0.009508313	
ERICH5	0.009508313	
MSTO1	0.009508313	
CHMP6	0.009508313	

CD8A	0.009457467	
SEC61B	0.009457467	
DGUOK	0.009457467	
ABCA1	0.009457467	
MT1E	0.009457467	
TGFBRAP1	0.009457467	
XDH	0.009457467	
SNRPB2	0.009457467	
DUT	0.009457467	
RPL17	0.009457467	
TAZ	0.009457467	
DGKH	0.009457467	
ERGIC3	0.009457467	
EEA1	0.009457467	
PCBD2	0.009457467	
APBA1	0.009457467	
PAH	0.009457467	
PRR13	0.009457467	
CD2	0.009457467	
PNP	0.009457467	
IL32	0.009457467	
PHF12	0.009457467	
MAL2	0.009457467	
MINPP1	0.00940662	
NID2	0.00940662	
RPL9	0.00940662	
NEK6	0.00940662	
RLIM	0.00940662	
MED4	0.00940662	
TGFB2	0.00940662	
ALX4	0.00940662	
CDC7	0.00940662	
LLGL2	0.00940662	
BTBD2	0.00940662	
CHMP4B	0.00940662	
NKD1	0.00940662	
TMEM169	0.00940662	
PRR14L	0.00940662	
CENPJ	0.00940662	
FRS2	0.00940662	
HOXD13	0.00940662	

MARVELD2	0.00940662	
RP11-95D17.1	0.00940662	
HOMER3	0.009355774	
RAB17	0.009355774	
RPL3	0.009355774	
DRG1	0.009355774	
NRM	0.009355774	
TBC1D14	0.009355774	
GAR1	0.009355774	
MYCBP2	0.009355774	
SHANK1	0.009355774	
TOE1	0.009355774	
MPG	0.009355774	
ALDH7A1	0.009355774	
TRAF4	0.009355774	
NOLC1	0.009355774	
LONP2	0.009355774	
RPS6KA2	0.009355774	
KLF10	0.009355774	
CYCS	0.009355774	
PRRC2B	0.009355774	
MRPL40	0.009355774	
FUOM	0.009355774	
MRPS28	0.009355774	
LPL	0.009304927	
RNF166	0.009304927	
CTTNBP2NL	0.009304927	
BHMT2	0.009304927	
MIR135A1	0.009304927	
SLC26A6	0.009304927	
ZNF250	0.009304927	
EIF3B	0.009304927	
HM13	0.009304927	
UTP6	0.009304927	
NCSTN	0.009304927	
GCN1L1	0.009304927	
PCSK6	0.009304927	
ERCC4	0.009304927	
GNAS	0.009304927	
ITPR3	0.009304927	
USE1	0.009304927	

LIPC	0.009304927	
USP8	0.009304927	
CKAP5	0.009304927	
RAP1A	0.009304927	
PCGF6	0.009304927	
ATP6V1C1	0.009304927	
IDH3G	0.009304927	
HIC1	0.009304927	
MT-ND5	0.009304927	
TAT	0.009304927	
APLP1	0.009304927	
ZCCHC8	0.009304927	
MAGI3	0.009304927	
ABCC2	0.009304927	
HEYL	0.009304927	
GAS2	0.009304927	
FARSB	0.009304927	
ARF1	0.009304927	
AMOTL2	0.00925408	
SYN1	0.00925408	
ZNF423	0.00925408	
TRAPPC2L	0.00925408	
WRAP73	0.00925408	
FADD	0.00925408	
HLTF	0.00925408	
TDG	0.00925408	
HELLS	0.00925408	
EFNB2	0.00925408	
MAP4K1	0.00925408	
TOX3	0.00925408	
HEATR1	0.00925408	
ACD	0.00925408	
PNRC1	0.00925408	
AC005336.4	0.00925408	
COTL1	0.00925408	
ZNF521	0.00925408	
HMGCS2	0.00925408	
SCD5	0.00925408	
RP11-673C5.1	0.00925408	
CLASP2	0.00925408	
PSMA6	0.00925408	

KAT6B	0.00925408	
NAB1	0.00925408	
ACADM	0.009203234	
ZNF639	0.009203234	
AOX1	0.009203234	
GAB1	0.009203234	
SCO2	0.009203234	
ATP5G2	0.009203234	
STX17	0.009203234	
SS18L2	0.009203234	
HSPG2	0.009203234	
RNMT	0.009203234	
MED29	0.009203234	
RP4-798P15.3	0.009203234	
MLXIPL	0.009203234	
GNL1	0.009203234	
U91324.1	0.009203234	
LEMD3	0.009203234	
DTYMK	0.009203234	
HOXA5	0.009203234	
IL16	0.009203234	
RASD1	0.009203234	
G6PC3	0.009203234	
MRPL14	0.009203234	
UCHL5	0.009203234	
PRPF6	0.009203234	
LCMT1	0.009203234	
RBP5	0.009203234	
PGP	0.009203234	
UBE2K	0.009203234	
SRSF11	0.009152387	
CPN1	0.009152387	
MACF1	0.009152387	
MICAL3	0.009152387	
SF3B2	0.009152387	
LDHB	0.009152387	
GPX3	0.009152387	
MUC1	0.009152387	
ATP5J	0.009152387	
SRSF4	0.009152387	
NUP133	0.009152387	

PAM16	0.009152387	
HSD17B10	0.009152387	
TIFA	0.009152387	
FXYD6	0.009152387	
AHCY	0.009152387	
ACIN1	0.009152387	
DPM2	0.009152387	
FLT1	0.009152387	
SCAMP3	0.009152387	
HPN	0.009152387	
BACE1	0.009152387	
PPRC1	0.009152387	
ATP5L	0.009152387	
GOLGA4	0.009152387	
STX10	0.009101541	
RBM8A	0.009101541	
KNTC1	0.009101541	
GLTSCR1L	0.009101541	
KRIT1	0.009101541	
LSM3	0.009101541	
MZT2B	0.009101541	
GRHPR	0.009101541	
RNASEH2C	0.009101541	
SLC25A13	0.009101541	
DNAJA3	0.009101541	
CTSK	0.009101541	
SGOL2	0.009101541	
MLLT4	0.009101541	
CDCA8	0.009101541	
CAMK2G	0.009101541	
RFX3	0.009101541	
STX5	0.009101541	
RIC8A	0.009101541	
DEPDC7	0.009101541	
RASAL2	0.009101541	
SUPT16H	0.009101541	
RP11-273B20.1	0.009101541	
IL6ST	0.009101541	
ADAMTS2	0.009101541	
SERPINA10	0.009101541	
ZUFSP	0.009101541	

SGOL1	0.009101541	
COL4A2	0.009101541	
EIF3M	0.009101541	
PTEN	0.009101541	
MED24	0.009101541	
COX8A	0.009101541	
CCDC88B	0.009101541	
ATP2A2	0.009101541	
GINS1	0.009101541	
GIPC1	0.009101541	
ZFP64	0.009101541	
TOB1	0.009101541	
CCL2	0.009101541	
PROC	0.009101541	
MTSS1	0.009101541	
PSMB9	0.009050694	
CAPN7	0.009050694	
RRAS	0.009050694	
ABHD5	0.009050694	
CDCA3	0.009050694	
SASH3	0.009050694	
ABHD16A	0.009050694	
USP28	0.009050694	
MRPS22	0.009050694	
BID	0.009050694	
CANT1	0.009050694	
CLASP1	0.009050694	
COA4	0.009050694	
NPDC1	0.009050694	
SUB1	0.009050694	
KIFC3	0.009050694	
GATSL3	0.009050694	
DNM2	0.009050694	
COL4A1	0.009050694	
CHD9	0.009050694	
DCTN1	0.009050694	
DHX8	0.009050694	
ISG15	0.008999847	
CSF2RB	0.008999847	
MCPH1	0.008999847	
RAI1	0.008999847	

IGKC	0.008999847	
MT-CYB	0.008999847	
FAM208A	0.008999847	
KRT19	0.008999847	
RALY-AS1	0.008999847	
CDKN3	0.008999847	
U2SURP	0.008999847	
SAR1B	0.008999847	
BCL2A1	0.008999847	
EDA	0.008999847	
COQ4	0.008999847	
RP11-35N6.1	0.008999847	
PLXNA1	0.008999847	
LEMD2	0.008999847	
HSPA9	0.008999847	
A1BG	0.008999847	
SNRNP35	0.008999847	
CRYBG3	0.008999847	
ANKRD28	0.008999847	
DPAGT1	0.008999847	
OTX2	0.008999847	
BSG	0.008999847	
PHLDA2	0.008999847	
TOMM40	0.008999847	
MED7	0.008999847	
LINC00261	0.008949001	
CACYBP	0.008949001	
SLC25A5	0.008949001	
ING5	0.008949001	
DGKA	0.008949001	
CTSA	0.008949001	
SIL1	0.008949001	
BLVRB	0.008949001	
TRPM7	0.008949001	
ALDH1A1	0.008949001	
ALDH1A2	0.008949001	
MYD88	0.008949001	
FRYL	0.008949001	
ABCB1	0.008949001	
APLP2	0.008949001	
NUP160	0.008949001	

KMT2C	0.008949001	
TACC3	0.008949001	
SP100	0.008949001	
HNF4G	0.008949001	
SAP30BP	0.008949001	
DYRK1A	0.008949001	
MRPL43	0.008949001	
IGFBP6	0.008949001	
C8orf59	0.008949001	
FGD5-AS1	0.008949001	
TFR2	0.008949001	
IPO9	0.008949001	
HOXB7	0.008898154	
YES1	0.008898154	
FBXL15	0.008898154	
SPAG9	0.008898154	
RPL39	0.008898154	
UCP2	0.008898154	
MSX2	0.008898154	
MAN1A2	0.008898154	
ITK	0.008898154	
SOD3	0.008898154	
MYH9	0.008898154	
RAD50	0.008898154	
ECHS1	0.008898154	
UTP14C	0.008898154	
NFIA	0.008898154	
DOK1	0.008898154	
PELO	0.008898154	
CENPL	0.008898154	
MVP	0.008898154	
PEX16	0.008898154	
IL1B	0.008898154	
STK24	0.008898154	
RNF25	0.008898154	
IRS2	0.008898154	
AP1G1	0.008847308	
ACTN4	0.008847308	
PUM2	0.008847308	
TTK	0.008847308	
SPIN1	0.008847308	

RPL15	0.008847308	
TMEM219	0.008847308	
PLCB3	0.008847308	
FAM195A	0.008847308	
MTHFD2	0.008847308	
TCF7	0.008847308	
DGKZ	0.008847308	
NARFL	0.008847308	
JUP	0.008847308	
WDR45	0.008847308	
UHRF1	0.008847308	
HNRNPL	0.008847308	
STK11	0.008847308	
SRP68	0.008847308	
ZBED6	0.008847308	
MRPL2	0.008847308	
RHOC	0.008847308	
NOL3	0.008847308	
ROCK2	0.008847308	
GFER	0.008847308	
BGN	0.008847308	
AFAP1	0.008847308	
ALKBH7	0.008847308	
NAIP	0.008796461	
CD4	0.008796461	
SLIT2	0.008796461	
GPSM3	0.008796461	
CASP9	0.008796461	
CDCP1	0.008796461	
ZBTB26	0.008796461	
DDOST	0.008796461	
POLR1C	0.008796461	
BCL9	0.008796461	
UGT1A1	0.008796461	
YIF1A	0.008796461	
PLCG2	0.008796461	
CDC27	0.008796461	
CCDC58	0.008796461	
KIF5B	0.008796461	
43718	0.008796461	
TBXAS1	0.008796461	

FANCI	0.008796461	
RCAN1	0.008796461	
RECQL5	0.008796461	
QKI	0.008796461	
RAC2	0.008796461	
ENG	0.008745614	
RPA2	0.008745614	
FLJ20021	0.008745614	
EXO1	0.008745614	
RGS10	0.008745614	
TAF5	0.008745614	
PTRF	0.008745614	
IFNG	0.008745614	
XRCC2	0.008745614	
EPOR	0.008745614	
IKBKAP	0.008745614	
MED6	0.008745614	
NPLOC4	0.008745614	
ZKSCAN8	0.008745614	
CD2AP	0.008745614	
ZMYND11	0.008745614	
SNAP25	0.008745614	
PEX14	0.008745614	
FUBP1	0.008745614	
F7	0.008745614	
CCDC88A	0.008745614	
MIER1	0.008745614	
GC	0.008745614	
JDP2	0.008745614	
MYCBP	0.008745614	
GNL3	0.008745614	
MT-CO1	0.008745614	
TMEM134	0.008745614	
ITIH3	0.008745614	
NENF	0.008745614	
MNDA	0.008694768	
ENTPD3-AS1	0.008694768	
RNH1	0.008694768	
AP2S1	0.008694768	
CTB-131K11.1	0.008694768	
RP11-468N14.3	0.008694768	

DPP4	0.008694768	
ASPM	0.008694768	
FES	0.008694768	
ROR2	0.008694768	
DHX36	0.008694768	
MED17	0.008694768	
EEF1B2	0.008694768	
ANXA6	0.008694768	
PAN3	0.008694768	
NIPBL	0.008694768	
SELK	0.008694768	
SNRPF	0.008694768	
USPL1	0.008694768	
PTGES2	0.008694768	
C11orf68	0.008694768	
EIF3I	0.008694768	
KDR	0.008694768	
SI	0.008694768	
DPP8	0.008694768	
ATP1A1	0.008694768	
CFH	0.008694768	
MLLT6	0.008694768	
CLSPN	0.008694768	
RPP38	0.008694768	
MIR4435-1HG	0.008694768	
RTN4	0.008643921	
DOCK2	0.008643921	
TRIM21	0.008643921	
PSMA7	0.008643921	
MELK	0.008643921	
OGG1	0.008643921	
STAG2	0.008643921	
ASPDH	0.008643921	
SLC25A20	0.008643921	
BBS4	0.008643921	
MSH6	0.008643921	
ZNF236	0.008643921	
TEF	0.008643921	
AGMAT	0.008643921	
INTS6	0.008643921	
USP19	0.008643921	

SORL1	0.008643921	
PGF	0.008643921	
BRD8	0.008593075	
ATP6V0B	0.008593075	
ALDH3A2	0.008593075	
HGD	0.008593075	
SUZ12	0.008593075	
ACSL5	0.008593075	
BRAF	0.008593075	
ASGR1	0.008593075	
43713	0.008593075	
LINC00870	0.008593075	
HPX	0.008593075	
PPP1CB	0.008593075	
CRYAB	0.008593075	
C2	0.008593075	
GJA1	0.008593075	
CLCF1	0.008593075	
SNRPG	0.008593075	
SIRT2	0.008593075	
ITCH	0.008593075	
CICP14	0.008593075	
UHMK1	0.008593075	
DCAF13	0.008593075	
PDLIM7	0.008593075	
PSMG3	0.008593075	
ARFGEF2	0.008593075	
ATP7A	0.008593075	
SNAPIN	0.008593075	
RAB3IL1	0.008593075	
CAMK2D	0.008593075	
TMEM53	0.008593075	
NFKBIL1	0.008593075	
HEXIM1	0.008593075	
TBC1D22B	0.008593075	
C8orf33	0.008593075	
NDUFB6	0.008593075	
TCEAL1	0.008593075	
NR0B1	0.008593075	
THOC6	0.008593075	
GSDMD	0.008593075	

MLYCD	0.008542228	
CD14	0.008542228	
CHD2	0.008542228	
TEAD4	0.008542228	
ARPC1B	0.008542228	
RBBP6	0.008542228	
SLC6A12	0.008542228	
FAM104A	0.008542228	
MBNL3	0.008542228	
AKAP11	0.008542228	
HOXB4	0.008542228	
SUPT3H	0.008542228	
SEMA4G	0.008542228	
M6PR	0.008542228	
NDUFB3	0.008542228	
CHMP5	0.008542228	
ENPP1	0.008542228	
USMG5	0.008542228	
C12orf57	0.008542228	
PRELID1	0.008542228	
RAPGEF4	0.008542228	
NKX2-1	0.008542228	
PSMD14	0.008542228	
DCUN1D1	0.008542228	
TPR	0.008542228	
RBM48	0.008542228	
EPO	0.008542228	
JMJD8	0.008542228	
MT-ATP6	0.008542228	
DCN	0.008491382	
MIR621	0.008491382	
ADAM28	0.008491382	
RELL1	0.008491382	
KLF11	0.008491382	
RBMS2	0.008491382	
ATPAF2	0.008491382	
TRAPPC3	0.008491382	
RECQL	0.008491382	
CHAF1B	0.008491382	
RAB31	0.008491382	
DNAJC17	0.008491382	

FBLN2	0.008491382	
CYP1A1	0.008491382	
POLD1	0.008491382	
KIF18A	0.008491382	
POU1F1	0.008491382	
PRC1	0.008491382	
KIF2C	0.008491382	
PPOX	0.008491382	
MRFAP1L1	0.008491382	
DNAJA1	0.008491382	
ITGA6	0.008491382	
TRAPPC6A	0.008491382	
KIF11	0.008491382	
SEMA4A	0.008491382	
NAV1	0.008440535	
NACA	0.008440535	
PLGLB2	0.008440535	
DMXL1	0.008440535	
MAP4K3	0.008440535	
FGR	0.008440535	
HCFC2	0.008440535	
KLHL24	0.008440535	
DIP2A	0.008440535	
FGG	0.008440535	
FGFR2	0.008440535	
PSMC1	0.008440535	
ADPRHL2	0.008440535	
MED26	0.008440535	
JMJD4	0.008440535	
CTNNA1	0.008440535	
ATP6V0E1	0.008440535	
PLA2G16	0.008440535	
ATP6V1E1	0.008440535	
FDX1	0.008440535	
ATP5A1	0.008440535	
ADCY3	0.008440535	
POLE4	0.008440535	
IL4R	0.008389688	
RP11-1151B14.2	0.008389688	
RHOBTB1	0.008389688	
EVA1B	0.008389688	

LAMA4	0.008389688	
KLHDC4	0.008389688	
ADRBK1	0.008389688	
HNRNPR	0.008389688	
HNF1A-AS1	0.008389688	
MRPL47	0.008389688	
COPA	0.008389688	
CFL2	0.008389688	
PRPF8	0.008389688	
CTH	0.008389688	
HIST1H3A	0.008389688	
DMPK	0.008389688	
PMP22	0.008389688	
EMD	0.008389688	
IPO4	0.008389688	
PRIM2	0.008389688	
ETV1	0.008389688	
ATP5I	0.008389688	
BBS2	0.008389688	
KLF2	0.008389688	
F12	0.008389688	
HECTD1	0.008389688	
EIF3G	0.008389688	
PIKFYVE	0.008389688	
STK3	0.008389688	
TMEM256	0.008338842	
AP1M1	0.008338842	
DEPDC1	0.008338842	
SDC1	0.008338842	
PPM1A	0.008338842	
ZBTB44	0.008338842	
E4F1	0.008338842	
COG5	0.008338842	
PHC1	0.008338842	
ELK3	0.008338842	
TRIM32	0.008338842	
CST3	0.008338842	
ZHX2	0.008338842	
TRIM25	0.008338842	
LRRC37A17P	0.008338842	
HLA-DPB1	0.008338842	

HUWE1	0.008338842	
ZCCHC11	0.008338842	
ACOT8	0.008338842	
RPL26L1	0.008338842	
ARG1	0.008338842	
ITPKB	0.008338842	
DMTF1	0.008338842	
ZNF185	0.008338842	
ZNF367	0.008338842	
TSHZ1	0.008338842	
COX7C	0.008338842	
CTSD	0.008338842	
UBE2A	0.008338842	
JAG1	0.008338842	
ACTR2	0.008338842	
MRPS16	0.008338842	
RP11-407B7.1	0.008338842	
GRB7	0.008338842	
CHTF18	0.008287995	
TSC1	0.008287995	
PLCD3	0.008287995	
SLC25A39	0.008287995	
DPY30	0.008287995	
DOK2	0.008287995	
RTFDC1	0.008287995	
ZNF205	0.008287995	
AHSG	0.008287995	
RHOJ	0.008287995	
SMARCD2	0.008287995	
TPRG1-AS1	0.008287995	
PLA2G12B	0.008287995	
CRIP2	0.008287995	
ITSN1	0.008287995	
ZYX	0.008287995	
NUP205	0.008287995	
CLPX	0.008287995	
RFX5	0.008287995	
C1QC	0.008287995	
PAFAH1B1	0.008287995	
KIF20A	0.008287995	
MIP	0.008287995	

PTMS	0.008287995	
BTG2	0.008287995	
HERC3	0.008287995	
BAP1	0.008287995	
RAD51AP1	0.008287995	
SMARCA1	0.008287995	
DTX2	0.008287995	
PPP2R5D	0.008287995	
ALK	0.008287995	
CNOT4	0.008287995	
PCSK4	0.008287995	
SETX	0.008287995	
DMD	0.008287995	
RNF41	0.008287995	
RAB3GAP2	0.008287995	
COL3A1	0.008237149	
ARPC3	0.008237149	
IREB2	0.008237149	
CLOCK	0.008237149	
JOSD2	0.008237149	
PHYHIP	0.008237149	
PRDM4	0.008237149	
GLYATL1	0.008237149	
FAM178A	0.008237149	
SYNE1	0.008237149	
KIAA1429	0.008237149	
PELP1	0.008237149	
MXD4	0.008237149	
C3P1	0.008237149	
RFXAP	0.008237149	
ARL2	0.008237149	
TLR4	0.008237149	
SRPK2	0.008237149	
RP13-650J16.1	0.008237149	
TAF15	0.008237149	
TEX11	0.008237149	
KBTBD7	0.008237149	
PMF1	0.008237149	
PARVG	0.008237149	
WDR46	0.008237149	
CPSF7	0.008237149	

TIMM9	0.008237149	
ARL6IP4	0.008237149	
SNAI2	0.008237149	
EIF4E	0.008186302	
NBN	0.008186302	
RMI1	0.008186302	
ATAD5	0.008186302	
TXLNA	0.008186302	
CUTA	0.008186302	
HIRA	0.008186302	
MEIS3	0.008186302	
CCP110	0.008186302	
ZFP36L2	0.008186302	
HTATIP2	0.008186302	
SMG9	0.008186302	
RAB1A	0.008186302	
ALYREF	0.008186302	
IFI16	0.008186302	
PRICKLE1	0.008186302	
PSMB5	0.008186302	
PER1	0.008186302	
OIP5	0.008186302	
LRRK1	0.008186302	
AXIN2	0.008186302	
HAT1	0.008186302	
RDH5	0.008186302	
ABI2	0.008186302	
SH3BGRL3	0.008186302	
CEP57L1	0.008186302	
SRSF3	0.008186302	
L3MBTL2	0.008186302	
ARHGAP30	0.008186302	
SLC25A3	0.008186302	
GOLT1A	0.008186302	
KLHDC1	0.008186302	
ASCL1	0.008186302	
TFAP2B	0.008186302	
FAM173A	0.008186302	
WIBG	0.008135455	
USO1	0.008135455	
VDAC3	0.008135455	

HJURP	0.008135455	
PPP1R7	0.008135455	
MLX	0.008135455	
SH3GL2	0.008135455	
PLAGL2	0.008135455	
AUP1	0.008135455	
UBE2L3	0.008135455	
MAVS	0.008135455	
ACADSB	0.008135455	
ITGA2B	0.008135455	
UBE2E3	0.008135455	
BDP1	0.008135455	
CCDC38	0.008135455	
KHSRP	0.008135455	
RDX	0.008135455	
AQP11	0.008135455	
PTPN1	0.008135455	
MRVI1	0.008135455	
USP1	0.008135455	
KIAA1161	0.008135455	
ARHGAP1	0.008135455	
BUB1	0.008135455	
MSRB1	0.008135455	
MRPL41	0.008135455	
NUP43	0.008135455	
PSEN2	0.008135455	
NDUFAF3	0.008135455	
RFWD3	0.008084609	
HPR	0.008084609	
ANLN	0.008084609	
ARID3C	0.008084609	
UPF3A	0.008084609	
ZNF212	0.008084609	
DVL1	0.008084609	
GATAD1	0.008084609	
PMS1	0.008084609	
CMBL	0.008084609	
KLKB1	0.008084609	
RAP2A	0.008084609	
DEF6	0.008084609	
IVL	0.008084609	

TLX1	0.008084609	
FHL1	0.008084609	
WDR62	0.008084609	
DDR1	0.008084609	
PPP3CA	0.008084609	
INAFM1	0.008084609	
ARHGEF1	0.008084609	
ANKRD10-IT1	0.008084609	
TRIP11	0.008084609	
ANGPTL3	0.008084609	
CD5	0.008084609	
GCDH	0.008084609	
RABEP1	0.008084609	
CYP27A1	0.008084609	
KCNQ1	0.008084609	
IL7R	0.008084609	
CAMSAP3	0.008084609	
HMGCL	0.008084609	
LDLRAP1	0.008084609	
HAVCR2	0.008084609	
NRGN	0.008084609	
SETD7	0.008084609	
OPA1	0.008084609	
TCF19	0.008084609	
DAAM2	0.008033762	
ITPR1	0.008033762	
RAB4B	0.008033762	
DAPK3	0.008033762	
FZD1	0.008033762	
CIRBP	0.008033762	
CNOT8	0.008033762	
COL8A1	0.008033762	
RP11-274B21.4	0.008033762	
LAMB1	0.008033762	
CCL16	0.008033762	
CRY1	0.008033762	
PLEKHM3	0.008033762	
GGH	0.008033762	
DECR1	0.008033762	
FH	0.008033762	
ZWINT	0.008033762	

RP11-266L9.8	0.008033762	
ZBTB43	0.008033762	
FAM63B	0.008033762	
CENPI	0.008033762	
FBN1	0.008033762	
MFSD3	0.008033762	
EFEMP1	0.008033762	
NAGS	0.008033762	
FERMT3	0.008033762	
MMP1	0.008033762	
NDUFB5	0.008033762	
MRPL9	0.008033762	
ELMSAN1	0.008033762	
PMS2	0.008033762	
SPINT1	0.008033762	
ADD1	0.007982916	
CENPF	0.007982916	
ATP5D	0.007982916	
PTP4A2	0.007982916	
SMLR1	0.007982916	
TBC1D5	0.007982916	
GYS1	0.007982916	
CYP2D6	0.007982916	
PSD	0.007982916	
COPS3	0.007982916	
NDRG2	0.007982916	
PES1	0.007982916	
DBH	0.007982916	
RFFL	0.007982916	
FSTL1	0.007982916	
SNAPC2	0.007982916	
MT-CO3	0.007982916	
NCDN	0.007982916	
POP5	0.007982916	
ERI3	0.007982916	
TERF2	0.007982916	
TRAIP	0.007982916	
ING4	0.007982916	
CFHR2	0.007982916	
CYP2E1	0.007982916	
PLIN3	0.007982916	

CORO1A	0.007982916	
GTF3C2	0.007982916	
GLRX	0.007982916	
PSMD2	0.007982916	
EXOSC1	0.007982916	
LRP2	0.007982916	
MGAT2	0.007982916	
CNBP	0.007982916	
TMED9	0.007932069	
C17orf59	0.007932069	
GNPTG	0.007932069	
ASNA1	0.007932069	
AASS	0.007932069	
NME1-NME2	0.007932069	
HAPLN3	0.007932069	
C8orf82	0.007932069	
MYO15B	0.007932069	
CSF1	0.007932069	
PKMYT1	0.007932069	
MEIS2	0.007932069	
LRFN4	0.007932069	
TNPO1	0.007932069	
ITGA9	0.007932069	
THOC2	0.007932069	
PARD6A	0.007932069	
PSMD1	0.007932069	
PDXP	0.007932069	
INPP5K	0.007932069	
ETNK2	0.007932069	
TUBA1B	0.007932069	
GPR183	0.007932069	
PPP1R18	0.007932069	
PTPN4	0.007932069	
UTP23	0.007932069	
MORF4L1	0.007932069	
SLAMF8	0.007932069	
SLC25A37	0.007932069	
SNX10	0.007932069	
FASTK	0.007932069	
TNKS2	0.007932069	
LGR4	0.007932069	

CMTM7	0.007932069	
KDM3B	0.007932069	
MRPL33	0.007932069	
RAD54B	0.007932069	
EIF3L	0.007881222	
ANKRD13D	0.007881222	
IL12RB1	0.007881222	
EGFL7	0.007881222	
RASSF5	0.007881222	
NOC4L	0.007881222	
NUDC	0.007881222	
LUC7L3	0.007881222	
HOXD4	0.007881222	
APOBEC3B	0.007881222	
RAB5A	0.007881222	
NECAB2	0.007881222	
PSMC4	0.007881222	
SLC8A1	0.007881222	
UBAP2	0.007881222	
DPM1	0.007881222	
TRMT61A	0.007881222	
LRP6	0.007881222	
ICAM1	0.007881222	
TPT1	0.007881222	
TIMP3	0.007881222	
DNAJB2	0.007881222	
DDX41	0.007881222	
PRKAR2B	0.007881222	
RP11-903H12.5	0.007881222	
RP11-977G19.5	0.007881222	
BBS1	0.007881222	
FMNL3	0.007881222	
SLC30A10	0.007881222	
SRSF9	0.007881222	
HBA2	0.007881222	
FOXN2	0.007881222	
SRSF7	0.007881222	
FIGF	0.007881222	
CTC-459F4.3	0.007881222	
MTHFD1L	0.007881222	
ETV3	0.007881222	

PYCRL	0.007881222	
DIDO1	0.007881222	
GFOD2	0.007881222	
CCT5	0.007830376	
GNAO1	0.007830376	
PAN2	0.007830376	
ZNF217	0.007830376	
VEGFC	0.007830376	
VPS4A	0.007830376	
LRRC14	0.007830376	
HDHD3	0.007830376	
CUEDC2	0.007830376	
CASK	0.007830376	
DCTN3	0.007830376	
TRAK1	0.007830376	
CYP17A1	0.007830376	
TLK1	0.007830376	
UBE2C	0.007830376	
UHRF1BP1	0.007830376	
QTRTD1	0.007830376	
PDE5A	0.007830376	
TARBP2	0.007830376	
CHRD	0.007830376	
CDC42EP3	0.007830376	
TNKS	0.007830376	
ZBTB3	0.007830376	
MKNK1	0.007830376	
ESPN	0.007830376	
PSMD11	0.007830376	
SPDYC	0.007830376	
RAC3	0.007830376	
DHRS4L1	0.007830376	
HOMEZ	0.007830376	
WDR77	0.007830376	
ACAD11	0.007830376	
CDK5RAP3	0.007830376	
NDUFA8	0.007779529	
GLYCTK	0.007779529	
NDUFAF2	0.007779529	
EPB41L3	0.007779529	
TRIB1	0.007779529	

MIA2	0.007779529	
UQCRC2	0.007779529	
ADNP	0.007779529	
GTSE1	0.007779529	
FGB	0.007779529	
UGT2B7	0.007779529	
PSMC2	0.007779529	
FGF2	0.007779529	
STX8	0.007779529	
ETFB	0.007779529	
ZNF322	0.007779529	
GXYLT1	0.007779529	
ACOX1	0.007779529	
IL18	0.007779529	
SELPLG	0.007779529	
FARP2	0.007779529	
EIF3F	0.007779529	
SRP19	0.007779529	
TGFA	0.007779529	
SLA	0.007779529	
ACP5	0.007779529	
BAAT	0.007779529	
EIF4E2	0.007779529	
SMNDC1	0.007779529	
GRWD1	0.007779529	
PTCH2	0.007779529	
ECT2	0.007779529	
GMNN	0.007779529	
SMYD2	0.007779529	
LINC01558	0.007779529	
ALDH3B1	0.007779529	
PRKX	0.007779529	
MNT	0.007779529	
TEX10	0.007779529	
SPCS1	0.007728683	
PMEPA1	0.007728683	
RP11-141M3.6	0.007728683	
NUSAP1	0.007728683	
KXD1	0.007728683	
EIF2B4	0.007728683	
IL18BP	0.007728683	

EAF2	0.007728683	
CREBZF	0.007728683	
PPP1R9B	0.007728683	
RORC	0.007728683	
LAMC2	0.007728683	
IL5	0.007728683	
POLR1A	0.007728683	
ARNT2	0.007728683	
POLR1B	0.007728683	
XRN2	0.007728683	
NSF	0.007728683	
TGM2	0.007728683	
C1orf94	0.007728683	
C18orf21	0.007728683	
MAPK4	0.007728683	
CCDC33	0.007728683	
OCLN	0.007728683	
DBNL	0.007728683	
LDHD	0.007728683	
ITIH4	0.007728683	
TBX3	0.007728683	
F9	0.007728683	
DAAM1	0.007728683	
ARHGAP31	0.007728683	
RP11-813N20.1	0.007728683	
EEF2K	0.007728683	
RP11-295G20.2	0.007677836	
NTRK3	0.007677836	
EXOC6B	0.007677836	
DGCR6L	0.007677836	
KIAA1524	0.007677836	
CD47	0.007677836	
ATP7B	0.007677836	
CERCAM	0.007677836	
VCPIP1	0.007677836	
HMMR	0.007677836	
BRE	0.007677836	
ANTXR1	0.007677836	
CNOT7	0.007677836	
IGFBP5	0.007677836	
LY86	0.007677836	

SETD8	0.007677836	
PDS5B	0.007677836	
SERPINA5	0.007677836	
SDC2	0.007677836	
TIMP1	0.007677836	
CSF1R	0.007677836	
XYLB	0.007677836	
CKLF	0.007677836	
ATP5G1	0.007677836	
NAMPT	0.007677836	
ZBTB9	0.007677836	
POLR1E	0.007677836	
SLC35A5	0.007677836	
MTF2	0.007677836	
MTDH	0.007677836	
HSD17B6	0.007677836	
EXOC5	0.007677836	
SLC3A2	0.007677836	
GATM	0.007677836	
ZNF516	0.007677836	
SNTB1	0.007677836	
ATPIF1	0.007626989	
STIL	0.007626989	
PDE1A	0.007626989	
43710	0.007626989	
ANAPC1	0.007626989	
MTTP	0.007626989	
PRR7	0.007626989	
CD48	0.007626989	
PDE4DIP	0.007626989	
TSC2	0.007626989	
FKBP4	0.007626989	
TPX2	0.007626989	
SERPINH1	0.007626989	
TBCC	0.007626989	
RPL39P3	0.007626989	
TSNAX	0.007626989	
TTC37	0.007626989	
TYRO3	0.007626989	
HLA-DRB1	0.007626989	
TNFAIP8	0.007626989	

GTF2A2	0.007626989	
NAA60	0.007626989	
DDX19B	0.007626989	
PON1	0.007626989	
UTP14A	0.007626989	
FGD6	0.007626989	
ALOX5	0.007626989	
PFDN6	0.007626989	
CD93	0.007626989	
CDA	0.007626989	
RP4-591C20.9	0.007626989	
SLC7A7	0.007626989	
ADAR	0.007626989	
FKBPL	0.007626989	
MPST	0.007626989	
GAS7	0.007626989	
WDR11	0.007626989	
AMOT	0.007626989	
S100A9	0.007626989	
CTTN	0.007576143	
SORBS3	0.007576143	
PI4K2B	0.007576143	
EMC7	0.007576143	
FEM1A	0.007576143	
APOA5	0.007576143	
KIF18B	0.007576143	
TEK	0.007576143	
UTP20	0.007576143	
RAD54L	0.007576143	
SLC25A4	0.007576143	
GPS1	0.007576143	
RARS	0.007576143	
MAP1A	0.007576143	
C1orf35	0.007576143	
ADH1B	0.007576143	
PTPN12	0.007576143	
RNF38	0.007576143	
CLK2	0.007576143	
IGFBP7	0.007576143	
PDE3A	0.007576143	
CEP170	0.007576143	

BCLAF1	0.007576143	
GOLM1	0.007576143	
GINS2	0.007576143	
PELI2	0.007576143	
ITGAX	0.007576143	
NOP58	0.007576143	
ARHGAP5	0.007576143	
FKBP8	0.007576143	
DNM1	0.007576143	
ZRANB2	0.007576143	
CLIP3	0.007576143	
FUBP3	0.007576143	
OTC	0.007576143	
TP53BP2	0.007576143	
PGM1	0.007576143	
MRPL16	0.007576143	
CHMP1A	0.007576143	
TUBGCP3	0.007576143	
DBF4B	0.007576143	
UGT2B10	0.007576143	
PSMD6	0.007576143	
TROAP	0.007576143	
COL5A1	0.007576143	
GCC1	0.007576143	
MRPL44	0.007576143	
DDX21	0.007576143	
PCGF2	0.007576143	
GSTO1	0.007576143	
CHST12	0.007576143	
RABL6	0.007576143	
CMTM3	0.007576143	
VSNL1	0.007576143	
ELAC2	0.007576143	
DZIP3	0.007525296	
NBL1	0.007525296	
RAB11FIP2	0.007525296	
TRIP4	0.007525296	
FBLN1	0.007525296	
TAF6L	0.007525296	
SBF2	0.007525296	
EPB41L5	0.007525296	

MT-ND4	0.007525296	
TAOK2	0.007525296	
MZT2A	0.007525296	
CACNB3	0.007525296	
SPC25	0.007525296	
LGR5	0.007525296	
PAPOLG	0.007525296	
LINC00662	0.007525296	
MOB3A	0.007525296	
STX11	0.007525296	
STOM	0.007525296	
RBM12B	0.007525296	
SNX20	0.007525296	
CENPE	0.007525296	
MMS22L	0.007525296	
F8	0.007525296	
PRDX4	0.007525296	
COG6	0.007525296	
FCER1G	0.007525296	
GUCY1A2	0.007525296	
PDIA6	0.007525296	
WDHD1	0.007525296	
SH3GL3	0.007525296	
PAFAH1B3	0.007525296	
RPL22	0.007525296	
HMGN1	0.007525296	
PDE4D	0.007525296	
PDCD5	0.007525296	
GNA13	0.007525296	
GTF3C3	0.007525296	
AIRE	0.007525296	
MT-ND6	0.007525296	
SPN	0.007525296	
HK1	0.007525296	
ABCA6	0.00747445	
TAF1A	0.00747445	
USP33	0.00747445	
ADH1C	0.00747445	
GAB3	0.00747445	
PIP5K1A	0.00747445	
IRF5	0.00747445	

RBMS3	0.00747445	
SPAG5	0.00747445	
MRPS25	0.00747445	
SYTL2	0.00747445	
CEP350	0.00747445	
CCDC53	0.00747445	
GPX2	0.00747445	
APOA1BP	0.00747445	
RCHY1	0.00747445	
MOB1A	0.00747445	
HSPB1P1	0.00747445	
RP11-384L8.1	0.00747445	
RP11-676M6.1	0.00747445	
SCRIB	0.00747445	
POLR3D	0.00747445	
LTBP4	0.00747445	
MKL2	0.00747445	
UBE3C	0.00747445	
CYB5R1	0.00747445	
MRPL53	0.00747445	
ACMSD	0.00747445	
GPAM	0.00747445	
CES2	0.00747445	
G2E3	0.00747445	
SIPA1L3	0.00747445	
ACP2	0.00747445	
FCGR2A	0.00747445	
C9orf142	0.007423603	
LGALS1	0.007423603	
PEAK1	0.007423603	
ITGA2	0.007423603	
HLA-DMB	0.007423603	
TFG	0.007423603	
GNL3L	0.007423603	
C1orf50	0.007423603	
PCK1	0.007423603	
PCTP	0.007423603	
GGA1	0.007423603	
LPXN	0.007423603	
DNAJC25	0.007423603	
CLNS1A	0.007423603	

INCA1	0.007423603	
PTPRE	0.007423603	
QTRT1	0.007423603	
MAP2	0.007423603	
C11orf71	0.007423603	
MAP2K5	0.007423603	
LOXL1	0.007423603	
SRM	0.007423603	
CCDC14	0.007423603	
SLC38A3	0.007423603	
GTF3A	0.007423603	
TTC5	0.007423603	
CKAP2L	0.007423603	
ZNHIT3	0.007423603	
PARD3	0.007423603	
ZC3H11A	0.007423603	
TH	0.007423603	
TBCCD1	0.007423603	
SLC25A21	0.007423603	
NCOA7	0.007423603	
PHYH	0.007423603	
C16orf91	0.007423603	
POLR3F	0.007423603	
MRPL4	0.007423603	
USP25	0.007423603	
GLYAT	0.007423603	
AK3	0.007423603	
FNBP4	0.007423603	
ASGR2	0.007423603	
USP46	0.007372756	
G3BP1	0.007372756	
MLLT3	0.007372756	
KISS1	0.007372756	
43714	0.007372756	
PPIB	0.007372756	
DSN1	0.007372756	
PFDN4	0.007372756	
HOXC11	0.007372756	
UEVLD	0.007372756	
MBD1	0.007372756	
GPX4	0.007372756	

KANSL1L	0.007372756	
SLC25A11	0.007372756	
C4BPB	0.007372756	
RTN3	0.007372756	
IGFBP3	0.007372756	
SWI5	0.007372756	
BRD1	0.007372756	
ZMAT2	0.007372756	
SIGLEC10	0.007372756	
RP11-545I5.3	0.007372756	
CEBPZ	0.007372756	
HAND1	0.007372756	
HOXA13	0.007372756	
EMB	0.007372756	
ERC1	0.007372756	
DMRTB1	0.007372756	
HMGXB4	0.007372756	
UBE2E2	0.007372756	
CHST3	0.007372756	
C19orf53	0.007372756	
PEX3	0.007372756	
PFKFB3	0.007372756	
RHOB	0.007372756	
CRADD	0.007372756	
ARID4B	0.007372756	
MAN2A2	0.007372756	
ALDH5A1	0.007372756	
WAC	0.007372756	
NDUFS2	0.007372756	
MAPT	0.007372756	
HIST4H4	0.007372756	
INHBC	0.007372756	
ZBTB4	0.00732191	
NUDT3	0.00732191	
UBE2V1	0.00732191	
CCNK	0.00732191	
SH2D2A	0.00732191	
PPIG	0.00732191	
ASAH1	0.00732191	
RNF168	0.00732191	
BRCC3	0.00732191	

L2HGDH	0.00732191	
FLNC	0.00732191	
KRT8P3	0.00732191	
EPN2	0.00732191	
TBC1D10B	0.00732191	
KIAA0930	0.00732191	
SSFA2	0.00732191	
AGTR1	0.00732191	
CD37	0.00732191	
TXN	0.00732191	
RMND5A	0.00732191	
MYSM1	0.00732191	
EEF1E1	0.00732191	
ARHGDIA	0.00732191	
FAM168A	0.00732191	
UBE2T	0.00732191	
PPP6C	0.00732191	
GPRASP1	0.00732191	
LIG1	0.00732191	
EPS8	0.00732191	
MED27	0.00732191	
AP5B1	0.00732191	
CLPP	0.00732191	
SFXN5	0.00732191	
RP11-1151B14.3	0.00732191	
FOXP2	0.00732191	
ATIC	0.00732191	
TMEM223	0.00732191	
TGFB3	0.00732191	
FNTA	0.00732191	
ANP32A	0.00732191	
MYH10	0.00732191	
PLK4	0.00732191	
ATP2A3	0.00732191	
ILDR2	0.00732191	
NSMCE2	0.00732191	
CSGALNACT1	0.00732191	
MED28	0.00732191	
NLK	0.00732191	
PPP1R14A	0.00732191	
AC012146.7	0.00732191	

NDUFV3	0.007271063	
CFLAR	0.007271063	
SNAP23	0.007271063	
PSME2	0.007271063	
RAB8B	0.007271063	
DR1	0.007271063	
ISG20L2	0.007271063	
SND1	0.007271063	
ADM	0.007271063	
SLC17A1	0.007271063	
ECSIT	0.007271063	
GGT7	0.007271063	
GLUD1	0.007271063	
RALA	0.007271063	
PARK2	0.007271063	
PRADC1	0.007271063	
SH2D1A	0.007271063	
NDUFA9	0.007271063	
CREB3L4	0.007271063	
RPL36AL	0.007271063	
RP11-465N4.4	0.007271063	
RAB1B	0.007271063	
P4HB	0.007271063	
TMEM222	0.007271063	
TGM3	0.007271063	
NRAS	0.007271063	
RELT	0.007271063	
IL2RA	0.007271063	
DLG2	0.007271063	
SLC35D1	0.007271063	
EPM2AIP1	0.007271063	
GIT2	0.007271063	
KCNS3	0.007271063	
DNAJC8	0.007271063	
IBTK	0.007271063	
ACSM5	0.007271063	
KPNA5	0.007271063	
C1orf54	0.007271063	
SENP6	0.007271063	
NT5DC1	0.007271063	
ZNF358	0.007271063	

C7orf55	0.007271063	
TBCD	0.007220217	
SLTM	0.007220217	
KIFC1	0.007220217	
IGF1	0.007220217	
PTCH1	0.007220217	
PRKRA	0.007220217	
MED30	0.007220217	
ZBTB25	0.007220217	
VCAN	0.007220217	
CHMP3	0.007220217	
NRG1	0.007220217	
DBP	0.007220217	
NUP93	0.007220217	
STAMBP	0.007220217	
WDR61	0.007220217	
TSPO	0.007220217	
ORM1	0.007220217	
ACACA	0.007220217	
EME1	0.007220217	
SLC2A2	0.007220217	
PABPN1	0.007220217	
MZB1	0.007220217	
IL6	0.007220217	
MKL1	0.007220217	
NCAPG2	0.007220217	
TBCB	0.007220217	
CNNM4	0.007220217	
CARM1	0.007220217	
CHCHD2	0.007220217	
SEL1L3	0.007220217	
PUS1	0.007220217	
NDUFS7	0.007220217	
DDT	0.007220217	
AGXT2	0.007220217	
WDR76	0.007220217	
PDK4	0.007220217	
HAUS7	0.007220217	
GCHFR	0.007220217	
PDE2A	0.007220217	
SOX5	0.007220217	

BAMBI	0.007220217	
IER3	0.007220217	
RECQL4	0.007220217	
AZI2	0.007220217	
RNF19A	0.007220217	
COL12A1	0.007220217	
ARL3	0.007220217	
TMEM175	0.007220217	
WHSC1	0.007220217	
KLHL15	0.00716937	
CD6	0.00716937	
MOGAT2	0.00716937	
APOBEC3C	0.00716937	
PROSER3	0.00716937	
PTBP1	0.00716937	
SLC25A15	0.00716937	
LENG8	0.00716937	
ADCY5	0.00716937	
MECR	0.00716937	
OXLD1	0.00716937	
MUT	0.00716937	
NTHL1	0.00716937	
C22orf29	0.00716937	
HOOK2	0.00716937	
CD248	0.00716937	
GBA	0.00716937	
THBS2	0.00716937	
ZNF292	0.00716937	
SLC25A26	0.00716937	
RFWD2	0.00716937	
PIGS	0.00716937	
EXOC7	0.00716937	
INADL	0.00716937	
PDF	0.00716937	
STXBP3	0.00716937	
UBN2	0.00716937	
NTRK1	0.00716937	
WASF1	0.00716937	
SSC5D	0.00716937	
U2AF1L4	0.00716937	
SH3PXD2B	0.00716937	

FAM214B	0.00716937	
MED25	0.00716937	
MBIP	0.00716937	
WNT2B	0.00716937	
ST3GAL4	0.00716937	
B3GALT6	0.00716937	
MTR	0.00716937	
COX7B	0.00716937	
NDUFA12	0.007118523	
CINP	0.007118523	
AOC3	0.007118523	
SAP130	0.007118523	
HAL	0.007118523	
TCP11L2	0.007118523	
KIF20B	0.007118523	
CD3EAP	0.007118523	
SH3GL1	0.007118523	
C1orf43	0.007118523	
ZNF232	0.007118523	
IL6R	0.007118523	
PPP2R5A	0.007118523	
PRPF3	0.007118523	
EIF2A	0.007118523	
BRK1	0.007118523	
FER	0.007118523	
EDC4	0.007118523	
ENOX2	0.007118523	
HDHD2	0.007118523	
TANC1	0.007118523	
TNK2	0.007118523	
ARMCX2	0.007118523	
GNPNAT1	0.007118523	
C17orf53	0.007118523	
43529	0.007118523	
PTPLAD1	0.007118523	
SORD	0.007118523	
MC1R	0.007118523	
FAM46A	0.007118523	
PFN2	0.007118523	
DECR2	0.007118523	
MARVELD1	0.007118523	

TNFSF11	0.007118523	
RGS4	0.007118523	
MRPS9	0.007118523	
PICALM	0.007118523	
FRG1	0.007118523	
HCFC1R1	0.007067677	
ZNF37BP	0.007067677	
AP3M1	0.007067677	
NCAPH	0.007067677	
PNISR	0.007067677	
TIMM22	0.007067677	
NCEH1	0.007067677	
COPE	0.007067677	
GPRASP2	0.007067677	
RP4-706A16.3	0.007067677	
HIST1H3H	0.007067677	
PNKP	0.007067677	
ZNF655	0.007067677	
JAM3	0.007067677	
TCOF1	0.007067677	
SLAMF7	0.007067677	
CBFA2T3	0.007067677	
AKR1C4	0.007067677	
PSAT1	0.007067677	
LINC01197	0.007067677	
KATNB1	0.007067677	
WDFY3	0.007067677	
SDHC	0.007067677	
CDYL	0.007067677	
ADAMTS1	0.007067677	
CCL4	0.007067677	
HSDL2	0.007067677	
PRPF19	0.007067677	
DNAJC16	0.007067677	
KCNB1	0.007067677	
DDX46	0.007067677	
DBN1	0.007067677	
CFB	0.007067677	
LGALS3	0.007067677	
ORC4	0.007067677	
RPIA	0.007067677	

LDB2	0.007067677	
SLC16A1	0.007067677	
PSMC6	0.007067677	
TRAF3	0.007067677	
EMC3	0.007067677	
SGSM2	0.007067677	
RALGDS	0.007067677	
PWP1	0.007067677	
PTPN7	0.007067677	
NDUFS1	0.007067677	
GYPC	0.007067677	
SMN2	0.007067677	
CHD1L	0.007067677	
TRPV2	0.00701683	
ARAF	0.00701683	
PLSCR4	0.00701683	
NUAK1	0.00701683	
PTRH2	0.00701683	
FBXW5	0.00701683	
POLDIP2	0.00701683	
CTSB	0.00701683	
STARD10	0.00701683	
ZBTB22	0.00701683	
HEXIM2	0.00701683	
CCND2P1	0.00701683	
SSBP1	0.00701683	
MRPL22	0.00701683	
AIMP2	0.00701683	
SERBP1	0.00701683	
TERF2IP	0.00701683	
RAB3GAP1	0.00701683	
FAM35DP	0.00701683	
CP	0.00701683	
RP11-70D24.2	0.00701683	
TLN1	0.00701683	
MEA1	0.00701683	
UBR5	0.00701683	
AZGP1	0.00701683	
RABGAP1	0.00701683	
MKI67	0.00701683	
BMP2	0.00701683	

SF3A3	0.00701683	
HK3	0.00701683	
RCAN2	0.00701683	
PDZD8	0.00701683	
PTPRB	0.00701683	
VIP	0.00701683	
TMEM131	0.00701683	
HACE1	0.00701683	
FETUB	0.00701683	
NUDCD1	0.00701683	
CENPB	0.00701683	
LILRB2	0.00701683	
MED16	0.00701683	
CCNH	0.00701683	
DDX17	0.00701683	
RBM22	0.00701683	
RGL2	0.00701683	
PDHA1	0.00701683	
SHMT1	0.00701683	
LIMS1	0.00701683	
FOXP3	0.00701683	
CCAR1	0.00701683	
SNAP29	0.00701683	
TMEM87A	0.00701683	
MRPS27	0.006965984	
DIAPH1	0.006965984	
AP3B1	0.006965984	
SCD	0.006965984	
LHFP	0.006965984	
OPRM1	0.006965984	
TMEM110	0.006965984	
BCAS2	0.006965984	
PXMP2	0.006965984	
CSK	0.006965984	
NSD1	0.006965984	
CTB-63M22.1	0.006965984	
AGT	0.006965984	
GPS2	0.006965984	
NLE1	0.006965984	
C1QB	0.006965984	
ATP5H	0.006965984	

KIRREL	0.006965984	
RRM1	0.006965984	
RPA3	0.006965984	
NEK3	0.006965984	
TRIM11	0.006965984	
ARPC5L	0.006965984	
ISLR	0.006965984	
AGAP2-AS1	0.006965984	
IL10	0.006965984	
HSPH1	0.006965984	
NRP2	0.006965984	
FCGR1A	0.006965984	
NDUFA5	0.006965984	
WDR27	0.006965984	
TNXB	0.006965984	
RNF7	0.006965984	
LAMTOR5	0.006965984	
DPYS	0.006965984	
LINC01348	0.006965984	
UGT1A6	0.006965984	
FAM58A	0.006965984	
GAMT	0.006965984	
LAMA3	0.006915137	
SH3BP4	0.006915137	
APOBR	0.006915137	
KIAA0101	0.006915137	
LRBA	0.006915137	
VAMP5	0.006915137	
BHMT	0.006915137	
NUDT18	0.006915137	
GNRH1	0.006915137	
MAP2K3	0.006915137	
NDUFC1	0.006915137	
PIK3R2	0.006915137	
NMT1	0.006915137	
CENPT	0.006915137	
HMOX1	0.006915137	
ARIH2	0.006915137	
GBP7	0.006915137	
ATP5B	0.006915137	
CNOT6L	0.006915137	

CCNT2	0.006915137	
TBXA2R	0.006915137	
FMO5	0.006915137	
COX5A	0.006915137	
PRDX6	0.006915137	
TRIM22	0.006915137	
CCDC57	0.006915137	
MTPN	0.006915137	
CFD	0.006915137	
GLYR1	0.006915137	
SLC2A4	0.006915137	
MTM1	0.006915137	
UBR3	0.006915137	
TRAP1	0.006915137	
CCNF	0.006915137	
IGBP1	0.006915137	
NDUFA4L2	0.006915137	
SLC30A1	0.006915137	
AHCYL2	0.006915137	
TRMT10C	0.006915137	
GHR	0.006915137	
QARS	0.006915137	
PIH1D1	0.006915137	
RP11-798K3.2	0.006915137	
PPP2R2A	0.006915137	
SPEG	0.006915137	
RRAS2	0.006915137	
TRAF5	0.00686429	
GOLGA5	0.00686429	
RP13-582O9.5	0.00686429	
CTD-2396E7.11	0.00686429	
ADCK5	0.00686429	
ABCC6P1	0.00686429	
IFNB1	0.00686429	
NT5E	0.00686429	
CCT2	0.00686429	
MRPL10	0.00686429	
TAGLN	0.00686429	
ACHE	0.00686429	
SLC25A18	0.00686429	
IGF2BP1	0.00686429	

PNO1	0.00686429	
GRINA	0.00686429	
GPSM2	0.00686429	
CPT1A	0.00686429	
GNAI3	0.00686429	
PTBP3	0.00686429	
ECI2	0.00686429	
C19orf25	0.00686429	
MBTD1	0.00686429	
EPHX2	0.00686429	
TIRAP	0.00686429	
TUT1	0.00686429	
SHH	0.00686429	
SHANK3	0.00686429	
SCARB1	0.00686429	
MED22	0.00686429	
GNPDA1	0.00686429	
DAD1	0.00686429	
CCHCR1	0.00686429	
PLTP	0.00686429	
RPL7P9	0.00686429	
GSPT1	0.00686429	
PLD1	0.00686429	
UBA1	0.00686429	
ABCB11	0.00686429	
BUD31	0.00686429	
BOLA1	0.00686429	
XRCC3	0.00686429	
PPP1R13B	0.00686429	
CDKN2C	0.00686429	
RAD23A	0.00686429	
CFAP57	0.00686429	
SAFB	0.006813444	
U2AF1	0.006813444	
ARIH1	0.006813444	
ALDH3A1	0.006813444	
MT-CO2	0.006813444	
RGS14	0.006813444	
SP5	0.006813444	
GTF2H3	0.006813444	
POLD4	0.006813444	

LXN	0.006813444	
GNA12	0.006813444	
KLF9	0.006813444	
PSMB8	0.006813444	
DLD	0.006813444	
TMEM147	0.006813444	
HADHB	0.006813444	
ABCC10	0.006813444	
TOP1MT	0.006813444	
COPS4	0.006813444	
GTF3C5	0.006813444	
CYP2J2	0.006813444	
SPERT	0.006813444	
PEF1	0.006813444	
MERTK	0.006813444	
TBL1X	0.006813444	
SERPINF2	0.006813444	
GBP5	0.006813444	
SDCBP2	0.006813444	
PCBP2	0.006813444	
SGK2	0.006813444	
MT-ND1	0.006813444	
NCAPD2	0.006813444	
NAGLU	0.006813444	
ORMDL2	0.006813444	
ADCY6	0.006813444	
CLEC7A	0.006813444	
DCAF5	0.006813444	
ZNF503	0.006813444	
VPS18	0.006813444	
DES	0.006813444	
PYCARD	0.006813444	
CTSF	0.006813444	
ABHD14B	0.006813444	
RP11-611O2.2	0.006813444	
ACVR1B	0.006813444	
TMEM33	0.006813444	
RPL41	0.006813444	
MRPS12	0.006813444	
GDF9	0.006813444	
CD276	0.006813444	

MARK4	0.006813444	
CALHM2	0.006813444	
MRPL39	0.006813444	
BCL10	0.006813444	
CWC27	0.006813444	
C4A	0.006813444	
C19orf60	0.006813444	
PHF19	0.006813444	
RBM25	0.006813444	
BCAS4	0.006813444	
ALDH1A3	0.006762597	
TLE2	0.006762597	
LGALS9	0.006762597	
MAN1B1	0.006762597	
TIMELESS	0.006762597	
ARFGEF1	0.006762597	
GSTA7P	0.006762597	
IRF6	0.006762597	
SLC25A53	0.006762597	
SERPINB6	0.006762597	
RSL1D1	0.006762597	
DYNLT1	0.006762597	
PLEKHA7	0.006762597	
GPR132	0.006762597	
EIF3C	0.006762597	
FAM83H	0.006762597	
SDHD	0.006762597	
RAB3IP	0.006762597	
NXT1	0.006762597	
GPR56	0.006762597	
SIVA1	0.006762597	
MT-TP	0.006762597	
TAGAP	0.006762597	
SDF2	0.006762597	
GLTP	0.006762597	
HAUS1	0.006762597	
MAP1LC3A	0.006762597	
ASL	0.006762597	
CKS1B	0.006762597	
PTGIR	0.006762597	
IL27	0.006762597	

ASAP1	0.006762597	
TMEM199	0.006762597	
TFB2M	0.006762597	
CPSF1	0.006762597	
CRISPLD2	0.006762597	
HSPA2	0.006762597	
AP4E1	0.006762597	
LRRC59	0.006762597	
SPATS2	0.006762597	
AP1S2	0.006762597	
IMP4	0.006762597	
TTN	0.006762597	
NR4A3	0.006762597	
IMP3	0.006762597	
TXNIP	0.006762597	
TTC27	0.006762597	
SSR1	0.006762597	
RAD9A	0.006762597	
SLC1A2	0.006762597	
GLIS3	0.006762597	
RPL3P4	0.006762597	
UBE2Q1	0.006762597	
CHKA	0.006762597	
FST	0.006762597	
THOC7	0.006762597	
ABCD3	0.006711751	
ATP1B1	0.006711751	
BDH1	0.006711751	
SEMA4D	0.006711751	
SNRPA1	0.006711751	
PLCB1	0.006711751	
GPN1	0.006711751	
CH17-340M24.3	0.006711751	
WWTR1	0.006711751	
POLE2	0.006711751	
AMDHD1	0.006711751	
PAK1IP1	0.006711751	
TMBIM4	0.006711751	
RP11-730G20.2	0.006711751	
TMEM56	0.006711751	
USP12	0.006711751	

OSBPL8	0.006711751	
NOV	0.006711751	
LIFR	0.006711751	
MRPS23	0.006711751	
SEC13	0.006711751	
SELL	0.006711751	
TNPO3	0.006711751	
MFAP1	0.006711751	
LYST	0.006711751	
DHCR7	0.006711751	
COL6A2	0.006711751	
ASCC3	0.006711751	
CXADR	0.006711751	
HRSP12	0.006711751	
TIAM1	0.006711751	
COBLL1	0.006711751	
WDR47	0.006711751	
GPR65	0.006711751	
STAU1	0.006711751	
COQ10A	0.006711751	
PDE1B	0.006711751	
SERPINA4	0.006711751	
SYNE2	0.006660904	
CNNM3	0.006660904	
ABHD6	0.006660904	
UBXN6	0.006660904	
CTU2	0.006660904	
HNF1B	0.006660904	
APOF	0.006660904	
TCF20	0.006660904	
ITIH2	0.006660904	
SSBP4	0.006660904	
AADAC	0.006660904	
ZBTB10	0.006660904	
SDAD1	0.006660904	
GUCY1A3	0.006660904	
SMCHD1	0.006660904	
LAMA5	0.006660904	
IPPK	0.006660904	
CREB3L2	0.006660904	
ARHGAP11A	0.006660904	

AP2A1	0.006660904	
SH3KBP1	0.006660904	
RAB27A	0.006660904	
ZNF8	0.006660904	
IFIT3	0.006660904	
UBA5	0.006660904	
TMPO	0.006660904	
ELOF1	0.006660904	
RPS26	0.006660904	
KCTD1	0.006660904	
SPATA24	0.006660904	
OTX1	0.006660904	
ARHGEF5	0.006660904	
RSBN1	0.006660904	
PSMG4	0.006660904	
KDM5A	0.006660904	
SMDT1	0.006660904	
BRSK1	0.006660904	
NFE2L3	0.006660904	
CD164	0.006660904	
GEMIN2	0.006660904	
ATXN2	0.006660904	
ACTC1	0.006660904	
PLEC	0.006610057	
DAPK1	0.006610057	
NCAPD3	0.006610057	
PIN4	0.006610057	
HOXC9	0.006610057	
ZC4H2	0.006610057	
SLC13A5	0.006610057	
ORM2	0.006610057	
POLR2M	0.006610057	
TRAF3IP3	0.006610057	
ROM1	0.006610057	
CACNA1C	0.006610057	
FLII	0.006610057	
ACAA1	0.006610057	
USP45	0.006610057	
ADH1A	0.006610057	
ZCCHC10	0.006610057	
RFC2	0.006610057	

FOXP1	0.006610057	
ZFPM1	0.006610057	
APOOL	0.006610057	
SIPA1	0.006610057	
DPYD	0.006610057	
ZFP41	0.006610057	
GALT	0.006610057	
ARHGEF12	0.006610057	
KCTD12	0.006610057	
HERC1	0.006610057	
ZBTB24	0.006610057	
RALGAPA2	0.006610057	
RP11-379B8.1	0.006610057	
KLF13	0.006610057	
CYTH4	0.006610057	
ZNF165	0.006610057	
LETM1	0.006610057	
CENPW	0.006610057	
SSR2	0.006610057	
SUCLG2	0.006610057	
DAPP1	0.006610057	
MTHFS	0.006610057	
CAMK4	0.006610057	
MAD2L2	0.006610057	
SLPI	0.006610057	
TXNDC11	0.006610057	
GGCX	0.006610057	
UNC5C	0.006610057	
TFF1	0.006610057	
EIF2S2	0.006610057	
KCNIP3	0.006610057	
MAST1	0.006610057	
COX6A1	0.006559211	
PSG2	0.006559211	
ST3GAL1	0.006559211	
COPB1	0.006559211	
GSTA1	0.006559211	
LAIR1	0.006559211	
BIN2	0.006559211	
RPP25L	0.006559211	
SEPSECS	0.006559211	

WBP4	0.006559211	
SKA3	0.006559211	
PECAM1	0.006559211	
ZNF496	0.006559211	
SH3GLB2	0.006559211	
ST6GAL1	0.006559211	
PCED1B-AS1	0.006559211	
TEC	0.006559211	
ADCY4	0.006559211	
DOPEY1	0.006559211	
CCT6A	0.006559211	
RPL7P1	0.006559211	
LILRB1	0.006559211	
ASXL2	0.006559211	
FGL1	0.006559211	
LHFPL2	0.006559211	
PHOX2A	0.006559211	
PER3	0.006559211	
DDX24	0.006559211	
RIN1	0.006559211	
CBLB	0.006559211	
CPEB3	0.006559211	
MSMO1	0.006559211	
CCDC94	0.006559211	
RIC1	0.006559211	
NAA15	0.006559211	
MED18	0.006559211	
CEP97	0.006559211	
ZZZ3	0.006559211	
ANPEP	0.006559211	
FAM149A	0.006559211	
CSNK1G1	0.006559211	
ACBD3	0.006559211	
RP4-710M16.2	0.006559211	
INTS8	0.006559211	
SAMSN1	0.006559211	
FOXG1	0.006559211	
RNF43	0.006559211	
TWF2	0.006559211	
HOOK1	0.006559211	
CPNE1	0.006559211	

PTPN22	0.006559211	
MYOC	0.006559211	
MOV10	0.006559211	
RANGRF	0.006559211	
FKBP3	0.006559211	
ZBTB37	0.006559211	
NBEAL2	0.006559211	
CSRP1	0.006559211	
FAM49B	0.006559211	
CAPRIN1	0.006559211	
TUSC8	0.006559211	
TNFSF13B	0.006559211	
MTCH1	0.006508364	
GCKR	0.006508364	
CDIPT	0.006508364	
TMCO1	0.006508364	
IVD	0.006508364	
COL5A2	0.006508364	
WDR4	0.006508364	
HEG1	0.006508364	
UBD	0.006508364	
THY1	0.006508364	
RAD23B	0.006508364	
MAP7	0.006508364	
BAG1	0.006508364	
MPDU1	0.006508364	
IGFBP4	0.006508364	
DCLRE1B	0.006508364	
C14orf79	0.006508364	
WDR66	0.006508364	
PER2	0.006508364	
PTGR1	0.006508364	
MKLN1	0.006508364	
PQLC1	0.006508364	
RPP21	0.006508364	
SDC4	0.006508364	
F11-AS1	0.006508364	
EBP	0.006508364	
CYTH3	0.006508364	
CYLD	0.006508364	
RLF	0.006508364	

ENO3	0.006508364	
PRDX2	0.006508364	
NEU1	0.006508364	
SNHG18	0.006508364	
C16orf59	0.006508364	
OSGEP	0.006508364	
RASGRP1	0.006508364	
DIP2B	0.006508364	
DNTTIP2	0.006508364	
TPD52L2	0.006508364	
PI3	0.006508364	
SORBS2	0.006508364	
SCCPDH	0.006508364	
FAP	0.006508364	
FMO4	0.006508364	
PRCC	0.006508364	
STAM2	0.006508364	
MYL6B	0.006508364	
MSRB2	0.006508364	
RP11-704M14.1	0.006508364	
TMEM51	0.006457518	
NPRL2	0.006457518	
GABBR2	0.006457518	
TOMM7	0.006457518	
43525	0.006457518	
KCNC4	0.006457518	
AFM	0.006457518	
OXCT1	0.006457518	
UPP1	0.006457518	
DACT3	0.006457518	
CLEC11A	0.006457518	
PROX1-AS1	0.006457518	
RNGTT	0.006457518	
RICTOR	0.006457518	
MRPS14	0.006457518	
MTBP	0.006457518	
AKR1C1	0.006457518	
TADA1	0.006457518	
CRY2	0.006457518	
C14orf105	0.006457518	
UNC45A	0.006457518	

OSTF1	0.006457518	
HAUS6	0.006457518	
CPSF3	0.006457518	
AEBP1	0.006457518	
NRBF2	0.006457518	
HSP90B2P	0.006457518	
SCAPER	0.006457518	
RNF113A	0.006457518	
CHST11	0.006457518	
TCEB3	0.006457518	
KIAA1841	0.006457518	
RPS27L	0.006457518	
CDCA2	0.006457518	
C1orf122	0.006457518	
POLQ	0.006457518	
ACSL1	0.006457518	
MYO1F	0.006457518	
SNRNP27	0.006457518	
EBI3	0.006457518	
KHDRBS2	0.006457518	
SNF8	0.006457518	
SOX6	0.006457518	
ARFGAP1	0.006457518	
POR	0.006457518	
CAPZA1	0.006457518	
CD96	0.006457518	
SCML1	0.006457518	
TMPRSS6	0.006457518	
TEAD2	0.006457518	
C4B	0.006457518	
CD180	0.006457518	
KAZN	0.006457518	
GRIN1	0.006457518	
ABCC6	0.006406671	
SEPHS2	0.006406671	
GNG7	0.006406671	
UBE2F	0.006406671	
ABLIM1	0.006406671	
HMGCS1	0.006406671	
PMEL	0.006406671	
CTNNBL1	0.006406671	

DHX16	0.006406671	
HFE2	0.006406671	
ZBED4	0.006406671	
PCDHGC3	0.006406671	
THEMIS2	0.006406671	
SLC7A2	0.006406671	
MRPL46	0.006406671	
C6orf1	0.006406671	
COL14A1	0.006406671	
DTX1	0.006406671	
SRRT	0.006406671	
HERC6	0.006406671	
STAG3L2	0.006406671	
TRIM33	0.006406671	
TMEM104	0.006406671	
EPM2A	0.006406671	
SRSF6	0.006406671	
KDM6A	0.006406671	
HNRNPH2	0.006406671	
AKR1B1	0.006406671	
ITIH1	0.006406671	
CHD1	0.006406671	
RGN	0.006406671	
HLA-DQA1	0.006406671	
PKP4	0.006406671	
POLE	0.006406671	
RP11-15I11.3	0.006406671	
RGS3	0.006406671	
MRPS33	0.006406671	
RB1CC1	0.006406671	
MRPS18B	0.006406671	
AKR7A2	0.006406671	
COL5A3	0.006406671	
MED20	0.006406671	
TGOLN2	0.006406671	
ATXN7L3	0.006406671	
NUP214	0.006406671	
F11R	0.006406671	
APBA2	0.006406671	
PPCS	0.006406671	
HMGN2	0.006406671	

AHSA2	0.006406671	
IGSF21	0.006406671	
RP11-428K3.1	0.006406671	
43712	0.006406671	
SHOC2	0.006406671	
TRIB2	0.006355824	
TARS	0.006355824	
LRRC32	0.006355824	
CFHR4	0.006355824	
SNX27	0.006355824	
FAM117B	0.006355824	
ZFAND5	0.006355824	
WASH1	0.006355824	
ALDH18A1	0.006355824	
AP1B1	0.006355824	
CBFB	0.006355824	
CRNKL1	0.006355824	
VAMP1	0.006355824	
C4orf29	0.006355824	
ZBTB20	0.006355824	
PSMD10	0.006355824	
PHAX	0.006355824	
MECOM	0.006355824	
GAL3ST4	0.006355824	
CEP89	0.006355824	
PLEKHH2	0.006355824	
SH3D19	0.006355824	
ZMAT5	0.006355824	
ZSCAN1	0.006355824	
RFC5	0.006355824	
LMO1	0.006355824	
CNTRL	0.006355824	
MID1	0.006355824	
CYP11B1	0.006355824	
GPLD1	0.006355824	
CYP2C8	0.006355824	
TRA2B	0.006355824	
SETD1A	0.006355824	
FBLIM1	0.006355824	
GCK	0.006355824	
SLC22A9	0.006355824	

TBC1D23	0.006355824	
RASSF3	0.006355824	
PUSL1	0.006355824	
SRFBP1	0.006355824	
PTCD3	0.006355824	
RPL18AP3	0.006355824	
PMPCA	0.006355824	
REXO2	0.006355824	
MSR1	0.006355824	
ZNF81	0.006355824	
CCL3	0.006355824	
ARL13B	0.006355824	
CYP2A6	0.006355824	
HEXB	0.006355824	
SLC50A1	0.006355824	
PHLPP1	0.006355824	
EFS	0.006355824	
ARCN1	0.006355824	
APTX	0.006355824	
ZFAND2B	0.006355824	
RP11-792A8.4	0.006355824	
GRID1	0.006355824	
C2CD2	0.006355824	
PARD6B	0.006355824	
POLH	0.006304978	
SARNP	0.006304978	
MRFAP1	0.006304978	
MDK	0.006304978	
ARL16	0.006304978	
CCL14	0.006304978	
RNPEPL1	0.006304978	
TAX1BP1	0.006304978	
MYO1B	0.006304978	
CES1	0.006304978	
RAP1GAP	0.006304978	
FRZB	0.006304978	
FAM207A	0.006304978	
EHF	0.006304978	
ALOX5AP	0.006304978	
RFX2	0.006304978	
ITFG1	0.006304978	

PPFIBP1	0.006304978	
TNIP2	0.006304978	
CDC25B	0.006304978	
NDUFA6	0.006304978	
RAB10	0.006304978	
RBM42	0.006304978	
RYR1	0.006304978	
SPOPL	0.006304978	
DIEXF	0.006304978	
TM4SF5	0.006304978	
BCKDHB	0.006304978	
ANKS1A	0.006304978	
PJA1	0.006304978	
GNB1	0.006304978	
LDHAP4	0.006304978	
TAF1B	0.006304978	
FABP1	0.006304978	
NUDT14	0.006304978	
ACY1	0.006304978	
CTDSP2	0.006304978	
TTBK1	0.006304978	
LCP1	0.006304978	
TICAM1	0.006304978	
PTPRS	0.006304978	
LRIG3	0.006304978	
HSD17B8	0.006304978	
JARID2	0.006304978	
CNRIP1	0.006304978	
PBXIP1	0.006304978	
SRPR	0.006304978	
SAA1	0.006304978	
DLL4	0.006304978	
GET4	0.006304978	
NUP85	0.006304978	
FASN	0.006304978	
ELANE	0.006304978	
FAM111B	0.006304978	
B4GALT5	0.006304978	
UVRAG	0.006304978	
DMXL2	0.006304978	
RP11-73M18.8	0.006304978	

DYNC1I2	0.006304978	
KRT1	0.006304978	
HDGFRP3	0.006254131	
YPEL3	0.006254131	
ZSCAN12	0.006254131	
ARL2BP	0.006254131	
SLC22A5	0.006254131	
HHLA3	0.006254131	
ANK2	0.006254131	
CHN2	0.006254131	
CDH5	0.006254131	
FBXL18	0.006254131	
LHX4	0.006254131	
RP11-379H18.1	0.006254131	
IL4	0.006254131	
C15orf39	0.006254131	
PRRC2C	0.006254131	
MCM3AP	0.006254131	
BTG1	0.006254131	
MXRA8	0.006254131	
AFP	0.006254131	
HPS6	0.006254131	
IKZF5	0.006254131	
MYO1E	0.006254131	
NEK9	0.006254131	
C17orf89	0.006254131	
RCC1	0.006254131	
CRP	0.006254131	
STK25	0.006254131	
CXCL1	0.006254131	
TARDBP	0.006254131	
EBAG9	0.006254131	
VAPA	0.006254131	
ANG	0.006254131	
FLT4	0.006254131	
AKR1C6P	0.006254131	
AOAH	0.006254131	
WWOX	0.006254131	
KIAA0196	0.006254131	
STX12	0.006254131	
TXNDC17	0.006254131	

EMP3	0.006254131	
PARG	0.006254131	
ECHDC2	0.006254131	
PDCD4	0.006254131	
ADAMTS4	0.006254131	
TFRC	0.006254131	
LBP	0.006254131	
RP11-706C16.7	0.006254131	
CEP63	0.006254131	
LRRC56	0.006254131	
RNASEH2A	0.006254131	
REXO1	0.006254131	
CDKL5	0.006254131	
ANKRD17	0.006254131	
LGALS8	0.006254131	
PTPN2	0.006254131	
MGMT	0.006254131	
SLC41A2	0.006254131	
WASL	0.006254131	
RTP3	0.006254131	
ABCG2	0.006254131	
XPA	0.006254131	
WDR74	0.006254131	
SIGLEC9	0.006254131	
ACAT1	0.006254131	
ZNF462	0.006254131	
HIST1H4F	0.006254131	
MAP3K10	0.006254131	
DHFR	0.006254131	
APPL1	0.006254131	
ZNF155	0.006254131	
SLC47A1	0.006254131	
PEX1	0.006254131	
KCTD21	0.006203285	
MPHOSPH8	0.006203285	
SPX	0.006203285	
FAM8A1	0.006203285	
LHX2	0.006203285	
GART	0.006203285	
NR1D1	0.006203285	
SEPP1	0.006203285	

C14orf166	0.006203285	
AP4M1	0.006203285	
MDH2	0.006203285	
APOM	0.006203285	
PGK1	0.006203285	
CD27	0.006203285	
ACTR3	0.006203285	
TONSL	0.006203285	
RABIF	0.006203285	
SLC35E2B	0.006203285	
DLGAP5	0.006203285	
ETFDH	0.006203285	
TUBB3	0.006203285	
TCF25	0.006203285	
NOVA2	0.006203285	
CBLN3	0.006203285	
SF3B6	0.006203285	
CLDN1	0.006203285	
PUM1	0.006203285	
PBK	0.006203285	
PDCD10	0.006203285	
DCAF17	0.006203285	
FAM26E	0.006203285	
TTPA	0.006203285	
DGCR2	0.006203285	
TCEA1	0.006203285	
ZNF576	0.006203285	
SYT13	0.006203285	
SUPT6H	0.006203285	
KIAA1211L	0.006203285	
FAM124A	0.006203285	
SYNGAP1	0.006203285	
DSE	0.006203285	
METTL1	0.006203285	
ATAD3B	0.006203285	
RAB11B	0.006203285	
NUCKS1	0.006203285	
NTN1	0.006203285	
ARL6IP5	0.006203285	
HSBP1	0.006203285	
DUSP12	0.006203285	

MTF1	0.006203285	
HOXB1	0.006203285	
ZNF445	0.006203285	
INPP4B	0.006203285	
GJB3	0.006203285	
RIPK1	0.006203285	
PLXNA2	0.006203285	
DHRS4L2	0.006203285	
WTIP	0.006203285	
ETNPPL	0.006203285	
NAA40	0.006203285	
KLHL26	0.006152438	
ATXN3	0.006152438	
ZNF619	0.006152438	
KIF14	0.006152438	
TBPL1	0.006152438	
PPM1D	0.006152438	
NRXN1	0.006152438	
PEX2	0.006152438	
ARHGEF2	0.006152438	
CNOT3	0.006152438	
EVI2B	0.006152438	
PTS	0.006152438	
C1S	0.006152438	
HOXB9	0.006152438	
SVIL	0.006152438	
SH3GLB1	0.006152438	
CPNE5	0.006152438	
MUC7	0.006152438	
GNS	0.006152438	
EFNA2	0.006152438	
GLA	0.006152438	
TPM4	0.006152438	
KIF15	0.006152438	
BRWD1	0.006152438	
TUBB4B	0.006152438	
GGA2	0.006152438	
TBC1D8B	0.006152438	
RP11-469A15.2	0.006152438	
RNF123	0.006152438	
PDE6D	0.006152438	

DTX3L	0.006152438	
C8G	0.006152438	
PSME1	0.006152438	
SERTAD2	0.006152438	
ZC3H10	0.006152438	
UBL7	0.006152438	
SH2D3C	0.006152438	
GNRHR	0.006152438	
RCC2	0.006152438	
HSPA1B	0.006152438	
RAMP2	0.006152438	
GPR116	0.006152438	
ZBTB7B	0.006152438	
LPCAT3	0.006152438	
EXOSC6	0.006152438	
NUDCD2	0.006152438	
RPUSD1	0.006152438	
EVI2A	0.006152438	
CYBB	0.006152438	
EIF2S1	0.006152438	
WDR82	0.006152438	
C14orf2	0.006152438	
BCKDHA	0.006152438	
SPNS1	0.006152438	
MAP2K6	0.006152438	
STK39	0.006152438	
KDF1	0.006152438	
LASP1	0.006152438	
C1orf123	0.006152438	
USH1C	0.006152438	
DUSP4	0.006152438	
ZNF142	0.006152438	
UFD1L	0.006101591	
KCTD13	0.006101591	
C1orf131	0.006101591	
APOPT1	0.006101591	
IGF2BP3	0.006101591	
FAM122A	0.006101591	
ELP3	0.006101591	
OSGIN1	0.006101591	
CDC14B	0.006101591	

UBASH3B	0.006101591	
C10orf54	0.006101591	
NDUFB4	0.006101591	
CDCA4	0.006101591	
RAB6A	0.006101591	
FAM110C	0.006101591	
TRIM52-AS1	0.006101591	
ACAP1	0.006101591	
SIRT3	0.006101591	
FMOD	0.006101591	
CLASRP	0.006101591	
SNX13	0.006101591	
UBL4A	0.006101591	
TYR	0.006101591	
SMG8	0.006101591	
LHX1	0.006101591	
DCTPP1	0.006101591	
FLRT2	0.006101591	
ETV5	0.006101591	
MTFR1	0.006101591	
APPL2	0.006101591	
C2orf47	0.006101591	
EPS8L1	0.006101591	
GPNMB	0.006101591	
MAP3K4	0.006101591	
SEMA5A	0.006101591	
ALDH4A1	0.006101591	
TMPRSS2	0.006101591	
TAF13	0.006101591	
STS	0.006101591	
DAZAP1	0.006101591	
FGF1	0.006101591	
FOXF1	0.006101591	
SKIDA1	0.006101591	
TMEM176B	0.006101591	
HPD	0.006101591	
ZNF688	0.006101591	
BLNK	0.006101591	
SP2	0.006101591	
YIPF2	0.006101591	
HSD17B1	0.006101591	

LINC00493	0.006101591	
PKN1	0.006101591	
CTDNEP1	0.006101591	
QDPR	0.006101591	
ZNF146	0.006101591	
MT-ND4L	0.006101591	
BBS7	0.006101591	
CHIC2	0.006050745	
ACACB	0.006050745	
CYTH1	0.006050745	
ATG2A	0.006050745	
PSMD12	0.006050745	
CEP290	0.006050745	
CSF3R	0.006050745	
LINC00574	0.006050745	
HLA-F	0.006050745	
RPN2	0.006050745	
PHACTR4	0.006050745	
DCLK1	0.006050745	
PRKRIP1	0.006050745	
DNAJB9	0.006050745	
THOC1	0.006050745	
PHLPP2	0.006050745	
DCAF7	0.006050745	
LIN7B	0.006050745	
LTBP2	0.006050745	
ALDH8A1	0.006050745	
EXOSC2	0.006050745	
RNASE4	0.006050745	
BNIP3L	0.006050745	
TIE1	0.006050745	
ZNF567	0.006050745	
LRP5	0.006050745	
CD302	0.006050745	
PTCD1	0.006050745	
SORD2P	0.006050745	
PDK2	0.006050745	
COMMD7	0.006050745	
MPP6	0.006050745	
HK2	0.006050745	
MSL2	0.006050745	

C2CD3	0.006050745	
STK36	0.006050745	
SLC38A4	0.006050745	
ABTB2	0.006050745	
RER1	0.006050745	
CYP7A1	0.006050745	
EPHA3	0.006050745	
NAP1L4	0.006050745	
APBB2	0.006050745	
ARHGAP9	0.006050745	
LDLR	0.006050745	
VPS45	0.006050745	
SYT11	0.006050745	
UAP1	0.006050745	
ITFG3	0.006050745	
ASS1	0.006050745	
LPAR2	0.006050745	
ARL4C	0.006050745	
EPS15	0.006050745	
GPATCH2L	0.006050745	
ZBTB8OS	0.006050745	
STOML2	0.006050745	
LRAT	0.006050745	
RAP1B	0.005999898	
USP13	0.005999898	
CHPT1	0.005999898	
ZNF646	0.005999898	
EDEM1	0.005999898	
STK19	0.005999898	
ALOX15	0.005999898	
FAM20A	0.005999898	
ZC3H4	0.005999898	
RPS6KA4	0.005999898	
ATP6V0D1	0.005999898	
SCYL1	0.005999898	
TRIM59	0.005999898	
IL1R1	0.005999898	
SUCLG1	0.005999898	
TLE3	0.005999898	
IDH3A	0.005999898	
PTGS1	0.005999898	

PRTN3	0.005999898	
CEP41	0.005999898	
PAK4	0.005999898	
MCEE	0.005999898	
STX7	0.005999898	
CDR2	0.005999898	
RCSD1	0.005999898	
TRPT1	0.005999898	
SPOP	0.005999898	
CYP1B1	0.005999898	
DSG1	0.005999898	
CPSF3L	0.005999898	
DCK	0.005999898	
NEDD9	0.005999898	
ATP6V1F	0.005999898	
PLXDC1	0.005999898	
MAPK8IP3	0.005999898	
USP10	0.005999898	
TCF7L1	0.005999898	
ZNF510	0.005999898	
GNB2	0.005999898	
HEXA	0.005999898	
PKLR	0.005999898	
SLC24A3	0.005999898	
NQO1	0.005999898	
CSE1L	0.005999898	
GYS2	0.005999898	
HIF1AN	0.005999898	
DGCR14	0.005999898	
MT-ND3	0.005999898	
PTDSS1	0.005999898	
SQRDL	0.005999898	
GDI1	0.005999898	
RAB11A	0.005999898	
BAG2	0.005999898	
RGS19	0.005999898	
MRPS10	0.005999898	
ABCF1	0.005999898	
FAR1	0.005999898	
ABI1	0.005999898	
ZNF277	0.005999898	

ZNF507	0.005999898	
THAP5	0.005999898	
SLC7A9	0.005999898	
SIX5	0.005999898	
OTUD6B	0.005999898	
MRPS31	0.005999898	
CXCL8	0.005999898	
BCL6B	0.005999898	
PHF3	0.005999898	
AIMP1	0.005999898	
ENAH	0.005999898	
KDELR1	0.005999898	
CD36	0.005999898	
PCAT7	0.005999898	
MT-RNR2	0.005999898	
ADRA2A	0.005999898	
DDX18	0.005949052	
RRP8	0.005949052	
SKA1	0.005949052	
RRN3	0.005949052	
MAFB	0.005949052	
PFKM	0.005949052	
ECHDC3	0.005949052	
WDR73	0.005949052	
VPS39	0.005949052	
CAMKK1	0.005949052	
CHMP4A	0.005949052	
TAF8	0.005949052	
ELAVL1	0.005949052	
ABHD1	0.005949052	
WDFY4	0.005949052	
TM4SF4	0.005949052	
DOHH	0.005949052	
TMEM170A	0.005949052	
TMEM184A	0.005949052	
RCN3	0.005949052	
PPL	0.005949052	
LAS1L	0.005949052	
PCDH12	0.005949052	
PLCL1	0.005949052	
SDHB	0.005949052	

CWC22	0.005949052	
PALMD	0.005949052	
BTBD7	0.005949052	
TICRR	0.005949052	
SLC44A2	0.005949052	
NUMBL	0.005949052	
YPEL1	0.005949052	
PRELP	0.005949052	
EMILIN2	0.005949052	
MRPL37	0.005949052	
KRTAP10-1	0.005949052	
NTRK2	0.005949052	
OCRL	0.005949052	
CES4A	0.005949052	
NDRG4	0.005949052	
PTAFR	0.005949052	
TIA1	0.005949052	
HLA-DPA1	0.005949052	
NLRP3	0.005949052	
RP11-42O15.3	0.005949052	
PANK2	0.005949052	
ANKFY1	0.005949052	
PJA2	0.005949052	
RNF220	0.005949052	
ZNF260	0.005949052	
PBRM1	0.005949052	
TOX4	0.005949052	
OIP5-AS1	0.005949052	
GNB5	0.005949052	
ERCC6L2	0.005949052	
PDCD1	0.005949052	
TMEM209	0.005949052	
MED11	0.005949052	
HIST1H3C	0.005949052	
BIRC3	0.005949052	
RWDD2B	0.005949052	
SPNS2	0.005949052	
GTF2H4	0.005949052	
MAGOH	0.005949052	
ST6GAL2	0.005898205	
RHOT1	0.005898205	

HIGD1A	0.005898205	
ISOC2	0.005898205	
RP11-352M15.2	0.005898205	
KCTD9	0.005898205	
OSBPL9	0.005898205	
HIST1H2BC	0.005898205	
AVP	0.005898205	
AGPAT2	0.005898205	
HLA-G	0.005898205	
L1CAM	0.005898205	
PTH1R	0.005898205	
ZBTB12	0.005898205	
PTGER4	0.005898205	
MT-ATP8	0.005898205	
SMPD4	0.005898205	
CYP19A1	0.005898205	
PRKD2	0.005898205	
PDGFRA	0.005898205	
PRAM1	0.005898205	
GYG1	0.005898205	
IFT43	0.005898205	
S100B	0.005898205	
MIS18BP1	0.005898205	
SPC24	0.005898205	
FNDC3A	0.005898205	
SERPINA12	0.005898205	
COL6A3	0.005898205	
RPP30	0.005898205	
FKBP11	0.005898205	
RIT1	0.005898205	
G6PC	0.005898205	
CD151	0.005898205	
CYP2C18	0.005898205	
MRPL15	0.005898205	
POP4	0.005898205	
MRPS11	0.005898205	
WDR83	0.005898205	
SFRP1	0.005898205	
KLHDC2	0.005898205	
PTK6	0.005898205	
PPIE	0.005898205	

SNAPC5	0.005898205	
TFPI	0.005898205	
MTMR9	0.005898205	
SOCS6	0.005898205	
SPRYD4	0.005898205	
ZNF263	0.005898205	
MTFR1L	0.005898205	
ATG4B	0.005898205	
ZBTB21	0.005898205	
TNPO2	0.005898205	
CD59	0.005898205	
SASS6	0.005898205	
LRRC40	0.005898205	
SEC23B	0.005898205	
REXO4	0.005898205	
ERF	0.005898205	
TAP1	0.005898205	
IFI35	0.005898205	
DHPS	0.005898205	
PROSER1	0.005898205	
FLAD1	0.005898205	
MSN	0.005847359	
CCR3	0.005847359	
PPP3CB	0.005847359	
CHMP1B	0.005847359	
XRCC1	0.005847359	
KLF3	0.005847359	
CGN	0.005847359	
NPR1	0.005847359	
PLEKHG2	0.005847359	
TMEM119	0.005847359	
CA2	0.005847359	
CTA-221G9.12	0.005847359	
IPO5	0.005847359	
BCAR3	0.005847359	
SLC1A5	0.005847359	
CETP	0.005847359	
SRSF5	0.005847359	
PDS5A	0.005847359	
LRSAM1	0.005847359	
XPO7	0.005847359	

RSU1	0.005847359	
DDI2	0.005847359	
GRIN2D	0.005847359	
STX6	0.005847359	
WDR45B	0.005847359	
ZNF622	0.005847359	
THAP4	0.005847359	
FBXL19	0.005847359	
DPYSL3	0.005847359	
OS9	0.005847359	
UROS	0.005847359	
DIS3	0.005847359	
PNPO	0.005847359	
HCN3	0.005847359	
LIN7A	0.005847359	
KDM6B	0.005847359	
SH2B3	0.005847359	
KANK1	0.005847359	
AGER	0.005847359	
TMEM184C	0.005847359	
ING3	0.005847359	
PROZ	0.005847359	
NCKAP1L	0.005847359	
CDK2AP2	0.005847359	
BRPF1	0.005847359	
NUBPL	0.005847359	
ARHGEF25	0.005847359	
DPCD	0.005847359	
TEX30	0.005847359	
RP11-394O4.5	0.005847359	
43526	0.005847359	
USP16	0.005847359	
PADI3	0.005847359	
IARS	0.005847359	
PARP4	0.005847359	
CHSY1	0.005847359	
LGALS3BP	0.005847359	
ECM2	0.005847359	
DNMBP	0.005847359	
BASP1	0.005847359	
POLR1D	0.005847359	

PDAP1	0.005847359	
MORN4	0.005847359	
LLPH	0.005847359	
TTC7A	0.005847359	
MBP	0.005847359	
PRG4	0.005847359	
MEGF8	0.005847359	
SYF2	0.005847359	
ZNF687	0.005847359	
TANK	0.005847359	
SH3RF1	0.005847359	
LMNB2	0.005847359	
DCLK2	0.005847359	
HOXD9	0.005847359	
DKC1	0.005796512	
NUDT5	0.005796512	
AJUBA	0.005796512	
SRP14	0.005796512	
SUGP1	0.005796512	
FDFT1	0.005796512	
PACSIN2	0.005796512	
ZNF385A	0.005796512	
ARFRP1	0.005796512	
GEMIN6	0.005796512	
TGFBR3	0.005796512	
NFRKB	0.005796512	
43532	0.005796512	
AP3D1	0.005796512	
ING2	0.005796512	
C19orf66	0.005796512	
IDH3B	0.005796512	
COA3	0.005796512	
CCNE2	0.005796512	
MRPL45	0.005796512	
IMPDH1	0.005796512	
ATP13A3	0.005796512	
SSBP3	0.005796512	
SPECC1	0.005796512	
SIM2	0.005796512	
SCYL2	0.005796512	
CLDN7	0.005796512	

ATAD3A	0.005796512	
RP11-116D2.1	0.005796512	
SLC5A6	0.005796512	
BCCIP	0.005796512	
HTRA2	0.005796512	
SPRED1	0.005796512	
NME7	0.005796512	
QPRT	0.005796512	
MPHOSPH6	0.005796512	
TMEM171	0.005796512	
PDGFB	0.005796512	
DLEU1	0.005796512	
CD72	0.005796512	
DDX27	0.005796512	
SNRK	0.005796512	
GPBAR1	0.005796512	
CAPZB	0.005796512	
NUGGC	0.005796512	
ACAN	0.005796512	
SIK2	0.005796512	
C1orf109	0.005796512	
RIPPLY1	0.005796512	
TMEM50A	0.005796512	
C9orf64	0.005796512	
ZDHHC12	0.005796512	
VPS25	0.005796512	
HVCN1	0.005796512	
CEPT1	0.005796512	
C6orf132	0.005796512	
ZNF219	0.005796512	
CCDC186	0.005796512	
IFITM2	0.005796512	
SPAG7	0.005796512	
TOMM22	0.005796512	
SMPDL3A	0.005796512	
KDM2A	0.005745665	
NTM	0.005745665	
DHX35	0.005745665	
PCNX	0.005745665	
ARHGEF6	0.005745665	
P2RY6	0.005745665	

CCM2	0.005745665	
IPCEF1	0.005745665	
GFPT2	0.005745665	
STAC3	0.005745665	
CEP44	0.005745665	
CA5A	0.005745665	
RP11-573D15.8	0.005745665	
AC000123.2	0.005745665	
ZNF70	0.005745665	
VANGL1	0.005745665	
ZCRB1	0.005745665	
N4BP2	0.005745665	
CHCHD3	0.005745665	
HEXDC	0.005745665	
MBL2	0.005745665	
RASA2	0.005745665	
QRICH1	0.005745665	
NPAS2	0.005745665	
FAM105A	0.005745665	
SLC34A2	0.005745665	
OAT	0.005745665	
ZFPL1	0.005745665	
BHLHA15	0.005745665	
CNN1	0.005745665	
ANKS3	0.005745665	
ARHGAP23	0.005745665	
ZNF775	0.005745665	
HECTD4	0.005745665	
RANGAP1	0.005745665	
SCAI	0.005745665	
UBAP2L	0.005745665	
HOMER2	0.005745665	
MICAL1	0.005745665	
TOMM20	0.005745665	
GGT5	0.005745665	
FTLP3	0.005745665	
EIF1	0.005745665	
UBE2J1	0.005745665	
SERPINA11	0.005745665	
RBAK	0.005745665	
ISCU	0.005745665	

IFI27L2	0.005745665	
C11orf84	0.005745665	
LIPE	0.005745665	
LIPG	0.005745665	
USP21	0.005745665	
SAA4	0.005745665	
PEX11G	0.005745665	
NPC1	0.005745665	
PTP4A1	0.005745665	
RAD51D	0.005745665	
RNF14	0.005745665	
ILF3-AS1	0.005745665	
SLC17A3	0.005745665	
CHST13	0.005745665	
TXNDC16	0.005745665	
WBP5	0.005745665	
RASGEF1B	0.005745665	
EDEM3	0.005694819	
GCLM	0.005694819	
ALDH1B1	0.005694819	
CENPU	0.005694819	
TDP2	0.005694819	
EHD1	0.005694819	
C16orf89	0.005694819	
RAD17	0.005694819	
CCDC80	0.005694819	
LYPLA2	0.005694819	
TFEC	0.005694819	
RPL13AP5	0.005694819	
ANKRD52	0.005694819	
VNN1	0.005694819	
CTD-3098H1.2	0.005694819	
TMEM186	0.005694819	
HIST1H2AC	0.005694819	
PELI1	0.005694819	
TTF1	0.005694819	
USP54	0.005694819	
TRIP12	0.005694819	
RP11-622A1.2	0.005694819	
FNDC5	0.005694819	
DUSP2	0.005694819	

FTLP2	0.005694819	
PPARGC1B	0.005694819	
PCMT1	0.005694819	
KCTD17	0.005694819	
CHRNB2	0.005694819	
IGLL1	0.005694819	
TEX264	0.005694819	
NEDD1	0.005694819	
SPATA2L	0.005694819	
CXCR3	0.005694819	
ABCB8	0.005694819	
PLEKHG1	0.005694819	
DYRK1B	0.005694819	
FRMD6	0.005694819	
TRIM16	0.005694819	
RNF44	0.005694819	
RP11-115J16.1	0.005694819	
CTNNBIP1	0.005694819	
PDE4B	0.005694819	
NKX2-5	0.005694819	
CD9	0.005694819	
CNTN4	0.005694819	
DTNA	0.005694819	
BRD4	0.005694819	
KIAA0226L	0.005643972	
THBD	0.005643972	
BPGM	0.005643972	
LRRC1	0.005643972	
NIPSNAP1	0.005643972	
CRTC2	0.005643972	
TMED2	0.005643972	
GNA11	0.005643972	
ZNF471	0.005643972	
DDX60	0.005643972	
COG3	0.005643972	
INHBE	0.005643972	
CDHR5	0.005643972	
HSPA1L	0.005643972	
PKD2	0.005643972	
STRN3	0.005643972	
BAG5	0.005643972	

UQCC2	0.005643972	
ACADVL	0.005643972	
CCNB2	0.005643972	
STAB1	0.005643972	
KCTD10	0.005643972	
GAS1	0.005643972	
EHBP1	0.005643972	
APCS	0.005643972	
RP11-513G11.3	0.005643972	
ATF7IP2	0.005643972	
IGLC2	0.005643972	
CLDN2	0.005643972	
TNFRSF19	0.005643972	
GJA4	0.005643972	
RAB7A	0.005643972	
ATP11B	0.005643972	
ZNF589	0.005643972	
SLC25A34	0.005643972	
SLU7	0.005643972	
DHX58	0.005643972	
ZSWIM4	0.005643972	
ADAM12	0.005643972	
INS	0.005643972	
GK	0.005643972	
TM4SF1	0.005643972	
DNAJB1	0.005643972	
RP11-46C24.7	0.005643972	
EHD2	0.005643972	
PHF5A	0.005643972	
LINGO1	0.005643972	
CNST	0.005643972	
ARL5B	0.005643972	
MCM9	0.005643972	
PDLIM1	0.005643972	
MAPKAPK5	0.005643972	
SORBS1	0.005643972	
PLD2	0.005643972	
BMP1	0.005643972	
TDGF1	0.005643972	
IK	0.005643972	
ERCC6	0.005643972	

SCARF2	0.005643972	
DLL1	0.005643972	
SYMPK	0.005643972	
PHF23	0.005593126	
PHIP	0.005593126	
GSTZ1	0.005593126	
EIF2B3	0.005593126	
ARHGEF18	0.005593126	
ACTR8	0.005593126	
CDPF1	0.005593126	
BCL2L11	0.005593126	
FILIP1	0.005593126	
TWF1	0.005593126	
GRN	0.005593126	
MYO1G	0.005593126	
APOBEC3G	0.005593126	
MAPK8IP2	0.005593126	
MAOA	0.005593126	
LURAP1L	0.005593126	
HADHA	0.005593126	
RABEP2	0.005593126	
NAPA	0.005593126	
NEK4	0.005593126	
RAD54L2	0.005593126	
FHL5	0.005593126	
SEC24A	0.005593126	
TCEANC	0.005593126	
PHLDB1	0.005593126	
RRP1	0.005593126	
ST6GALNAC4	0.005593126	
DNAJC24	0.005593126	
TTC33	0.005593126	
DCAF6	0.005593126	
STK38L	0.005593126	
CA9	0.005593126	
FBLN5	0.005593126	
CLK1	0.005593126	
CDK11B	0.005593126	
COL16A1	0.005593126	
PVRL3	0.005593126	
SAA2	0.005593126	

COMMD6	0.005593126	
CHM	0.005593126	
CTD-2114J12.1	0.005593126	
ZNF532	0.005593126	
IGHG2	0.005593126	
LGALS4	0.005593126	
UGT1A3	0.005593126	
KRT10	0.005593126	
APOLD1	0.005593126	
LRRC4B	0.005593126	
TNFRSF10B	0.005593126	
FGD3	0.005593126	
MRPS7	0.005593126	
VAMP2	0.005593126	
CNDP1	0.005593126	
LTF	0.005593126	
NEDD4L	0.005593126	
GLRX2	0.005593126	
BHLHE41	0.005593126	
RORB	0.005593126	
B9D1	0.005593126	
RAD51C	0.005593126	
CLEC3B	0.005593126	
LINC01355	0.005593126	
RANBP3L	0.005593126	
SMUG1	0.005593126	
MKRN3	0.005593126	
UBR2	0.005593126	
CTD-2256P15.2	0.005593126	
SERPING1	0.005593126	
C5orf42	0.005593126	
RP11-401P9.4	0.005593126	
OSBPL5	0.005593126	
OSBP	0.005593126	
POLL	0.005593126	
JAM2	0.005593126	
RNASE6	0.005593126	
HLA-E	0.005593126	
TMEM135	0.005542279	
DENND5B	0.005542279	
CD97	0.005542279	

MRPL34	0.005542279	
ARSA	0.005542279	
ARF5	0.005542279	
ELF2	0.005542279	
ZNF518A	0.005542279	
LYRM5	0.005542279	
RPP40	0.005542279	
IL10RA	0.005542279	
GBGT1	0.005542279	
CPT1B	0.005542279	
VTI1B	0.005542279	
COL6A1	0.005542279	
PTRHD1	0.005542279	
VAPB	0.005542279	
MTRNR2L12	0.005542279	
CCDC23	0.005542279	
PDE7B	0.005542279	
LDHA	0.005542279	
ARHGEF10L	0.005542279	
ANXA4	0.005542279	
CD40LG	0.005542279	
RASSF7	0.005542279	
PDCD6	0.005542279	
ISYNA1	0.005542279	
RALBP1	0.005542279	
CYP2C9	0.005542279	
MAP3K6	0.005542279	
IPO7	0.005542279	
CAMLG	0.005542279	
PFKL	0.005542279	
GNPAT	0.005542279	
RAD51B	0.005542279	
UCHL3	0.005542279	
SASH1	0.005542279	
LBH	0.005542279	
KIAA0355	0.005542279	
GAD1	0.005542279	
DNAJB11	0.005542279	
CD320	0.005542279	
PILRA	0.005542279	
B4GALT3	0.005542279	

RP11-209K10.2	0.005542279	
POLD2	0.005542279	
NCKAP1	0.005542279	
MMS19	0.005542279	
TLR3	0.005542279	
VPS36	0.005542279	
LTB	0.005542279	
ITPR2	0.005542279	
WEE1	0.005542279	
LY9	0.005542279	
DDX1	0.005542279	
OTUD5	0.005542279	
TACO1	0.005542279	
ATG16L2	0.005542279	
ZSCAN16-AS1	0.005542279	
GUCY1B3	0.005542279	
MPHOSPH9	0.005542279	
LRRC8C	0.005542279	
SMC5	0.005542279	
ASPH	0.005542279	
PLIN2	0.005542279	
CCDC146	0.005542279	
IL4I1	0.005491432	
FOXA3	0.005491432	
GSN	0.005491432	
GMEB1	0.005491432	
AIF1	0.005491432	
TSGA10	0.005491432	
LAT2	0.005491432	
HSCB	0.005491432	
TRAC	0.005491432	
GNA14	0.005491432	
MMP7	0.005491432	
JAG2	0.005491432	
PARVB	0.005491432	
ZNF438	0.005491432	
C6orf106	0.005491432	
CD52	0.005491432	
DBF4	0.005491432	
TMEM79	0.005491432	
C11orf54	0.005491432	

AC018738.2	0.005491432	
PIGV	0.005491432	
CEP250	0.005491432	
WDR86	0.005491432	
AUTS2	0.005491432	
RPS6KC1	0.005491432	
CLDN14	0.005491432	
TPM1	0.005491432	
MAD2L1BP	0.005491432	
TCFL5	0.005491432	
SENP2	0.005491432	
RPP25	0.005491432	
FAM13B	0.005491432	
ANKRD40	0.005491432	
ACOT12	0.005491432	
ACTR3B	0.005491432	
C5AR1	0.005491432	
VRK2	0.005491432	
GLB1	0.005491432	
CCDC90B	0.005491432	
YDJC	0.005491432	
ZBTB39	0.005491432	
NEUROD1	0.005491432	
SLC27A2	0.005491432	
REEP5	0.005491432	
N4BP2L1	0.005491432	
NOP56	0.005491432	
UBR4	0.005491432	
DUS2	0.005491432	
LCN2	0.005491432	
GPC3	0.005491432	
ZNF511	0.005491432	
SPDL1	0.005491432	
FTH1P7	0.005491432	
PTPN3	0.005491432	
GPR161	0.005491432	
PTOV1	0.005491432	
CYP4F3	0.005491432	
CA12	0.005491432	
AKAP8L	0.005491432	
COL8A2	0.005491432	

FRA10AC1	0.005491432	
VPS37C	0.005491432	
SRP9	0.005491432	
CDC26	0.005491432	
NKIRAS1	0.005491432	
GAS2L1	0.005491432	
ARHGEF17	0.005491432	
ODF2L	0.005491432	
KCTD6	0.005491432	
SNRPN	0.005491432	
CCT8	0.005491432	
TBC1D22A	0.005491432	
ADSL	0.005491432	
LYRM4	0.005440586	
LAMTOR3	0.005440586	
NOD1	0.005440586	
RAB4A	0.005440586	
PDGFA	0.005440586	
RPP14	0.005440586	
TBX2	0.005440586	
CCDC159	0.005440586	
DACT1	0.005440586	
ASF1A	0.005440586	
CAPG	0.005440586	
GOLGA1	0.005440586	
MCAM	0.005440586	
GZMA	0.005440586	
ATP5G3	0.005440586	
PPM1G	0.005440586	
ERBB4	0.005440586	
ZMYND19	0.005440586	
FIZ1	0.005440586	
TIMM17B	0.005440586	
PRDM10	0.005440586	
NOL12	0.005440586	
EIF4B	0.005440586	
PRPS2	0.005440586	
UNC13D	0.005440586	
LOX	0.005440586	
LRRC20	0.005440586	
ASB6	0.005440586	

HSD17B2	0.005440586	
HLA-DOA	0.005440586	
RP13-20L14.6	0.005440586	
OSBPL11	0.005440586	
SVEP1	0.005440586	
PDE3B	0.005440586	
MSANTD2	0.005440586	
SPG21	0.005440586	
PLRG1	0.005440586	
SHROOM3	0.005440586	
RRP12	0.005440586	
DENND3	0.005440586	
KRAS	0.005440586	
ATG3	0.005440586	
TNRC18	0.005440586	
LUC7L	0.005440586	
FBXO7	0.005440586	
DERL1	0.005440586	
UGT2B17	0.005440586	
EYA3	0.005440586	
NLRC4	0.005440586	
SNRNP40	0.005440586	
DNAJC30	0.005440586	
IRF2BP1	0.005440586	
SRP72	0.005440586	
PON3	0.005440586	
SLCO1B1	0.005440586	
ARR3	0.005440586	
AFMID	0.005440586	
POLR3C	0.005440586	
TINAGL1	0.005440586	
RNF128	0.005389739	
RP11-758H9.2	0.005389739	
HRG	0.005389739	
CLIP2	0.005389739	
GUCY2D	0.005389739	
SUDS3	0.005389739	
NUP107	0.005389739	
SDHAF1	0.005389739	
KANK2	0.005389739	
FAF2	0.005389739	

GPI	0.005389739	
GSS	0.005389739	
MRPS18A	0.005389739	
KLF15	0.005389739	
GSTO2	0.005389739	
STRA13	0.005389739	
NAT8	0.005389739	
ADAM15	0.005389739	
ARHGAP25	0.005389739	
MAP2K4	0.005389739	
NF2	0.005389739	
DOCK9	0.005389739	
UBE2R2	0.005389739	
PYGM	0.005389739	
NDUFA10	0.005389739	
REN	0.005389739	
ZNF45	0.005389739	
HOXD10	0.005389739	
CBR3	0.005389739	
CYP4V2	0.005389739	
FTCD	0.005389739	
BACE1-AS	0.005389739	
PAPSS2	0.005389739	
LINC01151	0.005389739	
TAF1C	0.005389739	
PEA15	0.005389739	
GOLPH3	0.005389739	
SMIM6	0.005389739	
DACH1	0.005389739	
SNAPC1	0.005389739	
HSD17B7	0.005389739	
ZNF469	0.005389739	
MRPS26	0.005389739	
TOR1AIP1	0.005389739	
EEF1B2P3	0.005389739	
CLPB	0.005389739	
USP3	0.005389739	
XPO4	0.005389739	
TLX3	0.005389739	
SNX1	0.005389739	
BRMS1L	0.005389739	

ATP6V1H	0.005389739	
NGFRAP1	0.005389739	
COPG2	0.005389739	
PRODH2	0.005389739	
ADAMTS12	0.005389739	
IYD	0.005389739	
ARHGAP22	0.005389739	
FCHO1	0.005389739	
IL33	0.005389739	
SHMT2	0.005389739	
THOC5	0.005389739	
PEX11A	0.005389739	
PDZD3	0.005389739	
AKTIP	0.005389739	
NELFE	0.005389739	
SLC25A10	0.005389739	
KLB	0.005389739	
OGFOD1	0.005389739	
B4GALT1-AS1	0.005389739	
FKBP9	0.005389739	
IL5RA	0.005389739	
UBXN11	0.005389739	
AKR1A1	0.005389739	
GPHN	0.005389739	
CBX2	0.005389739	
ESAM	0.005389739	
LRRC16A	0.005338893	
RAD52	0.005338893	
TSR1	0.005338893	
TCEB3B	0.005338893	
GNB4	0.005338893	
GPR128	0.005338893	
LRG1	0.005338893	
JRK	0.005338893	
CHMP4C	0.005338893	
IDI1	0.005338893	
NRL	0.005338893	
TNFSF10	0.005338893	
HERC5	0.005338893	
MARCKS	0.005338893	
GDI2	0.005338893	

HS3ST1	0.005338893	
ATP2C1	0.005338893	
S100A8	0.005338893	
IFNAR2	0.005338893	
PPIP5K2	0.005338893	
TMEM101	0.005338893	
AKAP8	0.005338893	
CDK13	0.005338893	
ARAP3	0.005338893	
SLC35C2	0.005338893	
MPL	0.005338893	
FSCN1	0.005338893	
PKIB	0.005338893	
TRMT2A	0.005338893	
NMNAT2	0.005338893	
SERPINA6	0.005338893	
PAF1	0.005338893	
ZNF451	0.005338893	
NBPF13P	0.005338893	
DSTYK	0.005338893	
MAN2A1	0.005338893	
FAM69B	0.005338893	
SLA2	0.005338893	
B4GALT2	0.005338893	
MSRB3	0.005338893	
TATDN1	0.005338893	
KIAA1468	0.005338893	
CD84	0.005338893	
AC005355.2	0.005338893	
ARMC5	0.005338893	
NIT1	0.005338893	
SPIN4	0.005338893	
TRIM69	0.005338893	
PIF1	0.005338893	
ZDHHC9	0.005338893	
SLC25A1	0.005338893	
POP7	0.005338893	
SLC10A1	0.005338893	
STK35	0.005338893	
TTC38	0.005338893	
NUCB1	0.005338893	

PPIL1	0.005338893	
CAD	0.005338893	
HIST1H3B	0.005338893	
TRIM41	0.005338893	
LRRC42	0.005338893	
SELE	0.005338893	
LOXL3	0.005338893	
DERL2	0.005338893	
ZHX3	0.005288046	
HLCS	0.005288046	
RP11-250B2.6	0.005288046	
WDTC1	0.005288046	
AEN	0.005288046	
NAGA	0.005288046	
ANKIB1	0.005288046	
EIF2B1	0.005288046	
MRPL50	0.005288046	
CYB5D1	0.005288046	
ZBED1	0.005288046	
C17orf62	0.005288046	
CENPO	0.005288046	
ZER1	0.005288046	
TECRP1	0.005288046	
CD33	0.005288046	
ADM2	0.005288046	
TIPRL	0.005288046	
ARF3	0.005288046	
TUBGCP2	0.005288046	
WDR25	0.005288046	
PDK1	0.005288046	
CEP57	0.005288046	
RBM41	0.005288046	
SAMD4B	0.005288046	
TNFRSF10D	0.005288046	
CYP1A2	0.005288046	
RP11-390E23.6	0.005288046	
MACROD1	0.005288046	
RGS20	0.005288046	
LARS	0.005288046	
HSD17B14	0.005288046	
SOX17	0.005288046	

CHAD	0.005288046	
UNC119B	0.005288046	
JAGN1	0.005288046	
CHI3L1	0.005288046	
SPRY1	0.005288046	
SP110	0.005288046	
CD300A	0.005288046	
AC108078.1	0.005288046	
ZNF7	0.005288046	
IRGQ	0.005288046	
METRNL	0.005288046	
C18orf32	0.005288046	
TPD52	0.005288046	
PDE7A	0.005288046	
TRIM16L	0.005288046	
PEX6	0.005288046	
STK11IP	0.005288046	
RP5-966M1.6	0.005288046	
SIAH2	0.005288046	
GIMAP8	0.005288046	
ZWILCH	0.005288046	
HOXB3	0.005288046	
MRPL49	0.005288046	
SUMO1P1	0.005288046	
BCL11B	0.005288046	
RDH16	0.005288046	
SLC9B2	0.005288046	
KPNA4	0.005288046	
NT5M	0.005288046	
SUCO	0.005288046	
ORC3	0.005288046	
SCYL3	0.005288046	
NANOG	0.005237199	
IGFBP2	0.005237199	
CLP1	0.005237199	
PCSK7	0.005237199	
EXOC8	0.005237199	
POLR3A	0.005237199	
TRIM42	0.005237199	
ABCG8	0.005237199	
RABGGTB	0.005237199	

WHSC1L1	0.005237199	
PALB2	0.005237199	
HNRNPA3	0.005237199	
MTMR2	0.005237199	
TBC1D17	0.005237199	
GSTM2	0.005237199	
GTPBP4	0.005237199	
CMPK2	0.005237199	
MINOS1	0.005237199	
SCML2	0.005237199	
DUSP23	0.005237199	
STX3	0.005237199	
RP11-278L15.6	0.005237199	
PPP2R5C	0.005237199	
SWSAP1	0.005237199	
MRPL1	0.005237199	
DTNB	0.005237199	
OXCT2	0.005237199	
ATP6V0C	0.005237199	
SULT1A1	0.005237199	
NAT9	0.005237199	
SGSM3	0.005237199	
RIC8B	0.005237199	
TTC21B	0.005237199	
RNMTL1	0.005237199	
PRSS1	0.005237199	
PCGF1	0.005237199	
ZNF502	0.005237199	
EDN1	0.005237199	
SEC11C	0.005237199	
WNT4	0.005237199	
ZNF326	0.005237199	
UGGT1	0.005237199	
MGAT1	0.005237199	
PYGO2	0.005237199	
TBCA	0.005237199	
HIST2H3C	0.005237199	
HIATL1	0.005237199	
NPHP3	0.005237199	
TAPBP	0.005237199	
ELOVL1	0.005237199	

RASAL3	0.005237199	
FIP1L1	0.005237199	
HSPB11	0.005237199	
HCG18	0.005237199	
WBSCR22	0.005237199	
CAPN2	0.005237199	
EIF4G3	0.005237199	
ZNF790	0.005237199	
GLDC	0.005237199	
ZBTB8A	0.005237199	
PPA1	0.005237199	
CDK5R1	0.005237199	
ESCO2	0.005237199	
TIGD5	0.005237199	
EPCAM	0.005237199	
CACNA2D1	0.005186353	
SPCS2	0.005186353	
SIK3	0.005186353	
FJX1	0.005186353	
PPP2R3A	0.005186353	
ELFN1	0.005186353	
SSH1	0.005186353	
ZNF767P	0.005186353	
TUBB1	0.005186353	
KIF1B	0.005186353	
DDAH2	0.005186353	
OSBPL10	0.005186353	
TSFM	0.005186353	
RP5-857K21.6	0.005186353	
GMPPB	0.005186353	
PSD3	0.005186353	
TOPORS-AS1	0.005186353	
LIMS2	0.005186353	
SMG5	0.005186353	
RP11-234A1.1	0.005186353	
PRRG4	0.005186353	
MAP3K11	0.005186353	
FRS3	0.005186353	
AMER1	0.005186353	
DCTN2	0.005186353	
VCAM1	0.005186353	

SRPX	0.005186353	
ERN1	0.005186353	
USP2	0.005186353	
LPA	0.005186353	
VAT1	0.005186353	
TTI2	0.005186353	
HKDC1	0.005186353	
FHIT	0.005186353	
TXNDC9	0.005186353	
HUS1	0.005186353	
CPEB2	0.005186353	
MFAP4	0.005186353	
RAB2B	0.005186353	
BX842568.2	0.005186353	
SMIM19	0.005186353	
NUTM2A-AS1	0.005186353	
CCDC59	0.005186353	
RBM6	0.005186353	
C5orf22	0.005186353	
COL18A1	0.005186353	
MCF2L	0.005186353	
ARL4D	0.005186353	
USP53	0.005186353	
TNFSF12	0.005186353	
BRIX1	0.005186353	
SAA2-SAA4	0.005186353	
YEATS2	0.005186353	
CEP135	0.005186353	
GSR	0.005186353	
RYK	0.005186353	
GALNS	0.005186353	
EIF3J	0.005186353	
ADAP1	0.005186353	
ICE2	0.005186353	
PTMAP5	0.005186353	
DIABLO	0.005186353	
UNC13B	0.005186353	
MMP11	0.005186353	
KLHDC3	0.005186353	
HOXB6	0.005186353	
HIST1H3D	0.005186353	

UBE2J2	0.005186353	
SH3BP1	0.005186353	
PPAPDC2	0.005186353	
SMYD5	0.005186353	
PMM1	0.005186353	
WDR24	0.005186353	
LINC00116	0.005186353	
RP11-35N6.6	0.005186353	
REEP6	0.005186353	
ODC1	0.005186353	
CTAGE5	0.005186353	
IFNAR1	0.005186353	
OAS2	0.005186353	
HP1BP3	0.005186353	
CMKLR1	0.005186353	
CFDP1	0.005186353	
SSPN	0.005186353	
UBALD2	0.005186353	
RP11-147L13.11	0.005186353	
DDIAS	0.005186353	
FZR1	0.005186353	
FAM110A	0.005135506	
ACAD10	0.005135506	
DCAF11	0.005135506	
RHOD	0.005135506	
ETV7	0.005135506	
ASPG	0.005135506	
C3AR1	0.005135506	
PATL1	0.005135506	
UBA7	0.005135506	
ARHGEF4	0.005135506	
UBA2	0.005135506	
AHCYL1	0.005135506	
DOCK8	0.005135506	
DEPTOR	0.005135506	
TPMT	0.005135506	
TMC4	0.005135506	
MYO10	0.005135506	
MYH11	0.005135506	
KLF16	0.005135506	
GPX8	0.005135506	

ZNF107	0.005135506	
HIST1H1C	0.005135506	
ABCE1	0.005135506	
PRR11	0.005135506	
RNF20	0.005135506	
EPHA2	0.005135506	
COX4I2	0.005135506	
CYP4A22	0.005135506	
COQ5	0.005135506	
RP11-404G16.2	0.005135506	
RLTPR	0.005135506	
PHF10	0.005135506	
TMEM39B	0.005135506	
C19orf57	0.005135506	
B4GALT1	0.005135506	
EEF1A1P6	0.005135506	
RMND5B	0.005135506	
CLSTN1	0.005135506	
ACOT13	0.005135506	
OCEL1	0.005135506	
OGDH	0.005135506	
PSME4	0.005135506	
CARHSP1	0.005135506	
BUD13	0.005135506	
CARD11	0.005135506	
SLC35F5	0.005135506	
PLEK	0.005135506	
ROBO4	0.005135506	
OSCAR	0.005135506	
VPS37B	0.005135506	
APITD1	0.005135506	
MSH3	0.005135506	
TPI1	0.005135506	
CYP2A7	0.005135506	
ACTR6	0.005135506	
SOCS5	0.005135506	
RHBG	0.005135506	
KPNA6	0.005135506	
TM9SF2	0.005135506	
TMEM203	0.005135506	
CDC42EP2	0.005135506	

RRAGC	0.005135506	
INTS3	0.00508466	
ANP32B	0.00508466	
GRAMD1A	0.00508466	
PRPF39	0.00508466	
SNX9	0.00508466	
H1F0	0.00508466	
GOLIM4	0.00508466	
MRPS24	0.00508466	
JPH2	0.00508466	
PODN	0.00508466	
ZMYND15	0.00508466	
FAM175A	0.00508466	
DPEP2	0.00508466	
CCDC136	0.00508466	
HIST1H4I	0.00508466	
YBX3	0.00508466	
SERINC1	0.00508466	
DEPDC1B	0.00508466	
SEC31A	0.00508466	
PUS7L	0.00508466	
GFOD1	0.00508466	
PPIL2	0.00508466	
VIPAS39	0.00508466	
PECR	0.00508466	
CST7	0.00508466	
CENPC	0.00508466	
SNCG	0.00508466	
CYP2D7	0.00508466	
TRMT12	0.00508466	
IFITM3	0.00508466	
SENP3	0.00508466	
FYB	0.00508466	
YY1AP1	0.00508466	
SUN2	0.00508466	
PCNXL4	0.00508466	
PSAP	0.00508466	
EXOC4	0.00508466	
FPGT	0.00508466	
PLAGL1	0.00508466	
HDGF	0.00508466	

GNG10	0.00508466	
SELP	0.00508466	
PPP1R8	0.00508466	
FADS2	0.00508466	
PSMB10	0.00508466	
PDE10A	0.00508466	
PPP1R9A	0.00508466	
TLR2	0.00508466	
SLC35B3	0.00508466	
TRIM63	0.00508466	
CDC42EP1	0.00508466	
EIF4EBP1	0.00508466	
TIMM17A	0.00508466	
SPTBN2	0.00508466	
FZD4	0.00508466	
YME1L1	0.00508466	
DDX39A	0.00508466	
DIO1	0.00508466	
BRI3	0.00508466	
MTIF3	0.00508466	
UBE4B	0.00508466	
LNX2	0.00508466	
TNKS1BP1	0.00508466	
ST8SIA4	0.00508466	
PREB	0.00508466	
HMBOX1	0.00508466	
BZW1	0.00508466	
METTL13	0.00508466	
EDNRA	0.00508466	
CELF1	0.00508466	
ABL2	0.00508466	
REC8	0.00508466	
RHOBTB2	0.00508466	
NIPAL1	0.00508466	
ABCC3	0.00508466	
POU6F1	0.00508466	
RP5-1033H22.2	0.005033813	
ANXA5	0.005033813	
GNE	0.005033813	
SERPINA7	0.005033813	
SMIM7	0.005033813	

FAM210B	0.005033813	
HTRA1	0.005033813	
CHCHD10	0.005033813	
VAV2	0.005033813	
LEAP2	0.005033813	
KAT8	0.005033813	
NOA1	0.005033813	
CSDE1	0.005033813	
RRP15	0.005033813	
EEF1A1P13	0.005033813	
VGLL3	0.005033813	
SLC25A12	0.005033813	
NTF3	0.005033813	
RFNG	0.005033813	
RAB11FIP5	0.005033813	
RHBDD2	0.005033813	
ETNK1	0.005033813	
TXLNG	0.005033813	
ACOT4	0.005033813	
SCARF1	0.005033813	
HGF	0.005033813	
TELO2	0.005033813	
MVD	0.005033813	
MPZ	0.005033813	
TBC1D13	0.005033813	
ZBTB32	0.005033813	
AKT1S1	0.005033813	
MGP	0.005033813	
PCAT19	0.005033813	
MLANA	0.005033813	
KIF3C	0.005033813	
TNFRSF4	0.005033813	
PHKG2	0.005033813	
MYLIP	0.005033813	
PEX10	0.005033813	
TNFRSF12A	0.005033813	
SGPL1	0.005033813	
YIPF4	0.005033813	
LINC00863	0.005033813	
UFL1	0.005033813	
FNDC1	0.005033813	

PRPF18	0.005033813	
MTA3	0.005033813	
CTSZ	0.005033813	
SFXN3	0.005033813	
MAPKAPK5-AS1	0.005033813	
ATP6V1G1	0.005033813	
FOXS1	0.005033813	
DCAF12	0.005033813	
ADRA1A	0.005033813	
EIF4A1	0.005033813	
BTBD10	0.005033813	
HSPA6	0.005033813	
LBR	0.005033813	
TTC36	0.005033813	
NFAM1	0.005033813	
SNX4	0.005033813	
ADCK3	0.005033813	
SDCCAG3	0.005033813	
PACSIN3	0.005033813	
ANKS4B	0.005033813	
MRPL30	0.005033813	
MST1R	0.005033813	
MT-ND2	0.005033813	
TBCEL	0.005033813	
TPRG1L	0.005033813	
SERPINA3	0.005033813	
NCBP2	0.005033813	
FBXW8	0.004982966	
THAP8	0.004982966	
RP5-827C21.4	0.004982966	
PCGF5	0.004982966	
CLIP1	0.004982966	
IL27RA	0.004982966	
PTK7	0.004982966	
ZADH2	0.004982966	
TNNI1	0.004982966	
PPP2R4	0.004982966	
DBNDD1	0.004982966	
TRUB2	0.004982966	
CABLES1	0.004982966	
RAB3A	0.004982966	

STEAP3	0.004982966	
BCYRN1	0.004982966	
NUDT7	0.004982966	
ZNF561	0.004982966	
PDE6G	0.004982966	
RP2	0.004982966	
MITD1	0.004982966	
EMP1	0.004982966	
EFNB1	0.004982966	
GTF2IRD1	0.004982966	
CASC5	0.004982966	
RMND1	0.004982966	
FMNL2	0.004982966	
FAM20B	0.004982966	
HERPUD1	0.004982966	
FDPS	0.004982966	
CDK2AP1	0.004982966	
RP1-60O19.1	0.004982966	
CERK	0.004982966	
TTLL3	0.004982966	
LPCAT4	0.004982966	
ZSCAN21	0.004982966	
GCSH	0.004982966	
ERCC5	0.004982966	
SLC43A1	0.004982966	
YIPF1	0.004982966	
DYNLL2	0.004982966	
ECE1	0.004982966	
TRMT6	0.004982966	
CTC-251I16.1	0.004982966	
C1orf86	0.004982966	
TRPC1	0.004982966	
CDK5RAP2	0.004982966	
ADI1	0.004982966	
MGST2	0.004982966	
RAPGEF5	0.004982966	
ENSA	0.004982966	
FAM83D	0.004982966	
PHPT1	0.004982966	
VPS26A	0.004982966	
ALKBH2	0.004982966	

CD8B	0.004982966	
ARFGAP2	0.004982966	
CD7	0.004982966	
SCIMP	0.004982966	
PRRX1	0.004982966	
GLI4	0.004982966	
TUBB2A	0.004982966	
GLT1D1	0.004982966	
ZNF280C	0.004982966	
RARRES3	0.004982966	
ATP11A	0.004982966	
EPB41L2	0.004982966	
MICALL2	0.004982966	
ILVBL	0.004982966	
LDLRAD3	0.004982966	
FXR1	0.004982966	
CAST	0.004982966	
RGS16	0.004982966	
VPS53	0.004982966	
ZNF621	0.004982966	
EIF4ENIF1	0.004982966	
INTS10	0.004982966	
ADCY7	0.004982966	
IDH1	0.004982966	
ACP1	0.004982966	
SH3BP5	0.004982966	
SMYD3	0.004982966	
LPHN1	0.004982966	
LONP1	0.004982966	
WDR81	0.004982966	
IGJ	0.004982966	
EIF2AK3	0.004982966	
TMEM126A	0.004982966	
CDADC1	0.004982966	
POLR2F	0.004982966	
ITIH5	0.004982966	
SYVN1	0.004982966	
DHTKD1	0.004982966	
PSIP1	0.004982966	
BDNF	0.004982966	
FAM193A	0.004982966	

CENPQ	0.004982966	
UFC1	0.004982966	
PPP1R3E	0.004982966	
AKR1D1	0.004982966	
GP6	0.004982966	
WASH2P	0.004982966	
COMTD1	0.004982966	
CCDC163P	0.004982966	
SLC25A25	0.004982966	
SLC4A1AP	0.00493212	
VOPP1	0.00493212	
ZNF436	0.00493212	
PPP1R1C	0.00493212	
YTHDF2	0.00493212	
SLC16A2	0.00493212	
EXOC3	0.00493212	
ADH5	0.00493212	
SLC43A3	0.00493212	
RP11-67L2.2	0.00493212	
C16orf86	0.00493212	
BLOC1S2	0.00493212	
BOD1	0.00493212	
EBLN3	0.00493212	
ST3GAL2	0.00493212	
ACOT7	0.00493212	
COMT	0.00493212	
SSU72	0.00493212	
CDC42EP5	0.00493212	
S1PR4	0.00493212	
GBAS	0.00493212	
SLC24A1	0.00493212	
KLK3	0.00493212	
TOB1-AS1	0.00493212	
PPP1R13L	0.00493212	
NNT	0.00493212	
VEZF1	0.00493212	
ALG2	0.00493212	
MLEC	0.00493212	
FAM53B	0.00493212	
TRIM31	0.00493212	
SLC51A	0.00493212	

PREX1	0.00493212	
OAF	0.00493212	
MEP1A	0.00493212	
CCDC107	0.00493212	
EFNB3	0.00493212	
ACY3	0.00493212	
AOC2	0.00493212	
SEMA6D	0.00493212	
MAML3	0.00493212	
RASSF8	0.00493212	
FAM107A	0.00493212	
RHPN2	0.00493212	
HADH	0.00493212	
GLCCI1	0.00493212	
MXRA5	0.00493212	
GRHL1	0.00493212	
BEND7	0.00493212	
PRKCDBP	0.00493212	
GSTCD	0.00493212	
CIT	0.00493212	
TDRKH	0.00493212	
MX1	0.00493212	
SLAMF6	0.00493212	
RAB2A	0.00493212	
CDKN2D	0.00493212	
TMEM141	0.00493212	
PSMC3IP	0.00493212	
MPZL1	0.00493212	
SLC7A8	0.00493212	
TSPAN15	0.00493212	
SULT1B1	0.00493212	
DBT	0.00493212	
CCDC24	0.00493212	
H2AFY	0.00493212	
ERLIN1	0.00493212	
CUL9	0.00493212	
ZNF644	0.00493212	
STXBP2	0.00493212	
ZBTB34	0.00493212	
UBE2O	0.00493212	
ZNF638	0.004881273	

THAP11	0.004881273	
FADS1	0.004881273	
NFATC2IP	0.004881273	
DFFB	0.004881273	
EXOC3-AS1	0.004881273	
TMC8	0.004881273	
TMBIM6	0.004881273	
RWDD1	0.004881273	
EID1	0.004881273	
SLX1B	0.004881273	
VBP1	0.004881273	
AEBP2	0.004881273	
LINC00543	0.004881273	
LFNG	0.004881273	
ABHD10	0.004881273	
OLFM2	0.004881273	
C20orf27	0.004881273	
R3HCC1L	0.004881273	
GRIN2B	0.004881273	
DDHD2	0.004881273	
SLC4A4	0.004881273	
CKAP4	0.004881273	
AP000892.6	0.004881273	
DDX23	0.004881273	
CLEC4G	0.004881273	
CHTOP	0.004881273	
AC005154.6	0.004881273	
SEL1L	0.004881273	
POLK	0.004881273	
CXorf40B	0.004881273	
ABCF3	0.004881273	
MS4A6A	0.004881273	
MPPED2	0.004881273	
RP11-51O6.1	0.004881273	
CDK19	0.004881273	
PTGER2	0.004881273	
C6orf203	0.004881273	
AIDA	0.004881273	
ESCO1	0.004881273	
SOX18	0.004881273	
CCDC47	0.004881273	

TOM1	0.004881273	
GFM2	0.004881273	
COMMD3	0.004881273	
ECH1	0.004881273	
CCL19	0.004881273	
GAK	0.004881273	
ALS2	0.004881273	
EXOC1	0.004881273	
MINK1	0.004881273	
SIRPG	0.004881273	
COG8	0.004881273	
CDC16	0.004881273	
SRSF10	0.004881273	
DNTTIP1	0.004881273	
LDLRAD1	0.004881273	
DOT1L	0.004881273	
FGD2	0.004881273	
LSR	0.004881273	
SOCS2	0.004881273	
GLT8D1	0.004881273	
IL1RN	0.004881273	
CPT1C	0.004881273	
ZNF572	0.004881273	
DOCK5	0.004881273	
MYF5	0.004881273	
LPAR5	0.004881273	
TPRA1	0.004881273	
SYT17	0.004881273	
FABP3	0.004881273	
SH2D4A	0.004881273	
AMFR	0.004881273	
RP11-290F5.1	0.004881273	
RPLP0P6	0.004881273	
DGKD	0.004881273	
TECPR2	0.004881273	
TIGIT	0.004881273	
CNTROB	0.004881273	
XPO5	0.004881273	
ATAD2	0.004881273	
ETFA	0.004881273	
EMC2	0.004881273	

ENTPD1	0.004881273	
OGFRL1	0.004881273	
PPP3R1	0.004881273	
SETD1B	0.004881273	
SFMBT1	0.004830427	
MYL12B	0.004830427	
ARHGEF11	0.004830427	
AGPAT3	0.004830427	
SUZ12P1	0.004830427	
ELMO2	0.004830427	
FOXRED2	0.004830427	
KYNU	0.004830427	
CHGB	0.004830427	
PRPSAP2	0.004830427	
EFNA3	0.004830427	
KDM1B	0.004830427	
PKD1	0.004830427	
NR1D2	0.004830427	
EFNA1	0.004830427	
EMX1	0.004830427	
PARPBP	0.004830427	
C9orf89	0.004830427	
TMEM220	0.004830427	
SEPHS1	0.004830427	
CNOT2	0.004830427	
MCM8	0.004830427	
CHN1	0.004830427	
NARS2	0.004830427	
CAB39	0.004830427	
CMC2	0.004830427	
NT5C1B	0.004830427	
CTC-490E21.11	0.004830427	
TMEM43	0.004830427	
SPR	0.004830427	
TTC4	0.004830427	
MRGBP	0.004830427	
PREP	0.004830427	
AHSA1	0.004830427	
PAAF1	0.004830427	
GAB2	0.004830427	
KIAA2013	0.004830427	

MTMR4	0.004830427	
HIST1H3J	0.004830427	
TMEM82	0.004830427	
SPP2	0.004830427	
PPP5C	0.004830427	
WAPAL	0.004830427	
NUP37	0.004830427	
TCF21	0.004830427	
C2CD5	0.004830427	
ZMYM3	0.004830427	
HIBADH	0.004830427	
CAMK1	0.004830427	
SNAPC4	0.004830427	
IGSF6	0.004830427	
CISD1	0.004830427	
ADHFE1	0.004830427	
MDN1	0.004830427	
COPS7A	0.004830427	
HPCAL1	0.004830427	
AMMECR1L	0.004830427	
MYO9B	0.004830427	
USP14	0.004830427	
DSC2	0.004830427	
GREB1	0.004830427	
CCDC132	0.004830427	
VDAC2	0.004830427	
MPEG1	0.004830427	
CCDC93	0.004830427	
CXCL12	0.004830427	
AP1M2	0.004830427	
AIM1	0.004830427	
COL4A3BP	0.004830427	
MPP5	0.004830427	
CENPBD1	0.004830427	
NUDT6	0.004830427	
AGPAT1	0.004830427	
ACKR2	0.004830427	
TP53INP1	0.004830427	
PLCL2	0.004830427	
CCDC8	0.004830427	
PPP2R2D	0.004830427	

ANXA10	0.004830427	
CCDC126	0.004830427	
UBE2V2	0.004830427	
AP1S3	0.004830427	
MAP3K7CL	0.00477958	
C11orf73	0.00477958	
ZNF267	0.00477958	
LATS2	0.00477958	
NOC2L	0.00477958	
OLFML2A	0.00477958	
MUS81	0.00477958	
NIPA2	0.00477958	
PTPRF	0.00477958	
SPHK1	0.00477958	
KLHL9	0.00477958	
MBNL1	0.00477958	
BTD	0.00477958	
PTPRK	0.00477958	
PXDN	0.00477958	
SLC9A9	0.00477958	
POLE3	0.00477958	
ERGIC1	0.00477958	
PPM1M	0.00477958	
RP11-627G23.1	0.00477958	
SLC6A6	0.00477958	
ZNF426	0.00477958	
RP11-396K3.1	0.00477958	
ANKHD1	0.00477958	
HS1BP3	0.00477958	
NPR2	0.00477958	
EAF1	0.00477958	
FANCM	0.00477958	
DIAPH3	0.00477958	
ERCC8	0.00477958	
EXOC3L1	0.00477958	
UCK1	0.00477958	
ALG5	0.00477958	
ACSL6	0.00477958	
CMAS	0.00477958	
PARP10	0.00477958	
NXF1	0.00477958	

MT-RNR1	0.00477958	
MAP10	0.00477958	
RP3-406A7.7	0.00477958	
CASP10	0.00477958	
ZKSCAN3	0.00477958	
SETD4	0.00477958	
CCDC84	0.00477958	
43723	0.00477958	
GAS5	0.00477958	
RAPGEF1	0.00477958	
CRYL1	0.00477958	
SFXN1	0.00477958	
MMACHC	0.00477958	
SIRPB2	0.00477958	
TESK1	0.00477958	
TMEM179B	0.00477958	
PPP6R3	0.00477958	
RIMS1	0.00477958	
HIST1H3E	0.00477958	
ZNF207	0.00477958	
GLG1	0.00477958	
COPB2	0.00477958	
HDLBP	0.00477958	
TPPP3	0.00477958	
MTMR6	0.00477958	
HARS	0.00477958	
DGAT1	0.00477958	
PEX13	0.00477958	
HDAC10	0.00477958	
SLC51B	0.00477958	
INO80E	0.00477958	
C6orf62	0.00477958	
PSMG2	0.00477958	
YIPF6	0.00477958	
GJD2	0.00477958	
LMTK2	0.00477958	
PPID	0.00477958	
SMS	0.00477958	
CYB561D2	0.00477958	
SFRP2	0.00477958	
WASF2	0.00477958	

VARS	0.00477958	
KRR1	0.00477958	
VAMP3	0.00477958	
CD3G	0.00477958	
S100P	0.004728733	
CYP4A11	0.004728733	
CMTM8	0.004728733	
IMPA2	0.004728733	
BAIAP2L1	0.004728733	
TDO2	0.004728733	
EPHA4	0.004728733	
ALG10B	0.004728733	
SNX3	0.004728733	
TMEM54	0.004728733	
IGSF23	0.004728733	
XPO6	0.004728733	
PRUNE	0.004728733	
HPS3	0.004728733	
CYB561D1	0.004728733	
MBD6	0.004728733	
AC092171.4	0.004728733	
GBP1	0.004728733	
APEH	0.004728733	
ZIC1	0.004728733	
SMARCAD1	0.004728733	
NMB	0.004728733	
ATP6AP1	0.004728733	
ARL1	0.004728733	
GRIP1	0.004728733	
POLG2	0.004728733	
RP11-466H18.1	0.004728733	
FUNDC1	0.004728733	
GBE1	0.004728733	
ST6GALNAC3	0.004728733	
RNU1-70P	0.004728733	
GPATCH3	0.004728733	
C20orf194	0.004728733	
TGS1	0.004728733	
RTN1	0.004728733	
SNX5	0.004728733	
MAPKBP1	0.004728733	

TRO	0.004728733	
CRLF3	0.004728733	
SUPT4H1	0.004728733	
CYP2B6	0.004728733	
RP11-283I3.6	0.004728733	
ZFC3H1	0.004728733	
COPS7B	0.004728733	
SPAST	0.004728733	
DHX57	0.004728733	
EPB41L4A	0.004728733	
NOMO2	0.004728733	
MCAT	0.004728733	
VRK1	0.004728733	
GRASP	0.004728733	
HIRIP3	0.004728733	
MUC4	0.004728733	
KIF1C	0.004728733	
GPR4	0.004728733	
THRSP	0.004728733	
NSFL1C	0.004728733	
CLDN5	0.004728733	
TRIM15	0.004728733	
PRKAG2	0.004728733	
ETF1	0.004728733	
SLC15A3	0.004728733	
TAF9B	0.004728733	
NUFIP2	0.004728733	
PCBP3	0.004728733	
MRPS18C	0.004728733	
ST3GAL6	0.004728733	
ABHD17A	0.004728733	
VPREB1	0.004728733	
DOLK	0.004728733	
CYP4F11	0.004728733	
STARD3	0.004728733	
C1R	0.004728733	
MEST	0.004728733	
ZNF598	0.004728733	
GZMM	0.004677887	
EFHC2	0.004677887	
TAGLN2	0.004677887	

ENTPD4	0.004677887	
EPHB3	0.004677887	
CDCA7L	0.004677887	
CSGALNACT2	0.004677887	
NGDN	0.004677887	
RSAD2	0.004677887	
ACVR2B	0.004677887	
SARS2	0.004677887	
MKKS	0.004677887	
ZFX	0.004677887	
RPTOR	0.004677887	
PRPS1	0.004677887	
ETV2	0.004677887	
HERC2	0.004677887	
USP42	0.004677887	
LAMC1	0.004677887	
RP11-390P2.4	0.004677887	
LPPR3	0.004677887	
TP53I11	0.004677887	
H3F3B	0.004677887	
ZKSCAN4	0.004677887	
ADAM9	0.004677887	
GINM1	0.004677887	
TFEB	0.004677887	
LRP8	0.004677887	
TNRC6B	0.004677887	
KIAA0368	0.004677887	
DEAF1	0.004677887	
KDM4A	0.004677887	
HS3ST3B1	0.004677887	
LOXL2	0.004677887	
PCDH18	0.004677887	
GCNT3	0.004677887	
TTC39B	0.004677887	
CDK14	0.004677887	
PHKB	0.004677887	
CELSR2	0.004677887	
CDC14A	0.004677887	
PDXK	0.004677887	
ARMC1	0.004677887	
TSKU	0.004677887	

FAH	0.004677887	
ZDHHC7	0.004677887	
SLC6A13	0.004677887	
TK2	0.004677887	
ZNF563	0.004677887	
ALKBH4	0.004677887	
CNTNAP2	0.004677887	
ARF4	0.004677887	
TSNARE1	0.004677887	
TBRG4	0.004677887	
RAB7B	0.004677887	
KRT20	0.004677887	
FASTKD5	0.004677887	
GPANK1	0.004677887	
ZFAS1	0.004677887	
GIMAP4	0.004677887	
HAGHL	0.004677887	
RHOT2	0.004677887	
MAK16	0.004677887	
NAT6	0.004677887	
PDCL	0.004677887	
SDSL	0.004677887	
PGAP2	0.004677887	
CPNE2	0.004677887	
FLNB	0.004677887	
VEZT	0.004677887	
NUDT1	0.004677887	
SRP54	0.004677887	
DSTN	0.004677887	
ARPC2	0.004677887	
CD83	0.004677887	
NRD1	0.004677887	
PYCR2	0.004677887	
KITLG	0.00462704	
SNHG19	0.00462704	
BLMH	0.00462704	
HNMT	0.00462704	
CCAT1	0.00462704	
MRPL3	0.00462704	
SCN8A	0.00462704	
RP11-480I12.5	0.00462704	

STRADA	0.00462704	
SERGEF	0.00462704	
BICC1	0.00462704	
DUSP28	0.00462704	
HLA-DQB1	0.00462704	
SEC61A1	0.00462704	
WNT1	0.00462704	
GZMB	0.00462704	
TMEM187	0.00462704	
CBLL1	0.00462704	
FAM175B	0.00462704	
ZSCAN25	0.00462704	
GEMIN5	0.00462704	
ALDH16A1	0.00462704	
MRPL32	0.00462704	
EMBP1	0.00462704	
C1QTNF3	0.00462704	
RP11-299G20.5	0.00462704	
RP5-1085F17.3	0.00462704	
DCP2	0.00462704	
C16orf70	0.00462704	
MAP1LC3C	0.00462704	
SLAMF1	0.00462704	
TOR1A	0.00462704	
BANF1P2	0.00462704	
RNFT1	0.00462704	
WNT5A	0.00462704	
CXCR2	0.00462704	
INPPL1	0.00462704	
CD22	0.00462704	
RAB37	0.00462704	
CCDC152	0.00462704	
PCNT	0.00462704	
GALNT7	0.00462704	
RBM17	0.00462704	
UNK	0.00462704	
NPIPP1	0.00462704	
THAP3	0.00462704	
PALM	0.00462704	
HSD11B1L	0.00462704	
SETBP1	0.00462704	

KLHL2	0.00462704	
AZIN1	0.00462704	
ENDOD1	0.00462704	
ZNF225	0.00462704	
ISG20	0.00462704	
ZBTB38	0.00462704	
HOXC8	0.00462704	
CD1D	0.00462704	
NCBP2-AS2	0.00462704	
ZNF383	0.00462704	
SCOC	0.00462704	
EXO5	0.00462704	
SPPL2B	0.00462704	
HN1L	0.00462704	
MAGEA12	0.00462704	
USP32	0.00462704	
ASIC1	0.00462704	
ZNF689	0.00462704	
ARHGAP35	0.00462704	
ICK	0.00462704	
PTCD2	0.00462704	
RFX7	0.00462704	
MRPS35	0.00462704	
ARHGEF39	0.00462704	
PARD6G	0.00462704	
UMPS	0.00462704	
KHDRBS3	0.00462704	
SLIT3	0.00462704	
MCOLN1	0.00462704	
ATL3	0.00462704	
HIST1H2BD	0.00462704	
CTD-2270L9.4	0.00462704	
SPRYD7	0.00462704	
TRMT1	0.00462704	
WIZ	0.00462704	
AMT	0.00462704	
POC1A	0.00462704	
POLD3	0.00462704	
ALCAM	0.00462704	
LMTK3	0.00462704	
PTPLB	0.00462704	

ZNF362	0.004576194	
CHID1	0.004576194	
WDR60	0.004576194	
RANBP10	0.004576194	
ITGB8	0.004576194	
FBXO2	0.004576194	
RP5-1074L1.4	0.004576194	
CETN2	0.004576194	
ABCC1	0.004576194	
RP11-33E12.2	0.004576194	
KLRB1	0.004576194	
CRELD1	0.004576194	
ORAI1	0.004576194	
KRTAP4-2	0.004576194	
RRAD	0.004576194	
MRPS6	0.004576194	
RAB25	0.004576194	
MYO1C	0.004576194	
NAT1	0.004576194	
PGRMC1	0.004576194	
MAGI2	0.004576194	
AARS	0.004576194	
ANGPTL6	0.004576194	
NCF2	0.004576194	
HN1	0.004576194	
MAST2	0.004576194	
LOR	0.004576194	
PGAM1	0.004576194	
NFASC	0.004576194	
UHRF2	0.004576194	
RSL24D1	0.004576194	
BEX2	0.004576194	
SPARCL1	0.004576194	
HABP2	0.004576194	
ESD	0.004576194	
CAP1	0.004576194	
C1D	0.004576194	
RAB11B-AS1	0.004576194	
PNMA5	0.004576194	
ZNFX1	0.004576194	
PSD4	0.004576194	

CD300LF	0.004576194	
TBC1D9B	0.004576194	
SIGLEC1	0.004576194	
RAMP3	0.004576194	
HSD11B1	0.004576194	
NPAS4	0.004576194	
CCDC51	0.004576194	
SIGMAR1	0.004576194	
FAM129B	0.004576194	
MMRN2	0.004576194	
BBC3	0.004576194	
NDFIP1	0.004576194	
UBFD1	0.004576194	
PLLP	0.004576194	
UPK3A	0.004576194	
EFCC1	0.004576194	
DDR2	0.004576194	
SEC23IP	0.004576194	
ZBTB33	0.004576194	
IGLV2-14	0.004576194	
MYF6	0.004576194	
RP11-661A12.8	0.004576194	
POLI	0.004576194	
AHDC1	0.004576194	
PKIG	0.004525347	
HSPB3	0.004525347	
FAM127A	0.004525347	
MEGF9	0.004525347	
IER3IP1	0.004525347	
GLTSCR2	0.004525347	
URM1	0.004525347	
FGD4	0.004525347	
WDR18	0.004525347	
TMTC3	0.004525347	
TIMM23	0.004525347	
TUBB2B	0.004525347	
IGHG3	0.004525347	
NAE1	0.004525347	
NSUN5	0.004525347	
CAND2	0.004525347	
NBR1	0.004525347	

DCTN4	0.004525347	
SPIN3	0.004525347	
LARP4B	0.004525347	
TMEM245	0.004525347	
EXD3	0.004525347	
NAF1	0.004525347	
EEF1A1P9	0.004525347	
ATXN10	0.004525347	
AL161668.5	0.004525347	
FABP5	0.004525347	
VIPR1	0.004525347	
INTS5	0.004525347	
HOXB13	0.004525347	
PBDC1	0.004525347	
TRAF7	0.004525347	
EDIL3	0.004525347	
HNRNPH3	0.004525347	
IL1A	0.004525347	
HSPB7	0.004525347	
ZNF140	0.004525347	
RP11-140K17.3	0.004525347	
CDKAL1	0.004525347	
DDX28	0.004525347	
IL13RA1	0.004525347	
TMEM11	0.004525347	
ACBD4	0.004525347	
EIF4H	0.004525347	
USP4	0.004525347	
NPC1L1	0.004525347	
AHCTF1	0.004525347	
WIPF2	0.004525347	
TMEM129	0.004525347	
IPO8	0.004525347	
CUEDC1	0.004525347	
RAB9A	0.004525347	
SDF4	0.004525347	
PXDC1	0.004525347	
NNMT	0.004525347	
UST	0.004525347	
STT3A	0.004525347	
KCNK5	0.004525347	

PTPRG	0.004525347	
PDE4A	0.004525347	
EMR2	0.004525347	
ARHGEF15	0.004525347	
GNG5	0.004525347	
C5orf15	0.004525347	
APOC1P1	0.004525347	
ADCY10	0.004525347	
SULT1E1	0.004525347	
DLX5	0.004525347	
VPS26B	0.004525347	
ATMIN	0.004525347	
PDE11A	0.004525347	
MND1	0.004525347	
PRKAG1	0.004525347	
BICD2	0.004525347	
KTI12	0.004525347	
ZNF202	0.004525347	
CYBA	0.004525347	
LYSMD3	0.004525347	
TOMM40L	0.004525347	
HAS2	0.004525347	
NUP188	0.004525347	
HERC2P9	0.004525347	
ARL4A	0.004525347	
PTHLH	0.004525347	
RSBN1L	0.004525347	
ZNF407	0.004525347	
RADIL	0.004525347	
CDKN2B	0.004525347	
CNTNAP1	0.004525347	
DNAJC10	0.0044745	
PAQR9	0.0044745	
RND1	0.0044745	
S100A1	0.0044745	
C21orf58	0.0044745	
CYP2C19	0.0044745	
BEND3	0.0044745	
DHRS3	0.0044745	
SUGT1	0.0044745	
HDGFRP2	0.0044745	

SLK	0.0044745	
CFHR3	0.0044745	
FXYD5	0.0044745	
LINC00869	0.0044745	
SELO	0.0044745	
AKAP1	0.0044745	
HSPB8	0.0044745	
ICE1	0.0044745	
SCTR	0.0044745	
CELSR3	0.0044745	
RNF152	0.0044745	
KRT7	0.0044745	
FAM219A	0.0044745	
HOXA7	0.0044745	
RP11-612B6.2	0.0044745	
TTC3	0.0044745	
SLC7A6OS	0.0044745	
PID1	0.0044745	
GMIP	0.0044745	
HDAC8	0.0044745	
C16orf58	0.0044745	
GRB10	0.0044745	
PPM1F	0.0044745	
STAMBPL1	0.0044745	
PRF1	0.0044745	
RNF185	0.0044745	
HOXC4	0.0044745	
ANKZF1	0.0044745	
CCDC106	0.0044745	
ANAPC5	0.0044745	
PWWP2B	0.0044745	
HNF4A-AS1	0.0044745	
TCIRG1	0.0044745	
USP30-AS1	0.0044745	
PSME2P2	0.0044745	
TUBA3C	0.0044745	
CGGBP1	0.0044745	
FUT6	0.0044745	
PAK7	0.0044745	
CHRDL2	0.0044745	
REPS2	0.0044745	

SDE2	0.0044745	
DFFA	0.0044745	
FCHO2	0.0044745	
NELFB	0.0044745	
HIST2H4A	0.0044745	
MIA3	0.0044745	
PPAT	0.0044745	
ST3GAL3	0.0044745	
CEP131	0.0044745	
MAGED2	0.0044745	
GHITM	0.0044745	
ELTD1	0.0044745	
GYLTL1B	0.0044745	
PPP1R12C	0.0044745	
CPS1	0.0044745	
MINA	0.0044745	
TMEM62	0.0044745	
CYFIP2	0.0044745	
LTBP1	0.0044745	
FARSA	0.0044745	
ACBD5	0.0044745	
DLGAP4	0.0044745	
CXorf40A	0.0044745	
ZNF707	0.0044745	
VPS13A	0.0044745	
AP4B1	0.0044745	
RP1-28O10.1	0.0044745	
PDXDC1	0.0044745	
CHTF8	0.0044745	
ARHGAP29	0.0044745	
CBR1	0.0044745	
HBEGF	0.0044745	
FAM60A	0.0044745	
SERINC3	0.0044745	
YIF1B	0.0044745	
MIPOL1	0.0044745	
DNAJC4	0.0044745	
ENPP7	0.0044745	
RQCD1	0.0044745	
IGKV3-20	0.0044745	
HS3ST2	0.0044745	

KIF5A	0.0044745	
CNTFR	0.0044745	
MTX1	0.0044745	
KLHL6	0.004423654	
SEMA6C	0.004423654	
KCTD20	0.004423654	
TSPAN5	0.004423654	
PPM1B	0.004423654	
PAQR9-AS1	0.004423654	
NDE1	0.004423654	
AHRR	0.004423654	
MARS	0.004423654	
FBXO38	0.004423654	
TBC1D12	0.004423654	
STOML1	0.004423654	
PIP4K2A	0.004423654	
BST2	0.004423654	
METTL16	0.004423654	
CCNG1	0.004423654	
SNHG9	0.004423654	
RUSC1-AS1	0.004423654	
DUS1L	0.004423654	
ISOC1	0.004423654	
SYNPO	0.004423654	
CHCHD7	0.004423654	
FARS2	0.004423654	
CIRH1A	0.004423654	
ANAPC2	0.004423654	
MBD5	0.004423654	
SYTL1	0.004423654	
LAD1	0.004423654	
P2RX7	0.004423654	
MATN2	0.004423654	
URI1	0.004423654	
TNC	0.004423654	
GINS3	0.004423654	
CPXM1	0.004423654	
APH1A	0.004423654	
L3MBTL1	0.004423654	
IGLV1-51	0.004423654	
POLN	0.004423654	

GTF2H2	0.004423654	
MAN1A1	0.004423654	
HGH1	0.004423654	
BDKRB2	0.004423654	
APLNR	0.004423654	
FERMT1	0.004423654	
EPN1	0.004423654	
PON2	0.004423654	
TRAPPC2	0.004423654	
C6orf211	0.004423654	
HSPBAP1	0.004423654	
HIVEP3	0.004423654	
SSH3	0.004423654	
LIMD2	0.004423654	
FGL2	0.004423654	
PMM2	0.004423654	
SFSWAP	0.004423654	
MMP25	0.004423654	
ADNP2	0.004423654	
PVRL2	0.004423654	
CLEC16A	0.004423654	
CTGF	0.004423654	
SURF6	0.004423654	
NUMB	0.004423654	
RBM10	0.004423654	
LYRM9	0.004423654	
FYCO1	0.004423654	
RHOQ	0.004423654	
SEMA6B	0.004423654	
SOCS7	0.004423654	
PODXL	0.004423654	
MYO9A	0.004423654	
RHOBTB3	0.004423654	
F2RL3	0.004423654	
FANCC	0.004423654	
SH2B2	0.004423654	
GGPS1	0.004423654	
DKK1	0.004423654	
MAPRE3	0.004423654	
TMEM204	0.004423654	
GATAD2A	0.004423654	

CD3D	0.004423654	
RSPRY1	0.004423654	
NR2C1	0.004423654	
SDHA	0.004423654	
KRT6B	0.004423654	
MDH1	0.004423654	
LTBR	0.004423654	
QRSL1	0.004423654	
IFITM1	0.004372807	
ZDHHC23	0.004372807	
EIF5	0.004372807	
RAB11FIP3	0.004372807	
ZGPAT	0.004372807	
SCLY	0.004372807	
CACFD1	0.004372807	
F5	0.004372807	
NME6	0.004372807	
MT1X	0.004372807	
ARHGEF7	0.004372807	
CCNG2	0.004372807	
SPTY2D1	0.004372807	
MIS12	0.004372807	
CSNK1G2	0.004372807	
SAT2	0.004372807	
CCNL2	0.004372807	
C1QTNF1	0.004372807	
CHCHD6	0.004372807	
ALG1L	0.004372807	
IL1RAP	0.004372807	
RBM23	0.004372807	
ARHGAP17	0.004372807	
CYTIP	0.004372807	
JAKMIP2	0.004372807	
RMDN3	0.004372807	
SMG7	0.004372807	
LRPAP1	0.004372807	
ADCY1	0.004372807	
IFFO1	0.004372807	
ZNF706	0.004372807	
CERKL	0.004372807	
SLC25A28	0.004372807	

CHKB	0.004372807	
MBTPS1	0.004372807	
SBF1	0.004372807	
MTCL1	0.004372807	
TNNI3	0.004372807	
MIIP	0.004372807	
ABCC6P2	0.004372807	
LEPR	0.004372807	
MADD	0.004372807	
PDCL3	0.004372807	
RNF130	0.004372807	
ABTB1	0.004372807	
CES3	0.004372807	
CRAMP1L	0.004372807	
SLC39A14	0.004372807	
CASP5	0.004372807	
COX7A2	0.004372807	
TRAM1	0.004372807	
DCTD	0.004372807	
BAZ1A	0.004372807	
FAM193B	0.004372807	
PRMT7	0.004372807	
CCDC3	0.004372807	
TPRG1	0.004372807	
TAOK3	0.004372807	
SYT7	0.004372807	
TPPP	0.004372807	
MAP4	0.004372807	
FRMD4B	0.004372807	
MLF2	0.004372807	
TRAF3IP1	0.004372807	
C1orf115	0.004372807	
KCNF1	0.004372807	
MBTPS2	0.004372807	
AVL9	0.004372807	
CACTIN	0.004372807	
ACSF2	0.004372807	
URAHP	0.004372807	
GRTP1	0.004372807	
CSTF1	0.004372807	
INPP4A	0.004372807	

GLS	0.004372807	
PITPNA	0.004372807	
WNK2	0.004372807	
CADM1	0.004372807	
NUP210	0.004372807	
HOMER1	0.004372807	
PAXIP1-AS1	0.004372807	
ACTR1A	0.004372807	
ACSL4	0.004372807	
TMEM176A	0.004372807	
CCDC25	0.004372807	
MKNK2	0.004372807	
SMOX	0.004372807	
BMX	0.004372807	
STMN3	0.004372807	
ARHGEF10	0.004372807	
AC007969.5	0.004372807	
MTATP6P1	0.004372807	
SLC39A1	0.004372807	
PDLIM5	0.004372807	
METTL7B	0.004372807	
TPPP2	0.004321961	
ITPKC	0.004321961	
AP1S1	0.004321961	
PIGC	0.004321961	
CS	0.004321961	
SNRNP25	0.004321961	
GPC6	0.004321961	
BNIP1	0.004321961	
CD226	0.004321961	
GCM1	0.004321961	
ANKRD11	0.004321961	
TMEM47	0.004321961	
APIP	0.004321961	
AGL	0.004321961	
SKAP1	0.004321961	
GOLGA7	0.004321961	
TSTA3	0.004321961	
CHPF	0.004321961	
BBX	0.004321961	
SMO	0.004321961	

HSDL1	0.004321961	
ATXN7L3B	0.004321961	
CDH24	0.004321961	
DDX54	0.004321961	
NUPR1	0.004321961	
ZFYVE1	0.004321961	
MMAA	0.004321961	
MAT2A	0.004321961	
FAM111A	0.004321961	
ELP2	0.004321961	
SERPINB9	0.004321961	
RPL13AP20	0.004321961	
PPFIA3	0.004321961	
FUT11	0.004321961	
C16orf54	0.004321961	
STK10	0.004321961	
MBOAT2	0.004321961	
ACLY	0.004321961	
CATSPER1	0.004321961	
FBXL6	0.004321961	
DNAJA2	0.004321961	
ARHGAP32	0.004321961	
IRAK3	0.004321961	
METTL22	0.004321961	
ACSF3	0.004321961	
TAF3	0.004321961	
SEPN1	0.004321961	
RP11-445P17.6	0.004321961	
PORCN	0.004321961	
CECR2	0.004321961	
IFNGR1	0.004321961	
SKIV2L	0.004321961	
DDX19A	0.004321961	
FKBP5	0.004321961	
COG4	0.004321961	
EPB41L1	0.004321961	
LRRC41	0.004321961	
LINC01127	0.004321961	
SYNPO2	0.004321961	
EPB41L4A-AS1	0.004321961	
LZTS1	0.004321961	

MUTYH	0.004321961	
PANK3	0.004321961	
PTPN9	0.004321961	
EPHA1	0.004321961	
EIF1AD	0.004321961	
DNPEP	0.004321961	
HMGA2	0.004321961	
FAM167B	0.004321961	
AADACP1	0.004321961	
LMCD1	0.004321961	
COG1	0.004321961	
PPDPF	0.004321961	
NAT10	0.004321961	
WNK3	0.004321961	
AC111186.1	0.004321961	
FAM129A	0.004321961	
RARS2	0.004321961	
USP24	0.004321961	
FAM26F	0.004321961	
EPG5	0.004321961	
TTC6	0.004321961	
EXOC3L2	0.004321961	
KDM3A	0.004321961	
SERPINE2	0.004321961	
TMEM57	0.004321961	
ZNF800	0.004321961	
C5orf63	0.004321961	
FAM127C	0.004321961	
NGRN	0.004321961	
TTLL4	0.004271114	
ZMYM6NB	0.004271114	
NCAM1	0.004271114	
RP11-588K22.2	0.004271114	
ITGB7	0.004271114	
TEX2	0.004271114	
SEPT7P2	0.004271114	
CCL25	0.004271114	
KLF1	0.004271114	
ZBTB5	0.004271114	
PHF6	0.004271114	
ZFAND6	0.004271114	

HSPB2	0.004271114	
ACVRL1	0.004271114	
ALG12	0.004271114	
SLBP	0.004271114	
PCBP4	0.004271114	
TMEM88	0.004271114	
AASDHPPT	0.004271114	
VPS35	0.004271114	
GSC	0.004271114	
TXNL1	0.004271114	
MAP1S	0.004271114	
STAP2	0.004271114	
IFT27	0.004271114	
ANKRD32	0.004271114	
SMCO4	0.004271114	
TECPR1	0.004271114	
CYP11B2	0.004271114	
EXOG	0.004271114	
RSRP1	0.004271114	
NES	0.004271114	
SFI1	0.004271114	
IGKV4-1	0.004271114	
NHLRC2	0.004271114	
POGK	0.004271114	
NFXL1	0.004271114	
EFR3A	0.004271114	
MOB3C	0.004271114	
NRBP1	0.004271114	
SKOR1	0.004271114	
CNTN1	0.004271114	
RASIP1	0.004271114	
BCAT1	0.004271114	
GS1-124K5.11	0.004271114	
ITGA1	0.004271114	
BAIAP2	0.004271114	
BROX	0.004271114	
ASCC1	0.004271114	
RSPO2	0.004271114	
FAM3C	0.004271114	
HFE	0.004271114	
CERS5	0.004271114	

DPYSL2	0.004271114	
GLS2	0.004271114	
RUSC2	0.004271114	
ZNF548	0.004271114	
KCNMA1	0.004271114	
GPR124	0.004271114	
FOXP4	0.004271114	
CTB-50L17.14	0.004271114	
BMPR1A	0.004271114	
AMPH	0.004271114	
MPC2	0.004271114	
XXYLT1	0.004271114	
PCNXL2	0.004271114	
DENND4B	0.004271114	
TRMT13	0.004271114	
FOXJ3	0.004271114	
RNF146	0.004271114	
DNAJB12	0.004271114	
SLC38A6	0.004271114	
IARS2	0.004271114	
CD163	0.004271114	
EPPK1	0.004271114	
TULP3	0.004271114	
PRSS36	0.004271114	
ZNF32	0.004271114	
HIST1H3G	0.004271114	
FAM195B	0.004271114	
BATF3	0.004271114	
FAM162A	0.004271114	
GALNT11	0.004271114	
ACAA2	0.004271114	
DENR	0.004271114	
SLX1A	0.004271114	
C1orf162	0.004220267	
SPATA13	0.004220267	
CHCHD4	0.004220267	
CCDC130	0.004220267	
TCL1A	0.004220267	
TSPYL1	0.004220267	
PLEKHJ1	0.004220267	
GDF11	0.004220267	

DNPH1	0.004220267	
HAO2	0.004220267	
SLC35A3	0.004220267	
DPP7	0.004220267	
NEU4	0.004220267	
PLCE1	0.004220267	
CYB5D2	0.004220267	
CRCP	0.004220267	
COL7A1	0.004220267	
FAM168B	0.004220267	
TRAPPC6B	0.004220267	
SDHAP1	0.004220267	
RP11-475C16.1	0.004220267	
PLEKHG5	0.004220267	
GRK5	0.004220267	
CR1	0.004220267	
AACS	0.004220267	
COL4A3	0.004220267	
CDC42BPB	0.004220267	
RPUSD2	0.004220267	
IGLC3	0.004220267	
KIF21A	0.004220267	
ABHD15	0.004220267	
CCL21	0.004220267	
AKR1B10	0.004220267	
LONRF1	0.004220267	
FAM101B	0.004220267	
POMGNT1	0.004220267	
SEC63	0.004220267	
UFSP1	0.004220267	
RAI2	0.004220267	
RP11-372E1.4	0.004220267	
TMC5	0.004220267	
ARNTL2	0.004220267	
ADK	0.004220267	
LYRM2	0.004220267	
AKNA	0.004220267	
ABHD8	0.004220267	
DHRS4	0.004220267	
LMO7	0.004220267	
RP11-452F19.3	0.004220267	

HDAC11	0.004220267	
KIAA1107	0.004220267	
ACADS	0.004220267	
MMP3	0.004220267	
UNC5CL	0.004220267	
RBP1	0.004220267	
ANO9	0.004220267	
NSDHL	0.004220267	
UHRF1BP1L	0.004220267	
C1orf106	0.004220267	
OXA1L	0.004220267	
FZD8	0.004220267	
KIAA0922	0.004220267	
ACYP2	0.004220267	
RC3H1	0.004220267	
AGRN	0.004220267	
LUM	0.004220267	
SLC7A6	0.004220267	
SS18	0.004220267	
SNCAIP	0.004220267	
PPP1R15B	0.004220267	
ANKRD27	0.004220267	
FCER2	0.004220267	
HOPX	0.004220267	
ZBTB41	0.004220267	
ACVR2A	0.004220267	
POFUT1	0.004220267	
ABHD14A	0.004220267	
C9	0.004220267	
ADH4	0.004220267	
DCDC2	0.004220267	
RPL15P3	0.004220267	
PEX26	0.004220267	
PIGT	0.004220267	
NANS	0.004220267	
C6orf48	0.004220267	
ACSS2	0.004220267	
CCL20	0.004220267	
CDKN2AIPNL	0.004220267	
SMC6	0.004220267	
UACA	0.004220267	

PRPSAP1	0.004220267	
COPZ1	0.004220267	
SLC12A2	0.004220267	
KMT2E-AS1	0.004220267	
ZNF789	0.004220267	
MGLL	0.004220267	
AP2A2	0.004220267	
MRPL57	0.004220267	
UBN1	0.004169421	
KCNN3	0.004169421	
ITGAL	0.004169421	
FAM179B	0.004169421	
CD68	0.004169421	
DNAJC5	0.004169421	
ARMCX1	0.004169421	
SERPINB1	0.004169421	
BAHD1	0.004169421	
PRKAR1B	0.004169421	
RAB5B	0.004169421	
AC009303.2	0.004169421	
ERVW-1	0.004169421	
STAM	0.004169421	
FLOT2	0.004169421	
ADORA3	0.004169421	
GOSR1	0.004169421	
ZNF37A	0.004169421	
ZNHIT6	0.004169421	
PARD3B	0.004169421	
NAT14	0.004169421	
RFX6	0.004169421	
MATK	0.004169421	
CPE	0.004169421	
ASB9	0.004169421	
LINC01023	0.004169421	
MRPL48	0.004169421	
RAB27B	0.004169421	
KCNE4	0.004169421	
DLGAP1-AS1	0.004169421	
TRDMT1	0.004169421	
PDSS2	0.004169421	
SYNJ2	0.004169421	

DDX42	0.004169421	
PARP3	0.004169421	
SH3BGRL2	0.004169421	
CAPN10	0.004169421	
TMPRSS3	0.004169421	
LECT2	0.004169421	
RCN2	0.004169421	
BCAM	0.004169421	
TOP3A	0.004169421	
GRK4	0.004169421	
TRAPPC11	0.004169421	
EIF4EBP2	0.004169421	
B3GNT3	0.004169421	
ALDH3B2	0.004169421	
PABPC4	0.004169421	
FOXD2	0.004169421	
CMTM6	0.004169421	
CEP95	0.004169421	
ZDHHC1	0.004169421	
PROSC	0.004169421	
DKFZp779M0652	0.004169421	
RP5-857K21.11	0.004169421	
ACTG2	0.004169421	
CPNE3	0.004169421	
HR	0.004169421	
FAM13A	0.004169421	
UCHL1	0.004169421	
S1PR1	0.004169421	
GRK6	0.004169421	
ANXA9	0.004169421	
ABHD13	0.004169421	
C16orf95	0.004169421	
VPS8	0.004169421	
RBFOX1	0.004169421	
IGHD	0.004169421	
NABP2	0.004169421	
CLK3	0.004169421	
NOD2	0.004169421	
UGT2B11	0.004169421	
TESK2	0.004169421	
VASN	0.004169421	

ORC5	0.004169421	
CYP2A13	0.004169421	
RBM15	0.004169421	
ADCK1	0.004169421	
DPF2	0.004169421	
SNTB2	0.004169421	
CTA-292E10.6	0.004169421	
TTC39A	0.004169421	
UGT2B15	0.004169421	
SIX3	0.004169421	
KIF24	0.004169421	
PPP6R1	0.004169421	
DKK3	0.004169421	
CREBRF	0.004169421	
TNS1	0.004169421	
CHMP7	0.004169421	
UBR7	0.004169421	
ABCB10	0.004118574	
PPIL3	0.004118574	
CD274	0.004118574	
PPP1R37	0.004118574	
MON1A	0.004118574	
ZC3H12A	0.004118574	
RIMKLB	0.004118574	
PAXBP1	0.004118574	
CASP16	0.004118574	
EDC3	0.004118574	
FAM98B	0.004118574	
ATG101	0.004118574	
FMNL1	0.004118574	
GP1BA	0.004118574	
SZRD1	0.004118574	
NUDT16L1	0.004118574	
NEAT1	0.004118574	
CYP2B7P	0.004118574	
ZNF398	0.004118574	
TBC1D7	0.004118574	
RAD1	0.004118574	
NRAP	0.004118574	
SIDT1	0.004118574	
TECR	0.004118574	

WSB1	0.004118574	
PTPMT1	0.004118574	
CLDN3	0.004118574	
AMMECR1	0.004118574	
LHCGR	0.004118574	
MAN2B1	0.004118574	
CCR2	0.004118574	
COMMD3-BMI1	0.004118574	
TRPM2	0.004118574	
VPS11	0.004118574	
FANCE	0.004118574	
CPED1	0.004118574	
UGT1A9	0.004118574	
COPRS	0.004118574	
NPPA	0.004118574	
ASXL1	0.004118574	
SHC2	0.004118574	
KLHL38	0.004118574	
FAM199X	0.004118574	
CCDC22	0.004118574	
SLC27A1	0.004118574	
GPRC5A	0.004118574	
SLC39A9	0.004118574	
KIAA0753	0.004118574	
CXorf57	0.004118574	
RALYL	0.004118574	
HIST2H2AC	0.004118574	
SLC7A11	0.004118574	
GCNT1	0.004118574	
TMED10	0.004118574	
ZMIZ2	0.004118574	
TYMP	0.004118574	
TREM2	0.004118574	
NDNL2	0.004118574	
GNMT	0.004118574	
MAEA	0.004118574	
TMEM99	0.004118574	
PRODH	0.004118574	
AIG1	0.004118574	
TNS3	0.004118574	
FUT8	0.004118574	

MILR1	0.004118574	
FAM13C	0.004118574	
TMED8	0.004118574	
SSBP2	0.004118574	
CCSER2	0.004118574	
DNM1L	0.004118574	
ANGEL2	0.004118574	
C4orf48	0.004118574	
GNG4	0.004118574	
PRX	0.004118574	
LUZP1	0.004118574	
ADAP2	0.004118574	
IVNS1ABP	0.004118574	
OLFML3	0.004118574	
GLMN	0.004118574	
ZNF615	0.004118574	
GRB14	0.004118574	
HBB	0.004118574	
CARD8	0.004118574	
HIST1H4A	0.004118574	
PRKD3	0.004118574	
CCRL2	0.004118574	
SLC39A7	0.004118574	
USP47	0.004118574	
PANX1	0.004118574	
ZNRD1-AS1	0.004118574	
ZRSR2	0.004118574	
POLG	0.004118574	
LRRC8B	0.004118574	
SPTBN4	0.004118574	
KTN1	0.004118574	
CFAP44	0.004118574	
PCDH17	0.004118574	
AC104667.3	0.004118574	
DLGAP1	0.004118574	
ASPA	0.004118574	
KLC4	0.004118574	
PLXNB1	0.004118574	
GGNBP2	0.004118574	
SNX22	0.004067728	
SIX1	0.004067728	

LPCAT2	0.004067728	
SHB	0.004067728	
CPTP	0.004067728	
PPP1R10	0.004067728	
LPAR1	0.004067728	
CYP51A1	0.004067728	
GLTPD2	0.004067728	
DUSP22	0.004067728	
ZNF420	0.004067728	
GANAB	0.004067728	
PLXND1	0.004067728	
SH2B1	0.004067728	
NCKIPSD	0.004067728	
C6orf120	0.004067728	
NDRG3	0.004067728	
EPS15L1	0.004067728	
SRGAP2	0.004067728	
PLP2	0.004067728	
FEM1B	0.004067728	
NBPF12	0.004067728	
SPSB1	0.004067728	
CTSG	0.004067728	
MLLT1	0.004067728	
DCTN6	0.004067728	
MYDGF	0.004067728	
JRKL	0.004067728	
RNF114	0.004067728	
DSCR3	0.004067728	
METTL18	0.004067728	
RND3	0.004067728	
FBRS	0.004067728	
R3HDM4	0.004067728	
CLDND2	0.004067728	
SRXN1	0.004067728	
MEF2BNB-MEF2B	0.004067728	
FNIP2	0.004067728	
FANCL	0.004067728	
IL17RC	0.004067728	
PCOLCE2	0.004067728	
AC079922.2	0.004067728	
SH3YL1	0.004067728	

LAP3	0.004067728	
43527	0.004067728	
NFS1	0.004067728	
CLINT1	0.004067728	
EXOSC3	0.004067728	
ZNF484	0.004067728	
CHP1	0.004067728	
SLCO2A1	0.004067728	
CHML	0.004067728	
KCNT2	0.004067728	
MPRIP	0.004067728	
C7orf25	0.004067728	
OR7E38P	0.004067728	
IL15RA	0.004067728	
MFAP3L	0.004067728	
TIPARP-AS1	0.004067728	
BAG4	0.004067728	
DENND2C	0.004067728	
TMEM170B	0.004067728	
EYA2	0.004067728	
C14orf159	0.004067728	
STRIP1	0.004067728	
VPS16	0.004067728	
MRPS17	0.004067728	
F13A1	0.004067728	
WIPI2	0.004067728	
RP1-95L4.4	0.004067728	
PPP1R2	0.004067728	
PSMF1	0.004067728	
FOXK1	0.004067728	
CTA-384D8.36	0.004067728	
SYS1	0.004067728	
CNGA1	0.004067728	
BEGAIN	0.004067728	
XAF1	0.004067728	
CXCR5	0.004067728	
CRELD2	0.004067728	
RBM4	0.004067728	
RNASEK	0.004067728	
SLC1A1	0.004067728	
SNHG7	0.004067728	

FAM222B	0.004067728	
TIMM8A	0.004067728	
RAB34	0.004067728	
AFF4	0.004067728	
EEF1A1P19	0.004067728	
DDRGK1	0.004067728	
PIGP	0.004067728	
BSCL2	0.004067728	
TMEM220-AS1	0.004067728	
TINF2	0.004067728	
NINJ2	0.004067728	
RSPO3	0.004067728	
NSL1	0.004067728	
TSEN54	0.004067728	
MPPED1	0.004067728	
ZNF664	0.004067728	
BACE2	0.004067728	
RASA3	0.004067728	
PDDC1	0.004067728	
ZNF444	0.004067728	
IDE	0.004067728	
CNIH3	0.004067728	
FTH1P20	0.004067728	
SLC30A9	0.004067728	
PRR15L	0.004067728	
ZNF18	0.004067728	
POT1	0.004067728	
GZMK	0.004067728	
RBM28	0.004067728	
PDIA4	0.004067728	
WDR7	0.004067728	
SLC4A7	0.004067728	
ERP44	0.004067728	
CGB	0.004067728	
CNOT11	0.004067728	
IGLL5	0.004067728	
GPC4	0.004067728	
CTHRC1	0.004067728	
RBM7	0.004067728	
B3GNT8	0.004016881	
KDM5C	0.004016881	

GLRX5	0.004016881	
CTF1	0.004016881	
KIF5C	0.004016881	
PHOSPHO2	0.004016881	
AC144652.1	0.004016881	
EXT2	0.004016881	
PDE8A	0.004016881	
TSEN34	0.004016881	
ENPP4	0.004016881	
CAPN6	0.004016881	
DEDD2	0.004016881	
INHBA	0.004016881	
PRKCSH	0.004016881	
UBAP1L	0.004016881	
CSF2	0.004016881	
PTPRM	0.004016881	
STC2	0.004016881	
MS4A7	0.004016881	
KLC3	0.004016881	
FBXW2	0.004016881	
GCAT	0.004016881	
PAFAH1B2	0.004016881	
TMEM9	0.004016881	
ANKRD2	0.004016881	
HOXA3	0.004016881	
GPC1	0.004016881	
GIPC2	0.004016881	
ZNF468	0.004016881	
COL4A4	0.004016881	
CCDC82	0.004016881	
CMPK1	0.004016881	
COQ9	0.004016881	
TBC1D8	0.004016881	
DEXI	0.004016881	
SLC13A3	0.004016881	
CEP19	0.004016881	
NUPL1	0.004016881	
ACAD9	0.004016881	
ZBTB47	0.004016881	
SMIM15	0.004016881	
DOCK4	0.004016881	

SCAMP4	0.004016881	
RP11-108O10.2	0.004016881	
NAA35	0.004016881	
CRHBP	0.004016881	
CYP11A1	0.004016881	
ANXA11	0.004016881	
BUB3	0.004016881	
ORMDL3	0.004016881	
KB-68A7.1	0.004016881	
POLR3E	0.004016881	
METTL5	0.004016881	
IGHG4	0.004016881	
CACNA1H	0.004016881	
DNASE2	0.004016881	
NME4	0.004016881	
PXN-AS1	0.004016881	
MANEA	0.004016881	
ARHGAP6	0.004016881	
DNAJC18	0.004016881	
CAMKK2	0.004016881	
ZNF350	0.004016881	
HPRT1	0.004016881	
GALNT3	0.004016881	
PLXNB3	0.004016881	
C6	0.004016881	
GNAZ	0.004016881	
RPL10AP6	0.004016881	
SAFB2	0.004016881	
CCDC137	0.004016881	
SLC22A18	0.004016881	
HMGN4	0.004016881	
B3GALNT2	0.004016881	
PUS10	0.004016881	
UBOX5	0.004016881	
RTN4IP1	0.004016881	
SFTPC	0.004016881	
AREG	0.004016881	
PACS2	0.004016881	
ORAOV1	0.004016881	
RP11-326I11.3	0.004016881	
MIEF1	0.004016881	

CHAC2	0.004016881	
RP11-693N9.2	0.004016881	
VAMP8	0.004016881	
UPF3B	0.004016881	
ATP1A3	0.004016881	
CTC-505O3.2	0.004016881	
ZNF623	0.004016881	
SLC4A2	0.004016881	
CTNNA3	0.004016881	
INO80C	0.004016881	
MAPK1IP1L	0.004016881	
SNRPGP2	0.004016881	
MOXD1	0.004016881	
KCNK6	0.004016881	
TXNDC12	0.004016881	
ZNF696	0.004016881	
SELM	0.004016881	
DHODH	0.004016881	
C11orf58	0.004016881	
ZNF543	0.003966034	
METTL9	0.003966034	
PLEKHM2	0.003966034	
RAB36	0.003966034	
RAB6B	0.003966034	
TMEM238	0.003966034	
MBLAC2	0.003966034	
ASNS	0.003966034	
EARS2	0.003966034	
GCLC	0.003966034	
OLFML2B	0.003966034	
WDR13	0.003966034	
GIMAP1	0.003966034	
PARN	0.003966034	
WDR44	0.003966034	
ZFAND2A	0.003966034	
ANO1	0.003966034	
SNAP47	0.003966034	
MRPL42	0.003966034	
TLCD1	0.003966034	
FCHSD2	0.003966034	
RAB8A	0.003966034	

EEF2KMT	0.003966034	
RPS6KB2	0.003966034	
STX16	0.003966034	
IFT88	0.003966034	
SHROOM4	0.003966034	
FAM122B	0.003966034	
PDE6B	0.003966034	
ILKAP	0.003966034	
ABHD11	0.003966034	
CLK4	0.003966034	
ZNF473	0.003966034	
RAP2C	0.003966034	
SLCO1A2	0.003966034	
TMEM97	0.003966034	
MYL5	0.003966034	
MFNG	0.003966034	
RNF214	0.003966034	
LRRC47	0.003966034	
SEC24D	0.003966034	
GID8	0.003966034	
CHST14	0.003966034	
SGPP1	0.003966034	
SFXN4	0.003966034	
TMEM255B	0.003966034	
ATXN2L	0.003966034	
TRPV4	0.003966034	
RP11-134G8.5	0.003966034	
RRN3P3	0.003966034	
CLPTM1	0.003966034	
TTPAL	0.003966034	
KIAA1683	0.003966034	
ZNRF1	0.003966034	
MAGT1	0.003966034	
RILP	0.003966034	
ZNF76	0.003966034	
TSEN2	0.003966034	
TYSND1	0.003966034	
MAP4K2	0.003966034	
PLEKHA6	0.003966034	
ARHGAP26	0.003966034	
RNF26	0.003966034	

SNX12	0.003966034	
CIR1	0.003966034	
MYOCD	0.003966034	
REEP1	0.003966034	
BBS10	0.003966034	
ANAPC10	0.003966034	
ZDHHC13	0.003966034	
RP4-639F20.1	0.003966034	
MOGAT3	0.003966034	
TNNI2	0.003966034	
TCEAL3	0.003966034	
ALPL	0.003966034	
COL27A1	0.003966034	
ELL	0.003966034	
SBNO1	0.003966034	
XPOT	0.003966034	
IGLV3-25	0.003966034	
LAMP1	0.003966034	
MYOM1	0.003966034	
PRRC1	0.003966034	
GIPC3	0.003966034	
ZNF853	0.003966034	
ABCG1	0.003966034	
FSD2	0.003966034	
C17orf85	0.003966034	
LEPROTL1	0.003966034	
CXCL10	0.003966034	
C7orf50	0.003966034	
SLC30A3	0.003966034	
KLHDC8B	0.003915188	
PDGFD	0.003915188	
IMPA1	0.003915188	
LAPTM4A	0.003915188	
RHBDD3	0.003915188	
FCGR3A	0.003915188	
SEMA3B	0.003915188	
AC016739.2	0.003915188	
SCAF1	0.003915188	
CDKN1C	0.003915188	
PROM2	0.003915188	
APC2	0.003915188	

HOXD12	0.003915188	
ICA1	0.003915188	
CEP72	0.003915188	
MAP3K9	0.003915188	
TIMM23B	0.003915188	
PRUNE2	0.003915188	
HOXB8	0.003915188	
CEP120	0.003915188	
TKTL2	0.003915188	
RNF103	0.003915188	
RP11-345P4.9	0.003915188	
CARKD	0.003915188	
RNF32	0.003915188	
CKB	0.003915188	
INHBB	0.003915188	
TLE6	0.003915188	
CWF19L2	0.003915188	
DUSP9	0.003915188	
IP6K1	0.003915188	
PANK1	0.003915188	
LINGO4	0.003915188	
MIER3	0.003915188	
NFKBID	0.003915188	
CTD-2562J17.6	0.003915188	
LSM14A	0.003915188	
NPM3	0.003915188	
CARF	0.003915188	
PIP4K2C	0.003915188	
FRMD3	0.003915188	
CCDC28A	0.003915188	
NLGN2	0.003915188	
LSM14B	0.003915188	
ANP32E	0.003915188	
RAD18	0.003915188	
ARMC8	0.003915188	
PIR	0.003915188	
TES	0.003915188	
HSD17B3	0.003915188	
ECD	0.003915188	
MPP7	0.003915188	
SGMS1	0.003915188	

RNASET2	0.003915188	
DGKQ	0.003915188	
MYO7A	0.003915188	
PLGLB1	0.003915188	
CXXC5	0.003915188	
CD28	0.003915188	
PTPN18	0.003915188	
H2AFY2	0.003915188	
BNIP2	0.003915188	
HTRA3	0.003915188	
TGM1	0.003915188	
GLCE	0.003915188	
ADD3	0.003915188	
RNASEH2B	0.003915188	
LIMD1	0.003915188	
CBX7	0.003915188	
OSER1	0.003915188	
FGFRL1	0.003915188	
UGT1A2P	0.003915188	
LINC01003	0.003915188	
HPS1	0.003915188	
SP140	0.003915188	
RAB18	0.003915188	
CREG1	0.003915188	
TMEM14B	0.003915188	
RSRC2	0.003915188	
SPRED2	0.003915188	
IL13	0.003915188	
SLC35B2	0.003915188	
PALM3	0.003915188	
NINJ1	0.003915188	
IDH2	0.003915188	
CLUH	0.003915188	
EML2	0.003915188	
ZNF414	0.003915188	
ZNF329	0.003915188	
MUC13	0.003915188	
NEK8	0.003915188	
UBE2DNL	0.003915188	
CLEC1A	0.003915188	
ELMO1	0.003915188	

CX3CL1	0.003915188	
CRB3	0.003915188	
FAM118B	0.003915188	
TMEM2	0.003915188	
OXSM	0.003915188	
NUP155	0.003915188	
LYSMD1	0.003915188	
SRRD	0.003915188	
R3HDM1	0.003915188	
ENPP3	0.003864341	
CLEC14A	0.003864341	
RPL23AP82	0.003864341	
RDH10	0.003864341	
CEP152	0.003864341	
BET1	0.003864341	
GLOD4	0.003864341	
TTC19	0.003864341	
RHNO1	0.003864341	
HOGA1	0.003864341	
CSTB	0.003864341	
SLC20A1	0.003864341	
FTSJ3	0.003864341	
BTBD3	0.003864341	
FBXL4	0.003864341	
RUNDC1	0.003864341	
S100PBP	0.003864341	
NOS2	0.003864341	
RUFY1	0.003864341	
HBS1L	0.003864341	
CLIP4	0.003864341	
HIAT1	0.003864341	
RNF139	0.003864341	
MAP3K13	0.003864341	
P2RY2	0.003864341	
LINC01093	0.003864341	
SUSD6	0.003864341	
C14orf80	0.003864341	
FAM171A1	0.003864341	
PLEKHB1	0.003864341	
LIN7C	0.003864341	
TNFAIP1	0.003864341	

F2RL1	0.003864341	
LRRC2	0.003864341	
CHRAC1	0.003864341	
KLC2	0.003864341	
CTD-3184A7.4	0.003864341	
RAB35	0.003864341	
OGN	0.003864341	
GINS4	0.003864341	
ATP10A	0.003864341	
POLR3GL	0.003864341	
SLC25A24	0.003864341	
GCGR	0.003864341	
FZD9	0.003864341	
DHX34	0.003864341	
COA6	0.003864341	
RNF187	0.003864341	
EOGT	0.003864341	
KIF1A	0.003864341	
SPATA5L1	0.003864341	
TAB3	0.003864341	
ZNF582	0.003864341	
LINC00242	0.003864341	
USP48	0.003864341	
RPAP1	0.003864341	
CABIN1	0.003864341	
CALCRL	0.003864341	
CSPG4	0.003864341	
EEF1A1P11	0.003864341	
OSBPL2	0.003864341	
AMPD2	0.003864341	
ATP6V0E2	0.003864341	
COMMD2	0.003864341	
TMTC2	0.003864341	
GARS	0.003864341	
XPR1	0.003864341	
MKRN2	0.003864341	
CTDSP1	0.003864341	
GUSB	0.003864341	
SLC25A33	0.003864341	
RP11-326C3.2	0.003864341	
PLOD1	0.003864341	

PDIK1L	0.003864341	
PAM	0.003864341	
KIFAP3	0.003864341	
CD55	0.003864341	
NSUN2	0.003864341	
LSM6	0.003864341	
CSTF2	0.003864341	
PTGIS	0.003864341	
METRN	0.003864341	
SERP1	0.003864341	
PHACTR1	0.003864341	
ANAPC4	0.003864341	
DDX58	0.003864341	
AGTPBP1	0.003864341	
HLA-H	0.003864341	
IL23A	0.003864341	
LILRB4	0.003864341	
RP11-1094M14.11	0.003864341	
RP11-408P14.1	0.003864341	
LSP1	0.003864341	
FNBP1L	0.003864341	
PDZK1IP1	0.003864341	
GJA5	0.003864341	
ZNF385B	0.003864341	
FDXR	0.003864341	
SMEK2	0.003864341	
SLC46A3	0.003864341	
POP1	0.003864341	
FZD7	0.003864341	
AZGP1P1	0.003864341	
ATRIP	0.003864341	
CCDC9	0.003864341	
TBX21	0.003864341	
ZC3H15	0.003864341	
RAB14	0.003864341	
AKR1C2	0.003864341	
LTA	0.003864341	
FABP4	0.003813495	
IDS	0.003813495	
CTB-89H12.4	0.003813495	
FPGS	0.003813495	

DHRS1	0.003813495	
BTN3A2	0.003813495	
GPT	0.003813495	
UBE4A	0.003813495	
PPP3CB-AS1	0.003813495	
CYP21A2	0.003813495	
KRT16	0.003813495	
FNBP1	0.003813495	
KALRN	0.003813495	
TUBE1	0.003813495	
CXorf56	0.003813495	
GRAMD3	0.003813495	
VSTM4	0.003813495	
ATP5F1	0.003813495	
SLC35F2	0.003813495	
TBC1D24	0.003813495	
TRPC5	0.003813495	
SRD5A2	0.003813495	
PLAA	0.003813495	
FBXO10	0.003813495	
PRR34-AS1	0.003813495	
ARL6	0.003813495	
ENTPD6	0.003813495	
DCLRE1A	0.003813495	
C10orf88	0.003813495	
EXOSC9	0.003813495	
MYO5B	0.003813495	
SLC25A46	0.003813495	
SPSB2	0.003813495	
XRCC4	0.003813495	
TMEM128	0.003813495	
GAPVD1	0.003813495	
SCAP	0.003813495	
TCAIM	0.003813495	
ZYG11B	0.003813495	
PQLC2	0.003813495	
ZNF300	0.003813495	
GUCY2C	0.003813495	
WNT3A	0.003813495	
SIRPA	0.003813495	
HIST1H4K	0.003813495	

FAM185A	0.003813495	
IGLV6-57	0.003813495	
FLJ37453	0.003813495	
AP3S1	0.003813495	
KIAA1033	0.003813495	
TRMT1L	0.003813495	
THAP6	0.003813495	
NAA50	0.003813495	
SACM1L	0.003813495	
LARP1	0.003813495	
SLC37A3	0.003813495	
PPP2R5B	0.003813495	
KIAA0907	0.003813495	
GALNT16	0.003813495	
RFK	0.003813495	
MICU2	0.003813495	
RIPK4	0.003813495	
GPRIN3	0.003813495	
SRGAP3	0.003813495	
SACS	0.003813495	
RN7SK	0.003813495	
RNF10	0.003813495	
ANAPC7	0.003813495	
FAM220A	0.003813495	
YPEL2	0.003813495	
UBAC1	0.003813495	
SRGAP2C	0.003813495	
DYRK2	0.003813495	
DNAL4	0.003813495	
EVL	0.003813495	
UBBP4	0.003813495	
CCDC109B	0.003813495	
WDR37	0.003813495	
PI4KA	0.003813495	
NCLN	0.003813495	
PSPH	0.003813495	
GRHL3	0.003813495	
DNAJC11	0.003813495	
CCM2L	0.003813495	
PLCB4	0.003813495	
CDR2L	0.003813495	

PLCD1	0.003813495	
CHST2	0.003762648	
HILPDA	0.003762648	
PAPPA	0.003762648	
NLRC3	0.003762648	
TPD52L1	0.003762648	
BLVRA	0.003762648	
43709	0.003762648	
MMP13	0.003762648	
NDUFAF1	0.003762648	
TMEM194A	0.003762648	
RPL41P5	0.003762648	
ZCCHC7	0.003762648	
IBSP	0.003762648	
SCAF4	0.003762648	
LMAN1	0.003762648	
STMN2	0.003762648	
MYL12A	0.003762648	
ISL2	0.003762648	
HEMK1	0.003762648	
SBDSP1	0.003762648	
OSM	0.003762648	
VWF	0.003762648	
ADORA1	0.003762648	
VAMP7	0.003762648	
KLF7	0.003762648	
CYB5B	0.003762648	
TICAM2	0.003762648	
DDX49	0.003762648	
RAI14	0.003762648	
SLC44A3	0.003762648	
CDC42SE1	0.003762648	
AIFM1	0.003762648	
SMIM8	0.003762648	
CHMP2B	0.003762648	
SCN1B	0.003762648	
MEF2BNB	0.003762648	
SLC46A1	0.003762648	
FPR1	0.003762648	
MFGE8	0.003762648	
COG7	0.003762648	

MRPS36	0.003762648	
TUBA3D	0.003762648	
CTC-297N7.9	0.003762648	
GTPBP3	0.003762648	
TUBG2	0.003762648	
NMT2	0.003762648	
LNPEP	0.003762648	
STEAP1	0.003762648	
HULC	0.003762648	
KIAA1958	0.003762648	
TPP2	0.003762648	
PIGQ	0.003762648	
HLA-DQB2	0.003762648	
ROPN1	0.003762648	
C4orf46	0.003762648	
CD46	0.003762648	
CXCL9	0.003762648	
CTSC	0.003762648	
RAB43	0.003762648	
ZNF200	0.003762648	
ZCWPW1	0.003762648	
USP5	0.003762648	
KCTD15	0.003762648	
UGT1A4	0.003762648	
TMEM106B	0.003762648	
MGAT4A	0.003762648	
TSEN15	0.003762648	
HIST1H2BE	0.003762648	
MYH7	0.003762648	
RALGPS2	0.003762648	
YKT6	0.003762648	
CKS2	0.003762648	
PAOX	0.003762648	
C9orf114	0.003762648	
GREB1L	0.003762648	
PARP14	0.003762648	
COL11A2	0.003762648	
ZNF681	0.003762648	
GPD1	0.003762648	
WDR26	0.003762648	
DAGLB	0.003762648	

TOMM70A	0.003762648	
ZNF503-AS2	0.003762648	
CXCR6	0.003762648	
OTUD6B-AS1	0.003762648	
HAND2-AS1	0.003762648	
PSTK	0.003762648	
YARS	0.003762648	
RUSC1	0.003762648	
SNX6	0.003762648	
CTD-2192J16.15	0.003762648	
FBXW9	0.003762648	
ANKRD1	0.003762648	
CGRRF1	0.003762648	
ZCCHC16	0.003762648	
AGPS	0.003762648	
KLF8	0.003762648	
TRIM4	0.003762648	
PIM2	0.003762648	
SOGA1	0.003762648	
DRG2	0.003762648	
PF4	0.003762648	
SCAMP2	0.003762648	
BMF	0.003711801	
BRAP	0.003711801	
RPL6P27	0.003711801	
SH2D3A	0.003711801	
ADARB1	0.003711801	
CCBE1	0.003711801	
PDZD11	0.003711801	
KCTD7	0.003711801	
CDH23	0.003711801	
UBXN10	0.003711801	
MID1IP1	0.003711801	
ASPN	0.003711801	
DLG5	0.003711801	
ZNF773	0.003711801	
RSPH3	0.003711801	
SEC14L3	0.003711801	
GNGT1	0.003711801	
MMAB	0.003711801	
BTN3A1	0.003711801	

UBALD1	0.003711801	
RNF167	0.003711801	
TNFAIP8L1	0.003711801	
NKRF	0.003711801	
AAMP	0.003711801	
TANC2	0.003711801	
ZNF692	0.003711801	
ELP5	0.003711801	
STAU2	0.003711801	
UVSSA	0.003711801	
FAM53C	0.003711801	
RP11-158I9.8	0.003711801	
PTPRH	0.003711801	
RP11-422N16.3	0.003711801	
CISD2	0.003711801	
ZNF691	0.003711801	
ELOVL6	0.003711801	
ZXDC	0.003711801	
DNAJB5	0.003711801	
HIST1H2BK	0.003711801	
PAIP2	0.003711801	
TBC1D16	0.003711801	
PTGFR	0.003711801	
C21orf2	0.003711801	
PIGK	0.003711801	
RERG	0.003711801	
C16orf52	0.003711801	
CASD1	0.003711801	
ZSCAN29	0.003711801	
NUS1	0.003711801	
ANXA13	0.003711801	
POLB	0.003711801	
L3MBTL3	0.003711801	
RPF1	0.003711801	
NOMO1	0.003711801	
FAM118A	0.003711801	
PPTC7	0.003711801	
C15orf61	0.003711801	
CCNB1IP1	0.003711801	
TMEM160	0.003711801	
NUFIP1	0.003711801	

SFT2D1	0.003711801	
CETN3	0.003711801	
LINC01124	0.003711801	
ALKBH3	0.003711801	
GS1-124K5.12	0.003711801	
DEGS1	0.003711801	
CCAR2	0.003711801	
TSPAN18	0.003711801	
RNF6	0.003711801	
SIGIRR	0.003711801	
ANKRD6	0.003711801	
TACC2	0.003711801	
HACL1	0.003711801	
FAIM3	0.003711801	
EDARADD	0.003711801	
UBL7-AS1	0.003711801	
SNUPN	0.003711801	
PIP4K2B	0.003711801	
MAN1C1	0.003711801	
RBM47	0.003711801	
CMC1	0.003711801	
RUNDC3A	0.003711801	
ST3GAL4-AS1	0.003711801	
C10orf10	0.003711801	
ZNF608	0.003711801	
INMT	0.003711801	
UTP18	0.003711801	
PIGU	0.003711801	
ZFP14	0.003711801	
DOCK10	0.003711801	
KIAA0391	0.003660955	
ELP6	0.003660955	
RGL3	0.003660955	
C7	0.003660955	
PKDCC	0.003660955	
RAB26	0.003660955	
PTPRA	0.003660955	
AB019441.29	0.003660955	
CD79B	0.003660955	
ODF3B	0.003660955	
CDH11	0.003660955	

DCAF8	0.003660955	
SPRN	0.003660955	
S100A2	0.003660955	
MYO6	0.003660955	
APOL1	0.003660955	
FECH	0.003660955	
RIN2	0.003660955	
DHX33	0.003660955	
AFTPH	0.003660955	
ABHD12	0.003660955	
HEIH	0.003660955	
UGT1A10	0.003660955	
SLC19A3	0.003660955	
FAM76A	0.003660955	
PPP1R11	0.003660955	
DCLRE1C	0.003660955	
AUH	0.003660955	
STRBP	0.003660955	
C12orf75	0.003660955	
BTN2A1	0.003660955	
CTD-2350C19.2	0.003660955	
SURF1	0.003660955	
DROSHA	0.003660955	
ITGB1BP1	0.003660955	
TMCC3	0.003660955	
INTS7	0.003660955	
CABLES2	0.003660955	
ADSS	0.003660955	
C2orf76	0.003660955	
ZG16	0.003660955	
IFT140	0.003660955	
OSGEPL1	0.003660955	
VPS37A	0.003660955	
SPON2	0.003660955	
LEO1	0.003660955	
LMX1A	0.003660955	
MUC20	0.003660955	
GTF2H5	0.003660955	
KIAA1671	0.003660955	
BMP7	0.003660955	
C10orf12	0.003660955	

GAS6	0.003660955	
ZNF189	0.003660955	
CYYR1	0.003660955	
TMEM65	0.003660955	
IGLV3-19	0.003660955	
C1orf112	0.003660955	
TDRD7	0.003660955	
SRR	0.003660955	
SFTPD	0.003660955	
PIP	0.003660955	
TMEM9B	0.003660955	
DGKE	0.003660955	
TJP2	0.003660955	
GFI1	0.003660955	
KIAA1598	0.003660955	
CLIC5	0.003660955	
FBXO17	0.003660955	
HAUS2	0.003660955	
SIM1	0.003660955	
FCN1	0.003660955	
RP11-539L10.3	0.003660955	
C19orf68	0.003660955	
H1FX	0.003660955	
AMOTL1	0.003660955	
PGM5	0.003660955	
DBI	0.003660955	
FERMT2	0.003660955	
MOB3B	0.003660955	
CBR4	0.003660955	
SLC2A4RG	0.003660955	
PSMD5	0.003660955	
PGLYRP2	0.003660955	
SALL2	0.003660955	
ADORA2BP1	0.003660955	
RANBP3	0.003660955	
SLC35B1	0.003660955	
ZFAND3	0.003660955	
PRDM2	0.003660955	
PLA2G2A	0.003660955	
DHRS2	0.003660955	
KIF9	0.003660955	

LRRC3	0.003660955	
TUFT1	0.003660955	
INTS9	0.003660955	
TRIM5	0.003660955	
GOLGA3	0.003660955	
PTPRJ	0.003660955	
INSIG1	0.003660955	
ZNF101	0.003660955	
HIST1H4J	0.003660955	
FAAH	0.003660955	
GYPB	0.003610108	
ADRA1B	0.003610108	
CTLA4	0.003610108	
MEOX1	0.003610108	
APOL6	0.003610108	
RP11-449P15.2	0.003610108	
PTPN23	0.003610108	
C1orf226	0.003610108	
NVL	0.003610108	
MYCT1	0.003610108	
MEX3A	0.003610108	
TYW5	0.003610108	
COG2	0.003610108	
SLC16A1-AS1	0.003610108	
FZD3	0.003610108	
CLEC2D	0.003610108	
PEG3	0.003610108	
RBPJL	0.003610108	
C17orf58	0.003610108	
NT5DC2	0.003610108	
ATG16L1	0.003610108	
ZNF48	0.003610108	
RP3-512B11.3	0.003610108	
DPH2	0.003610108	
LYZ	0.003610108	
RHEBL1	0.003610108	
PKHD1	0.003610108	
SCRN1	0.003610108	
SPDEF	0.003610108	
FAM160A2	0.003610108	
EFNA4	0.003610108	

C9orf152	0.003610108	
RP11-1148L6.8	0.003610108	
ZNF274	0.003610108	
TRIAP1	0.003610108	
LAT	0.003610108	
MTUS1	0.003610108	
MAPRE2	0.003610108	
43716	0.003610108	
TM2D3	0.003610108	
CIAPIN1	0.003610108	
RPL14P1	0.003610108	
ACO1	0.003610108	
AKR1B15	0.003610108	
BCKDK	0.003610108	
RHO	0.003610108	
B3GNT2	0.003610108	
CTB-58E17.1	0.003610108	
OIT3	0.003610108	
MAFA	0.003610108	
MBOAT1	0.003610108	
FKBP10	0.003610108	
TRNAU1AP	0.003610108	
ME1	0.003610108	
IL12A	0.003610108	
TPRKB	0.003610108	
KSR1	0.003610108	
DRD1	0.003610108	
NIPSNAP3A	0.003610108	
KANK3	0.003610108	
MRPL19	0.003610108	
ENPEP	0.003610108	
CH507-9B2.3	0.003610108	
SEMA4C	0.003610108	
KB-1507C5.2	0.003610108	
LINC01485	0.003610108	
SENP7	0.003610108	
EYA1	0.003610108	
PPWD1	0.003610108	
TARS2	0.003610108	
TMEM68	0.003610108	
MESDC2	0.003610108	

CCNYL1	0.003610108	
PMPCB	0.003610108	
SESN1	0.003610108	
MAPKAP1	0.003610108	
BLK	0.003610108	
ESF1	0.003610108	
TRIM26	0.003610108	
ZNF850	0.003610108	
ZNF574	0.003610108	
CAMTA2	0.003610108	
PCIF1	0.003610108	
AKAP17A	0.003610108	
YAE1D1	0.003610108	
CXCL14	0.003610108	
PDK3	0.003610108	
GNAL	0.003610108	
S100A10	0.003610108	
F2RL2	0.003610108	
DENND4A	0.003610108	
CBLC	0.003610108	
AKAP12	0.003610108	
LMOD1	0.003610108	
CRABP2	0.003610108	
COL4A5	0.003610108	
THADA	0.003610108	
TNNC1	0.003610108	
C6orf226	0.003610108	
RABGGTA	0.003610108	
FOXRED1	0.003610108	
KCNJ11	0.003610108	
UBTD1	0.003610108	
MPV17L2	0.003610108	
RTKN	0.003610108	
CRLF1	0.003610108	
MOAP1	0.003610108	
CPQ	0.003610108	
ZBED5	0.003610108	
NSUN6	0.003610108	
NSA2	0.003610108	
IL34	0.003610108	
CAMK2N1	0.003610108	

NIPA1	0.003610108	
ADAL	0.003610108	
TCN2	0.003610108	
UAP1L1	0.003610108	
ZNF791	0.003559262	
MFI2	0.003559262	
ARL6IP6	0.003559262	
RP11-505K9.1	0.003559262	
FAM57A	0.003559262	
KCNH2	0.003559262	
YTHDF3	0.003559262	
N4BP3	0.003559262	
SRD5A1	0.003559262	
FGF13	0.003559262	
NEURL4	0.003559262	
DDX52	0.003559262	
NUP54	0.003559262	
FANCF	0.003559262	
SMIM1	0.003559262	
ADAT2	0.003559262	
HOXD3	0.003559262	
THOC3	0.003559262	
TNFRSF8	0.003559262	
APOA4	0.003559262	
RHOH	0.003559262	
C12orf45	0.003559262	
IKZF2	0.003559262	
APBB3	0.003559262	
TESC	0.003559262	
THYN1	0.003559262	
DUS3L	0.003559262	
HOXA2	0.003559262	
CUL7	0.003559262	
ANKRD12	0.003559262	
GPKOW	0.003559262	
TMTC4	0.003559262	
RBM14	0.003559262	
IFNGR2	0.003559262	
ZNF667	0.003559262	
BMP6	0.003559262	
DSG2	0.003559262	

FAM90A1	0.003559262	
LSS	0.003559262	
JMJD6	0.003559262	
KLHL21	0.003559262	
TRIM13	0.003559262	
ALG3	0.003559262	
SUPV3L1	0.003559262	
ALG1	0.003559262	
ZZEF1	0.003559262	
TMEM69	0.003559262	
KIAA0319L	0.003559262	
ANKRD54	0.003559262	
CTNND2	0.003559262	
BTN1A1	0.003559262	
HSD17B13	0.003559262	
PRTFDC1	0.003559262	
AC016700.5	0.003559262	
LMF1	0.003559262	
KCTD11	0.003559262	
FAT1	0.003559262	
PAXIP1-AS2	0.003559262	
ZNF827	0.003559262	
ZFP36L1	0.003559262	
COQ3	0.003559262	
CISH	0.003559262	
LAMA2	0.003559262	
FA2H	0.003559262	
FAM213A	0.003559262	
STX1B	0.003559262	
AGAP6	0.003559262	
DENND6B	0.003559262	
PTPN14	0.003559262	
MPZL3	0.003559262	
ENPP2	0.003559262	
ZNF282	0.003559262	
TAP2	0.003559262	
RRP9	0.003559262	
TRMT61B	0.003559262	
RPL24P4	0.003559262	
ASMTL	0.003559262	
METTL20	0.003559262	

PARL	0.003559262	
LIG4	0.003559262	
GPRIN1	0.003559262	
U1	0.003559262	
AKIRIN2	0.003559262	
MRTO4	0.003559262	
C8orf49	0.003559262	
SLC7A1	0.003559262	
HYAL3	0.003559262	
RP11-452I5.2	0.003559262	
RRNAD1	0.003559262	
HSD3B7	0.003559262	
DDIT4	0.003559262	
BCAN	0.003559262	
IL12RB2	0.003559262	
TPBG	0.003559262	
ROBO2	0.003559262	
MCFD2	0.003559262	
ATP11C	0.003559262	
HNRNPA1P48	0.003559262	
FAM124B	0.003559262	
IFT172	0.003559262	
CEP104	0.003559262	
FRMD8	0.003559262	
IRF2BP2	0.003559262	
PHYHIPL	0.003559262	
SYDE1	0.003559262	
FXYD1	0.003559262	
DENND5A	0.003559262	
SIX2	0.003559262	
LRFN3	0.003559262	
SLC38A1	0.003559262	
CSAD	0.003559262	
RNF138	0.003559262	
LZIC	0.003559262	
RTN4R	0.003559262	
CTD-3252C9.4	0.003559262	
ARHGAP4	0.003559262	
AKAP10	0.003559262	
AP5Z1	0.003559262	
RYR2	0.003559262	

IFRD2	0.003559262	
MTERF3	0.003559262	
VAMP4	0.003508415	
AF127936.9	0.003508415	
MRPS30	0.003508415	
IL3RA	0.003508415	
ZNF813	0.003508415	
UGP2	0.003508415	
KCTD18	0.003508415	
ZMYM1	0.003508415	
FUT4	0.003508415	
SEMA3C	0.003508415	
CALU	0.003508415	
CTD-2186M15.3	0.003508415	
MRPL38	0.003508415	
JOSD1	0.003508415	
CASC4	0.003508415	
NEFM	0.003508415	
FAM120AOS	0.003508415	
PPCDC	0.003508415	
HPS5	0.003508415	
SYTL3	0.003508415	
PPIF	0.003508415	
CCDC85C	0.003508415	
MMGT1	0.003508415	
MRC2	0.003508415	
ZMYND8	0.003508415	
HTR2B	0.003508415	
NPPB	0.003508415	
DNASE1	0.003508415	
GFPT1	0.003508415	
TUBGCP6	0.003508415	
TTC23	0.003508415	
PFKFB4	0.003508415	
KBTBD6	0.003508415	
RP11-389C8.2	0.003508415	
KLHL13	0.003508415	
SCAMP1	0.003508415	
LACTB	0.003508415	
C17orf67	0.003508415	
WFDC1	0.003508415	

TTYH2	0.003508415	
VPS41	0.003508415	
SMCR8	0.003508415	
DLAT	0.003508415	
PTPRN	0.003508415	
ATG7	0.003508415	
NDUFAF4	0.003508415	
FMO3	0.003508415	
MFN1	0.003508415	
GDE1	0.003508415	
NOL8	0.003508415	
PIGM	0.003508415	
MAU2	0.003508415	
SARS	0.003508415	
STXBP1	0.003508415	
SLC29A2	0.003508415	
RTN4RL1	0.003508415	
USP18	0.003508415	
TBL1XR1	0.003508415	
NRCAM	0.003508415	
RAB5C	0.003508415	
PEX7	0.003508415	
CDK18	0.003508415	
GRPEL1	0.003508415	
MTX2	0.003508415	
PLCD4	0.003508415	
RP11-458F8.4	0.003508415	
ARL5A	0.003508415	
GFM1	0.003508415	
SLC27A4	0.003508415	
C4orf19	0.003508415	
ATP2B2	0.003508415	
ZNF317	0.003508415	
GNL2	0.003508415	
CXorf36	0.003508415	
TJP3	0.003508415	
ORAI3	0.003508415	
ERP29	0.003508415	
RNF8	0.003508415	
RP11-38L15.8	0.003508415	
ECSCR	0.003508415	

PLK2	0.003508415	
MYADM	0.003508415	
NAIF1	0.003508415	
HS1BP3-IT1	0.003508415	
MIB2	0.003508415	
R3HCC1	0.003508415	
ITPKA	0.003508415	
DLX4	0.003508415	
NRIP2	0.003508415	
ZBTB2	0.003508415	
AGPAT6	0.003508415	
IRAK4	0.003508415	
SAMD9	0.003508415	
IRF2BPL	0.003508415	
SULT2B1	0.003508415	
REPS1	0.003508415	
SLAIN2	0.003508415	
ROBO1	0.003508415	
TEP1	0.003508415	
SLC5A11	0.003508415	
SMPD1	0.003508415	
SDK1	0.003508415	
CSRP3	0.003508415	
CTD-2619J13.8	0.003508415	
SLC17A2	0.003508415	
ASB13	0.003508415	
ZFP28	0.003508415	
SLC2A8	0.003508415	
RND2	0.003508415	
BRAT1	0.003508415	
CKAP2	0.003508415	
PIGF	0.003508415	
BAG3	0.003508415	
C19orf80	0.003508415	
RP11-543P15.1	0.003508415	
SEMA6A	0.003508415	
TYMSOS	0.003508415	
CHL1	0.003508415	
MAL	0.003508415	
HPS4	0.003508415	
TBC1D10A	0.003508415	

TTI1	0.003508415	
DGKG	0.003508415	
SEMA3F	0.003457569	
PPP2R5E	0.003457569	
CFI	0.003457569	
GPR125	0.003457569	
GJB2	0.003457569	
C10orf76	0.003457569	
KAL1	0.003457569	
NFX1	0.003457569	
NEK7	0.003457569	
CGNL1	0.003457569	
PNMAL1	0.003457569	
PPP2R3B	0.003457569	
ZNF248	0.003457569	
PIGL	0.003457569	
ALG13	0.003457569	
MFSD11	0.003457569	
TRIM3	0.003457569	
LENG9	0.003457569	
CDH17	0.003457569	
PCYOX1	0.003457569	
C2CD4A	0.003457569	
UGT1A8	0.003457569	
STAR	0.003457569	
WDR6	0.003457569	
TGIF2	0.003457569	
CLYBL	0.003457569	
CXCL2	0.003457569	
SQLE	0.003457569	
WBP2	0.003457569	
PI4KB	0.003457569	
CBLN1	0.003457569	
NAT2	0.003457569	
PDZD4	0.003457569	
LRRC8D	0.003457569	
BPNT1	0.003457569	
WBSCR27	0.003457569	
TRAM2	0.003457569	
RILPL1	0.003457569	
DDX47	0.003457569	

CNDP2	0.003457569	
CADPS	0.003457569	
ZCCHC6	0.003457569	
ALKBH5	0.003457569	
TNFRSF25	0.003457569	
NTS	0.003457569	
SLC39A13	0.003457569	
TMEM70	0.003457569	
VPS29	0.003457569	
AP3S2	0.003457569	
TTC22	0.003457569	
TMC6	0.003457569	
C6orf165	0.003457569	
RP3-420J14.1	0.003457569	
MARS2	0.003457569	
GMPS	0.003457569	
RP11-872D17.8	0.003457569	
CCDC97	0.003457569	
OTUD7B	0.003457569	
WBSCR16	0.003457569	
GTPBP2	0.003457569	
USP31	0.003457569	
RP13-1032I1.7	0.003457569	
RP11-299G20.2	0.003457569	
ATP6V0A1	0.003457569	
ZNF768	0.003457569	
MYO5A	0.003457569	
ALS2CL	0.003457569	
PCDHB7	0.003457569	
MGAT4B	0.003457569	
RPGR	0.003457569	
TRIM8	0.003457569	
FLVCR1	0.003457569	
GALK1	0.003457569	
TMEM205	0.003457569	
MBLAC1	0.003457569	
ZNF862	0.003457569	
IQCB1	0.003457569	
TOB2	0.003457569	
NEURL3	0.003457569	
TMCO6	0.003457569	

ASNSD1	0.003457569	
POSTN	0.003457569	
INTS12	0.003457569	
FBXW4	0.003457569	
LCORL	0.003457569	
PUS3	0.003457569	
RP11-274B21.3	0.003457569	
SORT1	0.003457569	
WNT7B	0.003457569	
CAV2	0.003457569	
SEC14L1	0.003457569	
ERI2	0.003457569	
SNHG8	0.003457569	
LPCAT1	0.003457569	
ZNF410	0.003457569	
DYRK3	0.003457569	
ATP6V1B2	0.003457569	
GDF15	0.003457569	
CA8	0.003457569	
SCRN3	0.003457569	
NDOR1	0.003457569	
ITM2B	0.003457569	
ZFYVE19	0.003457569	
GDNF	0.003457569	
CCDC102A	0.003457569	
SIRT7	0.003457569	
ENDOV	0.003457569	
CLDN4	0.003457569	
ACTL6B	0.003457569	
SLC38A11	0.003457569	
METAP2	0.003457569	
MTHFR	0.003457569	
NEIL2	0.003457569	
BCHE	0.003457569	
C1orf74	0.003457569	
FAM198B	0.003457569	
CYP2U1	0.003457569	
PCCB	0.003457569	
APBA3	0.003457569	
VWA8	0.003457569	
ADCY9	0.003457569	

LSM11	0.003457569	
SGK3	0.003457569	
PCSK9	0.003457569	
SLC10A7	0.003457569	
SLC23A2	0.003406722	
LRRC4	0.003406722	
EAPP	0.003406722	
TMCC2	0.003406722	
EIF2S3	0.003406722	
EGLN1	0.003406722	
TRAPPC4	0.003406722	
SH3PXD2A	0.003406722	
FCF1	0.003406722	
ZNF772	0.003406722	
PDE12	0.003406722	
SECISBP2	0.003406722	
ABCD1	0.003406722	
TTC9C	0.003406722	
ANXA3	0.003406722	
PARP9	0.003406722	
DNAJC1	0.003406722	
HIST3H2A	0.003406722	
SLC2A6	0.003406722	
AP5M1	0.003406722	
RP11-355B11.2	0.003406722	
PDLIM2	0.003406722	
SETDB2	0.003406722	
BMP2K	0.003406722	
LINC01420	0.003406722	
MCCC2	0.003406722	
HOXC13	0.003406722	
SLC23A1	0.003406722	
NPRL3	0.003406722	
RP11-284F21.10	0.003406722	
EMC1	0.003406722	
CNKSR2	0.003406722	
ZNF490	0.003406722	
DCAF10	0.003406722	
PPP1R3D	0.003406722	
AC093838.4	0.003406722	
MCU	0.003406722	

DNAJC13	0.003406722	
GPR107	0.003406722	
EN2	0.003406722	
PURB	0.003406722	
HECTD2	0.003406722	
R3HDM2	0.003406722	
CEP85	0.003406722	
SLC5A1	0.003406722	
AHNAK	0.003406722	
ZNF160	0.003406722	
PLXNA4	0.003406722	
CDC42BPG	0.003406722	
HNRNPAB	0.003406722	
TMEM164	0.003406722	
RPS6KA6	0.003406722	
SLC25A36	0.003406722	
SLC16A6	0.003406722	
FAM134A	0.003406722	
ERLEC1	0.003406722	
FRG1B	0.003406722	
OAZ3	0.003406722	
PHLDA1	0.003406722	
HIST1H4E	0.003406722	
TAF4B	0.003406722	
CLIC2	0.003406722	
ZNF667-AS1	0.003406722	
CDK17	0.003406722	
CFP	0.003406722	
SLC30A6	0.003406722	
MICAL2	0.003406722	
ARFIP2	0.003406722	
PSMD9	0.003406722	
CARD10	0.003406722	
ANK1	0.003406722	
FAM103A1	0.003406722	
SAMD1	0.003406722	
CPNE7	0.003406722	
SEH1L	0.003406722	
NHSL1	0.003406722	
TMX1	0.003406722	
TTLL1	0.003406722	

NPEPPS	0.003406722	
AQP2	0.003406722	
RGS18	0.003406722	
ARID5A	0.003406722	
GBP2	0.003406722	
C1orf168	0.003406722	
EBF3	0.003406722	
TBCE	0.003406722	
APBB1IP	0.003406722	
RAB30	0.003406722	
ZNF618	0.003406722	
SLC35C1	0.003406722	
ZNF653	0.003406722	
NKG7	0.003406722	
RASGRP4	0.003406722	
SEPW1	0.003406722	
STX2	0.003406722	
SOCS4	0.003406722	
TMEM218	0.003406722	
PCED1B	0.003406722	
43717	0.003406722	
ZSWIM8	0.003406722	
KLRK1	0.003406722	
PREPL	0.003406722	
CDH15	0.003406722	
PFAS	0.003406722	
CCR10	0.003406722	
TPI1P2	0.003406722	
P2RX4	0.003406722	
RAPGEF3	0.003406722	
NRXN2	0.003406722	
ZFYVE16	0.003406722	
LY6E	0.003406722	
NUDT2	0.003406722	
NDUFAF6	0.003406722	
AGO3	0.003406722	
LONRF2	0.003406722	
SAMM50	0.003406722	
GGT1	0.003406722	
RABEPK	0.003406722	
RNF180	0.003406722	

SEC11A	0.003406722	
ATP6AP2	0.003406722	
VPS9D1	0.003406722	
AMIGO3	0.003406722	
LRRC29	0.003406722	
LINC00526	0.003406722	
PPP4R4	0.003406722	
ZCCHC17	0.003406722	
TIMM44	0.003406722	
CRYZL1	0.003406722	
ARL8B	0.003406722	
EEFSEC	0.003406722	
NCOA5	0.003406722	
ENDOG	0.003406722	
ZNF20	0.003406722	
PLXDC2	0.003406722	
ZNF493	0.003406722	
PMCH	0.003406722	
LRIG2	0.003406722	
SEZ6L2	0.003406722	
SUPT7L	0.003406722	
MLXIP	0.003355875	
CFHR1	0.003355875	
CASQ2	0.003355875	
GOLGA7B	0.003355875	
IL11RA	0.003355875	
ABHD17B	0.003355875	
GJC1	0.003355875	
ZNF382	0.003355875	
RPGRIP1	0.003355875	
DERA	0.003355875	
TMEM189	0.003355875	
CNIH4	0.003355875	
ZNF703	0.003355875	
EFNA5	0.003355875	
ADRA2C	0.003355875	
PLBD2	0.003355875	
RP4-665J23.1	0.003355875	
KLHL23	0.003355875	
AVIL	0.003355875	
XXbac-BPG283O16.9	0.003355875	

FTO	0.003355875	
TOP3B	0.003355875	
C1orf228	0.003355875	
MFN2	0.003355875	
ACTR5	0.003355875	
SOAT1	0.003355875	
PGD	0.003355875	
RRM2B	0.003355875	
RP11-423H2.1	0.003355875	
IRAK2	0.003355875	
GPR108	0.003355875	
SRRM3	0.003355875	
TBL2	0.003355875	
NUDT22	0.003355875	
MMP19	0.003355875	
SNHG14	0.003355875	
AP1G2	0.003355875	
CXCL6	0.003355875	
DNAAF5	0.003355875	
CTSO	0.003355875	
IL1RL1	0.003355875	
ELF5	0.003355875	
FXN	0.003355875	
SLC19A2	0.003355875	
FCN3	0.003355875	
NOXA1	0.003355875	
C2orf72	0.003355875	
C1orf216	0.003355875	
RCL1	0.003355875	
TMEM127	0.003355875	
DLEU2	0.003355875	
RAB3D	0.003355875	
CALD1	0.003355875	
PPFIBP2	0.003355875	
GPN3	0.003355875	
ZNF627	0.003355875	
TMEM156	0.003355875	
KCTD3	0.003355875	
PIGA	0.003355875	
SUPT20H	0.003355875	
LYPLA1	0.003355875	

SHC3	0.003355875	
C19orf48	0.003355875	
CXCL11	0.003355875	
MGC32805	0.003355875	
GPATCH1	0.003355875	
IL12B	0.003355875	
HERC4	0.003355875	
S100A13	0.003355875	
ANKHD1-EIF4EBP3	0.003355875	
ZNF440	0.003355875	
LRR1	0.003355875	
NIFK-AS1	0.003355875	
SNHG17	0.003355875	
BRWD3	0.003355875	
MAPKAPK3	0.003355875	
HMGB3	0.003355875	
UFM1	0.003355875	
KRBA1	0.003355875	
CCDC71	0.003355875	
TSN	0.003355875	
SOX30	0.003355875	
LIMA1	0.003355875	
DUSP10	0.003355875	
NEBL	0.003355875	
GPRC5C	0.003355875	
ALG11	0.003355875	
FBXO42	0.003355875	
RPUSD4	0.003355875	
CORO1B	0.003355875	
TXNRD2	0.003355875	
HIST1H3I	0.003355875	
LRRFIP1	0.003355875	
SSH2	0.003355875	
TIPARP	0.003355875	
SLX4IP	0.003355875	
HIST1H4D	0.003355875	
GDAP1	0.003355875	
SYNRG	0.003355875	
DHRS4-AS1	0.003355875	
BSPRY	0.003355875	
ME3	0.003355875	

AOC4P	0.003355875	
RAP2B	0.003305029	
GALK2	0.003305029	
IER5L	0.003305029	
TRAF3IP2	0.003305029	
JPX	0.003305029	
DNAJC27	0.003305029	
TFPI2	0.003305029	
BTF3L4	0.003305029	
VPS13B	0.003305029	
ZNF208	0.003305029	
ZMYM5	0.003305029	
IGHV3-23	0.003305029	
GKAP1	0.003305029	
RNF149	0.003305029	
PCBP1-AS1	0.003305029	
MKRN1	0.003305029	
PLCH2	0.003305029	
TPT1-AS1	0.003305029	
AARS2	0.003305029	
SPOCK2	0.003305029	
DESI2	0.003305029	
NRROS	0.003305029	
RP4-756H11.5	0.003305029	
PIGG	0.003305029	
CXXC1	0.003305029	
CDS2	0.003305029	
RALGAPA1	0.003305029	
FPR3	0.003305029	
RP4-773N10.4	0.003305029	
INTS4	0.003305029	
SFRP4	0.003305029	
HTR1D	0.003305029	
HOXA11	0.003305029	
DARS2	0.003305029	
ZBED5-AS1	0.003305029	
IFT20	0.003305029	
PKP2	0.003305029	
CH17-472G23.4	0.003305029	
PPT1	0.003305029	
NAPRT	0.003305029	

MTCH2	0.003305029	
EPHB6	0.003305029	
CLCN5	0.003305029	
ZNF785	0.003305029	
CLCN3	0.003305029	
SHKBP1	0.003305029	
EFCAB14	0.003305029	
RBBP9	0.003305029	
SLC39A5	0.003305029	
TMOD1	0.003305029	
DPY19L1	0.003305029	
IGHV3-74	0.003305029	
NEU3	0.003305029	
CCDC36	0.003305029	
DRAM1	0.003305029	
ABCD4	0.003305029	
HMGN3	0.003305029	
PCSK5	0.003305029	
PLEKHG4	0.003305029	
ANGEL1	0.003305029	
AADAT	0.003305029	
NUDT9	0.003305029	
FAM76B	0.003305029	
GTF3C6	0.003305029	
AGA	0.003305029	
KCMF1	0.003305029	
HIST1H3F	0.003305029	
SMOC1	0.003305029	
ARPC1A	0.003305029	
COX16	0.003305029	
KRT18P34	0.003305029	
IFT80	0.003305029	
NOB1	0.003305029	
TCEANC2	0.003305029	
WRNIP1	0.003305029	
C10orf2	0.003305029	
TSPAN3	0.003305029	
HOXC6	0.003305029	
FGF7	0.003305029	
CAP2	0.003305029	
SCO1	0.003305029	

RABGEF1	0.003305029	
HDDC3	0.003305029	
NREP	0.003305029	
FAM73A	0.003305029	
ATP1B3	0.003305029	
SLC25A22	0.003305029	
MOS	0.003305029	
AKAP6	0.003305029	
ELOVL7	0.003305029	
CLGN	0.003305029	
COPS8	0.003305029	
CMSS1	0.003305029	
ALKBH8	0.003305029	
ZNF12	0.003305029	
ATL2	0.003305029	
DONSON	0.003305029	
USP27X	0.003305029	
TBX15	0.003305029	
KCNMB4	0.003305029	
ZNF143	0.003305029	
PLCXD2	0.003305029	
ABI3BP	0.003305029	
HMP19	0.003305029	
GIGYF1	0.003305029	
IGLV2-23	0.003305029	
DARS	0.003305029	
CDC37L1	0.003305029	
RINL	0.003305029	
CCDC112	0.003305029	
LBX2-AS1	0.003305029	
CTSL	0.003305029	
TIGD2	0.003305029	
FBXL17	0.003305029	
NUP50	0.003305029	
C3orf62	0.003305029	
FBXO30	0.003305029	
SH3RF3	0.003305029	
VASH1	0.003305029	
H19	0.003305029	
TSPAN4	0.003305029	
NOTUM	0.003305029	

CXCR1	0.003305029	
HS3ST3A1	0.003305029	
C2orf44	0.003254182	
CRIM1	0.003254182	
RDH13	0.003254182	
LMBRD2	0.003254182	
FAM184A	0.003254182	
PEX11B	0.003254182	
UBA3	0.003254182	
AMPD3	0.003254182	
RP11-522I20.3	0.003254182	
DHCR24	0.003254182	
BZW2	0.003254182	
QSOX1	0.003254182	
UROC1	0.003254182	
NKAP	0.003254182	
SBDS	0.003254182	
MFSD12	0.003254182	
EOMES	0.003254182	
MBD4	0.003254182	
IL15	0.003254182	
IGSF1	0.003254182	
ST3GAL5	0.003254182	
TMUB1	0.003254182	
SAMD4A	0.003254182	
PLVAP	0.003254182	
MICA	0.003254182	
CLEC10A	0.003254182	
ALDH1L1	0.003254182	
TSSK3	0.003254182	
ZFYVE26	0.003254182	
ICT1	0.003254182	
CCDC67	0.003254182	
MSANTD3	0.003254182	
ZNF674	0.003254182	
MRRF	0.003254182	
TM9SF3	0.003254182	
IGLV1-44	0.003254182	
IFI44	0.003254182	
FAM219B	0.003254182	
FAM169A	0.003254182	

CPVL	0.003254182	
SCHIP1	0.003254182	
AGFG2	0.003254182	
AC245100.1	0.003254182	
RTTN	0.003254182	
KANSL1-AS1	0.003254182	
GAS8	0.003254182	
ZNF16	0.003254182	
LAMB2	0.003254182	
PRPF38B	0.003254182	
PITPNA-AS1	0.003254182	
EMCN	0.003254182	
NUB1	0.003254182	
TMEM239	0.003254182	
CCDC121	0.003254182	
IGHV5-51	0.003254182	
CXCL5	0.003254182	
BET1L	0.003254182	
PCDHGA2	0.003254182	
AAAS	0.003254182	
WRAP53	0.003254182	
TSSC1	0.003254182	
METTL17	0.003254182	
PRR14	0.003254182	
DNMT3L	0.003254182	
SSR3	0.003254182	
SPINK1	0.003254182	
IMPAD1	0.003254182	
RBFA	0.003254182	
OTUB2	0.003254182	
PPIC	0.003254182	
INSIG2	0.003254182	
ZNF670	0.003254182	
IGFLR1	0.003254182	
LRRC58	0.003254182	
SEC22B	0.003254182	
CABP1	0.003254182	
MUL1	0.003254182	
KLHL22	0.003254182	
HIP1R	0.003254182	
TMCC1	0.003254182	

CAPS	0.003254182	
PRSS8	0.003254182	
CDNF	0.003254182	
PCID2	0.003254182	
ZDHHC5	0.003254182	
MUC6	0.003254182	
NIP7	0.003254182	
ZKSCAN1	0.003254182	
CCBL1	0.003254182	
CLEC2B	0.003254182	
NARF	0.003254182	
PLXNB2	0.003254182	
P3H3	0.003254182	
HYOU1	0.003254182	
DIS3L	0.003254182	
IHH	0.003254182	
KLHL8	0.003254182	
HPGDS	0.003254182	
TRMT2B	0.003254182	
FRAT1	0.003254182	
SLC40A1	0.003254182	
COL15A1	0.003254182	
SLC35E1	0.003254182	
WNK4	0.003254182	
BTG3	0.003254182	
TMEM259	0.003254182	
VSIG4	0.003254182	
LGMN	0.003254182	
ZNF740	0.003254182	
CTSV	0.003254182	
TRIM44	0.003254182	
DNAJC3	0.003254182	
TRIM47	0.003254182	
MOCS1	0.003254182	
CAPZA2	0.003254182	
LMLN	0.003254182	
COMMD9	0.003254182	
ZBTB48	0.003254182	
SMIM4	0.003254182	
ARAP1	0.003254182	
FKTN	0.003254182	

TMEM123	0.003254182	
HMGCR	0.003254182	
YIPF5	0.003254182	
CHST8	0.003203336	
NECAB1	0.003203336	
NCS1	0.003203336	
CIDECP	0.003203336	
BCL11A	0.003203336	
RTCA	0.003203336	
PROSER2	0.003203336	
DIAPH2	0.003203336	
CECR1	0.003203336	
ATHL1	0.003203336	
ZXDA	0.003203336	
PRIMPOL	0.003203336	
PTBP2	0.003203336	
FGFR1OP	0.003203336	
LINC01314	0.003203336	
NECAP1	0.003203336	
ABLIM3	0.003203336	
WDPCP	0.003203336	
FGGY	0.003203336	
LINC01128	0.003203336	
RAB22A	0.003203336	
TNS2	0.003203336	
RASD2	0.003203336	
LYPLAL1	0.003203336	
DDX59	0.003203336	
DHRS12	0.003203336	
RSG1	0.003203336	
C11orf63	0.003203336	
RP11-793H13.11	0.003203336	
CPPED1	0.003203336	
SEC16B	0.003203336	
ZCCHC14	0.003203336	
HIST1H4C	0.003203336	
AGFG1	0.003203336	
GDPD5	0.003203336	
TMED7	0.003203336	
VMA21	0.003203336	
HCP5	0.003203336	

EIF4EBP3	0.003203336	
GPR137B	0.003203336	
FAM160B2	0.003203336	
FAM135A	0.003203336	
HNRNPCP2	0.003203336	
KCNJ2	0.003203336	
NOMO3	0.003203336	
NPR3	0.003203336	
POMT1	0.003203336	
TTC9	0.003203336	
TMEM150C	0.003203336	
SKAP2	0.003203336	
PERP	0.003203336	
KIF13B	0.003203336	
REPIN1	0.003203336	
SLC26A1	0.003203336	
HMHA1	0.003203336	
SERPINB8	0.003203336	
PIGH	0.003203336	
NUCB2	0.003203336	
TLR6	0.003203336	
ZNF84	0.003203336	
HOXB5	0.003203336	
GMFB	0.003203336	
TOM1L2	0.003203336	
PDZD2	0.003203336	
ZNF467	0.003203336	
FBXO44	0.003203336	
WFS1	0.003203336	
PPP1R32	0.003203336	
LINC00844	0.003203336	
DCP1B	0.003203336	
HRH1	0.003203336	
ATXN7L2	0.003203336	
CYFIP1	0.003203336	
CHAMP1	0.003203336	
VPS37D	0.003203336	
SYP	0.003203336	
MGAT5	0.003203336	
HSH2D	0.003203336	
PTX3	0.003203336	

RBMX2	0.003203336	
SMOC2	0.003203336	
CSPP1	0.003203336	
SPATA5	0.003203336	
C14orf119	0.003203336	
ALOX12	0.003203336	
ZNF592	0.003203336	
SH3RF2	0.003203336	
GOSR2	0.003203336	
ATG14	0.003203336	
GALE	0.003203336	
KCNJ8	0.003203336	
NPTX2	0.003203336	
B9D2	0.003203336	
PVR	0.003203336	
ANGPT2	0.003203336	
CYP4F2	0.003203336	
ZNF428	0.003203336	
RAB24	0.003203336	
DUSP3	0.003203336	
CD244	0.003203336	
KCNIP2	0.003203336	
SPATA20	0.003203336	
MARCO	0.003203336	
TMEM37	0.003203336	
SSTR2	0.003203336	
LRRC45	0.003203336	
CDC42BPA	0.003203336	
LIG3	0.003203336	
MMADHC	0.003203336	
FCAMR	0.003203336	
C19orf54	0.003203336	
TMEM14A	0.003203336	
SPATA2	0.003203336	
EIF2B5	0.003203336	
RNF219	0.003203336	
CTD-3074O7.5	0.003203336	
SPG7	0.003203336	
HIST1H2BG	0.003203336	
FAM91A1	0.003203336	
C21orf59	0.003203336	

NUDT16	0.003203336	
HMGB1P10	0.003152489	
MDFIC	0.003152489	
ZNF92	0.003152489	
CDH13	0.003152489	
CRTC1	0.003152489	
BLOC1S3	0.003152489	
NAGPA	0.003152489	
C11orf98	0.003152489	
GLOD5	0.003152489	
TMEM185B	0.003152489	
ZYG11A	0.003152489	
SLCO2B1	0.003152489	
SAE1	0.003152489	
ZBED8	0.003152489	
PLXNC1	0.003152489	
ZNF133	0.003152489	
CCDC6	0.003152489	
SDS	0.003152489	
OSTM1	0.003152489	
ZFYVE21	0.003152489	
UTP11L	0.003152489	
CLDN11	0.003152489	
DNAJC19	0.003152489	
LINC00174	0.003152489	
UBE3B	0.003152489	
WDR54	0.003152489	
TSPAN12	0.003152489	
OLR1	0.003152489	
BCAP29	0.003152489	
PVRL1	0.003152489	
CLIC4	0.003152489	
SLCO1B3	0.003152489	
SIGLEC7	0.003152489	
GNPDA2	0.003152489	
PYHIN1	0.003152489	
TRUB1	0.003152489	
ANGPTL2	0.003152489	
DSTNP2	0.003152489	
RFX4	0.003152489	
NQO2	0.003152489	

CCR6	0.003152489	
PDCD11	0.003152489	
ANO8	0.003152489	
CLC	0.003152489	
LNP1	0.003152489	
C6orf136	0.003152489	
FOXH1	0.003152489	
PRMT9	0.003152489	
NEXN	0.003152489	
APEX2	0.003152489	
MBOAT7	0.003152489	
ZIC4	0.003152489	
MFSD4	0.003152489	
GGCT	0.003152489	
AP1AR	0.003152489	
DOLPP1	0.003152489	
COX7A2L	0.003152489	
USP43	0.003152489	
KIAA1279	0.003152489	
CCDC115	0.003152489	
TIMMDC1	0.003152489	
RNF208	0.003152489	
ASB4	0.003152489	
NAA25	0.003152489	
PILRB	0.003152489	
CAMSAP2	0.003152489	
RP11-713M15.2	0.003152489	
OSBPL7	0.003152489	
CDC123	0.003152489	
DOCK3	0.003152489	
RTF1	0.003152489	
RHBDF1	0.003152489	
EIF2B2	0.003152489	
GORASP1	0.003152489	
IGKV2-24	0.003152489	
KMO	0.003152489	
CGA	0.003152489	
ZNF771	0.003152489	
PARP11	0.003152489	
ACOX3	0.003152489	
PIK3C2G	0.003152489	

THTPA	0.003152489	
PCNXL3	0.003152489	
EIF5A2	0.003152489	
NGLY1	0.003152489	
RP11-635N19.1	0.003152489	
ZNF3	0.003152489	
LHX9	0.003152489	
SMAP2	0.003152489	
SPON1	0.003152489	
CORO1C	0.003152489	
SEMA7A	0.003152489	
GGN	0.003152489	
SFR1	0.003152489	
LMBR1	0.003152489	
TMEM38B	0.003152489	
TSPYL4	0.003152489	
CHST9	0.003152489	
KLHL17	0.003152489	
UTP3	0.003152489	
CYP3A5	0.003152489	
DHX40	0.003152489	
ARHGAP27	0.003152489	
ZSCAN9	0.003152489	
SNHG16	0.003152489	
ABCC4	0.003152489	
MTPAP	0.003152489	
FAM3A	0.003152489	
P4HA3	0.003152489	
MGAT5B	0.003101642	
NEFL	0.003101642	
ZNF273	0.003101642	
TPP1	0.003101642	
RPAP2	0.003101642	
TOX2	0.003101642	
ARMCX5	0.003101642	
RP11-425L10.1	0.003101642	
EXT1	0.003101642	
URB2	0.003101642	
OAZ2	0.003101642	
THBS3	0.003101642	
TCEA3	0.003101642	

HEATR6	0.003101642	
CR2	0.003101642	
GFRA1	0.003101642	
GSTA2	0.003101642	
STXBP5	0.003101642	
WDR33	0.003101642	
ASUN	0.003101642	
SEC22A	0.003101642	
SOX7	0.003101642	
NBEA	0.003101642	
SLC39A6	0.003101642	
FAM65A	0.003101642	
MCMBP	0.003101642	
VANGL2	0.003101642	
ANGPT1	0.003101642	
PSMB8-AS1	0.003101642	
WDR53	0.003101642	
TNRC6C	0.003101642	
NAA30	0.003101642	
EID2	0.003101642	
GOLPH3L	0.003101642	
LAMC3	0.003101642	
CCNL1	0.003101642	
ZNF839	0.003101642	
HMGN5	0.003101642	
SWT1	0.003101642	
KDM4B	0.003101642	
PNPLA2	0.003101642	
CHRM3	0.003101642	
LTB4R	0.003101642	
ZXDB	0.003101642	
RASA4CP	0.003101642	
TMEM44-AS1	0.003101642	
EDNRB	0.003101642	
GALNT15	0.003101642	
SCNN1A	0.003101642	
PRPH2	0.003101642	
ZNF335	0.003101642	
DPH1	0.003101642	
TSPAN17	0.003101642	
NPTXR	0.003101642	

SIPA1L1	0.003101642	
ABCC9	0.003101642	
SAMHD1	0.003101642	
FAM50B	0.003101642	
ZNF397	0.003101642	
PRR5	0.003101642	
INPP5E	0.003101642	
IFI27	0.003101642	
PIK3AP1	0.003101642	
CARS	0.003101642	
ARRDC4	0.003101642	
CNN3	0.003101642	
CD1A	0.003101642	
DPF3	0.003101642	
BTN3A3	0.003101642	
ITGBL1	0.003101642	
GPIHBP1	0.003101642	
HIF3A	0.003101642	
BLOC1S6	0.003101642	
FUT3	0.003101642	
AVEN	0.003101642	
TUBA1C	0.003101642	
MUC12	0.003101642	
DCPS	0.003101642	
CLIC6	0.003101642	
ELAVL2	0.003101642	
STIM1	0.003101642	
MOCOS	0.003101642	
ABCC5	0.003101642	
SPTA1	0.003101642	
SGPP2	0.003101642	
PACRG	0.003101642	
NADSYN1	0.003101642	
FAM32A	0.003101642	
LINC00969	0.003101642	
EIF5B	0.003101642	
SPATA41	0.003101642	
PARVA	0.003101642	
WDFY1	0.003101642	
LRRCC1	0.003101642	
STK40	0.003101642	

C1GALT1	0.003101642	
RPH3AL	0.003101642	
MSRA	0.003101642	
TRIM65	0.003101642	
C7orf43	0.003101642	
EML1	0.003101642	
MTND5P11	0.003101642	
HTR2A	0.003101642	
GPBP1	0.003101642	
SLC9A5	0.003101642	
RP13-104F24.3	0.003101642	
MAMLD1	0.003101642	
FAM46B	0.003101642	
SHROOM1	0.003101642	
RDH14	0.003101642	
MMP17	0.003101642	
RASL12	0.003101642	
PMS2P1	0.003101642	
EMC8	0.003101642	
ARMC10	0.003101642	
DNAJC2	0.003101642	
AC093323.3	0.003101642	
PTGFRN	0.003101642	
PDHB	0.003101642	
C12orf60	0.003101642	
EML4	0.003050796	
HYAL1	0.003050796	
GBF1	0.003050796	
MAP6	0.003050796	
ZNF345	0.003050796	
CPSF4	0.003050796	
NOL9	0.003050796	
RNF183	0.003050796	
ZC3H6	0.003050796	
PHF20	0.003050796	
RTN4RL2	0.003050796	
PEX12	0.003050796	
SCN2A	0.003050796	
IGHGP	0.003050796	
GPSM1	0.003050796	
ARFGAP3	0.003050796	

PFKFB2	0.003050796	
HIST1H1E	0.003050796	
DAZ1	0.003050796	
RNASEL	0.003050796	
FKBP15	0.003050796	
TTC31	0.003050796	
RP11-545E17.3	0.003050796	
NET1	0.003050796	
H2AFV	0.003050796	
P3H4	0.003050796	
NT5C3B	0.003050796	
IGKV1-9	0.003050796	
AP006621.5	0.003050796	
MVK	0.003050796	
HLA-DOB	0.003050796	
KLHL5	0.003050796	
C19orf40	0.003050796	
KCNJ3	0.003050796	
COASY	0.003050796	
PDCD2L	0.003050796	
PANK4	0.003050796	
ZNF710	0.003050796	
PARP16	0.003050796	
RPL41P1	0.003050796	
CCDC92	0.003050796	
C12orf49	0.003050796	
TAS2R64P	0.003050796	
RHEB	0.003050796	
C2orf16	0.003050796	
RNU4-2	0.003050796	
TASP1	0.003050796	
WNT2	0.003050796	
KLHL29	0.003050796	
CCL22	0.003050796	
TTC32	0.003050796	
KIAA0895L	0.003050796	
TRIM35	0.003050796	
MALSU1	0.003050796	
ATP6V1D	0.003050796	
C8orf58	0.003050796	
CA14	0.003050796	

CD99	0.003050796	
S1PR2	0.003050796	
SRI	0.003050796	
TNFSF13	0.003050796	
SLC34A1	0.003050796	
AGPAT9	0.003050796	
ETHE1	0.003050796	
RNF150	0.003050796	
AK1	0.003050796	
GPX1P1	0.003050796	
WISP1	0.003050796	
PROCR	0.003050796	
ATXN7L1	0.003050796	
MS4A4A	0.003050796	
GRAP2	0.003050796	
LRRN2	0.003050796	
ANKRD16	0.003050796	
UBA6	0.003050796	
FKBP1B	0.003050796	
STYXL1	0.003050796	
INIP	0.003050796	
CAMK1D	0.003050796	
IGLON5	0.003050796	
CCL18	0.003050796	
CCT8P1	0.003050796	
DENND2D	0.003050796	
TMEM192	0.003050796	
MIF4GD	0.003050796	
NGF	0.003050796	
APLN	0.003050796	
PTPN21	0.003050796	
HOXA11-AS	0.003050796	
THEM6	0.003050796	
NXT2	0.003050796	
AC024560.3	0.003050796	
PLEKHA2	0.003050796	
ARSE	0.003050796	
HINT3	0.003050796	
ZNF331	0.003050796	
EMC4	0.003050796	
CDC42SE2	0.003050796	

PIK3CB	0.003050796	
GHDC	0.003050796	
FASTKD2	0.003050796	
H2AFJ	0.003050796	
TMEM216	0.003050796	
SNHG12	0.003050796	
OXR1	0.003050796	
ULK2	0.003050796	
CBY1	0.003050796	
SLC25A40	0.003050796	
FAM126B	0.003050796	
SLC10A3	0.003050796	
HIST1H4H	0.003050796	
ADAMTS15	0.003050796	
SEC14L4	0.003050796	
CHRNE	0.003050796	
RP11-90L1.8	0.003050796	
WAC-AS1	0.003050796	
FLT3LG	0.003050796	
ZNF251	0.003050796	
LRFN1	0.003050796	
FLT3	0.003050796	
PHRF1	0.003050796	
ELOVL2	0.003050796	
AMH	0.003050796	
MT1L	0.003050796	
TRPV1	0.003050796	
IGKV3-11	0.003050796	
MAN2C1	0.003050796	
EFHD1	0.003050796	
ASCL2	0.003050796	
KB-1208A12.3	0.003050796	
CDIP1	0.003050796	
LITAF	0.003050796	
HDDC2	0.003050796	
NTMT1	0.003050796	
ZNF675	0.003050796	
OMA1	0.003050796	
RP11-254I22.3	0.003050796	
CRYZ	0.003050796	
PIGN	0.002999949	

GRAMD1B	0.002999949	
PLB1	0.002999949	
AC012358.8	0.002999949	
CAMTA1	0.002999949	
SLC11A2	0.002999949	
NCK1-AS1	0.002999949	
TMOD3	0.002999949	
MSH4	0.002999949	
MT1A	0.002999949	
PI4K2A	0.002999949	
PLA2G2D	0.002999949	
GSKIP	0.002999949	
MIDN	0.002999949	
TMX3	0.002999949	
RP5-1065J22.8	0.002999949	
CTTNBP2	0.002999949	
FAM19A5	0.002999949	
ACTN3	0.002999949	
PPHLN1	0.002999949	
EVI5	0.002999949	
SERINC5	0.002999949	
RBM3	0.002999949	
TRIM56	0.002999949	
ENOPH1	0.002999949	
RPS4Y1	0.002999949	
TXLNB	0.002999949	
ZDHHC20	0.002999949	
FAM167A	0.002999949	
RBMS1	0.002999949	
USP30	0.002999949	
SCFD1	0.002999949	
PDCD1LG2	0.002999949	
WDR34	0.002999949	
ELP4	0.002999949	
ZNF527	0.002999949	
FMN1	0.002999949	
ADAMTS7	0.002999949	
ATPAF1	0.002999949	
RP11-119D9.1	0.002999949	
SNX7	0.002999949	
C5orf24	0.002999949	

POLRMT	0.002999949	
P2RY13	0.002999949	
DNA2	0.002999949	
VILL	0.002999949	
TSPYL5	0.002999949	
HBA1	0.002999949	
ARSI	0.002999949	
PHF8	0.002999949	
SPRY4	0.002999949	
PGBD1	0.002999949	
MGRN1	0.002999949	
FUK	0.002999949	
LIF	0.002999949	
BPHL	0.002999949	
BOK	0.002999949	
AKR1C3	0.002999949	
SPAG16	0.002999949	
CDHR3	0.002999949	
SLC22A4	0.002999949	
CH17-118O6.2	0.002999949	
IGKV1-5	0.002999949	
ITGA11	0.002999949	
HIST1H4B	0.002999949	
USB1	0.002999949	
HLA-DRB5	0.002999949	
ZNF718	0.002999949	
ARHGAP42	0.002999949	
VLDLR	0.002999949	
SHQ1	0.002999949	
SLC6A2	0.002999949	
GPR88	0.002999949	
GIMAP6	0.002999949	
IGLV1-40	0.002999949	
C16orf72	0.002999949	
CDX1	0.002999949	
RAG1	0.002999949	
SLC16A3	0.002999949	
TSHR	0.002999949	
RP11-175B9.3	0.002999949	
ZNF394	0.002999949	
MTMR9LP	0.002999949	

ZNF175	0.002999949	
DFNB31	0.002999949	
ZNF43	0.002999949	
EVA1C	0.002999949	
KRT17P8	0.002999949	
LTV1	0.002999949	
HECTD3	0.002999949	
NMRK1	0.002999949	
SUGCT	0.002999949	
RTCB	0.002999949	
43530	0.002999949	
ZNF792	0.002999949	
PRPF38A	0.002999949	
THEM4	0.002999949	
C1GALT1C1	0.002999949	
KCNE3	0.002999949	
PEPD	0.002999949	
NOL4	0.002999949	
GPM6A	0.002999949	
TACC1	0.002999949	
NIN	0.002999949	
CD38	0.002999949	
FCER1A	0.002999949	
C7orf26	0.002999949	
ENTPD2	0.002999949	
DUSP5	0.002999949	
ADAMTS17	0.002999949	
CKM	0.002999949	
POU4F1	0.002999949	
DYNLT3	0.002999949	
LINC00957	0.002999949	
AC069213.1	0.002999949	
STK17A	0.002999949	
PRCP	0.002999949	
RRP7A	0.002999949	
ZNF268	0.002999949	
GMPR2	0.002999949	
PCDH1	0.002999949	
DDX26B	0.002999949	
ZNF564	0.002999949	
ZSCAN26	0.002999949	

BNIPL	0.002999949	
STEAP2	0.002999949	
G0S2	0.002999949	
PHLDB3	0.002999949	
SMU1	0.002999949	
RBM45	0.002999949	
MORC2	0.002999949	
FBXL3	0.002999949	
NOL11	0.002999949	
SEC16A	0.002999949	
DNAJC12	0.002949103	
EI24	0.002949103	
NIT2	0.002949103	
ZFP62	0.002949103	
PCDHGB2	0.002949103	
PPA2	0.002949103	
HYAL2	0.002949103	
IWS1	0.002949103	
MANSC1	0.002949103	
MTFP1	0.002949103	
ZFHX4	0.002949103	
MEX3C	0.002949103	
IL22RA1	0.002949103	
MLKL	0.002949103	
RP11-59D5__B.2	0.002949103	
WDR89	0.002949103	
GATA5	0.002949103	
HLA-J	0.002949103	
WNT3	0.002949103	
C16orf87	0.002949103	
C14orf142	0.002949103	
UCK2	0.002949103	
FAM155B	0.002949103	
RP5-821D11.7	0.002949103	
ZNF668	0.002949103	
ZNF579	0.002949103	
SH3BGRL	0.002949103	
RNF217	0.002949103	
LL22NC03-80A10.6	0.002949103	
SPTLC2	0.002949103	
C11orf80	0.002949103	

HOXD8	0.002949103	
PGM2	0.002949103	
PITHD1	0.002949103	
NTPCR	0.002949103	
PAG1	0.002949103	
ZDHHC6	0.002949103	
TMPO-AS1	0.002949103	
RP4-568C11.4	0.002949103	
ARMC6	0.002949103	
SEC31B	0.002949103	
CYB5R3	0.002949103	
NPTN	0.002949103	
LONRF3	0.002949103	
CHFR	0.002949103	
IGSF8	0.002949103	
COX10	0.002949103	
EIF2AK1	0.002949103	
ZNF569	0.002949103	
DIXDC1	0.002949103	
FTCDNL1	0.002949103	
PARP2	0.002949103	
VPS51	0.002949103	
EXPH5	0.002949103	
CD300E	0.002949103	
SPRTN	0.002949103	
CDS1	0.002949103	
NOM1	0.002949103	
IFIT2	0.002949103	
SYNGR1	0.002949103	
AK5	0.002949103	
BTBD9	0.002949103	
PALLD	0.002949103	
TIMP4	0.002949103	
TMEM86A	0.002949103	
CIZ1	0.002949103	
ITPRIP	0.002949103	
SUN1	0.002949103	
AGAP3	0.002949103	
TANGO2	0.002949103	
IGHV4-59	0.002949103	
HOOK3	0.002949103	

SFMBT2	0.002949103	
ANKRD13A	0.002949103	
GTF2H2C	0.002949103	
AFAP1L2	0.002949103	
EXOC6	0.002949103	
RGS12	0.002949103	
TMEM115	0.002949103	
TTC3P1	0.002949103	
ARHGAP33	0.002949103	
STK32C	0.002949103	
SOWAHA	0.002949103	
FBXO27	0.002949103	
CPT2	0.002949103	
HSPA14	0.002949103	
RRBP1	0.002949103	
MAGOHB	0.002949103	
SNAPC3	0.002949103	
PLEKHA4	0.002949103	
ZNF585A	0.002949103	
FAM109A	0.002949103	
OXT	0.002949103	
AMBRA1	0.002949103	
BFAR	0.002949103	
TUBA4B	0.002949103	
PITPNM2	0.002949103	
UNG	0.002949103	
ZNF550	0.002949103	
PNPLA4	0.002949103	
ARRDC1-AS1	0.002949103	
CRTAP	0.002949103	
KLRC4	0.002949103	
FBXO4	0.002949103	
NPL	0.002949103	
ADIPOR2	0.002949103	
REEP3	0.002949103	
ZNF396	0.002949103	
RP11-1017G21.5	0.002949103	
RGL1	0.002949103	
KRT13	0.002949103	
GALNT14	0.002949103	
AC009299.3	0.002949103	

PGM2L1	0.002949103	
SCRN2	0.002949103	
ACADL	0.002949103	
IFT57	0.002949103	
TMEM198B	0.002949103	
PAPD7	0.002949103	
SLC39A11	0.002949103	
EPSTI1	0.002949103	
C5orf30	0.002949103	
ZNF266	0.002949103	
PCCA	0.002949103	
CHST1	0.002898256	
DNAJB6	0.002898256	
KIAA1522	0.002898256	
PTTG1IP	0.002898256	
DAK	0.002898256	
PTPRZ1	0.002898256	
THUMPD2	0.002898256	
RP11-504P24.8	0.002898256	
C11orf57	0.002898256	
TRNP1	0.002898256	
CCNI	0.002898256	
DBR1	0.002898256	
TMEM254	0.002898256	
AAK1	0.002898256	
EN1	0.002898256	
TMEM180	0.002898256	
P2RX2	0.002898256	
TBC1D15	0.002898256	
MTMR11	0.002898256	
HEBP2	0.002898256	
NEURL1B	0.002898256	
RABL2A	0.002898256	
RP11-158K1.3	0.002898256	
ACO2	0.002898256	
CD200	0.002898256	
MMP24	0.002898256	
ZCCHC24	0.002898256	
COQ6	0.002898256	
DSCC1	0.002898256	
MPND	0.002898256	

CAB39L	0.002898256	
ST7	0.002898256	
CNOT10	0.002898256	
FAM126A	0.002898256	
TMEM44	0.002898256	
MN1	0.002898256	
QPCTL	0.002898256	
DHRS7B	0.002898256	
HIST1H1A	0.002898256	
MAGEH1	0.002898256	
METTL23	0.002898256	
TRPC4AP	0.002898256	
ZMYND10	0.002898256	
PPT2	0.002898256	
HSD3B2	0.002898256	
IL1R2	0.002898256	
DOCK7	0.002898256	
C9orf156	0.002898256	
ANKRA2	0.002898256	
NAPEPLD	0.002898256	
SMPD3	0.002898256	
SLC39A10	0.002898256	
ABRACL	0.002898256	
PPP1R26	0.002898256	
KLRD1	0.002898256	
PPP1R1B	0.002898256	
PAX9	0.002898256	
RETSAT	0.002898256	
PTGER3	0.002898256	
EHBP1L1	0.002898256	
MARCKSL1	0.002898256	
TJAP1	0.002898256	
AVPI1	0.002898256	
GTPBP10	0.002898256	
C1orf27	0.002898256	
KDM2B	0.002898256	
NPEPL1	0.002898256	
ACER3	0.002898256	
ZNF551	0.002898256	
CHIC1	0.002898256	
HIST3H2BB	0.002898256	

SMIM10	0.002898256	
FN3K	0.002898256	
PHGDH	0.002898256	
UBE2Q2	0.002898256	
ST14	0.002898256	
CENPN	0.002898256	
RP11-736K20.4	0.002898256	
PITPNM3	0.002898256	
FAM217B	0.002898256	
ADIPOR1	0.002898256	
TMEM177	0.002898256	
FITM1	0.002898256	
H3F3AP4	0.002898256	
LRCH3	0.002898256	
IL17RB	0.002898256	
IGLV2-11	0.002898256	
GPALPP1	0.002898256	
RALB	0.002898256	
LTC4S	0.002898256	
IFI27L1	0.002898256	
TRIM14	0.002898256	
RNF34	0.002898256	
ZNF131	0.002898256	
LRP3	0.002898256	
MAT2B	0.002898256	
SLC22A1	0.002898256	
FNIP1	0.002898256	
CTD-3138B18.5	0.002898256	
SLCO4A1	0.002898256	
MYT1	0.002898256	
PET117	0.002898256	
TCHP	0.002898256	
ZNF852	0.002898256	
OPLAH	0.002898256	
PLCH1	0.002898256	
IKBIP	0.002898256	
LMAN2L	0.002898256	
MFSD1	0.002898256	
PHACTR2	0.002898256	
TM2D2	0.002898256	
MOGS	0.002898256	

PRPF40B	0.002898256	
DMC1	0.002898256	
DAP	0.002898256	
CORO2A	0.002898256	
NFU1	0.002898256	
TNN	0.002898256	
IQCH	0.002898256	
ZNF74	0.002898256	
ALKBH1	0.002898256	
COA5	0.002898256	
RNF216	0.002898256	
RBM4B	0.002898256	
ACOT9	0.002898256	
HPGD	0.002898256	
PACRGL	0.002898256	
ATP2B4	0.002898256	
TRPC6	0.002898256	
ERI1	0.002898256	
HGFAC	0.002898256	
LA16c-380H5.5	0.002898256	
ABCB7	0.002898256	
FCGR2B	0.002898256	
GLTSCR1	0.002898256	
GPR37	0.002898256	
IFI6	0.002898256	
ACAP3	0.002898256	
ARG2	0.002898256	
ANKRD13C	0.002898256	
ISPD	0.002898256	
AC074117.10	0.002898256	
TMEM39A	0.002898256	
HSD17B7P2	0.002898256	
RPE	0.002898256	
PCMTD2	0.002898256	
LAMB3	0.002898256	
NKIRAS2	0.002847409	
HAMP	0.002847409	
AMACR	0.002847409	
HIST1H1B	0.002847409	
PISD	0.002847409	
PITPNC1	0.002847409	

HECW2	0.002847409	
BDH2	0.002847409	
CTD-2017D11.1	0.002847409	
DPH5	0.002847409	
CCDC61	0.002847409	
ZNF595	0.002847409	
UNC5B	0.002847409	
C1orf21	0.002847409	
NHLRC1	0.002847409	
CCDC127	0.002847409	
BATF2	0.002847409	
RP1-193H18.2	0.002847409	
PAX1	0.002847409	
NAPB	0.002847409	
UMAD1	0.002847409	
BOLA3	0.002847409	
PCDH9	0.002847409	
MALL	0.002847409	
OPA3	0.002847409	
ARHGAP12	0.002847409	
CDHR2	0.002847409	
ZNF75D	0.002847409	
SLC44A4	0.002847409	
MGEA5	0.002847409	
LIMK2	0.002847409	
LARP1B	0.002847409	
SLC39A3	0.002847409	
MT1G	0.002847409	
C17orf80	0.002847409	
RPL4P4	0.002847409	
CTD-2287O16.1	0.002847409	
ARVCF	0.002847409	
CPD	0.002847409	
RAB20	0.002847409	
IMMP1L	0.002847409	
ATP5S	0.002847409	
HHIP	0.002847409	
SLC25A38	0.002847409	
RP11-181C3.1	0.002847409	
ENKD1	0.002847409	
MZT1	0.002847409	

ABCB6	0.002847409	
RP4-717I23.3	0.002847409	
PPBP	0.002847409	
C9orf72	0.002847409	
ZNF787	0.002847409	
TIAF1	0.002847409	
NCALD	0.002847409	
ZFAND4	0.002847409	
RP11-10A14.4	0.002847409	
SNX19	0.002847409	
DEPDC5	0.002847409	
DPY19L4	0.002847409	
LACC1	0.002847409	
C17orf107	0.002847409	
NRSN2	0.002847409	
PDE4C	0.002847409	
IFIT1	0.002847409	
PXK	0.002847409	
VWA1	0.002847409	
KIAA0040	0.002847409	
STON2	0.002847409	
NR2C2AP	0.002847409	
RAB40C	0.002847409	
C1orf233	0.002847409	
INVS	0.002847409	
HNRNPA0	0.002847409	
ENGASE	0.002847409	
THSD1	0.002847409	
LINC00339	0.002847409	
43719	0.002847409	
EGF	0.002847409	
CAMKMT	0.002847409	
LRRC37A7P	0.002847409	
C10orf35	0.002847409	
SEC24B	0.002847409	
ZNF816	0.002847409	
SLC20A2	0.002847409	
FEM1C	0.002847409	
PPP1R3F	0.002847409	
HNRNPUL2	0.002847409	
BTBD11	0.002847409	

KIAA0141	0.002847409	
SLCO3A1	0.002847409	
DCX	0.002847409	
SLC29A1	0.002847409	
ADPRH	0.002847409	
ZNF747	0.002847409	
PHKA1	0.002847409	
TMEM30A	0.002847409	
TNFRSF21	0.002847409	
RP11-30P6.6	0.002847409	
RDH12	0.002847409	
RNF13	0.002847409	
FSTL3	0.002847409	
CCNY	0.002847409	
SAV1	0.002847409	
ELOVL4	0.002847409	
pk	0.002847409	
SLMO2	0.002847409	
MIR600HG	0.002847409	
CTSS	0.002847409	
LILRA5	0.002847409	
TMEM242	0.002847409	
RBSN	0.002847409	
CLUAP1	0.002847409	
KRT5	0.002847409	
GLT8D2	0.002847409	
GNGT2	0.002847409	
BDKRB1	0.002847409	
ST8SIA1	0.002847409	
AC004453.8	0.002847409	
BIN3	0.002847409	
ATG9A	0.002847409	
HIST1H2BM	0.002847409	
CNTN2	0.002847409	
CNTF	0.002847409	
HNRNPU-AS1	0.002847409	
PITPNM1	0.002847409	
GAPDHP1	0.002847409	
TMEM161B	0.002847409	
NPHP1	0.002847409	
ZNF83	0.002847409	

ZNF337	0.002847409	
XXbac-BPG248L24.12	0.002847409	
NMNAT1	0.002847409	
ZNF629	0.002847409	
RARRES2	0.002847409	
VEPH1	0.002847409	
IGHV1-24	0.002847409	
C17orf61-PLSCR3	0.002847409	
IGF2BP2	0.002847409	
RP11-130L8.2	0.002847409	
ADCK4	0.002847409	
CTIF	0.002847409	
COLCA2	0.002847409	
CPEB4	0.002847409	
ARRDC3	0.002847409	
MOB1B	0.002847409	
TSR2	0.002847409	
ZPR1	0.002847409	
XRCC6BP1	0.002847409	
SUSD2	0.002847409	
CYP21A1P	0.002847409	
TGFBI	0.002847409	
TRIM9	0.002796563	
SLC2A13	0.002796563	
PDE1C	0.002796563	
SFXN2	0.002796563	
RS1	0.002796563	
NISCH	0.002796563	
SMIM20	0.002796563	
HOXA-AS3	0.002796563	
OCIAD2	0.002796563	
MAST4	0.002796563	
RGP1	0.002796563	
COMMD10	0.002796563	
MST1P2	0.002796563	
MLF1	0.002796563	
CEP78	0.002796563	
RNPEP	0.002796563	
SBNO2	0.002796563	
VPS33A	0.002796563	
SLC2A1	0.002796563	

ZKSCAN2	0.002796563	
IER5	0.002796563	
ITGA7	0.002796563	
PPP2R3C	0.002796563	
GLO1	0.002796563	
THNSL1	0.002796563	
DCUN1D3	0.002796563	
RP11-484N16.1	0.002796563	
ADCY2	0.002796563	
SURF4	0.002796563	
CLDN16	0.002796563	
NHLH1	0.002796563	
ZKSCAN5	0.002796563	
DHRS13	0.002796563	
CLDN9	0.002796563	
UGDH	0.002796563	
CH17-472G23.2	0.002796563	
PAPSS1	0.002796563	
TMEM42	0.002796563	
RP11-166P13.3	0.002796563	
LDHC	0.002796563	
SPRYD3	0.002796563	
ZSWIM6	0.002796563	
RPL13AP25	0.002796563	
KIF12	0.002796563	
CCDC158	0.002796563	
BZRAP1	0.002796563	
DMRT3	0.002796563	
ZNF91	0.002796563	
CCRN4L	0.002796563	
CRLS1	0.002796563	
PTPRO	0.002796563	
PTGES3P1	0.002796563	
PYGB	0.002796563	
CENPP	0.002796563	
NIPAL2	0.002796563	
SAMD9L	0.002796563	
API5	0.002796563	
CAMSAP1	0.002796563	
HESX1	0.002796563	
USP50	0.002796563	

CHST7	0.002796563	
TUSC3	0.002796563	
TAPBPL	0.002796563	
PEG10	0.002796563	
ICAM2	0.002796563	
TEFM	0.002796563	
MCHR1	0.002796563	
SSUH2	0.002796563	
SH3BP5L	0.002796563	
ZNF112	0.002796563	
SELT	0.002796563	
TNFRSF11A	0.002796563	
C9orf41	0.002796563	
FAM189B	0.002796563	
ZNF559	0.002796563	
SCNN1B	0.002796563	
PYURF	0.002796563	
ITGAE	0.002796563	
RP5-857K21.7	0.002796563	
TPRN	0.002796563	
CERS4	0.002796563	
PCOLCE	0.002796563	
CACHD1	0.002796563	
RASGRF2	0.002796563	
SLC22A10	0.002796563	
HHIPL1	0.002796563	
SIKE1	0.002796563	
AIF1L	0.002796563	
NUDT8	0.002796563	
RNF213	0.002796563	
CIPC	0.002796563	
SMIM14	0.002796563	
LPIN1	0.002796563	
AC009403.2	0.002796563	
RSRC1	0.002796563	
RAB32	0.002796563	
AMICA1	0.002796563	
AGR2	0.002796563	
RCBTB1	0.002796563	
POLR3H	0.002796563	
COLEC12	0.002796563	

TMED5	0.002796563	
CFHR5	0.002796563	
HSPA12B	0.002796563	
TKTL1	0.002796563	
NLRC5	0.002796563	
PPFIA2	0.002796563	
NADK	0.002796563	
LINC00467	0.002796563	
FAM109B	0.002796563	
B3GNT7	0.002796563	
ZNF764	0.002796563	
KIF3A	0.002796563	
C4orf33	0.002796563	
PWWP2A	0.002796563	
ZNF256	0.002796563	
TMEM81	0.002796563	
GRSF1	0.002796563	
N4BP2L2	0.002796563	
PLEKHF1	0.002796563	
CD1C	0.002796563	
C1orf174	0.002796563	
KPTN	0.002796563	
CNKSR1	0.002796563	
ZSWIM7	0.002796563	
MIR497HG	0.002796563	
CNGB1	0.002796563	
PTPRU	0.002796563	
RASGRP2	0.002796563	
TSPAN6	0.002796563	
SPPL2A	0.002796563	
CCDC120	0.002796563	
TRIM45	0.002796563	
MAGEA4	0.002796563	
CCR7	0.002796563	
GALNT6	0.002796563	
ACSS3	0.002796563	
PIGBOS1	0.002796563	
USP38	0.002796563	
GLE1	0.002796563	
ADRB1	0.002796563	
PAQR7	0.002796563	

PPAP2C	0.002796563	
SLC4A8	0.002796563	
RDH11	0.002796563	
AP3B2	0.002745716	
ERMP1	0.002745716	
MARK1	0.002745716	
ERMAP	0.002745716	
HDC	0.002745716	
PGS1	0.002745716	
TMEM154	0.002745716	
C11orf1	0.002745716	
PLA2G6	0.002745716	
FIGNL1	0.002745716	
GEMIN8	0.002745716	
LHX6	0.002745716	
L3MBTL4	0.002745716	
RAP1GDS1	0.002745716	
NELL2	0.002745716	
AKR7A3	0.002745716	
KIAA1217	0.002745716	
VAV3	0.002745716	
ELAVL4	0.002745716	
RNF125	0.002745716	
ASPHD1	0.002745716	
ABHD4	0.002745716	
GRIN2A	0.002745716	
C7orf49	0.002745716	
TAL2	0.002745716	
RBM15B	0.002745716	
FUCA2	0.002745716	
CCR4	0.002745716	
CYP4F8	0.002745716	
MORN3	0.002745716	
DALRD3	0.002745716	
MIEF2	0.002745716	
CACNB4	0.002745716	
SYT6	0.002745716	
RP11-443B7.1	0.002745716	
ATG4C	0.002745716	
ARMC7	0.002745716	
TMEM241	0.002745716	

POLM	0.002745716	
HIBCH	0.002745716	
TMEM109	0.002745716	
CDK11A	0.002745716	
UNKL	0.002745716	
ZDHHC24	0.002745716	
POLDIP3	0.002745716	
PLEKHA1	0.002745716	
SIAE	0.002745716	
PAK3	0.002745716	
BOC	0.002745716	
MAGEA1	0.002745716	
DCHS1	0.002745716	
SNRNP48	0.002745716	
SEMA5B	0.002745716	
CLN6	0.002745716	
TBC1D25	0.002745716	
FBP2	0.002745716	
LMX1B	0.002745716	
FBXL5	0.002745716	
POU2F3	0.002745716	
C1orf56	0.002745716	
ARSB	0.002745716	
CSTA	0.002745716	
TMEM8A	0.002745716	
HIST1H2BF	0.002745716	
MFSD6	0.002745716	
LINC00924	0.002745716	
FXYD2	0.002745716	
DIRC2	0.002745716	
JADE2	0.002745716	
DUSP16	0.002745716	
ACOT2	0.002745716	
PTPRD	0.002745716	
DCBLD2	0.002745716	
TMEM139	0.002745716	
PARS2	0.002745716	
TARSL2	0.002745716	
SGK223	0.002745716	
PDGFRL	0.002745716	
ZNF641	0.002745716	

MIR22HG	0.002745716	
MT2P1	0.002745716	
GATC	0.002745716	
RP11-147L13.13	0.002745716	
SARAF	0.002745716	
PHF20L1	0.002745716	
RANBP6	0.002745716	
CMTR2	0.002745716	
ACTR10	0.002745716	
STXBP4	0.002745716	
GAL3ST1	0.002745716	
ULK1	0.002745716	
CUX2	0.002745716	
IBA57	0.002745716	
GS1-124K5.4	0.002745716	
KIAA1191	0.002745716	
THOP1	0.002745716	
AREL1	0.002745716	
ICAM5	0.002745716	
SMPD2	0.002745716	
LRRC8A	0.002745716	
SIDT2	0.002745716	
FCGRT	0.002745716	
PCDH7	0.002745716	
RP1-27K12.2	0.002745716	
TOR1AIP2	0.002745716	
PYGL	0.002745716	
NPY	0.002745716	
KCNN2	0.002745716	
SLC35A1	0.002745716	
RP11-147L13.12	0.002745716	
GMEB2	0.002745716	
RMDN1	0.002745716	
TDRD3	0.002745716	
UPRT	0.002745716	
C1RL-AS1	0.002745716	
MAGEF1	0.002745716	
PLIN5	0.002745716	
FAM86DP	0.002745716	
BCL2L2	0.002745716	
C1orf52	0.002745716	

RP11-226L15.5	0.002745716	
RP9P	0.002745716	
JKAMP	0.002745716	
PALM2	0.002745716	
BCAT2	0.002745716	
MRAS	0.002745716	
EZH1	0.002745716	
EFHC1	0.002745716	
BTNL9	0.002745716	
SLC35A4	0.002745716	
TLN2	0.002745716	
DDX50	0.002745716	
BAHCC1	0.002745716	
RAB3B	0.002745716	
TRAPPC9	0.002745716	
GCG	0.002745716	
PELI3	0.002745716	
UBE2G1	0.002745716	
ROGDI	0.002745716	
MALT1	0.002745716	
ZNF197	0.002745716	
PLD4	0.002745716	
MVB12A	0.002745716	
FAM188B	0.002745716	
SSTR3	0.002745716	
UGGT2	0.002745716	
CD80	0.002745716	
ACCS	0.002745716	
MMP15	0.002745716	
LIME1	0.002745716	
ST7L	0.00269487	
EDA2R	0.00269487	
TRAFD1	0.00269487	
SH3BP2	0.00269487	
ZNF555	0.00269487	
LINC00963	0.00269487	
VWA5A	0.00269487	
TPGS2	0.00269487	
NFKBIZ	0.00269487	
CCDC18	0.00269487	
LINC01018	0.00269487	

NABP1	0.00269487	
RAB15	0.00269487	
KLRC1	0.00269487	
ZNF808	0.00269487	
PARP8	0.00269487	
DTX3	0.00269487	
SH3D21	0.00269487	
FUT1	0.00269487	
METTL6	0.00269487	
GNB3	0.00269487	
BCDIN3D	0.00269487	
ADCYAP1	0.00269487	
UBXN8	0.00269487	
CTC-451P13.1	0.00269487	
USP6NL	0.00269487	
SDCBP2-AS1	0.00269487	
INHA	0.00269487	
SGTB	0.00269487	
HNRNPDL	0.00269487	
PNPLA8	0.00269487	
ZNF304	0.00269487	
SCFD2	0.00269487	
PAPLN	0.00269487	
CBWD1	0.00269487	
NAA16	0.00269487	
KCNK1	0.00269487	
EIF4E3	0.00269487	
RNF115	0.00269487	
ZNF844	0.00269487	
MYO1A	0.00269487	
NDUFAF5	0.00269487	
DNAH5	0.00269487	
RP11-383H13.1	0.00269487	
AP3M2	0.00269487	
NPHS1	0.00269487	
MUC5B	0.00269487	
SYNGR2	0.00269487	
CIB2	0.00269487	
GIMAP7	0.00269487	
ERRFI1	0.00269487	
PKIA	0.00269487	

RP11-800A3.4	0.00269487	
SPTB	0.00269487	
TMEM14C	0.00269487	
LRCH1	0.00269487	
NLRP2	0.00269487	
FAR2	0.00269487	
MEPCE	0.00269487	
TP53RK	0.00269487	
TATDN3	0.00269487	
ATP9B	0.00269487	
UBP1	0.00269487	
RAB11FIP4	0.00269487	
ZNF799	0.00269487	
TNXA	0.00269487	
TOX	0.00269487	
STARD9	0.00269487	
TBKBP1	0.00269487	
BCORL1	0.00269487	
SYCE1	0.00269487	
CYP4F22	0.00269487	
ADAT1	0.00269487	
RP11-196G11.5	0.00269487	
FAT4	0.00269487	
ARHGAP20	0.00269487	
GAP43	0.00269487	
IPMK	0.00269487	
DCAF16	0.00269487	
KIAA0226	0.00269487	
AANAT	0.00269487	
GSTT2B	0.00269487	
HES6	0.00269487	
TMEM87B	0.00269487	
ANKRD39	0.00269487	
ATRAID	0.00269487	
FOPNL	0.00269487	
ATP6V1A	0.00269487	
CHERP	0.00269487	
RGCC	0.00269487	
DPPA2	0.00269487	
UBXN2B	0.00269487	
FAM114A1	0.00269487	

CLN5	0.00269487	
WIPI1	0.00269487	
FAM21A	0.00269487	
DISP1	0.00269487	
DAB2IP	0.00269487	
TCAP	0.00269487	
RPUSD3	0.00269487	
ARHGEF26	0.00269487	
KCNAB2	0.00269487	
TRAK2	0.00269487	
MEX3B	0.00269487	
METTL2A	0.00269487	
SEC62	0.00269487	
ZMYND12	0.00269487	
PDP1	0.00269487	
SLC5A9	0.00269487	
STK38	0.00269487	
VIMP	0.00269487	
HSPA4L	0.00269487	
EXOC2	0.00269487	
GBP4	0.00269487	
CBWD2	0.00269487	
GALNT2	0.00269487	
LINC00324	0.00269487	
SLC37A4	0.00269487	
GTPBP1	0.00269487	
ANKRD24	0.00269487	
SUMF2	0.00269487	
TMIE	0.00269487	
NXPE3	0.00269487	
PMAIP1	0.00269487	
HLX	0.00269487	
ARHGEF34P	0.00269487	
KLHDC10	0.00269487	
STRADB	0.00269487	
TP73-AS1	0.00269487	
SULF1	0.00269487	
LRRC37B	0.00269487	
TMEM229B	0.00269487	
CHD7	0.00269487	
FOXD2-AS1	0.00269487	

FGFR1OP2	0.00269487	
QRICH2	0.00269487	
SC5D	0.00269487	
IGHV1-18	0.00269487	
STX18	0.00269487	
HSD3B1	0.00269487	
GABPB1-AS1	0.00269487	
TXNL4B	0.00269487	
LY96	0.00269487	
DNAAF2	0.002644023	
ATG4D	0.002644023	
NRXN3	0.002644023	
FAM173B	0.002644023	
PHOX2B	0.002644023	
ARL15	0.002644023	
FADS3	0.002644023	
GDAP2	0.002644023	
GUCY1B2	0.002644023	
NBEAL1	0.002644023	
UBL3	0.002644023	
LIN52	0.002644023	
RCOR2	0.002644023	
PLS3	0.002644023	
ECE2	0.002644023	
MS4A1	0.002644023	
DET1	0.002644023	
ZNF585B	0.002644023	
TMEM100	0.002644023	
AIFM2	0.002644023	
RTN2	0.002644023	
SOX12	0.002644023	
YIPF3	0.002644023	
INPP5B	0.002644023	
SNX17	0.002644023	
MARVELD3	0.002644023	
GK5	0.002644023	
TNFRSF9	0.002644023	
ESM1	0.002644023	
PPP3CC	0.002644023	
FOXJ1	0.002644023	
TMEM230	0.002644023	

HELZ2	0.002644023	
IGLV3-1	0.002644023	
KRT38	0.002644023	
NGB	0.002644023	
SLC17A9	0.002644023	
ULBP1	0.002644023	
SDPR	0.002644023	
ZNF134	0.002644023	
IL10RB	0.002644023	
MIR17HG	0.002644023	
CRH	0.002644023	
ZC3H7B	0.002644023	
GPR39	0.002644023	
MACC1	0.002644023	
ILDR1	0.002644023	
GSTA4	0.002644023	
PDLIM4	0.002644023	
MPP1	0.002644023	
CSRNP2	0.002644023	
TFB1M	0.002644023	
FLJ22763	0.002644023	
VPS4B	0.002644023	
GAN	0.002644023	
ATP2B1	0.002644023	
CDK5RAP1	0.002644023	
TSPAN14	0.002644023	
LLGL1	0.002644023	
CMIP	0.002644023	
DFNA5	0.002644023	
TLR8	0.002644023	
LIMCH1	0.002644023	
PLEKHG6	0.002644023	
PRKRIR	0.002644023	
KCND2	0.002644023	
ARSG	0.002644023	
LIX1L	0.002644023	
ZNF513	0.002644023	
UGDH-AS1	0.002644023	
GZF1	0.002644023	
ALG10	0.002644023	
SPPL3	0.002644023	

OTUD3	0.002644023	
SLC25A35	0.002644023	
MT1F	0.002644023	
TBC1D19	0.002644023	
PPP1R3B	0.002644023	
TG	0.002644023	
NUAK2	0.002644023	
ANKMY2	0.002644023	
ARHGEF28	0.002644023	
STAC	0.002644023	
ITM2C	0.002644023	
RPAIN	0.002644023	
RBM18	0.002644023	
GMDS	0.002644023	
PGAP1	0.002644023	
PNPT1	0.002644023	
C8orf4	0.002644023	
FAM198A	0.002644023	
SGCA	0.002644023	
FAM96A	0.002644023	
H6PD	0.002644023	
HIST1H2AG	0.002644023	
SGCE	0.002644023	
WDR3	0.002644023	
ARHGEF16	0.002644023	
MCCC1	0.002644023	
AP5S1	0.002644023	
RP11-284F21.7	0.002644023	
POLR3K	0.002644023	
CSTF3	0.002644023	
PCDHGB4	0.002644023	
CPAMD8	0.002644023	
NUP50-AS1	0.002644023	
NEUROG3	0.002644023	
FLYWCH1	0.002644023	
SLC44A1	0.002644023	
TDRD10	0.002644023	
PGPEP1	0.002644023	
NKD2	0.002644023	
GRAMD4	0.002644023	
TTLL11	0.002644023	

C17orf75	0.002644023	
PP7080	0.002644023	
HS6ST1	0.002644023	
FOXK2	0.002644023	
AC106876.2	0.002644023	
MCF2	0.002644023	
ESYT1	0.002644023	
RILPL2	0.002644023	
RPE65	0.002593176	
DDX11	0.002593176	
ISCA2	0.002593176	
ASTN2	0.002593176	
SLC4A3	0.002593176	
CNP	0.002593176	
CABYR	0.002593176	
FAM107B	0.002593176	
ZC3HC1	0.002593176	
YTHDC2	0.002593176	
ATP5SL	0.002593176	
TBC1D10C	0.002593176	
FNDC4	0.002593176	
ZNRF2	0.002593176	
TSPAN1	0.002593176	
TXNRD3	0.002593176	
AMIGO2	0.002593176	
MATN3	0.002593176	
EML3	0.002593176	
TRMT5	0.002593176	
HELQ	0.002593176	
SGCB	0.002593176	
ZC3H18	0.002593176	
TPST1	0.002593176	
PPP6R2	0.002593176	
C6orf89	0.002593176	
IDUA	0.002593176	
BTBD1	0.002593176	
FBXL12	0.002593176	
FBXO45	0.002593176	
SATB2	0.002593176	
TMED1	0.002593176	
LEFTY1	0.002593176	

IDO1	0.002593176	
MISP	0.002593176	
PRRT3-AS1	0.002593176	
LHPP	0.002593176	
ZNF264	0.002593176	
POMC	0.002593176	
ARHGEF3	0.002593176	
LINC01138	0.002593176	
SNURF	0.002593176	
GAA	0.002593176	
TREM1	0.002593176	
HAUS3	0.002593176	
TBC1D30	0.002593176	
AGTR2	0.002593176	
NAPSB	0.002593176	
MGST3	0.002593176	
NACC1	0.002593176	
ZSCAN18	0.002593176	
ALKBH6	0.002593176	
ZNF746	0.002593176	
PAEP	0.002593176	
OSBPL3	0.002593176	
ICOS	0.002593176	
SH3BP5-AS1	0.002593176	
CC2D1B	0.002593176	
BTBD6	0.002593176	
ATG10	0.002593176	
DSEL	0.002593176	
PAQR4	0.002593176	
OLMALINC	0.002593176	
TYRP1	0.002593176	
SLC25A42	0.002593176	
ZNF470	0.002593176	
TFCP2L1	0.002593176	
HAUS5	0.002593176	
HTR7P1	0.002593176	
MYRF	0.002593176	
TNFAIP2	0.002593176	
PMF1-BGLAP	0.002593176	
LTA4H	0.002593176	
UBQLN2	0.002593176	

P3H2	0.002593176	
IFIT5	0.002593176	
MYRIP	0.002593176	
SERPINI1	0.002593176	
CIDEB	0.002593176	
LRCOL1	0.002593176	
TUBD1	0.002593176	
PBLD	0.002593176	
ZC3H8	0.002593176	
NDST1	0.002593176	
NPY1R	0.002593176	
ERLIN2	0.002593176	
ZNF614	0.002593176	
FAM150B	0.002593176	
PCSK1	0.002593176	
ELMOD2	0.002593176	
TOMM5	0.002593176	
KDELC1	0.002593176	
SLC39A4	0.002593176	
OR2I1P	0.002593176	
OTUD1	0.002593176	
DYNC2LI1	0.002593176	
SCN5A	0.002593176	
GRIK5	0.002593176	
SHD	0.002593176	
FLYWCH2	0.002593176	
METAP1	0.002593176	
RRAGA	0.002593176	
IFI44L	0.002593176	
NPW	0.002593176	
ZNF606	0.002593176	
KLK2	0.002593176	
JMY	0.002593176	
FOXQ1	0.002593176	
TSPYL2	0.002593176	
FAM65B	0.002593176	
LINC00342	0.002593176	
AGAP2	0.002593176	
FUCA1	0.002593176	
TMEM167A	0.002593176	
DOK4	0.002593176	

ELMO3	0.002593176	
RP11-96D1.6	0.002593176	
RP11-923I11.6	0.002593176	
ENC1	0.002593176	
CYP3A43	0.002593176	
ASCL3	0.002593176	
RP11-96H19.1	0.002593176	
CD24	0.002593176	
PVT1	0.002593176	
IGKV1-16	0.002593176	
HLA-DQA2	0.002593176	
RIMS2	0.002593176	
ACKR1	0.002593176	
SHF	0.002593176	
PIGO	0.002593176	
TMEM38A	0.002593176	
MOSPD1	0.002593176	
TCTEX1D4	0.002593176	
CCDC91	0.002593176	
CLSTN2	0.002593176	
INTS1	0.002593176	
IRS4	0.002593176	
UGT2A3	0.00254233	
ARMCX6	0.00254233	
GMCL1P1	0.00254233	
THUMPD3-AS1	0.00254233	
RP11-231P20.2	0.00254233	
SYPL1	0.00254233	
AKAP5	0.00254233	
EMC10	0.00254233	
TSC22D2	0.00254233	
VWA9	0.00254233	
ATG13	0.00254233	
AP4S1	0.00254233	
PHYHD1	0.00254233	
PIEZO1	0.00254233	
ZNF121	0.00254233	
ELOVL5	0.00254233	
P4HA1	0.00254233	
HINFP	0.00254233	
LAPTM4B	0.00254233	

FBXO33	0.00254233	
ADAM19	0.00254233	
FBXO28	0.00254233	
LSM12	0.00254233	
PRR36	0.00254233	
SLC30A2	0.00254233	
C12orf29	0.00254233	
MOCS2	0.00254233	
KIF16B	0.00254233	
C9orf78	0.00254233	
C3orf17	0.00254233	
CPNE4	0.00254233	
ZNF433	0.00254233	
ZNF500	0.00254233	
IGHMBP2	0.00254233	
PCNP	0.00254233	
KIAA0430	0.00254233	
PCP4L1	0.00254233	
FAHD1	0.00254233	
NELFA	0.00254233	
RNF126	0.00254233	
MYH14	0.00254233	
ANGPTL4	0.00254233	
ZNF135	0.00254233	
NOG	0.00254233	
ARHGAP10	0.00254233	
PCYT1A	0.00254233	
PRMT6	0.00254233	
TRIM2	0.00254233	
METTL3	0.00254233	
ACSS1	0.00254233	
SLC17A4	0.00254233	
CARS2	0.00254233	
SMTN	0.00254233	
MOK	0.00254233	
PPP2R2C	0.00254233	
OGFOD3	0.00254233	
SPECC1L	0.00254233	
TARBP1	0.00254233	
GRIA1	0.00254233	
SUSD4	0.00254233	

C1orf198	0.00254233	
SREK1IP1	0.00254233	
DLGAP3	0.00254233	
TRMT11	0.00254233	
ZNF34	0.00254233	
MTMR12	0.00254233	
TTYH3	0.00254233	
RNLS	0.00254233	
LINC00659	0.00254233	
PIWIL4	0.00254233	
CFAP36	0.00254233	
OSTC	0.00254233	
DPH3	0.00254233	
IL23R	0.00254233	
RP11-91K9.1	0.00254233	
TRIM10	0.00254233	
UBTD2	0.00254233	
C10orf11	0.00254233	
RC3H2	0.00254233	
PAFAH2	0.00254233	
RP11-44M6.7	0.00254233	
TPD52L3	0.00254233	
ERAL1	0.00254233	
RECK	0.00254233	
OAS1	0.00254233	
RP5-1039K5.19	0.00254233	
IGHV3-48	0.00254233	
SENP8	0.00254233	
ALAS2	0.00254233	
AC009014.3	0.00254233	
C20orf96	0.00254233	
N6AMT1	0.00254233	
RRAGD	0.00254233	
FRAS1	0.00254233	
EGFLAM	0.00254233	
PCGF3	0.00254233	
GRAMD1C	0.00254233	
TNFSF15	0.00254233	
SLC35G1	0.00254233	
AGPAT5	0.00254233	
IFT81	0.00254233	

IGSF3	0.00254233	
DLGAP2	0.00254233	
TRIQK	0.00254233	
CHST10	0.00254233	
RARA-AS1	0.00254233	
DCAKD	0.00254233	
CLCN7	0.00254233	
C4orf27	0.00254233	
RP11-88E10.5	0.00254233	
PRR22	0.00254233	
KIF17	0.00254233	
WNT11	0.00254233	
U2	0.00254233	
IGKV3-15	0.00254233	
RASGRF1	0.00254233	
RIMS3	0.00254233	
PGRMC2	0.00254233	
ZNF334	0.00254233	
HEATR5A	0.00254233	
OCIAD1	0.00254233	
PKP3	0.00254233	
ACSM3	0.00254233	
P2RY11	0.00254233	
MROH6	0.00254233	
DQX1	0.00254233	
NBR2	0.00254233	
TMEM165	0.00254233	
CCDC28B	0.00254233	
GAD2	0.00254233	
ZBTB18	0.00254233	
RNF135	0.00254233	
CNKSR3	0.00254233	
TLR10	0.00254233	
CECR5	0.00254233	
FTOP1	0.00254233	
GYG2	0.00254233	
SLC25A32	0.00254233	
WNT6	0.00254233	
FAM49A	0.00254233	
CTC-360G5.9	0.00254233	
ERP27	0.00254233	

TPH1	0.00254233	
TCTN1	0.00254233	
RRAGB	0.00254233	
SLC1A4	0.00254233	
PRRT2	0.00254233	
ADAMTS6	0.00254233	
GPRC5B	0.00254233	
KIAA0319	0.002491483	
GMPR	0.002491483	
RPRD1A	0.002491483	
PLAG1	0.002491483	
PEMT	0.002491483	
ARID3B	0.002491483	
FAM72A	0.002491483	
INPP1	0.002491483	
NECAB3	0.002491483	
KRT4	0.002491483	
ANKRD44	0.002491483	
ZBTB45	0.002491483	
AC026271.5	0.002491483	
WDR35	0.002491483	
EPHB4	0.002491483	
IL17A	0.002491483	
CEP170P1	0.002491483	
ZBTB40	0.002491483	
LSM5	0.002491483	
SLC25A17	0.002491483	
APOD	0.002491483	
STON1	0.002491483	
NOVA1	0.002491483	
PREX2	0.002491483	
PARM1	0.002491483	
DLX2	0.002491483	
DPT	0.002491483	
RWDD2A	0.002491483	
GPBP1L1	0.002491483	
SLC38A9	0.002491483	
PDIA5	0.002491483	
CTD-3092A11.2	0.002491483	
GPR34	0.002491483	
ZDHHC16	0.002491483	

TM9SF4	0.002491483	
KDELC2	0.002491483	
EPS8L3	0.002491483	
KRTAP3-2	0.002491483	
NUP35	0.002491483	
POC5	0.002491483	
SNX24	0.002491483	
RAPGEF6	0.002491483	
KIAA0556	0.002491483	
CDAN1	0.002491483	
NCKAP5	0.002491483	
ASB8	0.002491483	
TRAM2-AS1	0.002491483	
VSIG10L	0.002491483	
SAMD10	0.002491483	
LDHAL6B	0.002491483	
RBM27	0.002491483	
LZTFL1	0.002491483	
NEIL1	0.002491483	
MEMO1	0.002491483	
ZNF302	0.002491483	
HIST1H2AE	0.002491483	
ACSM1	0.002491483	
MAMDC4	0.002491483	
MDM1	0.002491483	
MX2	0.002491483	
MICB	0.002491483	
ASAP3	0.002491483	
GPR114	0.002491483	
WDR59	0.002491483	
RP11-53O19.3	0.002491483	
ZSCAN16	0.002491483	
CCDC142	0.002491483	
TTC17	0.002491483	
TDP1	0.002491483	
APOO	0.002491483	
THSD4	0.002491483	
XCR1	0.002491483	
CSNK1G3	0.002491483	
LINC00667	0.002491483	
AAMDC	0.002491483	

ERO1L	0.002491483	
ZNF652	0.002491483	
RPAP3	0.002491483	
WDR36	0.002491483	
NOL4L	0.002491483	
COX18	0.002491483	
GLB1L	0.002491483	
AS3MT	0.002491483	
SLC41A3	0.002491483	
ZNF354B	0.002491483	
TMEM120A	0.002491483	
NKX3-2	0.002491483	
MLIP	0.002491483	
CASC10	0.002491483	
CH507-9B2.5	0.002491483	
BEND5	0.002491483	
SLC25A44	0.002491483	
RASGRP3	0.002491483	
SULT1A2	0.002491483	
TMEM55A	0.002491483	
SYTL4	0.002491483	
NOC3L	0.002491483	
COL13A1	0.002491483	
ZDHHC14	0.002491483	
ANTXR2	0.002491483	
GALNT10	0.002491483	
VTI1A	0.002491483	
C3orf18	0.002491483	
GPATCH2	0.002491483	
KCNQ1OT1	0.002491483	
FAM131A	0.002491483	
RABGAP1L	0.002491483	
SSX3	0.002491483	
MSL3	0.002491483	
CHDH	0.002491483	
CRBN	0.002491483	
UBAC2	0.002491483	
SAR1A	0.002491483	
WDR75	0.002491483	
INSM1	0.002491483	
CLPTM1L	0.002491483	

RP11-285F7.2	0.002491483	
CLEC12A	0.002491483	
CTD-2583A14.8	0.002491483	
SGCD	0.002491483	
NOP2	0.002491483	
GOLGA8B	0.002491483	
ZNF761	0.002491483	
C9orf116	0.002491483	
MNS1	0.002491483	
SYBU	0.002491483	
NUDCD3	0.002491483	
SRPX2	0.002491483	
RP11-41O4.1	0.002491483	
CELF4	0.002491483	
TAPT1	0.002491483	
COA7	0.002491483	
CBLN4	0.002491483	
THAP10	0.002491483	
LINC01564	0.002491483	
ADAMTS13	0.002491483	
PGAM5	0.002491483	
KCNQ3	0.002491483	
MAFG-AS1	0.002491483	
APLF	0.002491483	
S100A16	0.002491483	
RAB29	0.002491483	
USH2A	0.002491483	
C12orf73	0.002491483	
MC4R	0.002491483	
IFT74	0.002491483	
SLC16A11	0.002440637	
CCDC116	0.002440637	
TMEM151A	0.002440637	
SRGAP1	0.002440637	
TIGD6	0.002440637	
NBPF9	0.002440637	
TSPAN7	0.002440637	
DPP3	0.002440637	
IL11	0.002440637	
TSPAN9	0.002440637	
FRK	0.002440637	

OSMR	0.002440637	
ZNF10	0.002440637	
PIGX	0.002440637	
EMC9	0.002440637	
MEP1B	0.002440637	
GMNC	0.002440637	
IAPP	0.002440637	
GANC	0.002440637	
CIRBP-AS1	0.002440637	
NEURL2	0.002440637	
MRI1	0.002440637	
COLGALT1	0.002440637	
RPF2	0.002440637	
GTF2A1L	0.002440637	
BICD1	0.002440637	
AASDH	0.002440637	
RBMY1F	0.002440637	
MT1M	0.002440637	
LY75	0.002440637	
RP11-488L18.4	0.002440637	
ZNF213	0.002440637	
OSBPL6	0.002440637	
FASTKD3	0.002440637	
RYR3	0.002440637	
OTOP2	0.002440637	
KB-431C1.4	0.002440637	
GEN1	0.002440637	
KCTD14	0.002440637	
OXER1	0.002440637	
AGGF1	0.002440637	
CDKL3	0.002440637	
ESYT2	0.002440637	
DMKN	0.002440637	
PADI2	0.002440637	
TRAPPC12	0.002440637	
CKMT2-AS1	0.002440637	
CACNB2	0.002440637	
SPTLC3	0.002440637	
PLD3	0.002440637	
APOBEC3F	0.002440637	
IGIP	0.002440637	

CBX3P2	0.002440637	
PLA2G15	0.002440637	
RP1-152L7.5	0.002440637	
SYN2	0.002440637	
RP11-863K10.7	0.002440637	
EXD2	0.002440637	
DGCR8	0.002440637	
SP140L	0.002440637	
RIOK1	0.002440637	
NUDT16P1	0.002440637	
DNAJC9	0.002440637	
DHFRL1	0.002440637	
ZNF30	0.002440637	
APMAP	0.002440637	
EFCAB7	0.002440637	
CDV3	0.002440637	
TMTC1	0.002440637	
LTBP3	0.002440637	
SLC22A23	0.002440637	
HEATR3	0.002440637	
HENMT1	0.002440637	
C15orf52	0.002440637	
VNN3	0.002440637	
DENND1B	0.002440637	
TMEM26	0.002440637	
GUCY2F	0.002440637	
CLTCL1	0.002440637	
PIBF1	0.002440637	
CXorf38	0.002440637	
CKMT2	0.002440637	
GVINP1	0.002440637	
AK4	0.002440637	
CACNA1B	0.002440637	
C11orf74	0.002440637	
TMEM198	0.002440637	
RAB12	0.002440637	
ASB7	0.002440637	
EMID1	0.002440637	
LRP2BP	0.002440637	
TRPS1	0.002440637	
SPAG4	0.002440637	

KIF22	0.002440637	
LINC00346	0.002440637	
NMRAL1	0.002440637	
SNHG1	0.002440637	
EVPL	0.002440637	
AP000355.2	0.002440637	
UGCG	0.002440637	
FBXO8	0.002440637	
TTC21A	0.002440637	
TP53INP2	0.002440637	
EIF3J-AS1	0.002440637	
DDAH1	0.002440637	
BCL7B	0.002440637	
GM2A	0.002440637	
LYRM1	0.002440637	
RP11-799B12.1	0.002440637	
C9orf69	0.002440637	
AQP6	0.002440637	
FAM154A	0.002440637	
OGFOD2	0.002440637	
TOR3A	0.002440637	
PLIN4	0.002440637	
CSRP2BP	0.002440637	
SLC15A4	0.002440637	
SLC22A31	0.002440637	
ARSJ	0.002440637	
WDR55	0.002440637	
FSD1L	0.002440637	
CEP162	0.002440637	
ABCA4	0.002440637	
CCDC148	0.002440637	
TRPM8	0.002440637	
CARD6	0.002440637	
KRT80	0.002440637	
SLC13A2	0.002440637	
MICALL1	0.002440637	
ZNF565	0.002440637	
SZT2	0.002440637	
LARP6	0.002440637	
PAPD5	0.002440637	
RPH3A	0.002440637	

PLEKHA8	0.002440637	
DCC	0.002440637	
ERC2	0.002440637	
KIAA0586	0.002440637	
VPS13D	0.002440637	
KIAA0195	0.002440637	
RNF24	0.002440637	
RASSF4	0.002440637	
SOBP	0.002440637	
TPST2	0.002440637	
JADE3	0.002440637	
GPX6	0.002440637	
IPO11	0.002440637	
MPI	0.002440637	
DCTN5	0.002440637	
TRIM55	0.002440637	
PLD6	0.002440637	
GGTA1P	0.002440637	
CRHR2	0.002440637	
PGAP3	0.002440637	
PPIAP22	0.002440637	
WNT10A	0.002440637	
TGFBR3L	0.002440637	
TLDC2	0.002440637	
SNN	0.002440637	
COL23A1	0.002440637	
HIST1H2BN	0.002440637	
TMEM234	0.002440637	
GORAB	0.002440637	
DESI1	0.00238979	
PTPRN2	0.00238979	
FZD6	0.00238979	
NEFH	0.00238979	
CA13	0.00238979	
CHRNB4	0.00238979	
EMP2	0.00238979	
NOL10	0.00238979	
DOCK6	0.00238979	
RMDN2	0.00238979	
HIST1H2AM	0.00238979	
ARGLU1	0.00238979	

COQ10B	0.00238979	
RNF207	0.00238979	
ZNF461	0.00238979	
WDR12	0.00238979	
C15orf62	0.00238979	
CAPN5	0.00238979	
SLC30A5	0.00238979	
C12orf65	0.00238979	
KIAA0232	0.00238979	
SLC22A3	0.00238979	
SNTG1	0.00238979	
HOXB-AS3	0.00238979	
FUT10	0.00238979	
EPHB1	0.00238979	
MOCS3	0.00238979	
TMEM105	0.00238979	
REEP4	0.00238979	
RNPC3	0.00238979	
CNIH1	0.00238979	
SP6	0.00238979	
RP13-516M14.1	0.00238979	
RP11-166D19.1	0.00238979	
SLC30A7	0.00238979	
KRI1	0.00238979	
GATS	0.00238979	
TBX6	0.00238979	
PIH1D2	0.00238979	
ARX	0.00238979	
GRIK2	0.00238979	
FAM180A	0.00238979	
STARD7	0.00238979	
EPS8L2	0.00238979	
PPM1L	0.00238979	
PAQR6	0.00238979	
ZFP2	0.00238979	
NLRP1	0.00238979	
SNX29	0.00238979	
FAM83G	0.00238979	
MFSD2A	0.00238979	
RNF103-CHMP3	0.00238979	
WBSCR17	0.00238979	

CROCCP2	0.00238979	
DPH7	0.00238979	
KCNC1	0.00238979	
DNASE1L1	0.00238979	
VARS2	0.00238979	
HRC	0.00238979	
KATNAL1	0.00238979	
RGS9	0.00238979	
PDPR	0.00238979	
ITGB1BP2	0.00238979	
LBX2	0.00238979	
RWDD3	0.00238979	
RGMA	0.00238979	
ARL14	0.00238979	
MAPK15	0.00238979	
SETMAR	0.00238979	
GBA2	0.00238979	
MYNN	0.00238979	
IMMP2L	0.00238979	
TIAL1	0.00238979	
CASR	0.00238979	
TOR1B	0.00238979	
TTC30B	0.00238979	
TRPV6	0.00238979	
PCMTD1	0.00238979	
HS3ST5	0.00238979	
MBNL2	0.00238979	
ZNF672	0.00238979	
FXYD3	0.00238979	
RP11-254F7.2	0.00238979	
CA5B	0.00238979	
ABT1	0.00238979	
CYB561	0.00238979	
PHF7	0.00238979	
CEL	0.00238979	
OPRD1	0.00238979	
CTC-444N24.8	0.00238979	
ABHD3	0.00238979	
GRIP2	0.00238979	
SNX8	0.00238979	
PODXL2	0.00238979	

AC007405.6	0.00238979	
NSUN3	0.00238979	
TP53TG1	0.00238979	
TCEAL4	0.00238979	
RP11-81H14.2	0.00238979	
TMEM63A	0.00238979	
MYL3	0.00238979	
ZNF2	0.00238979	
LPGAT1	0.00238979	
CRCT1	0.00238979	
MAGEB2	0.00238979	
PLOD2	0.00238979	
GSAP	0.00238979	
MYO1D	0.00238979	
RP11-39H3.2	0.00238979	
LIPT2	0.00238979	
C17orf70	0.00238979	
AK9	0.00238979	
STX16-NPEPL1	0.00238979	
EVA1A	0.00238979	
CRIPT	0.00238979	
C1orf210	0.00238979	
RP11-1148L6.5	0.00238979	
HBD	0.00238979	
CCDC64	0.00238979	
KDM8	0.00238979	
C9orf85	0.00238979	
ARPP21	0.00238979	
MYPOP	0.00238979	
YBX2	0.00238979	
LUZP4	0.00238979	
MYBPC2	0.00238979	
IL7	0.00238979	
LAMP2	0.00238979	
GLUD2	0.00238979	
UTP15	0.00238979	
SLC6A16	0.00238979	
CASKIN2	0.00238979	
ZNF620	0.00238979	
MKS1	0.00238979	
AKAP3	0.00238979	

TXNDC15	0.00238979	
IQUB	0.00238979	
CD109	0.00238979	
TTLL12	0.00238979	
MTRR	0.00238979	
VSTM2L	0.00238979	
LINC01503	0.00238979	
AFG3L2	0.00238979	
BIVM	0.00238979	
DDX31	0.00238979	
AC004447.2	0.00238979	
FAM131C	0.00238979	
LRRC4C	0.00238979	
MGME1	0.00238979	
SEPSECS-AS1	0.00238979	
IDNK	0.00238979	
TEKT4	0.00238979	
MTURN	0.00238979	
CEP128	0.00238979	
GSTM4	0.00238979	
CDK10	0.00238979	
POM121C	0.00238979	
RGS7	0.002338943	
LANCL1	0.002338943	
LGALSL	0.002338943	
ZNF708	0.002338943	
CNPPD1	0.002338943	
ELSPBP1	0.002338943	
ZNF439	0.002338943	
SLC4A1	0.002338943	
TP53I3	0.002338943	
TRDN	0.002338943	
TMEM91	0.002338943	
SYTL5	0.002338943	
TMEM237	0.002338943	
EIF1AX	0.002338943	
RPL7L1	0.002338943	
RP11-344P13.4	0.002338943	
PLN	0.002338943	
GTF2IRD2B	0.002338943	
TNRC6C-AS1	0.002338943	

CTNNAL1	0.002338943	
TTC30A	0.002338943	
ANKRD33B	0.002338943	
RHBDL1	0.002338943	
RP11-620J15.3	0.002338943	
RP11-465B22.3	0.002338943	
SHROOM2	0.002338943	
PABPC3	0.002338943	
NLRP6	0.002338943	
SAP30L	0.002338943	
CLCN6	0.002338943	
C9orf91	0.002338943	
ANKRD35	0.002338943	
C19orf47	0.002338943	
PLCZ1	0.002338943	
KRT81	0.002338943	
COQ7	0.002338943	
WWC1	0.002338943	
C2orf42	0.002338943	
AMIGO1	0.002338943	
LRRC25	0.002338943	
AC009133.12	0.002338943	
RCAN3	0.002338943	
CAPRIN2	0.002338943	
CCSAP	0.002338943	
FUZ	0.002338943	
BCS1L	0.002338943	
IGHV3-73	0.002338943	
RP11-337C18.8	0.002338943	
CEACAM19	0.002338943	
PLBD1	0.002338943	
CTNS	0.002338943	
PCDHA4	0.002338943	
SLC25A14	0.002338943	
AQP5	0.002338943	
RAB33B	0.002338943	
HORMAD2	0.002338943	
ZNF823	0.002338943	
CCL11	0.002338943	
SOAT2	0.002338943	
COX8C	0.002338943	

POC1B	0.002338943	
CRTC3	0.002338943	
SOCS2-AS1	0.002338943	
C12orf66	0.002338943	
MAP9	0.002338943	
TLR1	0.002338943	
GRM1	0.002338943	
FSHR	0.002338943	
ZNF449	0.002338943	
TRIO	0.002338943	
MORN2	0.002338943	
LRTOMT	0.002338943	
XPNPEP3	0.002338943	
RASL10B	0.002338943	
MTO1	0.002338943	
MT1H	0.002338943	
RUFY2	0.002338943	
HCG4P7	0.002338943	
FAM20C	0.002338943	
CASZ1	0.002338943	
SPAG8	0.002338943	
YTHDF1	0.002338943	
OSER1-AS1	0.002338943	
SNX30	0.002338943	
RP11-46D6.1	0.002338943	
CYP2F1	0.002338943	
CSF2RA	0.002338943	
RP11-81A1.6	0.002338943	
SLC18B1	0.002338943	
GNG12	0.002338943	
GRIPAP1	0.002338943	
GCFC2	0.002338943	
LPP-AS2	0.002338943	
RBM24	0.002338943	
C19orf52	0.002338943	
DHRSX	0.002338943	
ECEL1	0.002338943	
YRDC	0.002338943	
ZNF226	0.002338943	
GNAT1	0.002338943	
C11orf49	0.002338943	

METTL12	0.002338943	
HSPA12A	0.002338943	
CROCC	0.002338943	
OFD1	0.002338943	
PINK1	0.002338943	
ZNF354C	0.002338943	
SAMD8	0.002338943	
LRRC31	0.002338943	
NAP1L2	0.002338943	
AC092066.1	0.002338943	
TMCO3	0.002338943	
ATP6V0A2	0.002338943	
PYROXD1	0.002338943	
GRM2	0.002338943	
SPA17	0.002338943	
EHD3	0.002338943	
COMMD8	0.002338943	
SYDE2	0.002338943	
ADCY8	0.002338943	
KCNJ4	0.002338943	
ZNF697	0.002338943	
SHISA5	0.002338943	
TMEM189-UBE2V1	0.002338943	
LILRA2	0.002338943	
CTD-2583A14.11	0.002338943	
FLVCR2	0.002338943	
PPP1R1A	0.002338943	
EMX2	0.002338943	
LAMP3	0.002338943	
ZNF180	0.002338943	
CEP85L	0.002338943	
ZEB2-AS1	0.002338943	
HSD17B11	0.002338943	
RP11-1069G10.1	0.002338943	
PLEKHM1	0.002338943	
SHISA4	0.002338943	
ADAMTSL3	0.002338943	
SLC35D2	0.002338943	
DUSP13	0.002338943	
RP11-660L16.2	0.002338943	
SLC35A2	0.002338943	

NOP14	0.002338943	
METTL15	0.002338943	
ITGA8	0.002338943	
MDGA1	0.002338943	
SSTR1	0.002338943	
FAM64A	0.002338943	
PTH	0.002288097	
TMEM18	0.002288097	
TAS1R3	0.002288097	
ROBO3	0.002288097	
A1BG-AS1	0.002288097	
RP3-467N11.1	0.002288097	
FAM98C	0.002288097	
C14orf132	0.002288097	
OGDHL	0.002288097	
HCN2	0.002288097	
ANGPTL1	0.002288097	
RP11-390F4.3	0.002288097	
C1RL	0.002288097	
DGKI	0.002288097	
SLC2A5	0.002288097	
TMEM117	0.002288097	
TOR2A	0.002288097	
NMD3	0.002288097	
BCAS1	0.002288097	
PPIP5K1	0.002288097	
MTMR1	0.002288097	
ADAMTSL5	0.002288097	
PNPLA3	0.002288097	
PIK3C2B	0.002288097	
RP11-876N24.3	0.002288097	
PIDD1	0.002288097	
IFT52	0.002288097	
CAMP	0.002288097	
CTU1	0.002288097	
PRDM16	0.002288097	
N4BP1	0.002288097	
KIF3B	0.002288097	
NDC1	0.002288097	
CTDSPL	0.002288097	
TMEM183A	0.002288097	

C19orf12	0.002288097	
MTRF1	0.002288097	
PPP1R3C	0.002288097	
RBPMS2	0.002288097	
CACNA1A	0.002288097	
HIST1H2BL	0.002288097	
GLIS2	0.002288097	
OPRL1	0.002288097	
BST1	0.002288097	
SLC33A1	0.002288097	
ZNF628	0.002288097	
UCKL1	0.002288097	
PYCR1	0.002288097	
SLC30A4	0.002288097	
SHE	0.002288097	
HLA-DRB6	0.002288097	
S100A14	0.002288097	
DYNLL1-AS1	0.002288097	
CORO2B	0.002288097	
OBSL1	0.002288097	
SNX21	0.002288097	
ZP3	0.002288097	
MEGF6	0.002288097	
C3orf58	0.002288097	
STARD3NL	0.002288097	
DHX37	0.002288097	
KCTD21-AS1	0.002288097	
CTC1	0.002288097	
ZC3H7A	0.002288097	
SLC12A9	0.002288097	
RNASE1	0.002288097	
CSTF2T	0.002288097	
GABRA1	0.002288097	
ENOX1	0.002288097	
LINC00671	0.002288097	
COX6A2	0.002288097	
MORC4	0.002288097	
ABHD2	0.002288097	
CUTC	0.002288097	
CRYBB1	0.002288097	
TTC39C	0.002288097	

ZCCHC3	0.002288097	
PLEKHN1	0.002288097	
AC016768.1	0.002288097	
GOLT1B	0.002288097	
RUFY3	0.002288097	
C15orf40	0.002288097	
HCN4	0.002288097	
IGKV1-17	0.002288097	
TRIM62	0.002288097	
S1PR5	0.002288097	
ZFAND1	0.002288097	
TRIM66	0.002288097	
TMEM190	0.002288097	
MLH3	0.002288097	
CH17-189H20.1	0.002288097	
CLDN15	0.002288097	
ARTN	0.002288097	
INF2	0.002288097	
PRRG1	0.002288097	
VIL1	0.002288097	
SWAP70	0.002288097	
ANKRD9	0.002288097	
RP4-564F22.6	0.002288097	
TNIP3	0.002288097	
ZNF700	0.002288097	
B3GNT9	0.002288097	
MAB21L2	0.002288097	
F8A1	0.002288097	
ZNF766	0.002288097	
LINC00674	0.002288097	
ZNF230	0.002288097	
TNFRSF10C	0.002288097	
BTBD16	0.002288097	
ASAH2	0.002288097	
CTB-129P6.7	0.002288097	
KCNJ12	0.002288097	
DCBLD1	0.002288097	
PHTF1	0.002288097	
MCUR1	0.002288097	
PDCD7	0.002288097	
DNMBP-AS1	0.002288097	

CTSW	0.002288097	
GATA6-AS1	0.002288097	
HOXA4	0.002288097	
RAVER2	0.002288097	
ZNF814	0.002288097	
ZNF497	0.002288097	
IL20RA	0.002288097	
PUS7	0.002288097	
AKR1C8P	0.002288097	
ZNF44	0.002288097	
KEL	0.002288097	
PAIP1	0.002288097	
NPTX1	0.002288097	
IFRD1	0.002288097	
LRP1B	0.002288097	
KIAA0020	0.002288097	
ZNF441	0.002288097	
CPOX	0.002288097	
PNPLA6	0.002288097	
OPN3	0.002288097	
TMX4	0.002288097	
GPT2	0.002288097	
LEP	0.002288097	
TBCK	0.002288097	
AFG3L1P	0.002288097	
MDH1B	0.002288097	
KIFC2	0.002288097	
CTD-2545H1.2	0.002288097	
SLC25A51	0.002288097	
RELN	0.002288097	
FBXL13	0.002288097	
MANBAL	0.002288097	
LPIN2	0.002288097	
PCDHB14	0.002288097	
AMD1	0.002288097	
APOBEC1	0.002288097	
NDFIP2	0.002288097	
YOD1	0.002288097	
SIK1	0.002288097	
CLCN2	0.002288097	
CACNB1	0.002288097	

NRDE2	0.002288097	
ERMARD	0.002288097	
TNR	0.002288097	
ZNF234	0.002288097	
TTLL5	0.002288097	
CGREF1	0.002288097	
PCDHB13	0.002288097	
FAM134C	0.002288097	
C18orf25	0.002288097	
UGT3A1	0.002288097	
SSTR5	0.002288097	
ZNF252P	0.002288097	
TUBA8	0.002288097	
WDSUB1	0.002288097	
MFF	0.002288097	
FAM98A	0.002288097	
RP11-258C19.7	0.002288097	
PDE6C	0.002288097	
ACOT11	0.002288097	
RBP7	0.002288097	
TBC1D2B	0.002288097	
CRAT	0.002288097	
RP4-758J18.2	0.002288097	
SDR39U1	0.002288097	
RAMP1	0.002288097	
POLR3G	0.002288097	
ADPRM	0.002288097	
XCL1	0.002288097	
B4GALNT4	0.002288097	
NPNT	0.002288097	
DIO2	0.002288097	
SEMA3E	0.002288097	
RFTN1	0.002288097	
EPT1	0.00223725	
FABP7	0.00223725	
SCUBE1	0.00223725	
TMEM74	0.00223725	
LDHAL6A	0.00223725	
PLA2G7	0.00223725	
AC079922.3	0.00223725	
MSH5	0.00223725	

THEM5	0.00223725	
COLEC10	0.00223725	
AVPR1A	0.00223725	
PSKH1	0.00223725	
GFRA2	0.00223725	
CCDC170	0.00223725	
NELFCD	0.00223725	
KIZ	0.00223725	
ZNF557	0.00223725	
THAP9	0.00223725	
QSOX2	0.00223725	
VMO1	0.00223725	
CIB3	0.00223725	
CNNM1	0.00223725	
LRRC27	0.00223725	
TBC1D1	0.00223725	
MEFV	0.00223725	
LRRFIP2	0.00223725	
ZNF503-AS1	0.00223725	
MTMR10	0.00223725	
NLN	0.00223725	
RASA4	0.00223725	
FOXL2	0.00223725	
EFCAB11	0.00223725	
SLC38A7	0.00223725	
ZNF821	0.00223725	
FCHSD1	0.00223725	
LRRC16B	0.00223725	
TMEM147-AS1	0.00223725	
SHOX2	0.00223725	
DUSP19	0.00223725	
BIK	0.00223725	
CYP4F12	0.00223725	
LSG1	0.00223725	
LRRC17	0.00223725	
CAMK2N2	0.00223725	
CCL15-CCL14	0.00223725	
IGHV3-21	0.00223725	
CELF3	0.00223725	
HAUS4	0.00223725	
TRPC2	0.00223725	

PAPD4	0.00223725	
IZUMO4	0.00223725	
HNRNPUL2-BSCL2	0.00223725	
PVRL4	0.00223725	
ZNF607	0.00223725	
XPNPEP1	0.00223725	
HSPE1-MOB4	0.00223725	
LPPR2	0.00223725	
TMEM140	0.00223725	
DTWD2	0.00223725	
ANKH	0.00223725	
CADPS2	0.00223725	
ATP13A1	0.00223725	
ARMC9	0.00223725	
IGLV1-47	0.00223725	
HIST1H2AB	0.00223725	
STAG3L5P	0.00223725	
RNF144B	0.00223725	
IGHV3-15	0.00223725	
ECHDC1	0.00223725	
LPHN2	0.00223725	
GAS2L3	0.00223725	
SPTSSA	0.00223725	
CCDC88C	0.00223725	
PRRG2	0.00223725	
SPIRE1	0.00223725	
ZNF227	0.00223725	
UBE2QL1	0.00223725	
UCN2	0.00223725	
SCG5	0.00223725	
CCT6P3	0.00223725	
MIR4664	0.00223725	
TBX5	0.00223725	
RHPN1	0.00223725	
FAM160B1	0.00223725	
LINC01137	0.00223725	
LMNTD2	0.00223725	
RBMXL1	0.00223725	
FBXO32	0.00223725	
KCNJ16	0.00223725	
SLC35F6	0.00223725	

IL36RN	0.00223725	
KHNYN	0.00223725	
PNKD	0.00223725	
EME2	0.00223725	
GTDC1	0.00223725	
XK	0.00223725	
TMEM248	0.00223725	
SKA2	0.00223725	
IL17RE	0.00223725	
WBP1L	0.00223725	
PWAR6	0.00223725	
CHGA	0.00223725	
MGAM	0.00223725	
PHF14	0.00223725	
POMT2	0.00223725	
MTX3	0.00223725	
CAPN3	0.00223725	
AC013275.2	0.00223725	
MUM1	0.00223725	
ASRGL1	0.00223725	
BCO2	0.00223725	
ACTR1B	0.00223725	
CD5L	0.00223725	
FBXL20	0.00223725	
CAV3	0.00223725	
RP5-1021I20.1	0.00223725	
CBX6	0.00223725	
ZNF616	0.00223725	
RP3-368A4.5	0.00223725	
ULBP2	0.00223725	
CDH3	0.00223725	
ELAVL3	0.00223725	
ITPK1	0.00223725	
CPLX1	0.00223725	
SYT5	0.00223725	
TM2D1	0.00223725	
NKX2-3	0.00223725	
KIAA1147	0.00223725	
BLOC1S5	0.00223725	
SV2A	0.00223725	
SUSD1	0.00223725	

HIST1H2AI	0.00223725	
DUSP18	0.00223725	
C10orf32	0.00223725	
LOXL4	0.00223725	
MYEF2	0.00223725	
SLC36A1	0.00223725	
ZDHHC2	0.00223725	
RHOU	0.00223725	
MADCAM1	0.00223725	
CALCR	0.00223725	
THG1L	0.00223725	
EPB41L4B	0.00223725	
SLC12A7	0.00223725	
URB1	0.00223725	
NUDT12	0.00223725	
TTC25	0.00223725	
SERINC2	0.00223725	
SERPINB2	0.00223725	
MB21D2	0.002186404	
GSTM3	0.002186404	
ZDHHC3	0.002186404	
DDX51	0.002186404	
P4HA2	0.002186404	
SOWAHC	0.002186404	
NSMAF	0.002186404	
INPP5A	0.002186404	
FBXO18	0.002186404	
FGF14-AS2	0.002186404	
AVPR2	0.002186404	
C15orf48	0.002186404	
GPER1	0.002186404	
ENHO	0.002186404	
EXTL2	0.002186404	
RNASEH1	0.002186404	
ADRA1D	0.002186404	
U6	0.002186404	
KIAA0895	0.002186404	
HTR4	0.002186404	
SNX14	0.002186404	
SP8	0.002186404	
SLC45A3	0.002186404	

JAZF1	0.002186404	
BCAS3	0.002186404	
LINC00884	0.002186404	
B3GALNT1	0.002186404	
ZNF566	0.002186404	
FAM216A	0.002186404	
TRA2A	0.002186404	
N6AMT2	0.002186404	
KRCC1	0.002186404	
RFT1	0.002186404	
TWISTNB	0.002186404	
RIN3	0.002186404	
SOX9-AS1	0.002186404	
RP11-111M22.3	0.002186404	
PRDM15	0.002186404	
ADPGK	0.002186404	
AC093673.5	0.002186404	
RP11-61K9.3	0.002186404	
ZSWIM3	0.002186404	
RP11-54C4.3	0.002186404	
RP11-462L8.1	0.002186404	
COX19	0.002186404	
CYP4B1	0.002186404	
AFF3	0.002186404	
TSTD1	0.002186404	
MYOM2	0.002186404	
LAMA5-AS1	0.002186404	
TMEM19	0.002186404	
GPR153	0.002186404	
GALNT1	0.002186404	
ZNF114	0.002186404	
TAC1	0.002186404	
MPLKIP	0.002186404	
PCYT1B	0.002186404	
LARGE	0.002186404	
ADSSL1	0.002186404	
UBE2Q2P6	0.002186404	
SLC29A3	0.002186404	
ZCCHC9	0.002186404	
RPS2P46	0.002186404	
PABPC1L	0.002186404	

RP11-421F16.3	0.002186404	
GSE1	0.002186404	
HCN1	0.002186404	
TMBIM1	0.002186404	
REG3A	0.002186404	
ZNF443	0.002186404	
RSAD1	0.002186404	
GCA	0.002186404	
IQSEC1	0.002186404	
ZNF415	0.002186404	
ADO	0.002186404	
TMEM143	0.002186404	
BLOC1S4	0.002186404	
RP11-400F19.6	0.002186404	
IL1RAPL1	0.002186404	
ZNF784	0.002186404	
ZNF224	0.002186404	
ZFAT	0.002186404	
ANKS6	0.002186404	
RGSL1	0.002186404	
LINC01089	0.002186404	
GSDMB	0.002186404	
KDELR2	0.002186404	
SUV420H2	0.002186404	
TMEM45B	0.002186404	
MTND2P28	0.002186404	
C8orf37	0.002186404	
FGD5	0.002186404	
EVC2	0.002186404	
LRP10	0.002186404	
PDE6A	0.002186404	
KLHL18	0.002186404	
C14orf93	0.002186404	
LMF2	0.002186404	
TRAPPC13	0.002186404	
ZC2HC1C	0.002186404	
NIPAL3	0.002186404	
C1orf159	0.002186404	
NMUR1	0.002186404	
RP11-284F21.9	0.002186404	
SPICE1	0.002186404	

TSPAN31	0.002186404	
EPDR1	0.002186404	
SCN1A	0.002186404	
INPP5J	0.002186404	
SLC6A8	0.002186404	
LRRTM2	0.002186404	
TOMM34	0.002186404	
FBXO9	0.002186404	
CDH16	0.002186404	
ARHGEF40	0.002186404	
CALCA	0.002186404	
HPCA	0.002186404	
RAB38	0.002186404	
CEP68	0.002186404	
KLHL3	0.002186404	
MTMR3	0.002186404	
SAYSD1	0.002186404	
UXS1	0.002186404	
VGLL1	0.002186404	
EBF4	0.002186404	
STT3B	0.002186404	
GRHL2	0.002186404	
RP11-101E13.5	0.002186404	
ATP6V1E2	0.002186404	
ZNF319	0.002186404	
GBX2	0.002186404	
MMP12	0.002186404	
SNCB	0.002186404	
NBAS	0.002186404	
RP11-553L6.5	0.002186404	
ATAD1	0.002186404	
SULT1A3	0.002186404	
WBP1	0.002186404	
RGS17	0.002186404	
TPM3P9	0.002186404	
UTF1	0.002186404	
GALM	0.002186404	
LACTB2	0.002186404	
HUNK	0.002186404	
MIS18A	0.002186404	
NTN4	0.002186404	

GPR126	0.002186404	
CCDC149	0.002186404	
ARHGAP24	0.002186404	
UTS2	0.002186404	
FAM229B	0.002186404	
FAM46C	0.002186404	
HIST1H2BB	0.002186404	
LGI4	0.002186404	
DOPEY2	0.002135557	
MYH1	0.002135557	
CHURC1	0.002135557	
ADRA2B	0.002135557	
COL17A1	0.002135557	
FGF8	0.002135557	
RP11-750B16.1	0.002135557	
WDFY2	0.002135557	
ABCG4	0.002135557	
ENTHD2	0.002135557	
ZMPSTE24	0.002135557	
DCAF15	0.002135557	
DNTT	0.002135557	
TMEM55B	0.002135557	
TMED3	0.002135557	
BIRC7	0.002135557	
ADH7	0.002135557	
PLA2R1	0.002135557	
ATE1	0.002135557	
ZNF780B	0.002135557	
PHOSPHO1	0.002135557	
PCSK2	0.002135557	
RP11-390F4.6	0.002135557	
SAPCD2	0.002135557	
CXCL13	0.002135557	
CCDC43	0.002135557	
KRTAP9-4	0.002135557	
RP11-1260E13.2	0.002135557	
KIAA1143	0.002135557	
NEB	0.002135557	
NXN	0.002135557	
CYSTM1	0.002135557	
GPR158	0.002135557	

UFSP2	0.002135557	
MIER2	0.002135557	
MIR4653	0.002135557	
CCDC15	0.002135557	
CRYAA	0.002135557	
LINC01268	0.002135557	
IRX3	0.002135557	
C20orf195	0.002135557	
SNHG10	0.002135557	
KRT23	0.002135557	
KRT17	0.002135557	
PLEKHA3	0.002135557	
PSMG1	0.002135557	
TMEM106C	0.002135557	
NMNAT3	0.002135557	
BGLAP	0.002135557	
BRI3BP	0.002135557	
PPAP2A	0.002135557	
MGAT3	0.002135557	
SRD5A3	0.002135557	
AK2	0.002135557	
CWC25	0.002135557	
SLC7A5	0.002135557	
PPM1K	0.002135557	
LCN12	0.002135557	
EFHD2	0.002135557	
FRMPD1	0.002135557	
TREX2	0.002135557	
MYBPH	0.002135557	
IRX5	0.002135557	
SLC8B1	0.002135557	
ZNF721	0.002135557	
SLC16A14	0.002135557	
HOTAIRM1	0.002135557	
MASTL	0.002135557	
GNB1L	0.002135557	
HSF2BP	0.002135557	
BMP8B	0.002135557	
DOK6	0.002135557	
CD58	0.002135557	
FAM84B	0.002135557	

PCDHGB5	0.002135557	
BHLHB9	0.002135557	
EDEM2	0.002135557	
RP11-867G23.8	0.002135557	
THAP7-AS1	0.002135557	
FBRSL1	0.002135557	
ROR1	0.002135557	
MSANTD4	0.002135557	
MSI1	0.002135557	
ST7-OT4	0.002135557	
GNG3	0.002135557	
UNC50	0.002135557	
EIF2AK4	0.002135557	
FRY	0.002135557	
GRAP	0.002135557	
GRIN3A	0.002135557	
TPGS1	0.002135557	
MPV17L	0.002135557	
FAM63A	0.002135557	
DBNDD2	0.002135557	
ZNF671	0.002135557	
SRP14-AS1	0.002135557	
IGHV4-39	0.002135557	
HAUS8	0.002135557	
ZNF599	0.002135557	
TTL	0.002135557	
GAST	0.002135557	
LINC00461	0.002135557	
IGHV4-31	0.002135557	
SPAG1	0.002135557	
CILP	0.002135557	
RP11-244O19.1	0.002135557	
SMAP1	0.002135557	
TRIM68	0.002135557	
LCMT2	0.002135557	
LHB	0.002135557	
COL4A6	0.002135557	
MRC1	0.002135557	
IQCC	0.002135557	
ZNF77	0.002135557	
TNNI3K	0.002135557	

C6orf47	0.002135557	
RNF145	0.002135557	
HIST1H4L	0.002135557	
CCDC113	0.002135557	
SUV420H1	0.002135557	
CXXC4	0.002135557	
UQCRHL	0.002135557	
TGM4	0.002135557	
SGMS2	0.002135557	
ADAM22	0.002135557	
STOX1	0.002135557	
BRD9	0.002135557	
GZMH	0.002135557	
CHCHD2P9	0.002135557	
ASB1	0.002135557	
ZNF544	0.002135557	
FAM171B	0.002135557	
ZNF662	0.002135557	
PYY	0.002135557	
ANKRD13B	0.002135557	
FBXO22	0.002135557	
ZNF419	0.002135557	
EIF2D	0.002135557	
CSH1	0.002135557	
IL13RA2	0.002135557	
PIM3	0.002135557	
SUGP2	0.002135557	
PRL	0.002135557	
CTB-193M12.5	0.002135557	
ARMCX3	0.002135557	
LRRC75A-AS1	0.002135557	
LMBR1L	0.002135557	
RP11-572P18.1	0.002135557	
ZNF316	0.002135557	
PIP5K1B	0.002135557	
ZCCHC2	0.002135557	
CENPV	0.002135557	
KIAA1462	0.002135557	
SLC22A15	0.002135557	
RGS5	0.002135557	
TTC8	0.002135557	

CNPY3	0.002135557	
SMIM24	0.002135557	
DDHD1	0.002135557	
PITPNB	0.002135557	
APOBEC3D	0.00208471	
FAM47E	0.00208471	
CES1P1	0.00208471	
HIST2H4B	0.00208471	
TMEM8B	0.00208471	
FAM136A	0.00208471	
CTPS2	0.00208471	
SMARCAL1	0.00208471	
SPATA25	0.00208471	
AK7	0.00208471	
LDOC1L	0.00208471	
RP11-864N7.2	0.00208471	
IGHV4-34	0.00208471	
CCDC150	0.00208471	
FGF21	0.00208471	
ZNF485	0.00208471	
APOL3	0.00208471	
ZNF701	0.00208471	
SAAL1	0.00208471	
SAMD5	0.00208471	
C19orf44	0.00208471	
ST6GALNAC6	0.00208471	
DNALI1	0.00208471	
TMEM231	0.00208471	
B3GALT1	0.00208471	
HOXC5	0.00208471	
MXRA7	0.00208471	
METTL21A	0.00208471	
USP40	0.00208471	
C7orf73	0.00208471	
CPXM2	0.00208471	
FGF12	0.00208471	
RAB30-AS1	0.00208471	
SAMD3	0.00208471	
CPNE8	0.00208471	
PAPPA2	0.00208471	
CTD-3128G10.7	0.00208471	

BOLL	0.00208471	
TRABD	0.00208471	
PPT2-EGFL8	0.00208471	
GHRL	0.00208471	
KRTAP12-2	0.00208471	
BLCAP	0.00208471	
UPK2	0.00208471	
DAPK2	0.00208471	
IPP	0.00208471	
FYTTD1	0.00208471	
CKMT1B	0.00208471	
MIR1915	0.00208471	
KIAA1328	0.00208471	
NUDT4	0.00208471	
HMGXB3	0.00208471	
DNAJC6	0.00208471	
ANAPC13	0.00208471	
HOTTIP	0.00208471	
HES5	0.00208471	
NAT8L	0.00208471	
FAM72B	0.00208471	
MIR4500HG	0.00208471	
EID2B	0.00208471	
CCDC71L	0.00208471	
TP53I13	0.00208471	
KDM7A	0.00208471	
RXFP1	0.00208471	
TYW1	0.00208471	
RCE1	0.00208471	
TFF3	0.00208471	
RP11-574K11.24	0.00208471	
AC125232.1	0.00208471	
DUSP8	0.00208471	
XXbac-B476C20.9	0.00208471	
TCTA	0.00208471	
AC016708.2	0.00208471	
CHRNA4	0.00208471	
TM4SF18	0.00208471	
CAPN15	0.00208471	
GRIA2	0.00208471	
SERPINB5	0.00208471	

SLCO4C1	0.00208471	
SAPCD1	0.00208471	
RPARP-AS1	0.00208471	
ADAMTS5	0.00208471	
SIX4	0.00208471	
43531	0.00208471	
BEX5	0.00208471	
RABL3	0.00208471	
PLEKHH3	0.00208471	
NEK11	0.00208471	
NNAT	0.00208471	
C5orf51	0.00208471	
SCNN1D	0.00208471	
SLC8A3	0.00208471	
EMR1	0.00208471	
RABL2B	0.00208471	
NOL6	0.00208471	
FAM227B	0.00208471	
COX11	0.00208471	
ZNF213-AS1	0.00208471	
LYVE1	0.00208471	
SNHG11	0.00208471	
DUSP26	0.00208471	
DGKB	0.00208471	
MTNR1A	0.00208471	
MSI2	0.00208471	
SARM1	0.00208471	
NXPH4	0.00208471	
SPRY3	0.00208471	
TMEM161A	0.00208471	
DRD2	0.00208471	
LRRC75B	0.00208471	
NBPF3	0.00208471	
LRIG1	0.00208471	
YBEY	0.00208471	
RP1-151F17.2	0.00208471	
CTSH	0.00208471	
BFSP1	0.00208471	
CXorf23	0.00208471	
LAG3	0.00208471	
FOLR2	0.00208471	

RPL22L1	0.00208471	
OCM	0.00208471	
WDR1	0.00208471	
EHD4	0.00208471	
TGDS	0.00208471	
PCF11	0.00208471	
AGBL5	0.00208471	
CRIP1	0.00208471	
GPR98	0.00208471	
SESN3	0.00208471	
AAGAB	0.00208471	
CDCA7	0.00208471	
LINC00511	0.00208471	
RP11-295P9.3	0.00208471	
SLC38A2	0.00208471	
ZNF554	0.00208471	
STEAP4	0.00208471	
TCTN3	0.00208471	
IER2	0.00208471	
CHAT	0.00208471	
IGHV1-2	0.00208471	
PHF21B	0.00208471	
DNASE1L3	0.00208471	
TLL1	0.00208471	
SLC15A1	0.00208471	
RP11-418J17.1	0.00208471	
CCDC86	0.00208471	
TMEM52	0.00208471	
TMEM59	0.002033864	
FLJ44511	0.002033864	
DCUN1D2	0.002033864	
KCNA2	0.002033864	
MAGEA2B	0.002033864	
AIM1L	0.002033864	
RPS7P1	0.002033864	
SPSB3	0.002033864	
PLA2G12A	0.002033864	
SLC37A1	0.002033864	
ADORA2B	0.002033864	
ABCA5	0.002033864	
CNN2	0.002033864	

GRPR	0.002033864	
PRR19	0.002033864	
TMEM200B	0.002033864	
A2M-AS1	0.002033864	
DNAH14	0.002033864	
FAM149B1	0.002033864	
OGFR	0.002033864	
MESP1	0.002033864	
MEX3D	0.002033864	
DERL3	0.002033864	
RTP4	0.002033864	
TMEM138	0.002033864	
HIST1H1D	0.002033864	
PNMA3	0.002033864	
WWC3	0.002033864	
S100A3	0.002033864	
HORMAD2-AS1	0.002033864	
ZBTB7C	0.002033864	
PRCD	0.002033864	
CORT	0.002033864	
FAM3B	0.002033864	
TSPAN8	0.002033864	
CCL17	0.002033864	
RIOK3	0.002033864	
NSUN4	0.002033864	
CDC42EP4	0.002033864	
ADAM8	0.002033864	
ZNF512	0.002033864	
COQ2	0.002033864	
SRSF8	0.002033864	
HSPA13	0.002033864	
CYB5R2	0.002033864	
FGD1	0.002033864	
SGMS1-AS1	0.002033864	
ZNF343	0.002033864	
NAA20	0.002033864	
IGHV3-30	0.002033864	
CHRNA1	0.002033864	
CC2D1A	0.002033864	
KRT85	0.002033864	
SLC9A3	0.002033864	

KCNA5	0.002033864	
GPR25	0.002033864	
KCNH4	0.002033864	
SEC22C	0.002033864	
KCNH1	0.002033864	
ZNF283	0.002033864	
ZNF568	0.002033864	
PPME1	0.002033864	
SGSH	0.002033864	
FAM120A	0.002033864	
SNED1	0.002033864	
LARP7	0.002033864	
POFUT2	0.002033864	
TMA16	0.002033864	
ANKRD10	0.002033864	
UBE3D	0.002033864	
MESDC1	0.002033864	
HGSNAT	0.002033864	
SHOX	0.002033864	
RP11-317J10.2	0.002033864	
MLPH	0.002033864	
FICD	0.002033864	
ZNF562	0.002033864	
ANKS1B	0.002033864	
FNDC3B	0.002033864	
FAM92A1	0.002033864	
ASB15	0.002033864	
CSN2	0.002033864	
GCNT4	0.002033864	
FAM127B	0.002033864	
GRID2	0.002033864	
JPH4	0.002033864	
ERAP1	0.002033864	
CSRP2	0.002033864	
ZC3H3	0.002033864	
TULP2	0.002033864	
MTHFSD	0.002033864	
TCTN2	0.002033864	
CWF19L1	0.002033864	
STYX	0.002033864	
STAP1	0.002033864	

PTGDR2	0.002033864	
RP11-108M9.4	0.002033864	
SEMA4B	0.002033864	
UNC5A	0.002033864	
CRYBA2	0.002033864	
CAAP1	0.002033864	
DOCK11	0.002033864	
SNX16	0.002033864	
TTC23L	0.002033864	
CHSY3	0.002033864	
IGKV1-6	0.002033864	
ITFG2	0.002033864	
SDCCAG8	0.002033864	
PEAR1	0.002033864	
C17orf49	0.002033864	
TNFRSF13C	0.002033864	
ZBTB46	0.002033864	
ARHGAP19	0.002033864	
TSSK4	0.002033864	
LINC00886	0.002033864	
KCNK3	0.002033864	
AFAP1L1	0.002033864	
DIP2C	0.002033864	
LARS2	0.002033864	
MYOZ3	0.002033864	
ZNF432	0.002033864	
TRIM52	0.002033864	
LMBRD1	0.002033864	
ZNF480	0.002033864	
OSBP2	0.002033864	
ENTPD7	0.002033864	
TPK1	0.002033864	
MAG	0.002033864	
RP11-33O4.1	0.002033864	
DNAJA4	0.002033864	
LGR6	0.002033864	
RP11-760H22.2	0.002033864	
SERHL2	0.002033864	
ANKRD50	0.002033864	
CAGE1	0.002033864	
P3H1	0.002033864	

PPAP2B	0.002033864	
PITRM1	0.002033864	
HIST2H3A	0.002033864	
POLR2J4	0.002033864	
MIOS	0.002033864	
HTR1B	0.002033864	
OXNAD1	0.002033864	
SYNM	0.002033864	
TREH	0.002033864	
MUC2	0.002033864	
PTCHD2	0.002033864	
CD27-AS1	0.002033864	
SLC9A8	0.002033864	
LHX4-AS1	0.002033864	
ZCCHC4	0.002033864	
HOXC10	0.001983017	
THUMPD3	0.001983017	
RP11-347C12.10	0.001983017	
AQP4	0.001983017	
SUMF1	0.001983017	
B4GALT4	0.001983017	
KCNMB2	0.001983017	
RP11-4O1.2	0.001983017	
ZNF136	0.001983017	
RNF5P1	0.001983017	
IGHV3-53	0.001983017	
AKIP1	0.001983017	
RIT2	0.001983017	
ZFYVE27	0.001983017	
ZNF100	0.001983017	
SCPEP1	0.001983017	
METTL14	0.001983017	
SNHG5	0.001983017	
ZNF341	0.001983017	
STARD4	0.001983017	
AGK	0.001983017	
KRT8P12	0.001983017	
NHEJ1	0.001983017	
TRIM38	0.001983017	
C9orf9	0.001983017	
MYPN	0.001983017	

GPN2	0.001983017	
LINC00910	0.001983017	
FLG2	0.001983017	
ZNF611	0.001983017	
MANBA	0.001983017	
TMEM263	0.001983017	
RP11-303E16.2	0.001983017	
TYW3	0.001983017	
PDGFC	0.001983017	
LCLAT1	0.001983017	
RIMBP3	0.001983017	
CD209	0.001983017	
MDGA2	0.001983017	
SEMA3A	0.001983017	
SPCS3	0.001983017	
DNASE1L2	0.001983017	
SULF2	0.001983017	
CCR8	0.001983017	
RP11-403I13.5	0.001983017	
KANSL3	0.001983017	
ADIPOQ	0.001983017	
JHDM1D-AS1	0.001983017	
C15orf41	0.001983017	
CTPS1	0.001983017	
HDHD1	0.001983017	
CHRM2	0.001983017	
ATP9A	0.001983017	
ABCA3	0.001983017	
TMEM214	0.001983017	
C1orf61	0.001983017	
U4	0.001983017	
TNNC2	0.001983017	
CYGB	0.001983017	
TRIOBP	0.001983017	
PDP2	0.001983017	
MRAP2	0.001983017	
RBMY1A1	0.001983017	
MYH3	0.001983017	
PPP4R1	0.001983017	
OCA2	0.001983017	
SETD3	0.001983017	

SLC25A45	0.001983017	
MR1	0.001983017	
RP11-440D17.3	0.001983017	
ZDHHC11B	0.001983017	
SLC16A5	0.001983017	
PPP1R21	0.001983017	
ZNF826P	0.001983017	
GH2	0.001983017	
PPP1R16B	0.001983017	
KIN	0.001983017	
SLC2A9	0.001983017	
IP6K2	0.001983017	
ANXA2P2	0.001983017	
CMTR1	0.001983017	
HSBP1L1	0.001983017	
INSC	0.001983017	
C8orf44-SGK3	0.001983017	
TNFRSF17	0.001983017	
FAM86C2P	0.001983017	
ERO1LB	0.001983017	
TMED4	0.001983017	
MSTO2P	0.001983017	
WARS2	0.001983017	
DUSP7	0.001983017	
IAH1	0.001983017	
FCGBP	0.001983017	
KCNRG	0.001983017	
MIR568	0.001983017	
WDR5B	0.001983017	
SMIM3	0.001983017	
TMC7	0.001983017	
SYT3	0.001983017	
RP11-1259L22.2	0.001983017	
CPLX2	0.001983017	
PDLIM3	0.001983017	
NAAA	0.001983017	
NOX1	0.001983017	
TENM2	0.001983017	
TRIM46	0.001983017	
KCNJ5	0.001983017	
BBIP1	0.001983017	

LDLRAD4	0.001983017	
CYB5R4	0.001983017	
GNLY	0.001983017	
ANKEF1	0.001983017	
ADRB3	0.001983017	
MICU3	0.001983017	
GUCA2B	0.001983017	
RP11-156E6.1	0.001983017	
DGCR5	0.001983017	
NBPF8P	0.001983017	
RCN1	0.001983017	
RELL2	0.001983017	
PCDHB15	0.001983017	
DCAF4	0.001983017	
RPL4P5	0.001983017	
ZNF678	0.001983017	
WSB2	0.001983017	
DCUN1D4	0.001983017	
GGACT	0.001983017	
SCN9A	0.001983017	
VPREB3	0.001983017	
SCARB2	0.001983017	
MTMR14	0.001983017	
NHLRC3	0.001983017	
ANXA2R	0.001983017	
TNFRSF18	0.001983017	
CCDC50	0.001983017	
TRHR	0.001983017	
GPR89B	0.001983017	
ZNF124	0.001983017	
LIPH	0.001983017	
ZNF330	0.001983017	
ANO6	0.001983017	
SLC9A6	0.001983017	
FAM83A-AS1	0.001983017	
PKN3	0.001983017	
EPHA5	0.001983017	
ZNF613	0.001983017	
CETN1	0.001983017	
TSPAN33	0.001983017	
RP11-567M16.6	0.001983017	

TRPC3	0.001983017	
EVI5L	0.001983017	
CCDC40	0.001983017	
ELL3	0.001983017	
RP11-49I11.1	0.001983017	
C7orf60	0.001983017	
DOK5	0.001983017	
FAM171A2	0.001983017	
CTC-429P9.3	0.001983017	
PRSS3	0.001983017	
ZBTB42	0.001983017	
TC2N	0.001932171	
FIBCD1	0.001932171	
ZNF320	0.001932171	
CRNDE	0.001932171	
PHF13	0.001932171	
LEFTY2	0.001932171	
MYBPC3	0.001932171	
ATOH8	0.001932171	
MYCNOS	0.001932171	
FOXN4	0.001932171	
TIAM2	0.001932171	
PAIP2B	0.001932171	
RBM33	0.001932171	
FANCB	0.001932171	
UNCX	0.001932171	
FAM35A	0.001932171	
PDXDC2P	0.001932171	
KCNIP4	0.001932171	
KLHDC7A	0.001932171	
SIGLEC5	0.001932171	
FAM104B	0.001932171	
TOR4A	0.001932171	
URGCP	0.001932171	
WNT5B	0.001932171	
AC010894.3	0.001932171	
MCTP2	0.001932171	
GRIN2C	0.001932171	
DNAH6	0.001932171	
RP11-160O5.1	0.001932171	
NUBP1	0.001932171	

SLFN11	0.001932171	
TMEM60	0.001932171	
B3GNT4	0.001932171	
LETMD1	0.001932171	
GNPTAB	0.001932171	
MAGIX	0.001932171	
DYSF	0.001932171	
C17orf100	0.001932171	
NRTN	0.001932171	
METTL4	0.001932171	
ACTRT3	0.001932171	
ZNF35	0.001932171	
ANAPC16	0.001932171	
ZNF605	0.001932171	
PRMT3	0.001932171	
TRMU	0.001932171	
AICDA	0.001932171	
DRD4	0.001932171	
TMEM74B	0.001932171	
ZNF33A	0.001932171	
FKBP7	0.001932171	
TNFAIP6	0.001932171	
INAFM2	0.001932171	
FADS6	0.001932171	
CRYBB3	0.001932171	
PRSS22	0.001932171	
COPZ2	0.001932171	
RPS28P7	0.001932171	
C2CD2L	0.001932171	
CCDC78	0.001932171	
KIAA1715	0.001932171	
FDXACB1	0.001932171	
DTX4	0.001932171	
PLGLA	0.001932171	
NLGN4X	0.001932171	
DNAH1	0.001932171	
RP11-93B14.9	0.001932171	
EML6	0.001932171	
PRDM14	0.001932171	
RP11-159J3.1	0.001932171	
TTC13	0.001932171	

SLC22A17	0.001932171	
FAM47E-STBD1	0.001932171	
AC016747.3	0.001932171	
KIAA0513	0.001932171	
ORMDL1	0.001932171	
BTC	0.001932171	
WIPF3	0.001932171	
B3GALT4	0.001932171	
FAM89A	0.001932171	
TMEM116	0.001932171	
MRM1	0.001932171	
FTSJ2	0.001932171	
STMN4	0.001932171	
MOSPD2	0.001932171	
BAI1	0.001932171	
PHKG1	0.001932171	
SULT1C2	0.001932171	
TCAF2	0.001932171	
CASC3	0.001932171	
CAPS2	0.001932171	
CPM	0.001932171	
RASL11A	0.001932171	
GRPEL2	0.001932171	
GBP3	0.001932171	
CCDC138	0.001932171	
MTAP	0.001932171	
CPEB1	0.001932171	
FAM72D	0.001932171	
TLR5	0.001932171	
ARHGEF9	0.001932171	
ACP6	0.001932171	
HECA	0.001932171	
CTB-171A8.1	0.001932171	
RPL32P3	0.001932171	
ODF2	0.001932171	
POU4F3	0.001932171	
FBN2	0.001932171	
TMEM41B	0.001932171	
SLC9A7	0.001932171	
FAM212A	0.001932171	
RAB21	0.001932171	

IQCE	0.001932171	
GALC	0.001932171	
KCNJ2-AS1	0.001932171	
ALOX12-AS1	0.001932171	
MALAT1	0.001932171	
RBM5	0.001932171	
MIR3936	0.001932171	
NARS	0.001932171	
SRBD1	0.001932171	
VGF	0.001932171	
DBH-AS1	0.001932171	
NOX4	0.001932171	
PDE8B	0.001932171	
ZNF416	0.001932171	
CTD-2636A23.2	0.001932171	
FCF1P2	0.001932171	
UGT2B28	0.001932171	
ZNF195	0.001932171	
SLC24A2	0.001932171	
KCNMB1	0.001932171	
HARS2	0.001932171	
ITPRIPL2	0.001932171	
SLC27A3	0.001932171	
OSBPL1A	0.001932171	
RTBDN	0.001932171	
AQP3	0.001932171	
DCT	0.001932171	
CCL7	0.001932171	
FAM200B	0.001932171	
SLC25A47	0.001932171	
TCAF1	0.001932171	
COX15	0.001932171	
CTC-534A2.2	0.001932171	
ACE2	0.001932171	
FAM99B	0.001932171	
NT5DC3	0.001932171	
NUDT15	0.001932171	
KRTAP5-6	0.001932171	
TNFSF18	0.001932171	
ICOSLG	0.001932171	
CARD14	0.001932171	

ZNF669	0.001932171	
KCNJ10	0.001932171	
PHF11	0.001932171	
SCAND2P	0.001932171	
FBN3	0.001932171	
MIR4530	0.001932171	
ZSCAN20	0.001932171	
SLC45A4	0.001932171	
PRDM8	0.001932171	
ZBP1	0.001932171	
SEMA3G	0.001932171	
ZC3HAV1	0.001932171	
ZNF280D	0.001932171	
C11orf87	0.001932171	
DHX32	0.001932171	
TATDN2	0.001932171	
TRIM6	0.001932171	
CLCN4	0.001932171	
HRNR	0.001932171	
DPF1	0.001932171	
AMHR2	0.001932171	
RP11-135F9.3	0.001932171	
SLC10A5	0.001932171	
YARS2	0.001932171	
PAQR5	0.001932171	
ATRN	0.001932171	
SCGB3A1	0.001932171	
FBXO3	0.001932171	
ISM1	0.001932171	
BTBD19	0.001932171	
CYP39A1	0.001932171	
AP001065.15	0.001932171	
FAM213B	0.001932171	
C1orf53	0.001932171	
ARV1	0.001932171	
ARPP19	0.001932171	
PHLDB2	0.001932171	
C7orf55-LUC7L2	0.001881324	
NBPF22P	0.001881324	
LRRC28	0.001881324	
EPB42	0.001881324	

TEKT5	0.001881324	
CACNA1D	0.001881324	
JAKMIP1	0.001881324	
WDR31	0.001881324	
CTD-2228K2.7	0.001881324	
ZNF528	0.001881324	
IFNLR1	0.001881324	
STXBP6	0.001881324	
TEX261	0.001881324	
GBA3	0.001881324	
DAZ4	0.001881324	
AGO2	0.001881324	
CRYBA1	0.001881324	
CYS1	0.001881324	
SCUBE2	0.001881324	
LIPT1	0.001881324	
PYGO1	0.001881324	
SNORD104	0.001881324	
ACVR1C	0.001881324	
RPRM	0.001881324	
NCKAP5L	0.001881324	
ZNF517	0.001881324	
GNG8	0.001881324	
ABCF2	0.001881324	
TPO	0.001881324	
ZNF276	0.001881324	
EVC	0.001881324	
MPPE1	0.001881324	
STK26	0.001881324	
SPRR1B	0.001881324	
KIAA1324	0.001881324	
MEF2B	0.001881324	
EPX	0.001881324	
METAP1D	0.001881324	
BFSP2	0.001881324	
HAPLN1	0.001881324	
ALG9	0.001881324	
METTL8	0.001881324	
ZNF23	0.001881324	
ZNF33B	0.001881324	
IGFN1	0.001881324	

UBA6-AS1	0.001881324	
MLLT11	0.001881324	
SEC61A2	0.001881324	
GPR157	0.001881324	
CRIPAK	0.001881324	
SSPO	0.001881324	
SRGAP2B	0.001881324	
CCDC134	0.001881324	
PCDHGA9	0.001881324	
MAST3	0.001881324	
HEPACAM	0.001881324	
MFHAS1	0.001881324	
NAP1L5	0.001881324	
AHNAK2	0.001881324	
CA11	0.001881324	
MCTP1	0.001881324	
MYH7B	0.001881324	
CRACR2B	0.001881324	
ZNF526	0.001881324	
KDSR	0.001881324	
NUP88	0.001881324	
AIFM3	0.001881324	
SLC25A23	0.001881324	
PLA2G5	0.001881324	
SLC12A4	0.001881324	
GSG2	0.001881324	
TLK2	0.001881324	
BHLHE22	0.001881324	
KIF13A	0.001881324	
STC1	0.001881324	
SLC12A8	0.001881324	
SCN4B	0.001881324	
C9orf172	0.001881324	
FLRT1	0.001881324	
TEKT4P2	0.001881324	
ARSD	0.001881324	
RP11-159D12.8	0.001881324	
PRRT1	0.001881324	
MMD	0.001881324	
OBSCN	0.001881324	
OLAH	0.001881324	

C1QL1	0.001881324	
LYRM7	0.001881324	
LETM2	0.001881324	
PRR18	0.001881324	
RP11-115C10.1	0.001881324	
RFESD	0.001881324	
RIBC1	0.001881324	
GIMAP2	0.001881324	
FAM65C	0.001881324	
RP13-270P17.2	0.001881324	
WDR43	0.001881324	
MPHOSPH10	0.001881324	
ROPN1L	0.001881324	
CFAP20	0.001881324	
HERPUD2	0.001881324	
PI4KAP2	0.001881324	
SETD6	0.001881324	
RIBC2	0.001881324	
TSC22D1-AS1	0.001881324	
USP51	0.001881324	
ST20	0.001881324	
ARHGAP5-AS1	0.001881324	
C2orf68	0.001881324	
MIR3143	0.001881324	
NAPG	0.001881324	
COCH	0.001881324	
NUDT13	0.001881324	
GOLGA2P5	0.001881324	
ZNF222	0.001881324	
ABCD2	0.001881324	
FZD2	0.001881324	
TTC7B	0.001881324	
UBLCP1	0.001881324	
INA	0.001881324	
HMCN1	0.001881324	
COX10-AS1	0.001881324	
NTNG2	0.001881324	
C7orf31	0.001881324	
PZP	0.001881324	
C19orf73	0.001881324	
SLC5A3	0.001881324	

IGHV3-49	0.001881324	
CTC-444N24.11	0.001881324	
TSPAN10	0.001881324	
IQCG	0.001881324	
C8orf46	0.001881324	
IGHV1-69-2	0.001881324	
LILRB3	0.001881324	
ZNF577	0.001881324	
HPCAL4	0.001881324	
RCBTB2	0.001881324	
TPH2	0.001881324	
PTPRT	0.001881324	
CH507-42P11.8	0.001881324	
SLC41A1	0.001881324	
TMEM63B	0.001881324	
LINC01451	0.001881324	
XRRA1	0.001881324	
TMEM98	0.001881324	
RNF19B	0.001881324	
FAM84A	0.001881324	
HBQ1	0.001881324	
ZSCAN2	0.001881324	
VMAC	0.001881324	
MMP10	0.001881324	
FMO1	0.001881324	
MORN1	0.001881324	
DOC2B	0.001881324	
WDR70	0.001881324	
RP4-763G1.2	0.001881324	
HMCN2	0.001881324	
RP11-173M11.2	0.001881324	
CRIP3	0.001881324	
ZMAT1	0.001881324	
ENTPD8	0.001881324	
LYSMD2	0.001881324	
ADAMTSL2	0.001881324	
IGFALS	0.001881324	
TMEM25	0.001881324	
ARFIP1	0.001881324	
ZBTB49	0.001881324	
FOXN3-AS1	0.001881324	

ATP1A2	0.001881324	
SLC37A2	0.001881324	
FAM222A	0.001830477	
KCNK15	0.001830477	
RASGEF1A	0.001830477	
EPHA7	0.001830477	
ZNF347	0.001830477	
PLCXD3	0.001830477	
GLIPR1L2	0.001830477	
MLC1	0.001830477	
FAT2	0.001830477	
TSPAN13	0.001830477	
FOLR1	0.001830477	
INPP5F	0.001830477	
PKNOX2	0.001830477	
SH3TC1	0.001830477	
SMYD1	0.001830477	
LYPD3	0.001830477	
FOXE3	0.001830477	
SLC16A13	0.001830477	
WLS	0.001830477	
MTND1P23	0.001830477	
MIR4297	0.001830477	
MIRLET7BHG	0.001830477	
PAQR3	0.001830477	
DOC2GP	0.001830477	
ASIC4	0.001830477	
DYNC1LI1	0.001830477	
TANGO6	0.001830477	
SCT	0.001830477	
GDA	0.001830477	
ZNF626	0.001830477	
ZSCAN30	0.001830477	
IL17RA	0.001830477	
MIR5187	0.001830477	
COX7B2	0.001830477	
AC004381.6	0.001830477	
PXYLP1	0.001830477	
ZRANB3	0.001830477	
PFN1P11	0.001830477	
GPR68	0.001830477	

KLHL32	0.001830477	
ZC2HC1A	0.001830477	
GAS6-AS1	0.001830477	
MREG	0.001830477	
RP11-521M14.1	0.001830477	
DPP9	0.001830477	
SDK2	0.001830477	
NUDT19	0.001830477	
MPO	0.001830477	
LINC00857	0.001830477	
KCTD5	0.001830477	
SPIRE2	0.001830477	
RP11-15H20.6	0.001830477	
ENTPD3	0.001830477	
IQCD	0.001830477	
TMEM126B	0.001830477	
ZNF182	0.001830477	
ACOT1	0.001830477	
UPK1A	0.001830477	
GPR146	0.001830477	
ACN9	0.001830477	
B4GAT1	0.001830477	
AC113189.5	0.001830477	
RP11-26J3.3	0.001830477	
TNFRSF11B	0.001830477	
DNAJC15	0.001830477	
ATP8A1	0.001830477	
JPH1	0.001830477	
TACSTD2	0.001830477	
MTRF1L	0.001830477	
TMEM80	0.001830477	
RP5-894A10.2	0.001830477	
MIPEP	0.001830477	
POGLUT1	0.001830477	
DHFRP1	0.001830477	
CPO	0.001830477	
DDX10	0.001830477	
AKAP7	0.001830477	
WASH6P	0.001830477	
ZNF425	0.001830477	
ZNF460	0.001830477	

TTLL7	0.001830477	
CYP2R1	0.001830477	
TMEM200A	0.001830477	
GAL	0.001830477	
C12orf43	0.001830477	
FKBP14	0.001830477	
ABCC11	0.001830477	
C22orf39	0.001830477	
CACNA1F	0.001830477	
BEX4	0.001830477	
MIOX	0.001830477	
FAM83A	0.001830477	
ALPP	0.001830477	
SLC25A27	0.001830477	
RP11-345J4.5	0.001830477	
SCLT1	0.001830477	
BBOX1	0.001830477	
WDFY3-AS2	0.001830477	
FBXO25	0.001830477	
EBLN2	0.001830477	
C3orf67	0.001830477	
DUSP14	0.001830477	
IL17B	0.001830477	
APOL2	0.001830477	
KIAA1467	0.001830477	
FAM174A	0.001830477	
CACNA1G	0.001830477	
HLA-K	0.001830477	
WNT9A	0.001830477	
CA4	0.001830477	
PALD1	0.001830477	
ASTE1	0.001830477	
C2orf69	0.001830477	
TMEM136	0.001830477	
COLCA1	0.001830477	
SLC1A7	0.001830477	
MTG2	0.001830477	
ESPNL	0.001830477	
CD300LG	0.001830477	
TMEM67	0.001830477	
CHRNB1	0.001830477	

MANEAL	0.001830477	
TMCO4	0.001830477	
GPR143	0.001830477	
RPRD1B	0.001830477	
CLCNKA	0.001830477	
SULT1C4	0.001830477	
RAPSN	0.001830477	
FBXL8	0.001830477	
TBX19	0.001830477	
IGLV3-21	0.001830477	
PPP1R3G	0.001830477	
P4HTM	0.001830477	
SYN3	0.001830477	
TRMT10B	0.001830477	
FBXO24	0.001830477	
HMGCLL1	0.001830477	
ZNF430	0.001830477	
43534	0.001830477	
HLA-L	0.001830477	
KANSL2	0.001830477	
BTN2A2	0.001830477	
CCDC174	0.001830477	
ZNF841	0.001830477	
DRD3	0.001830477	
SLC26A2	0.001830477	
UBXN2A	0.001830477	
COLQ	0.001830477	
HELB	0.001830477	
FEZ2	0.001830477	
PHKA2	0.001830477	
RALGPS1	0.001830477	
POLR3B	0.001830477	
TCEAL8	0.001830477	
SHPK	0.001830477	
AGBL2	0.001830477	
RNASE2	0.001830477	
COLEC11	0.001830477	
CCDC69	0.001830477	
RHBDL2	0.001830477	
UBASH3A	0.001830477	
SLC16A10	0.001830477	

ZNF649	0.001830477	
GNG13	0.001830477	
DUSP11	0.001830477	
PRKG2	0.001830477	
MSLN	0.001830477	
RP1-30M3.5	0.001830477	
TMEM102	0.001830477	
DUXAP8	0.001830477	
GARNL3	0.001830477	
ZNF22	0.001779631	
OVGP1	0.001779631	
ZNF506	0.001779631	
ZSCAN22	0.001779631	
CNPY4	0.001779631	
FAM45A	0.001779631	
NAALADL1	0.001779631	
TRABD2B	0.001779631	
REM1	0.001779631	
P2RX1	0.001779631	
ALDH1L1-AS2	0.001779631	
FAM99A	0.001779631	
ABCA8	0.001779631	
CSF3	0.001779631	
MIR4258	0.001779631	
STAG3	0.001779631	
AKIRIN1	0.001779631	
EBF2	0.001779631	
PRDM11	0.001779631	
H1FX-AS1	0.001779631	
C2orf50	0.001779631	
MAGI2-AS3	0.001779631	
RP11-517I3.2	0.001779631	
SALL4	0.001779631	
SLC18A2	0.001779631	
CDC20B	0.001779631	
SLC39A8	0.001779631	
PRAME	0.001779631	
C8orf44	0.001779631	
CYP7B1	0.001779631	
FBXO16	0.001779631	
DEF8	0.001779631	

GRM8	0.001779631	
RP11-420L9.5	0.001779631	
CCDC151	0.001779631	
MIR4740	0.001779631	
NHLH2	0.001779631	
CDKN2AIP	0.001779631	
NRG2	0.001779631	
RHOF	0.001779631	
DACT2	0.001779631	
RNF157	0.001779631	
LINC01232	0.001779631	
SIRT4	0.001779631	
CCDC125	0.001779631	
TSSK6	0.001779631	
SLC3A1	0.001779631	
ZNF549	0.001779631	
LPAL2	0.001779631	
PAK6	0.001779631	
PNPLA7	0.001779631	
CRHR1	0.001779631	
CYB5RL	0.001779631	
DENND1A	0.001779631	
WHAMM	0.001779631	
CDYL2	0.001779631	
SNAP91	0.001779631	
SST	0.001779631	
MAGEA8	0.001779631	
ICMT	0.001779631	
TMEM260	0.001779631	
DYRK4	0.001779631	
KLHDC9	0.001779631	
HNRNPLL	0.001779631	
CHPF2	0.001779631	
TUBGCP5	0.001779631	
LTK	0.001779631	
LINC00525	0.001779631	
ATP1A1-AS1	0.001779631	
ATP6V1B1	0.001779631	
FBXO48	0.001779631	
ZNF17	0.001779631	
DHX29	0.001779631	

NCAM2	0.001779631	
BSDC1	0.001779631	
CCDC122	0.001779631	
OBFC1	0.001779631	
ARHGAP18	0.001779631	
RP11-258F1.2	0.001779631	
PXMP4	0.001779631	
RP11-47A8.5	0.001779631	
RPL36A-HNRNPH2	0.001779631	
PSPC1	0.001779631	
KIAA1919	0.001779631	
RAPGEFL1	0.001779631	
SYT2	0.001779631	
ARPC4-TTLL3	0.001779631	
ESRP1	0.001779631	
SCG2	0.001779631	
RPS4Y2	0.001779631	
THUMPD1	0.001779631	
TMEM64	0.001779631	
ASMT	0.001779631	
MIR210HG	0.001779631	
FAM189A2	0.001779631	
CDK20	0.001779631	
TXK	0.001779631	
ULK4	0.001779631	
FAM86HP	0.001779631	
ZDHHC8	0.001779631	
TLR9	0.001779631	
LRRC61	0.001779631	
C9orf43	0.001779631	
SLC38A10	0.001779631	
WDR91	0.001779631	
FAM120B	0.001779631	
CYP26A1	0.001779631	
FAM86FP	0.001779631	
TSHZ2	0.001779631	
KCNQ4	0.001779631	
HIST1H2AL	0.001779631	
ZNF138	0.001779631	
BOK-AS1	0.001779631	
NBPF14	0.001779631	

ADAMTS9	0.001779631	
COL9A2	0.001779631	
LINC00888	0.001779631	
TLCD2	0.001779631	
FCAR	0.001779631	
C12orf5	0.001779631	
HRCT1	0.001779631	
DUOX2	0.001779631	
RNF121	0.001779631	
GPR89A	0.001779631	
MIR212	0.001779631	
DDX60L	0.001779631	
JPH3	0.001779631	
FARP1	0.001779631	
CA5BP1	0.001779631	
PLGRKT	0.001779631	
PLXNA3	0.001779631	
ZNF711	0.001779631	
SERP2	0.001779631	
WISP2	0.001779631	
WWC2-AS2	0.001779631	
FAM102A	0.001779631	
MICU1	0.001779631	
SNX11	0.001779631	
MAMSTR	0.001779631	
C15orf57	0.001779631	
NXPH3	0.001779631	
GDF7	0.001779631	
RNU6ATAC	0.001779631	
USP44	0.001779631	
C10orf62	0.001779631	
SOX15	0.001779631	
DDIT4L	0.001779631	
DPY19L3	0.001779631	
FAM21C	0.001779631	
VENTX	0.001779631	
CERS6	0.001779631	
TTC14	0.001779631	
NATD1	0.001779631	
AATK	0.001779631	
NRBP2	0.001779631	

BRSK2	0.001779631	
AC004862.6	0.001779631	
DEGS2	0.001779631	
AC004967.7	0.001779631	
CCT6B	0.001779631	
IGLV4-69	0.001728784	
ZNF346	0.001728784	
SLC35E3	0.001728784	
NEIL3	0.001728784	
TMEM30B	0.001728784	
SMYD4	0.001728784	
PIP5KL1	0.001728784	
DAOA	0.001728784	
TAF1A-AS1	0.001728784	
KRTAP10-4	0.001728784	
ANO7	0.001728784	
RP11-35G9.3	0.001728784	
KCNA1	0.001728784	
TMEM181	0.001728784	
TWSG1	0.001728784	
ERCC6L	0.001728784	
NEUROD6	0.001728784	
HSPB6	0.001728784	
CCL24	0.001728784	
PTGER1	0.001728784	
C14orf28	0.001728784	
IGSF9	0.001728784	
SMIM2-AS1	0.001728784	
UCP3	0.001728784	
CYBRD1	0.001728784	
PTPDC1	0.001728784	
WNT16	0.001728784	
TMEM86B	0.001728784	
LSM8	0.001728784	
PSMA3-AS1	0.001728784	
DLC1	0.001728784	
BORA	0.001728784	
SPG20	0.001728784	
GIMAP5	0.001728784	
SPDYE3	0.001728784	
FAM210A	0.001728784	

DICER1-AS1	0.001728784	
ZNF546	0.001728784	
ZNF529	0.001728784	
SNX25	0.001728784	
FBXO46	0.001728784	
PGBD4	0.001728784	
RP11-327E2.5	0.001728784	
RWDD4	0.001728784	
HOTAIR	0.001728784	
TRPM4	0.001728784	
TNK1	0.001728784	
ALOX15B	0.001728784	
NAALADL2	0.001728784	
ZNF271P	0.001728784	
DTD1	0.001728784	
TMEM133	0.001728784	
SLC31A2	0.001728784	
VNN2	0.001728784	
MAN2B2	0.001728784	
IGHV3-33	0.001728784	
FAHD2A	0.001728784	
TEKT1	0.001728784	
NPHP4	0.001728784	
CNTLN	0.001728784	
AHI1	0.001728784	
TMED6	0.001728784	
FAM206A	0.001728784	
SNHG3	0.001728784	
ZGRF1	0.001728784	
CCNJL	0.001728784	
TRAF3IP2-AS1	0.001728784	
UPP2	0.001728784	
APCDD1	0.001728784	
LINC00998	0.001728784	
C5AR2	0.001728784	
POMGNT2	0.001728784	
UCN	0.001728784	
YPEL5	0.001728784	
FXYD7	0.001728784	
APH1B	0.001728784	
PIK3IP1	0.001728784	

LAYN	0.001728784	
HIST1H2BI	0.001728784	
KCND1	0.001728784	
AGAP1	0.001728784	
PRELID2	0.001728784	
PHACTR3	0.001728784	
CLCN1	0.001728784	
ACYP1	0.001728784	
FOCAD	0.001728784	
XYLT2	0.001728784	
GAREML	0.001728784	
OMP	0.001728784	
RUFY4	0.001728784	
SFTPA1	0.001728784	
DNAJC21	0.001728784	
NALCN	0.001728784	
ZNF571	0.001728784	
RAB39A	0.001728784	
EMC6	0.001728784	
TUSC1	0.001728784	
UNC93A	0.001728784	
C21orf33	0.001728784	
MYO16	0.001728784	
FAIM	0.001728784	
ZNF836	0.001728784	
HOXD11	0.001728784	
TMEM158	0.001728784	
CALML4	0.001728784	
PARP6	0.001728784	
ERICH1	0.001728784	
TBC1D31	0.001728784	
ADD2	0.001728784	
RP11-61J19.5	0.001728784	
DYNC2H1	0.001728784	
RP1-39G22.7	0.001728784	
OLIG3	0.001728784	
MYO19	0.001728784	
ITGAD	0.001728784	
PDE6H	0.001728784	
CELSR1	0.001728784	
ASAH2B	0.001728784	

RBM26	0.001728784	
ZMAT3	0.001728784	
ZNF486	0.001728784	
TMEM167B	0.001728784	
EFCAB10	0.001728784	
SPNS3	0.001728784	
ZNF786	0.001728784	
KLK6	0.001728784	
NPB	0.001728784	
ARSF	0.001728784	
KRT222	0.001728784	
SIRPB1	0.001728784	
ODF1	0.001728784	
NCAN	0.001728784	
DCUN1D5	0.001728784	
ATP6V1C2	0.001728784	
GABRR1	0.001728784	
ATG4A	0.001728784	
FAM221B	0.001728784	
PAQR8	0.001728784	
NANOS1	0.001728784	
ZDHHC11	0.001728784	
TBCAP1	0.001728784	
ZNF525	0.001728784	
MPZL2	0.001728784	
IGLV2-8	0.001728784	
C20orf196	0.001728784	
STIM2	0.001728784	
DLL3	0.001728784	
LCNL1	0.001728784	
RSPH9	0.001728784	
TAC3	0.001728784	
RP9	0.001728784	
CAMKV	0.001728784	
LINC01146	0.001728784	
IL18R1	0.001728784	
GTPBP6	0.001728784	
PYY2	0.001728784	
PTPRR	0.001728784	
ZBED3	0.001728784	
CTB-25B13.12	0.001728784	

VPS54	0.001728784	
ANGPT4	0.001728784	
CLIC3	0.001728784	
PTGR2	0.001728784	
PHTF2	0.001728784	
RNF216P1	0.001728784	
MPC1	0.001728784	
RP3-395M20.12	0.001728784	
KBTBD2	0.001728784	
FREM2	0.001728784	
TIMM10B	0.001728784	
COA1	0.001728784	
PTGES3L	0.001728784	
KCNA3	0.001728784	
FAM73B	0.001728784	
MAGEB18	0.001728784	
TEN1-CDK3	0.001728784	
GPC2	0.001728784	
ST6GALNAC2	0.001728784	
MAPT-IT1	0.001728784	
CALML3	0.001728784	
KIF26B	0.001728784	
AAR2	0.001728784	
SBSPON	0.001677938	
CXCR2P1	0.001677938	
TMEM150A	0.001677938	
MUC5AC	0.001677938	
NRN1	0.001677938	
COX20	0.001677938	
ZNF829	0.001677938	
GDPGP1	0.001677938	
SLC16A7	0.001677938	
IGHV1-46	0.001677938	
SSX2	0.001677938	
SNHG20	0.001677938	
IL24	0.001677938	
ACKR3	0.001677938	
MYLK-AS1	0.001677938	
GIN1	0.001677938	
TMEM246	0.001677938	
ZNF536	0.001677938	

STAG3L5P-PVRIG2P-PILRB	0.001677938	
PCLO	0.001677938	
HARBI1	0.001677938	
HOXA-AS2	0.001677938	
S100A7	0.001677938	
U5	0.001677938	
CTD-2270P14.1	0.001677938	
PLEKHG3	0.001677938	
CLDN12	0.001677938	
GID4	0.001677938	
BCL7A	0.001677938	
HTR3A	0.001677938	
KIAA0408	0.001677938	
GABRB3	0.001677938	
ZC3H12C	0.001677938	
IMPACT	0.001677938	
STAB2	0.001677938	
KDM4C	0.001677938	
FGF11	0.001677938	
CTD-2134A5.4	0.001677938	
COPG1	0.001677938	
C3orf38	0.001677938	
ZFP1	0.001677938	
SLC4A5	0.001677938	
NSUN5P1	0.001677938	
FAM212B	0.001677938	
CHAC1	0.001677938	
RP5-1154L15.2	0.001677938	
CYB561A3	0.001677938	
FLJ12825	0.001677938	
SLC43A2	0.001677938	
RP11-12G12.7	0.001677938	
AF064858.6	0.001677938	
LCT	0.001677938	
KIF21B	0.001677938	
NPBWR1	0.001677938	
KCND3	0.001677938	
KIF26A	0.001677938	
UBE2Q2P1	0.001677938	
ODF3L1	0.001677938	
NRIP3	0.001677938	

SNX33	0.001677938	
EIF3CL	0.001677938	
LINC01272	0.001677938	
DUOXA2	0.001677938	
NLRX1	0.001677938	
NAV3	0.001677938	
LRMP	0.001677938	
FAM117A	0.001677938	
ATAD3C	0.001677938	
HID1	0.001677938	
HIST2H2BF	0.001677938	
SVIP	0.001677938	
C17orf96	0.001677938	
DENND2A	0.001677938	
KBTBD8	0.001677938	
CCZ1	0.001677938	
ZNF211	0.001677938	
TRH	0.001677938	
HOXA10-HOXA9	0.001677938	
RP11-175O19.4	0.001677938	
DYDC1	0.001677938	
AGR3	0.001677938	
CTC-575D19.1	0.001677938	
CACNA2D3	0.001677938	
KBTBD3	0.001677938	
BAIAP2L2	0.001677938	
ZNF552	0.001677938	
GPR133	0.001677938	
CCL4L1	0.001677938	
HOXC12	0.001677938	
JMJD7-PLA2G4B	0.001677938	
PIEZO2	0.001677938	
PCP2	0.001677938	
EIF5AL1	0.001677938	
OSR1	0.001677938	
CDON	0.001677938	
CLDN6	0.001677938	
MAEL	0.001677938	
RP11-223I10.1	0.001677938	
CIDEC	0.001677938	
FBXO6	0.001677938	

MFSD8	0.001677938	
NSRP1	0.001677938	
SLC6A4	0.001677938	
ALS2CR11	0.001677938	
MTSS1L	0.001677938	
HCRT	0.001677938	
CCL23	0.001677938	
LINC00890	0.001677938	
TFF2	0.001677938	
SORCS2	0.001677938	
NYNRIN	0.001677938	
IFFO2	0.001677938	
MIR3682	0.001677938	
TMEM163	0.001677938	
LRRK2	0.001677938	
SCARNA13	0.001677938	
GCNT2	0.001677938	
LILRB5	0.001677938	
GLMP	0.001677938	
ZNF600	0.001677938	
WFIKKN1	0.001677938	
SNX18	0.001677938	
LINC01547	0.001677938	
FBXO36	0.001677938	
ATP13A2	0.001677938	
XPNPEP2	0.001677938	
EFCAB2	0.001677938	
TMEM184B	0.001677938	
FIGN	0.001677938	
RGS7BP	0.001677938	
RPL39L	0.001677938	
XKR8	0.001677938	
RAB28	0.001677938	
SNHG15	0.001677938	
B3GNTL1	0.001677938	
KCNN4	0.001677938	
RP11-884K10.7	0.001677938	
MAATS1	0.001677938	
PPM1J	0.001677938	
PNMA6A	0.001677938	
GUSBP1	0.001677938	

EXTL3	0.001677938	
AMN	0.001677938	
MRGPRF	0.001677938	
GDF10	0.001677938	
SCUBE3	0.001677938	
UPK3B	0.001677938	
ZNF431	0.001677938	
CCT6P1	0.001677938	
ATP8B2	0.001677938	
LINC00313	0.001677938	
SNORA28	0.001677938	
RIOK2	0.001677938	
TMX2	0.001677938	
FGF6	0.001677938	
S100A12	0.001677938	
CRYBB2	0.001677938	
PTPN5	0.001677938	
RAX	0.001677938	
ZNF488	0.001677938	
DUS4L	0.001677938	
HCG17	0.001677938	
RPL13P12	0.001677938	
CELF6	0.001677938	
RP1-228H13.5	0.001677938	
CTD-2012K14.8	0.001677938	
PLEKHM1P	0.001677938	
RP11-218M22.1	0.001677938	
EFTUD1	0.001677938	
INSL3	0.001677938	
AQP7P1	0.001677938	
ZNF395	0.001677938	
UBQLNL	0.001677938	
43711	0.001677938	
LYSMD4	0.001677938	
CCDC103	0.001677938	
ARL10	0.001677938	
DNAL1	0.001677938	
DLK2	0.001677938	
ZNF25	0.001677938	
ZNF736	0.001677938	
STARD7-AS1	0.001677938	

DZIP1L	0.001677938	
MTMR7	0.001677938	
SRCIN1	0.001677938	
HIST1H2BJ	0.001627091	
RP11-25K19.1	0.001627091	
EEPD1	0.001627091	
ST6GALNAC1	0.001627091	
TBX4	0.001627091	
TTLL10	0.001627091	
C2orf82	0.001627091	
PLA2G1B	0.001627091	
CHST4	0.001627091	
CAPN12	0.001627091	
IRAK1BP1	0.001627091	
SPEF2	0.001627091	
CNNM2	0.001627091	
SIT1	0.001627091	
PARP15	0.001627091	
FOLH1	0.001627091	
ARRDC1	0.001627091	
SLAIN1	0.001627091	
ZFP90	0.001627091	
WSCD1	0.001627091	
CNR1	0.001627091	
GPR155	0.001627091	
SCAMP5	0.001627091	
DLX3	0.001627091	
NAT8B	0.001627091	
BCL2L12	0.001627091	
MKX	0.001627091	
SECTM1	0.001627091	
ETAA1	0.001627091	
MATN1	0.001627091	
SERAC1	0.001627091	
CLDND1	0.001627091	
RP11-582E3.6	0.001627091	
FAM204A	0.001627091	
KIRREL3	0.001627091	
ZNF354A	0.001627091	
SDR16C5	0.001627091	
LRRC23	0.001627091	

C1orf95	0.001627091	
CCK	0.001627091	
IGHV3-11	0.001627091	
STATH	0.001627091	
GPM6B	0.001627091	
BOLA2B	0.001627091	
CD163L1	0.001627091	
CPNE6	0.001627091	
MSL1	0.001627091	
ANO10	0.001627091	
TMEM150B	0.001627091	
AGO4	0.001627091	
ZNF14	0.001627091	
C6orf57	0.001627091	
ALS2CR12	0.001627091	
NLGN3	0.001627091	
SNORA14B	0.001627091	
PCDHGA12	0.001627091	
FAM74A4	0.001627091	
PTH2R	0.001627091	
DYM	0.001627091	
RP11-452L6.5	0.001627091	
OLFM1	0.001627091	
CEP170B	0.001627091	
LRP12	0.001627091	
GABRB1	0.001627091	
NNT-AS1	0.001627091	
C5orf58	0.001627091	
RMRP	0.001627091	
LINC01578	0.001627091	
PPM1N	0.001627091	
ATRNL1	0.001627091	
RHBDL3	0.001627091	
TMEM194B	0.001627091	
GRM3	0.001627091	
MTND4P20	0.001627091	
KBTBD11	0.001627091	
PDIA2	0.001627091	
MIR4734	0.001627091	
TTC28-AS1	0.001627091	
CCDC7	0.001627091	

ZNF737	0.001627091	
LPIN3	0.001627091	
CEBPA-AS1	0.001627091	
OPHN1	0.001627091	
LIPA	0.001627091	
ZFP30	0.001627091	
KCNS2	0.001627091	
RHBDD1	0.001627091	
SYAP1	0.001627091	
KRT9	0.001627091	
LEKR1	0.001627091	
P2RY1	0.001627091	
ZNF69	0.001627091	
TMEM120B	0.001627091	
C21orf91	0.001627091	
HS3ST4	0.001627091	
CCL8	0.001627091	
AGPAT4	0.001627091	
SLC2A10	0.001627091	
TCP10L	0.001627091	
PYROXD2	0.001627091	
ABCA9	0.001627091	
ZNF337-AS1	0.001627091	
PTER	0.001627091	
ATP6V1G2	0.001627091	
SCGN	0.001627091	
PADI4	0.001627091	
CAPN11	0.001627091	
ZNF239	0.001627091	
GPR135	0.001627091	
ABLIM2	0.001627091	
SLC25A5-AS1	0.001627091	
C1orf186	0.001627091	
MAGEC1	0.001627091	
ADAM33	0.001627091	
KLHL7	0.001627091	
GPATCH11	0.001627091	
RPSAP58	0.001627091	
ASB14	0.001627091	
CEP112	0.001627091	
GPR176	0.001627091	

IGLV3-10	0.001627091	
C15orf59	0.001627091	
TMPRSS13	0.001627091	
GRIK3	0.001627091	
ZNF865	0.001627091	
TM6SF1	0.001627091	
RPLP0P2	0.001627091	
B3GAT2	0.001627091	
PLEKHH1	0.001627091	
PCDHB16	0.001627091	
RP5-1136G13.2	0.001627091	
AL450992.2	0.001627091	
MLLT4-AS1	0.001627091	
CEBPZOS	0.001627091	
SAMD11	0.001627091	
SCNN1G	0.001627091	
CMA1	0.001627091	
KNSTRN	0.001627091	
ACPP	0.001627091	
PROCA1	0.001627091	
CCBL2	0.001627091	
LSAMP	0.001627091	
HYI	0.001627091	
AC004893.11	0.001627091	
B3GALT2	0.001627091	
C11orf24	0.001627091	
IL37	0.001627091	
PKD2L1	0.001627091	
ARHGAP44	0.001627091	
MEIG1	0.001627091	
SUGT1P3	0.001627091	
BBS5	0.001627091	
SLC5A5	0.001627091	
TMEM200C	0.001627091	
RAP1GAP2	0.001627091	
TIPIN	0.001627091	
VRK3	0.001627091	
MTHFD2L	0.001627091	
ACRC	0.001627091	
CAMK1G	0.001627091	
ZNF596	0.001627091	

KLRG1	0.001627091	
FRMD5	0.001627091	
FGF18	0.001627091	
KIAA1244	0.001627091	
CLEC4M	0.001627091	
B3GNT5	0.001627091	
PRICKLE4	0.001627091	
STARD5	0.001627091	
DNAJC28	0.001627091	
P2RX3	0.001627091	
DRAM2	0.001627091	
CDX4	0.001627091	
UBAP1	0.001627091	
ASPRV1	0.001576244	
DUOX1	0.001576244	
UPK1B	0.001576244	
C9orf139	0.001576244	
DNLZ	0.001576244	
LDB3	0.001576244	
DAGLA	0.001576244	
FGF4	0.001576244	
CYP4X1	0.001576244	
HBE1	0.001576244	
GPR113	0.001576244	
ST7-AS1	0.001576244	
SIPA1L2	0.001576244	
SYNE4	0.001576244	
PRSS53	0.001576244	
ZNF85	0.001576244	
NETO2	0.001576244	
SMAGP	0.001576244	
BCL2L10	0.001576244	
CHRNA5	0.001576244	
SNORD12B	0.001576244	
PRPH	0.001576244	
GPR180	0.001576244	
RNF122	0.001576244	
FLRT3	0.001576244	
CORO6	0.001576244	
TMPRSS9	0.001576244	
RP11-121C2.2	0.001576244	

CEND1	0.001576244	
RP11-1072A3.3	0.001576244	
CCDC114	0.001576244	
GPR137C	0.001576244	
FBLN7	0.001576244	
SPRED3	0.001576244	
LINC00114	0.001576244	
ITGA10	0.001576244	
LCA5L	0.001576244	
CLSTN3	0.001576244	
TPCN2	0.001576244	
LRRC3-AS1	0.001576244	
DNAH10OS	0.001576244	
ITM2A	0.001576244	
MYT1L	0.001576244	
ZNF391	0.001576244	
SMIM13	0.001576244	
DNM3	0.001576244	
MIR155HG	0.001576244	
FAM133B	0.001576244	
RP5-907C10.3	0.001576244	
HIPK4	0.001576244	
GRM7	0.001576244	
ZNF286A	0.001576244	
PHYKPL	0.001576244	
DPPA4	0.001576244	
IQSEC2	0.001576244	
GABRB2	0.001576244	
CCDC153	0.001576244	
ZC3HAV1L	0.001576244	
ZNF778	0.001576244	
LRP4	0.001576244	
FGF19	0.001576244	
RCVRN	0.001576244	
OTOA	0.001576244	
COL9A3	0.001576244	
RPS2P5	0.001576244	
ZNF774	0.001576244	
ZNF223	0.001576244	
SYNC	0.001576244	
DNAJB4	0.001576244	

MTFMT	0.001576244	
METTL21B	0.001576244	
HS3ST6	0.001576244	
ALG6	0.001576244	
COL25A1	0.001576244	
GTF2IRD2	0.001576244	
MFSD7	0.001576244	
ZNF542P	0.001576244	
HSD11B2	0.001576244	
PPP1R14D	0.001576244	
DDN	0.001576244	
TMEM121	0.001576244	
SPATC1L	0.001576244	
FAM120C	0.001576244	
TBC1D2	0.001576244	
KCTD2	0.001576244	
SSX5	0.001576244	
FAM122C	0.001576244	
WASH7P	0.001576244	
ARL11	0.001576244	
LINC00909	0.001576244	
DHH	0.001576244	
PSRC1	0.001576244	
KCNK9	0.001576244	
RUNDC3B	0.001576244	
ZNF132	0.001576244	
DDX55	0.001576244	
SLC38A5	0.001576244	
POMZP3	0.001576244	
TPCN1	0.001576244	
SPINK7	0.001576244	
NXF3	0.001576244	
SLMO1	0.001576244	
LINC00885	0.001576244	
LRRC7	0.001576244	
C5orf28	0.001576244	
ABCC8	0.001576244	
INHBA-AS1	0.001576244	
SPIDR	0.001576244	
MUM1L1	0.001576244	
CDK5R2	0.001576244	

CCL13	0.001576244	
TSNAXIP1	0.001576244	
LRRC48	0.001576244	
ANKRD55	0.001576244	
SPATA18	0.001576244	
ZMYM6	0.001576244	
SPRR3	0.001576244	
KISS1R	0.001576244	
FOLH1B	0.001576244	
HM13-AS1	0.001576244	
SLC17A7	0.001576244	
C19orf26	0.001576244	
LYNX1	0.001576244	
ZNF783	0.001576244	
KIAA1875	0.001576244	
SLC16A9	0.001576244	
RHPN1-AS1	0.001576244	
SLC9A2	0.001576244	
SLC6A3	0.001576244	
SNORA4	0.001576244	
RNASEH2B-AS1	0.001576244	
ZFY	0.001576244	
MAP2K4P1	0.001576244	
GALNT12	0.001576244	
C8orf48	0.001576244	
MYOZ2	0.001576244	
GLRB	0.001576244	
FAM86C1	0.001576244	
GALNT4	0.001576244	
RP11-479G22.8	0.001576244	
BANK1	0.001576244	
FLG	0.001576244	
COX6B2	0.001576244	
B3GAT1	0.001576244	
CREBL2	0.001576244	
NPIPB15	0.001576244	
NUPL2	0.001576244	
C11orf16	0.001576244	
VASH2	0.001576244	
HCG25	0.001576244	
TPSB2	0.001576244	

CKMT1A	0.001576244	
PLAC8	0.001576244	
MTG1	0.001576244	
HIST1H2BA	0.001576244	
VMP1	0.001576244	
FAM154B	0.001576244	
CLDN19	0.001576244	
RP11-434D9.1	0.001576244	
ATG9B	0.001576244	
C18orf8	0.001576244	
ZNF680	0.001576244	
LRRC34	0.001576244	
D2HGDH	0.001576244	
TNFSF4	0.001576244	
DOK7	0.001576244	
NUDT17	0.001576244	
ITGB6	0.001576244	
NLRP12	0.001576244	
ELFN2	0.001576244	
KRTCAP3	0.001576244	
DFNB59	0.001576244	
GFRA3	0.001576244	
NDUFA6-AS1	0.001576244	
TRIM67	0.001576244	
TSHB	0.001576244	
DNHD1	0.001576244	
ADAMDEC1	0.001576244	
TAC4	0.001576244	
SLC25A16	0.001576244	
LINC00473	0.001576244	
PFN4	0.001576244	
EID3	0.001576244	
NTAN1	0.001576244	
FAM78B	0.001576244	
RP11-81A22.4	0.001576244	
C17orf82	0.001576244	
ADARB2	0.001576244	
FAM53A	0.001576244	
SLC1A3	0.001576244	
PAGR1	0.001525398	
SOSTDC1	0.001525398	

SLC48A1	0.001525398	
ZNF275	0.001525398	
ANKDD1A	0.001525398	
WASH3P	0.001525398	
IL17D	0.001525398	
SPOCK3	0.001525398	
INTU	0.001525398	
PCSK1N	0.001525398	
HS2ST1	0.001525398	
ZDHHC4	0.001525398	
GPCPD1	0.001525398	
LPPR4	0.001525398	
SCN4A	0.001525398	
FKRP	0.001525398	
MYH4	0.001525398	
MURC	0.001525398	
WDR19	0.001525398	
LINC01554	0.001525398	
SH2D1B	0.001525398	
CHRNA3	0.001525398	
ASTN1	0.001525398	
SLC35B4	0.001525398	
KRT33B	0.001525398	
LINC00607	0.001525398	
ZDHHC18	0.001525398	
NKX1-2	0.001525398	
RP5-991G20.1	0.001525398	
PRPS1L1	0.001525398	
NPPC	0.001525398	
PTGES3L-AARSD1	0.001525398	
ZNF704	0.001525398	
GUCA1B	0.001525398	
RNF170	0.001525398	
MGC16275	0.001525398	
CYP27B1	0.001525398	
SBK1	0.001525398	
C16orf74	0.001525398	
CTBS	0.001525398	
ZNRF2P1	0.001525398	
C4orf32	0.001525398	
C10orf128	0.001525398	

ZNF79	0.001525398	
CMYA5	0.001525398	
LINC00570	0.001525398	
RBM11	0.001525398	
FLVCR1-AS1	0.001525398	
RP11-342K6.1	0.001525398	
MIR4458HG	0.001525398	
GPR19	0.001525398	
ZDHHC19	0.001525398	
CTD-2521M24.9	0.001525398	
NPBWR2	0.001525398	
KATNAL2	0.001525398	
UBAC2-AS1	0.001525398	
ANKRD29	0.001525398	
SCARNA12	0.001525398	
ARHGEF35	0.001525398	
FBXO21	0.001525398	
LINC00176	0.001525398	
CTD-2034I21.2	0.001525398	
ST8SIA5	0.001525398	
SP7	0.001525398	
TMEM201	0.001525398	
PPAPDC1B	0.001525398	
RBM19	0.001525398	
PCDHGA3	0.001525398	
DKFZP434I0714	0.001525398	
FAM27E3	0.001525398	
LIAS	0.001525398	
MOSPD3	0.001525398	
TEN1	0.001525398	
OTOG	0.001525398	
FAM177B	0.001525398	
KLLN	0.001525398	
KIAA1551	0.001525398	
WFDC3	0.001525398	
LINC00641	0.001525398	
PTCHD1	0.001525398	
LURAP1	0.001525398	
GRIA4	0.001525398	
FASTKD1	0.001525398	
FHOD3	0.001525398	

LINC01320	0.001525398	
SGCG	0.001525398	
CDH18	0.001525398	
MSH5-SAPCD1	0.001525398	
SCARNA11	0.001525398	
LINC01569	0.001525398	
SESN2	0.001525398	
SAP25	0.001525398	
ATP8B1	0.001525398	
RP11-441O15.3	0.001525398	
GTPBP8	0.001525398	
TMEM45A	0.001525398	
PCYOX1L	0.001525398	
ZNF782	0.001525398	
C16orf62	0.001525398	
AP001258.4	0.001525398	
ZNF287	0.001525398	
PRDM12	0.001525398	
DENND1C	0.001525398	
CD70	0.001525398	
CD177	0.001525398	
CUBN	0.001525398	
PCDHA3	0.001525398	
C3orf14	0.001525398	
LINC00294	0.001525398	
LCE1B	0.001525398	
OPCML	0.001525398	
PASK	0.001525398	
ZIK1	0.001525398	
C2orf15	0.001525398	
AC005077.14	0.001525398	
C19orf38	0.001525398	
DYX1C1	0.001525398	
EFCAB5	0.001525398	
ZNF682	0.001525398	
ZNF254	0.001525398	
NFE4	0.001525398	
FAM110B	0.001525398	
ALG8	0.001525398	
SCGB1A1	0.001525398	
PRICKLE2	0.001525398	

PITX3	0.001525398	
KCNQ5	0.001525398	
GLUD1P3	0.001525398	
LRRC57	0.001525398	
OMG	0.001525398	
TTC28	0.001525398	
LINC01270	0.001525398	
KIAA1614	0.001525398	
KLHL42	0.001525398	
CNTNAP4	0.001525398	
SPATC1	0.001525398	
43533	0.001525398	
FAM114A2	0.001525398	
ZNF530	0.001525398	
PI15	0.001525398	
WNT7A	0.001525398	
ANKRD42	0.001525398	
KLHL35	0.001525398	
RP3-425C14.4	0.001525398	
AC011899.9	0.001525398	
CCDC66	0.001525398	
LINC00087	0.001525398	
UNC93B1	0.001525398	
ZNF385D	0.001525398	
TMEM206	0.001525398	
RASAL1	0.001525398	
RHD	0.001525398	
CASC2	0.001525398	
MRS2	0.001525398	
NTN3	0.001525398	
FOXC2	0.001525398	
PIWIL2	0.001525398	
KIR2DL4	0.001525398	
SLC13A4	0.001525398	
MYOZ1	0.001525398	
FAM133A	0.001474551	
CD101	0.001474551	
KIAA1586	0.001474551	
KIAA1407	0.001474551	
DSC1	0.001474551	
PNLIPRP1	0.001474551	

CTA-445C9.14	0.001474551	
WDR90	0.001474551	
NBPF1	0.001474551	
NACC2	0.001474551	
FO538757.2	0.001474551	
KCNIP1	0.001474551	
MCMDC2	0.001474551	
LPAR6	0.001474551	
RBM12B-AS1	0.001474551	
ANKRD49	0.001474551	
LRRC73	0.001474551	
TBX18	0.001474551	
SERTAD4	0.001474551	
LRWD1	0.001474551	
TCEAL5	0.001474551	
LAMB4	0.001474551	
SPEF1	0.001474551	
ANKRD46	0.001474551	
TBC1D20	0.001474551	
MTCP1	0.001474551	
CCPG1	0.001474551	
RP11-390P24.1	0.001474551	
HRASLS2	0.001474551	
FSHB	0.001474551	
ZSCAN31	0.001474551	
FCGR2C	0.001474551	
C1orf220	0.001474551	
AC091729.9	0.001474551	
PRR16	0.001474551	
RP11-48B3.4	0.001474551	
C9orf40	0.001474551	
ATP8B3	0.001474551	
DENND6A	0.001474551	
SNPH	0.001474551	
GREM1	0.001474551	
GSTM1	0.001474551	
SYS1-DBNDD2	0.001474551	
BEND6	0.001474551	
BSN	0.001474551	
SLC25A30	0.001474551	
DSCAML1	0.001474551	

RP11-467L13.7	0.001474551	
REP15	0.001474551	
SEMA4F	0.001474551	
HOXD1	0.001474551	
IPO5P1	0.001474551	
APOBEC2	0.001474551	
ERGIC2	0.001474551	
C18orf54	0.001474551	
WHAMML1	0.001474551	
NDST2	0.001474551	
PPP4R1L	0.001474551	
HSD17B12	0.001474551	
PARP12	0.001474551	
ZNF28	0.001474551	
MRO	0.001474551	
P2RX5	0.001474551	
METTL2B	0.001474551	
GPR137	0.001474551	
FAM177A1	0.001474551	
ERAP2	0.001474551	
CTNNA2	0.001474551	
RP11-20I20.4	0.001474551	
FIG4	0.001474551	
FRAT2	0.001474551	
ZNF788	0.001474551	
ZBTB14	0.001474551	
FAM71E1	0.001474551	
PIGW	0.001474551	
TULP4	0.001474551	
ACR	0.001474551	
MIR4787	0.001474551	
HMCES	0.001474551	
ULK3	0.001474551	
ICAM3	0.001474551	
ZNF71	0.001474551	
GDPD3	0.001474551	
CLN8	0.001474551	
IDH1-AS1	0.001474551	
ATP10D	0.001474551	
TUB	0.001474551	
CTA-292E10.9	0.001474551	

EGR4	0.001474551	
GDF5	0.001474551	
METTL10	0.001474551	
DANCR	0.001474551	
FAM174B	0.001474551	
ZNF106	0.001474551	
SPATA7	0.001474551	
MIR4292	0.001474551	
HIST2H2AB	0.001474551	
VTCN1	0.001474551	
ARL14EP	0.001474551	
PCDHGB1	0.001474551	
ZNF845	0.001474551	
CMTM4	0.001474551	
LBHD1	0.001474551	
CYSLTR2	0.001474551	
LIN28B	0.001474551	
THSD7A	0.001474551	
PCDHGA4	0.001474551	
KDELR3	0.001474551	
ERICH2	0.001474551	
STAG3L3	0.001474551	
PCDHA6	0.001474551	
MCM3AP-AS1	0.001474551	
CCL3L3	0.001474551	
TMEM92	0.001474551	
GPR35	0.001474551	
PWP2	0.001474551	
STARD8	0.001474551	
SIGLEC14	0.001474551	
SLC25A29	0.001474551	
FAM110D	0.001474551	
RP1-283E3.4	0.001474551	
KCNQ1DN	0.001474551	
TEX41	0.001474551	
SCARA3	0.001474551	
SLC22A2	0.001474551	
RP11-712L6.5	0.001474551	
EIF4BP7	0.001474551	
NEUROG1	0.001474551	
ZNF181	0.001474551	

RP11-458J1.1	0.001474551	
XCL2	0.001474551	
FLJ38576	0.001474551	
SEC14L5	0.001474551	
ARC	0.001474551	
DHRS11	0.001474551	
IDO2	0.001474551	
ZNF117	0.001474551	
NBPF15	0.001474551	
CCDC17	0.001474551	
XKR9	0.001474551	
MMRN1	0.001474551	
MYBPHL	0.001474551	
CRTAC1	0.001474551	
MPP2	0.001474551	
TACR2	0.001474551	
MROH2B	0.001474551	
NPIPA1	0.001474551	
KNOP1	0.001474551	
ZNF777	0.001474551	
ARSK	0.001474551	
ZC3H12B	0.001474551	
BARX1	0.001474551	
ANKRD37	0.001474551	
RP11-159D12.2	0.001474551	
ARHGEF19	0.001474551	
TSLP	0.001474551	
SETD9	0.001474551	
ZNF830	0.001474551	
KLHDC7B	0.001474551	
MS4A14	0.001474551	
RAET1G	0.001474551	
EDRF1	0.001474551	
KCNJ6	0.001474551	
MRAP	0.001474551	
FAM188A	0.001474551	
PDIA3P1	0.001474551	
MTERF4	0.001474551	
ZNF846	0.001474551	
C9orf3	0.001474551	
RITA1	0.001474551	

GPR27	0.001474551	
SHISA3	0.001474551	
RP11-213G2.3	0.001474551	
FRMD6-AS1	0.001474551	
PMS2P5	0.001474551	
HPDL	0.001474551	
REG4	0.001474551	
CNTN3	0.001474551	
GHRHR	0.001474551	
FAM200A	0.001474551	
TMEM168	0.001474551	
MBD3L1	0.001474551	
ACTR3C	0.001474551	
TRDC	0.001474551	
TCF23	0.001474551	
PLEKHA8P1	0.001474551	
STK4-AS1	0.001474551	
PXDNL	0.001474551	
ZNF518B	0.001474551	
NCR3	0.001474551	
ATP8B4	0.001474551	
WRB	0.001474551	
LEPROT	0.001474551	
MIR26B	0.001474551	
DLK1	0.001474551	
CLCC1	0.001474551	
LGALS14	0.001474551	
PSPN	0.001474551	
AMN1	0.001474551	
RP11-37B2.1	0.001423705	
TMEM50B	0.001423705	
FLCN	0.001423705	
NPY5R	0.001423705	
RGS6	0.001423705	
C6orf223	0.001423705	
ATP1B4	0.001423705	
RP11-156K23.3	0.001423705	
CYP26C1	0.001423705	
GPRIN2	0.001423705	
SCML4	0.001423705	
PRRG3	0.001423705	

B4GALNT3	0.001423705	
ARL8A	0.001423705	
FGFBP3	0.001423705	
CATSPER2P1	0.001423705	
ADCK2	0.001423705	
GULP1	0.001423705	
DIRAS3	0.001423705	
ZNF587B	0.001423705	
SENP5	0.001423705	
CSMD2	0.001423705	
CSMD1	0.001423705	
LRRC46	0.001423705	
LINC00167	0.001423705	
GPR162	0.001423705	
CNFN	0.001423705	
CECR5-AS1	0.001423705	
FAAH2	0.001423705	
TRPC4	0.001423705	
SAMD12	0.001423705	
AURKC	0.001423705	
FAM178B	0.001423705	
PNLIP	0.001423705	
C2orf43	0.001423705	
CALML5	0.001423705	
CTD-2336O2.1	0.001423705	
PCDHB3	0.001423705	
ABCA7	0.001423705	
TCEAL2	0.001423705	
CCDC68	0.001423705	
PRSS21	0.001423705	
BTLA	0.001423705	
TNFSF14	0.001423705	
GRIK1	0.001423705	
TNFSF9	0.001423705	
IL1RL2	0.001423705	
NDUFAF7	0.001423705	
TRAPPC2B	0.001423705	
NETO1	0.001423705	
C2orf49	0.001423705	
IFT46	0.001423705	
EXOC3L4	0.001423705	

LINC01426	0.001423705	
CHST15	0.001423705	
CENPBD1P1	0.001423705	
FRMD4A	0.001423705	
GAPDHS	0.001423705	
ZG16B	0.001423705	
LRRC10B	0.001423705	
NRARP	0.001423705	
IZUMO1	0.001423705	
SYNGR3	0.001423705	
ZNF624	0.001423705	
IFT122	0.001423705	
SLFN13	0.001423705	
ZFHX2	0.001423705	
CTBP1-AS2	0.001423705	
GRP	0.001423705	
TMEM108	0.001423705	
BBS9	0.001423705	
C16orf71	0.001423705	
RNF215	0.001423705	
WT1-AS	0.001423705	
HCCS	0.001423705	
SFT2D2	0.001423705	
RAB41	0.001423705	
DAZL	0.001423705	
ARMCX4	0.001423705	
C4orf3	0.001423705	
LINC00094	0.001423705	
KCNN1	0.001423705	
HKR1	0.001423705	
DGCR6	0.001423705	
PCDHAC2	0.001423705	
RPGRIP1L	0.001423705	
RBP2	0.001423705	
COL9A1	0.001423705	
CAHM	0.001423705	
ASB16	0.001423705	
FAM135B	0.001423705	
FAM86EP	0.001423705	
ZNF253	0.001423705	
RP11-452G18.2	0.001423705	

FAM156A	0.001423705	
GPR97	0.001423705	
PQLC3	0.001423705	
CEACAM21	0.001423705	
CHKB-CPT1B	0.001423705	
RP11-156K13.1	0.001423705	
ADAMTS3	0.001423705	
WFDC2	0.001423705	
JADE1	0.001423705	
GABRE	0.001423705	
DIS3L2	0.001423705	
EDN2	0.001423705	
SYT12	0.001423705	
GLP1R	0.001423705	
OTULIN	0.001423705	
LRP5L	0.001423705	
TMEM132E	0.001423705	
LGALS7	0.001423705	
HDX	0.001423705	
GPR55	0.001423705	
UNC13A	0.001423705	
TREML1	0.001423705	
CTB-36H16.2	0.001423705	
TRIM7	0.001423705	
PRRT3	0.001423705	
BMP5	0.001423705	
TMEM185A	0.001423705	
CITF22-92A6.1	0.001423705	
CX3CR1	0.001423705	
SLC35E2	0.001423705	
SUSD3	0.001423705	
BCL2L15	0.001423705	
ZNF780A	0.001423705	
SLC52A3	0.001423705	
CACNA1E	0.001423705	
KLHL7-AS1	0.001423705	
MAP6D1	0.001423705	
SYPL2	0.001423705	
VPS33B	0.001423705	
DMBT1	0.001423705	
SPRR1A	0.001423705	

ST13P4	0.001423705	
LYPD1	0.001423705	
RP1-313I6.12	0.001423705	
VKORC1L1	0.001423705	
MROH8	0.001423705	
DYDC2	0.001423705	
CARD8-AS1	0.001423705	
RFTN2	0.001423705	
FAM13A-AS1	0.001423705	
MROH2A	0.001423705	
DMRTA1	0.001423705	
CSN1S1	0.001423705	
SIGLEC11	0.001423705	
CIDEA	0.001423705	
MIR3666	0.001423705	
LCE2D	0.001423705	
HCG4P5	0.001423705	
NDP	0.001423705	
NCR1	0.001423705	
FIBIN	0.001423705	
NPAS3	0.001423705	
ZNF324	0.001423705	
ZNF683	0.001423705	
TCTEX1D2	0.001423705	
ASIC2	0.001423705	
DGCR11	0.001423705	
RNASEH1-AS1	0.001423705	
ZACN	0.001423705	
GPX7	0.001423705	
AC068580.6	0.001423705	
THNSL2	0.001423705	
SLC14A1	0.001423705	
CBWD5	0.001423705	
CROT	0.001423705	
ESRRB	0.001423705	
PPP1R36	0.001423705	
GJC3	0.001372858	
NPPA-AS1	0.001372858	
CNTNAP3B	0.001372858	
YBX1P10	0.001372858	
NBPF19	0.001372858	

PGBD5	0.001372858	
SLC45A2	0.001372858	
KCNAB1	0.001372858	
LOH12CR2	0.001372858	
TMUB2	0.001372858	
HIST1H2AH	0.001372858	
KCNA4	0.001372858	
FAM24B	0.001372858	
SNORD94	0.001372858	
RHBDF2	0.001372858	
MESP2	0.001372858	
ZNF880	0.001372858	
KHDC1	0.001372858	
LY6H	0.001372858	
CLRN3	0.001372858	
SOWAHD	0.001372858	
RPL23AP53	0.001372858	
ROPN1B	0.001372858	
SLC25A19	0.001372858	
HIST1H2AD	0.001372858	
SCG3	0.001372858	
BMS1P1	0.001372858	
IGSF22	0.001372858	
KRTAP10-11	0.001372858	
USP6	0.001372858	
RBM14-RBM4	0.001372858	
CACNA2D2	0.001372858	
FAM172A	0.001372858	
CRYM	0.001372858	
UQCC1	0.001372858	
ELMOD3	0.001372858	
TCP11L1	0.001372858	
C12orf4	0.001372858	
CLDN23	0.001372858	
SNORA60	0.001372858	
CADM3	0.001372858	
TPSAB1	0.001372858	
SLC22A20	0.001372858	
MLNR	0.001372858	
KRT14	0.001372858	
PAX7	0.001372858	

ADAMTS16	0.001372858	
TMEM56-RWDD3	0.001372858	
PPAPDC1A	0.001372858	
CELA1	0.001372858	
NUDT11	0.001372858	
POF1B	0.001372858	
CCDC77	0.001372858	
SLC11A1	0.001372858	
DKFZP434H168	0.001372858	
ZNF558	0.001372858	
FSD1	0.001372858	
EDAR	0.001372858	
RHOV	0.001372858	
CTAG1B	0.001372858	
CLDN10	0.001372858	
KCNAB3	0.001372858	
CIART	0.001372858	
ZNF26	0.001372858	
OR7E14P	0.001372858	
FUT7	0.001372858	
SPATA33	0.001372858	
LINC00466	0.001372858	
ZNF720	0.001372858	
MYH2	0.001372858	
NUP62CL	0.001372858	
LAX1	0.001372858	
WASF3	0.001372858	
ALG14	0.001372858	
MYLK4	0.001372858	
CYP26B1	0.001372858	
CPA6	0.001372858	
WFIKKN2	0.001372858	
HYLS1	0.001372858	
C17orf97	0.001372858	
FAM134B	0.001372858	
CRHR1-IT1	0.001372858	
ADAMTS10	0.001372858	
RP11-767N6.7	0.001372858	
ALOXE3	0.001372858	
RP13-616I3.1	0.001372858	
C1orf204	0.001372858	

CTRB1	0.001372858	
AARSD1	0.001372858	
RNF141	0.001372858	
SYCP3	0.001372858	
TCHH	0.001372858	
CPA1	0.001372858	
C5orf45	0.001372858	
CHORDC1	0.001372858	
CYSLTR1	0.001372858	
TOB2P1	0.001372858	
RCCD1	0.001372858	
KREMEN2	0.001372858	
KCNE2	0.001372858	
SFRP5	0.001372858	
CNTD1	0.001372858	
ADAM23	0.001372858	
LCA5	0.001372858	
GABRP	0.001372858	
IL22	0.001372858	
chr22-38_28785274-29006793.1	0.001372858	
TSPY26P	0.001372858	
CTB-31O20.2	0.001372858	
ZNF702P	0.001372858	
WWTR1-AS1	0.001372858	
KSR2	0.001372858	
SYCE1L	0.001372858	
RBP3	0.001372858	
FAM71D	0.001372858	
RASSF8-AS1	0.001372858	
KCNJ9	0.001372858	
PCDH10	0.001372858	
CXorf65	0.001372858	
CD99L2	0.001372858	
RAC1P2	0.001372858	
PGGT1B	0.001372858	
AGBL3	0.001372858	
GIP	0.001372858	
DACH2	0.001372858	
GABRR2	0.001372858	
HS6ST3	0.001372858	
AK8	0.001372858	

C16orf46	0.001372858	
RP11-488L18.10	0.001372858	
CASKIN1	0.001372858	
TRIT1	0.001372858	
IQGAP3	0.001372858	
BPIFB2	0.001372858	
ZFX-AS1	0.001372858	
NEGR1	0.001372858	
DIRAS1	0.001372858	
PAGE5	0.001372858	
ZNF366	0.001372858	
NAP1L3	0.001372858	
ZDHHC21	0.001372858	
CYP20A1	0.001372858	
FSTL4	0.001372858	
RP11-644F5.10	0.001372858	
NPM2	0.001372858	
OTP	0.001372858	
PINK1-AS	0.001372858	
CTD-2284J15.1	0.001372858	
CCDC81	0.001372858	
SPRR2A	0.001372858	
PCDHGB6	0.001372858	
MSS51	0.001372858	
PLA1A	0.001372858	
LGALS8-AS1	0.001372858	
KNDC1	0.001372858	
KLRC2	0.001372858	
PROB1	0.001372858	
GNRHR2	0.001372858	
SMIM22	0.001372858	
CXCL3	0.001372858	
SFTPB	0.001372858	
KCNJ15	0.001372858	
KIAA1324L	0.001372858	
RBM34	0.001372858	
PTCHD4	0.001372858	
C22orf24	0.001372858	
HNRNPA1L2	0.001372858	
FCRLB	0.001372858	
ZIC5	0.001372858	

ADAM2	0.001372858	
ZFP92	0.001372858	
SNHG4	0.001372858	
C1QTNF7	0.001372858	
CCNJ	0.001372858	
D4S234E	0.001372858	
MYO7B	0.001372858	
CATSPER2	0.001372858	
CEACAM5	0.001372858	
PGBD2	0.001372858	
CLEC1B	0.001372858	
STAG3L4	0.001372858	
C9orf163	0.001372858	
STOX2	0.001372858	
RAB33A	0.001372858	
CTA-29F11.1	0.001372858	
MIR100HG	0.001372858	
QPCT	0.001372858	
TIGD3	0.001372858	
ERMN	0.001372858	
PSG9	0.001372858	
H3F3AP6	0.001372858	
CEP164	0.001372858	
GALNT18	0.001372858	
BCL2L2-PABPN1	0.001372858	
CARNS1	0.001372858	
RP11-342K2.1	0.001322011	
SGIP1	0.001322011	
JMJD7	0.001322011	
ZIC3	0.001322011	
FMO2	0.001322011	
KCNJ1	0.001322011	
SV2B	0.001322011	
PRG2	0.001322011	
SLC26A7	0.001322011	
VSIG1	0.001322011	
MIR324	0.001322011	
SLC34A3	0.001322011	
SYT16	0.001322011	
NRK	0.001322011	
MEG3	0.001322011	

NPFFR2	0.001322011	
GSG1	0.001322011	
MCF2L-AS1	0.001322011	
GGT3P	0.001322011	
MRPL23-AS1	0.001322011	
ST20-MTHFS	0.001322011	
MYLPF	0.001322011	
ZNF491	0.001322011	
RAB40B	0.001322011	
GATB	0.001322011	
MC5R	0.001322011	
SLC15A2	0.001322011	
GAL3ST2	0.001322011	
MYO15A	0.001322011	
ARMC2	0.001322011	
CCKBR	0.001322011	
IQCK	0.001322011	
MFAP2	0.001322011	
ATP1B2	0.001322011	
LINC00189	0.001322011	
GPR50	0.001322011	
RP11-328K4.1	0.001322011	
ST20-AS1	0.001322011	
SCAMP1-AS1	0.001322011	
SPATS2L	0.001322011	
LRRC37A3	0.001322011	
MIR143HG	0.001322011	
RP11-499P20.2	0.001322011	
CABP7	0.001322011	
HLA-F-AS1	0.001322011	
GUF1	0.001322011	
CLDN18	0.001322011	
ACRBP	0.001322011	
FBXL22	0.001322011	
TMEM161B-AS1	0.001322011	
FN3KRP	0.001322011	
ABCA10	0.001322011	
SNORD19	0.001322011	
HECW1	0.001322011	
ZNF584	0.001322011	
MIR3131	0.001322011	

RAB3C	0.001322011	
ZNRF3	0.001322011	
SAMD14	0.001322011	
DDI1	0.001322011	
APOBEC3H	0.001322011	
C11orf94	0.001322011	
ATP13A4	0.001322011	
CST1	0.001322011	
GSPT2	0.001322011	
TRAM1L1	0.001322011	
RPSAP9	0.001322011	
PCDHB4	0.001322011	
BDNF-AS	0.001322011	
LRRN4CL	0.001322011	
TIGD7	0.001322011	
CFAP74	0.001322011	
HAR1A	0.001322011	
SESTD1	0.001322011	
ZFP3	0.001322011	
NEURL1	0.001322011	
KLK10	0.001322011	
IFITM4P	0.001322011	
SLC35G2	0.001322011	
PRKAG2-AS1	0.001322011	
ADAM11	0.001322011	
CPA3	0.001322011	
THAP2	0.001322011	
ANKRD65	0.001322011	
NRG4	0.001322011	
KRT36	0.001322011	
SMCR5	0.001322011	
SERPINA9	0.001322011	
KLHL4	0.001322011	
CHODL	0.001322011	
LECT1	0.001322011	
GPR18	0.001322011	
TMEM254-AS1	0.001322011	
NUTM2B-AS1	0.001322011	
CC2D2A	0.001322011	
NMU	0.001322011	
MEI1	0.001322011	

SCGB1D1	0.001322011	
PHEX	0.001322011	
APOL4	0.001322011	
NSMCE4A	0.001322011	
SLC16A8	0.001322011	
DCD	0.001322011	
CADM2	0.001322011	
FLJ27354	0.001322011	
PDZD7	0.001322011	
ZNF19	0.001322011	
RHEBP2	0.001322011	
ZNF561-AS1	0.001322011	
CD200R1	0.001322011	
TMEM41A	0.001322011	
ZNF630	0.001322011	
ANKRD23	0.001322011	
NOSTRIN	0.001322011	
EPN3	0.001322011	
REM2	0.001322011	
GDPD1	0.001322011	
DUSP15	0.001322011	
PDC	0.001322011	
CHRNA10	0.001322011	
RP11-714M23.2	0.001322011	
CCDC65	0.001322011	
ZNF575	0.001322011	
FGF20	0.001322011	
MB21D1	0.001322011	
RAET1K	0.001322011	
ANKRD22	0.001322011	
HOXA6	0.001322011	
MIR99AHG	0.001322011	
GRK1	0.001322011	
PLCXD1	0.001322011	
FCN2	0.001322011	
BMS1	0.001322011	
KLK1	0.001322011	
C2orf54	0.001322011	
DDX12P	0.001322011	
ASIP	0.001322011	
FAM83F	0.001322011	

ZNF214	0.001322011	
PPFIA4	0.001322011	
ART4	0.001322011	
EFCAB12	0.001322011	
PPM1H	0.001322011	
HS6ST2	0.001322011	
GLUD1P7	0.001322011	
ROS1	0.001322011	
PCDHB11	0.001322011	
MUC3A	0.001322011	
TTC12	0.001322011	
PCDHB10	0.001322011	
WNT10B	0.001322011	
EGFL8	0.001322011	
DCST2	0.001322011	
ANKLE2	0.001322011	
C11orf95	0.001322011	
ASMTL-AS1	0.001322011	
SNORA33	0.001322011	
FGFBP1	0.001322011	
MDP1	0.001322011	
C22orf23	0.001322011	
ZSWIM1	0.001322011	
RETN	0.001322011	
RGAG1	0.001322011	
WDR72	0.001322011	
AC096772.6	0.001322011	
KCNC3	0.001322011	
MIR647	0.001322011	
NANP	0.001322011	
FAXDC2	0.001322011	
ANKAR	0.001322011	
SLC2A11	0.001322011	
SPTSSB	0.001322011	
ADAMTSL1	0.001322011	
EMILIN3	0.001322011	
SNORD12C	0.001322011	
EPHX3	0.001322011	
CEP83	0.001322011	
KIRREL2	0.001322011	
OR2A1-AS1	0.001271165	

NKAPL	0.001271165	
IFI30	0.001271165	
FGF9	0.001271165	
LINC01474	0.001271165	
NLGN1	0.001271165	
FCGR3B	0.001271165	
KL	0.001271165	
SMAD5-AS1	0.001271165	
PMS2CL	0.001271165	
ODF3L2	0.001271165	
CHRM1	0.001271165	
AKAP4	0.001271165	
TCTE3	0.001271165	
MYCL	0.001271165	
MMP28	0.001271165	
CCDC74A	0.001271165	
PPAPDC3	0.001271165	
C2orf74	0.001271165	
RNF39	0.001271165	
CFAP97	0.001271165	
RBM43	0.001271165	
GP2	0.001271165	
S100A5	0.001271165	
ZNF804A	0.001271165	
EP400NL	0.001271165	
ZNF296	0.001271165	
CBR3-AS1	0.001271165	
TAS1R1	0.001271165	
CATSPERD	0.001271165	
RP11-658F2.8	0.001271165	
PSTPIP2	0.001271165	
DLEU7	0.001271165	
GDF6	0.001271165	
ANKRD34A	0.001271165	
SLC28A1	0.001271165	
MTX1P1	0.001271165	
HBZ	0.001271165	
EFCAB13	0.001271165	
LINS	0.001271165	
LGI3	0.001271165	
RGS9BP	0.001271165	

CHIT1	0.001271165	
PMS2P3	0.001271165	
MMP25-AS1	0.001271165	
AAED1	0.001271165	
KDM4D	0.001271165	
SLC16A4	0.001271165	
TMEM191A	0.001271165	
RHCG	0.001271165	
SNORA65	0.001271165	
SLC14A2	0.001271165	
VAT1L	0.001271165	
MIR4737	0.001271165	
GPR61	0.001271165	
ZNF891	0.001271165	
RDH8	0.001271165	
ITLN1	0.001271165	
TRPV3	0.001271165	
LGSN	0.001271165	
FZD10	0.001271165	
ZNF333	0.001271165	
RAG2	0.001271165	
KRT6A	0.001271165	
TNNT3	0.001271165	
AMZ2	0.001271165	
MMP23A	0.001271165	
VCPKMT	0.001271165	
RP11-448A19.1	0.001271165	
GLP2R	0.001271165	
FAM161B	0.001271165	
MAP3K15	0.001271165	
AC007246.3	0.001271165	
MIR25	0.001271165	
LTB4R2	0.001271165	
MROH1	0.001271165	
BHLHE23	0.001271165	
C2orf81	0.001271165	
ZNF805	0.001271165	
CTD-2328D6.1	0.001271165	
C19orf71	0.001271165	
ENKUR	0.001271165	
RLN1	0.001271165	

HNRNPA1P10	0.001271165	
RP11-22B23.1	0.001271165	
DLX1	0.001271165	
TMEM110-MUSTN1	0.001271165	
LGI2	0.001271165	
MEMO1P1	0.001271165	
MAB21L3	0.001271165	
FBXO41	0.001271165	
SH3BGR	0.001271165	
SERPINB13	0.001271165	
LOH12CR1	0.001271165	
GRM5	0.001271165	
RPL7P32	0.001271165	
HAVCR1	0.001271165	
C2CD4B	0.001271165	
TMEM5	0.001271165	
UBIAD1	0.001271165	
YY2	0.001271165	
TSGA10IP	0.001271165	
IL18RAP	0.001271165	
SRL	0.001271165	
BAIAP3	0.001271165	
CABP4	0.001271165	
ZBED6CL	0.001271165	
COL10A1	0.001271165	
C9orf24	0.001271165	
NCF1B	0.001271165	
FOXD4	0.001271165	
UNC79	0.001271165	
MIR3188	0.001271165	
HUS1B	0.001271165	
DDTL	0.001271165	
PROM1	0.001271165	
TYW1B	0.001271165	
RBM26-AS1	0.001271165	
RAD9B	0.001271165	
RP3-395M20.8	0.001271165	
LRRN3	0.001271165	
HBG2	0.001271165	
P2RX5-TAX1BP3	0.001271165	
GRIA3	0.001271165	

SEBOX	0.001271165	
CSDC2	0.001271165	
VWCE	0.001271165	
MT1DP	0.001271165	
ADAMTS8	0.001271165	
TPSG1	0.001271165	
ZNF483	0.001271165	
HSF5	0.001271165	
C11orf45	0.001271165	
HIST2H2BC	0.001271165	
PRKAG3	0.001271165	
GS1-358P8.4	0.001271165	
DHRS7	0.001271165	
FBXL19-AS1	0.001271165	
THBS4	0.001271165	
LRP11	0.001271165	
DDO	0.001271165	
C1orf105	0.001271165	
TCTEX1D1	0.001271165	
BBS12	0.001271165	
VWA5B2	0.001271165	
PRDM6	0.001271165	
MAGEC2	0.001271165	
SH2D6	0.001271165	
RP11-134L10.1	0.001271165	
PVRL3-AS1	0.001271165	
RP11-132A1.6	0.001271165	
LINC00222	0.001271165	
OSR2	0.001271165	
MIR3646	0.001271165	
C3orf70	0.001271165	
TRIM36	0.001271165	
AC093627.10	0.001271165	
MNX1	0.001271165	
DKFZP434K028	0.001271165	
C1orf101	0.001271165	
ADPRHL1	0.001271165	
PAMR1	0.001271165	
USP35	0.001271165	
SNORA31	0.001271165	
TBRG1	0.001271165	

ABCA12	0.001271165	
PTGDR	0.001271165	
SPINK5	0.001271165	
DIO3OS	0.001271165	
CDH9	0.001271165	
CH25H	0.001271165	
RP11-21L23.2	0.001271165	
SLCO1C1	0.001271165	
FCRL3	0.001271165	
SLC6A20	0.001271165	
ASB16-AS1	0.001271165	
PLA2G10	0.001271165	
ZNF583	0.001220318	
MSTN	0.001220318	
SLC22A8	0.001220318	
PCDHB8	0.001220318	
CCDC108	0.001220318	
ZNF726	0.001220318	
TRIM50	0.001220318	
TEX14	0.001220318	
LINC00299	0.001220318	
C9orf66	0.001220318	
TEX19	0.001220318	
TAF7L	0.001220318	
TLX1NB	0.001220318	
ZNF154	0.001220318	
TM4SF20	0.001220318	
GLRA2	0.001220318	
SLC4A10	0.001220318	
FAM86B2	0.001220318	
RP11-19D2.1	0.001220318	
WDR78	0.001220318	
KCNE1	0.001220318	
RP11-568K15.1	0.001220318	
MLK4	0.001220318	
KLHDC8A	0.001220318	
EYA4	0.001220318	
PIGZ	0.001220318	
CCZ1B	0.001220318	
NUDT10	0.001220318	
ZNF738	0.001220318	

TMEM191B	0.001220318	
LYG1	0.001220318	
ACTL10	0.001220318	
P2RY8	0.001220318	
RTEL1-TNFRSF6B	0.001220318	
IL21R	0.001220318	
ABCA13	0.001220318	
ANO5	0.001220318	
LCTL	0.001220318	
ZFYVE28	0.001220318	
TMEM240	0.001220318	
SLAMF9	0.001220318	
ADCY10P1	0.001220318	
EDN3	0.001220318	
PARD6G-AS1	0.001220318	
LRRC37A	0.001220318	
PCDHGB7	0.001220318	
PRH1	0.001220318	
SSX1	0.001220318	
WDR88	0.001220318	
RP11-1275H24.1	0.001220318	
ZNF404	0.001220318	
DLX6	0.001220318	
GNRH2	0.001220318	
IQCH-AS1	0.001220318	
RNF165	0.001220318	
TMEM27	0.001220318	
GEMIN8P4	0.001220318	
CLUL1	0.001220318	
KANK4	0.001220318	
CHRM5	0.001220318	
TAMM41	0.001220318	
SNX32	0.001220318	
MTNR1B	0.001220318	
ARHGDIG	0.001220318	
AHSP	0.001220318	
LCE2A	0.001220318	
RRP7B	0.001220318	
ANKRD53	0.001220318	
FBXO39	0.001220318	
COBL	0.001220318	

CMAHP	0.001220318	
DNAJC9-AS1	0.001220318	
GPAT2	0.001220318	
BEX1	0.001220318	
SLFN5	0.001220318	
HCRTR2	0.001220318	
RLN2	0.001220318	
CDH10	0.001220318	
PGLYRP3	0.001220318	
PCDHGA8	0.001220318	
P2RX6	0.001220318	
KLHL11	0.001220318	
C2orf40	0.001220318	
AFF2	0.001220318	
SOX14	0.001220318	
MYCBPAP	0.001220318	
SMCP	0.001220318	
MMP24-AS1	0.001220318	
TMEM261	0.001220318	
SLITRK5	0.001220318	
NAPSA	0.001220318	
SNORD6	0.001220318	
CA10	0.001220318	
ATL1	0.001220318	
NBPF11	0.001220318	
OR2A7	0.001220318	
SGK494	0.001220318	
CFL1P1	0.001220318	
FBXO43	0.001220318	
TSKS	0.001220318	
TRNT1	0.001220318	
PTOV1-AS1	0.001220318	
VSIG2	0.001220318	
CCDC171	0.001220318	
NSUN5P2	0.001220318	
SLC8A2	0.001220318	
COMP	0.001220318	
C17orf102	0.001220318	
EFCAB6	0.001220318	
LINC00476	0.001220318	
B4GALT6	0.001220318	

SPOCK1	0.001220318	
MAB21L1	0.001220318	
ZNF32-AS2	0.001220318	
CRYM-AS1	0.001220318	
DNAJB3	0.001220318	
C10orf25	0.001220318	
FAM155A	0.001220318	
HRH4	0.001220318	
C2orf48	0.001220318	
RP11-644F5.11	0.001220318	
SLC25A43	0.001220318	
C2CD4C	0.001220318	
RGL4	0.001220318	
C10orf90	0.001220318	
NYAP1	0.001220318	
SLC17A5	0.001220318	
PLEK2	0.001220318	
POU4F2	0.001220318	
AC009948.5	0.001220318	
SCARNA10	0.001220318	
CRYGS	0.001220318	
GPR37L1	0.001220318	
RP11-696N14.1	0.001220318	
MAP7D3	0.001220318	
KRT8P41	0.001220318	
ZNF677	0.001220318	
EGOT	0.001220318	
RP11-259K5.2	0.001220318	
HAS3	0.001220318	
MATN4	0.001220318	
NSMF	0.001220318	
WNT9B	0.001220318	
ZNF815P	0.001220318	
GOLGA8A	0.001220318	
MAGEB4	0.001220318	
CAPN8	0.001220318	
GPR17	0.001220318	
PCDH8	0.001220318	
ZNF529-AS1	0.001220318	
KIAA0825	0.001220318	
CTD-3080P12.3	0.001220318	

RP11-334C17.5	0.001220318	
NBPF10	0.001220318	
SLC28A2	0.001220318	
MMP23B	0.001220318	
RGAG4	0.001220318	
RARRES1	0.001220318	
ALPI	0.001220318	
SDHAP3	0.001220318	
KAZALD1	0.001220318	
C1QTNF6	0.001220318	
CHRFAM7A	0.001220318	
RGPD5	0.001220318	
MIR4489	0.001220318	
EPHA6	0.001220318	
DNM3OS	0.001220318	
SLC6A1-AS1	0.001220318	
PNCK	0.001220318	
BOLA3-AS1	0.001220318	
SPATA12	0.001220318	
LIN28A	0.001220318	
PCDHGA11	0.001220318	
ADAMTS14	0.001220318	
CCDC89	0.001169472	
C1QTNF9	0.001169472	
ZNF594	0.001169472	
MC2R	0.001169472	
FAM131B	0.001169472	
CALB2	0.001169472	
SLC26A3	0.001169472	
C6orf52	0.001169472	
LINC00115	0.001169472	
INSRR	0.001169472	
FAM229A	0.001169472	
LHFPL5	0.001169472	
MCOLN2	0.001169472	
AMY1B	0.001169472	
NCF1C	0.001169472	
DLX6-AS1	0.001169472	
C6orf141	0.001169472	
SLIT1	0.001169472	
PAN3-AS1	0.001169472	

POPDC2	0.001169472	
AMY2A	0.001169472	
SLC12A3	0.001169472	
AQP7	0.001169472	
ENO1-AS1	0.001169472	
FAHD2B	0.001169472	
TBX10	0.001169472	
ZNF311	0.001169472	
SAMD15	0.001169472	
APITD1-CORT	0.001169472	
TMEM243	0.001169472	
TNFRSF13B	0.001169472	
GPR52	0.001169472	
BTBD8	0.001169472	
KRT86	0.001169472	
STAG3L1	0.001169472	
C15orf27	0.001169472	
OSGIN2	0.001169472	
MFAP5	0.001169472	
A2MP1	0.001169472	
OLFML1	0.001169472	
FAM129C	0.001169472	
HCG27	0.001169472	
RP11-864I4.1	0.001169472	
LRRC66	0.001169472	
SRSF12	0.001169472	
SPACA5	0.001169472	
ISX	0.001169472	
SOX1	0.001169472	
RP11-890B15.3	0.001169472	
POU6F2-AS1	0.001169472	
HHAT	0.001169472	
DBIL5P	0.001169472	
ZNF90	0.001169472	
CACNA2D4	0.001169472	
ABCA11P	0.001169472	
CBY3	0.001169472	
GPR75-ASB3	0.001169472	
GCSAM	0.001169472	
SLC22A18AS	0.001169472	
ITLN2	0.001169472	

ZDHHC22	0.001169472	
SLC2A12	0.001169472	
FAM222A-AS1	0.001169472	
ANP32AP1	0.001169472	
PLD5	0.001169472	
IRX6	0.001169472	
ULBP3	0.001169472	
FGF17	0.001169472	
CTD-2540F13.2	0.001169472	
APOL5	0.001169472	
CNTD2	0.001169472	
NKX2-8	0.001169472	
PSMD5-AS1	0.001169472	
PI4KAP1	0.001169472	
TRAT1	0.001169472	
CEACAM6	0.001169472	
PLA2G4B	0.001169472	
C1QTNF2	0.001169472	
CACNG4	0.001169472	
SUCNR1	0.001169472	
CHRM4	0.001169472	
RP11-392P7.6	0.001169472	
ZNF573	0.001169472	
MIR4257	0.001169472	
LCE3C	0.001169472	
TMPRSS4	0.001169472	
FBXW10	0.001169472	
SPOCD1	0.001169472	
OSCP1	0.001169472	
CASS4	0.001169472	
SDR42E1	0.001169472	
ZNF833P	0.001169472	
B3GALTL	0.001169472	
TNMD	0.001169472	
DTWD1	0.001169472	
ST8SIA6	0.001169472	
RSPH1	0.001169472	
DHRS9	0.001169472	
ATP2A1	0.001169472	
C19orf33	0.001169472	
MAMDC2	0.001169472	

ASB12	0.001169472	
ABHD17C	0.001169472	
ISCA1	0.001169472	
LRRTM1	0.001169472	
C1QL3	0.001169472	
NACAD	0.001169472	
FAM186A	0.001169472	
ATP8B5P	0.001169472	
DPYSL4	0.001169472	
SYNJ2BP-COX16	0.001169472	
DPY19L2P2	0.001169472	
MTRNR2L8	0.001169472	
CUZD1	0.001169472	
FSCN3	0.001169472	
RP3-414A15.12	0.001169472	
KCNC2	0.001169472	
KB-1732A1.1	0.001169472	
PCDHGA10	0.001169472	
DNAH17	0.001169472	
KRTAP12-4	0.001169472	
TMEM107	0.001169472	
TIMM21	0.001169472	
SLFN12	0.001169472	
MIR3677	0.001169472	
FITM2	0.001169472	
PA2G4P4	0.001169472	
TRPM1	0.001169472	
DIRAS2	0.001169472	
FAM186B	0.001169472	
MYOT	0.001169472	
FAM69A	0.001169472	
USH1G	0.001169472	
QRFP	0.001169472	
TBR1	0.001169472	
DNAJB7	0.001169472	
TTC26	0.001169472	
DPEP1	0.001169472	
KLRAP1	0.001169472	
C3orf33	0.001169472	
CCL28	0.001169472	
ZIM2	0.001169472	

AGRP	0.001169472	
TECTA	0.001169472	
GPRC5D	0.001169472	
LACE1	0.001169472	
GIPR	0.001169472	
ALPK2	0.001169472	
SYT14	0.001169472	
FABP5P3	0.001169472	
SPHK2	0.001169472	
FCGR1C	0.001169472	
LY6K	0.001169472	
KCNK12	0.001169472	
MIR181A2HG	0.001169472	
TMOD2	0.001169472	
ZNF284	0.001169472	
BMPER	0.001169472	
TMEM59L	0.001169472	
KCNK17	0.001169472	
SMC1B	0.001169472	
DPYSL5	0.001169472	
MIR4692	0.001169472	
ATP6V1G2-DDX39B	0.001169472	
ARHGAP36	0.001169472	
LAMTOR5-AS1	0.001169472	
SYCE2	0.001118625	
SLC25A25-AS1	0.001118625	
FUT2	0.001118625	
C11orf91	0.001118625	
ZNF257	0.001118625	
PIPSL	0.001118625	
OARD1	0.001118625	
SNORA74A	0.001118625	
MOV10L1	0.001118625	
OR10J6P	0.001118625	
CHRDL1	0.001118625	
ACER2	0.001118625	
DYNLRB2	0.001118625	
MICALCL	0.001118625	
ZNF837	0.001118625	
CLVS1	0.001118625	
SPAG6	0.001118625	

LRRC69	0.001118625	
IGHE	0.001118625	
FCGR1B	0.001118625	
ZNF597	0.001118625	
SNORD117	0.001118625	
FRRS1L	0.001118625	
PKD1L1	0.001118625	
MSMB	0.001118625	
SLC5A4	0.001118625	
KDM5D	0.001118625	
SCARA5	0.001118625	
ERVMER34-1	0.001118625	
SEZ6	0.001118625	
ZPBP	0.001118625	
ZNF141	0.001118625	
ZNF418	0.001118625	
ABHD14A-ACY1	0.001118625	
GPR75	0.001118625	
CLCA2	0.001118625	
C9orf173	0.001118625	
CTRC	0.001118625	
AC068535.3	0.001118625	
GJA3	0.001118625	
SLC6A11	0.001118625	
CREG2	0.001118625	
MIR4322	0.001118625	
SCIN	0.001118625	
SRPK3	0.001118625	
POC1B-GALNT4	0.001118625	
SCEL	0.001118625	
MIR129-2	0.001118625	
C7orf61	0.001118625	
PNOC	0.001118625	
CCNO	0.001118625	
ARHGAP28	0.001118625	
CNTN5	0.001118625	
CLMP	0.001118625	
SH2D5	0.001118625	
KCNK10	0.001118625	
TM7SF3	0.001118625	
RP11-632C17__A.1	0.001118625	

MIR4666A	0.001118625	
C1orf100	0.001118625	
ZNF442	0.001118625	
C2orf88	0.001118625	
NPHS2	0.001118625	
TRAPPC5	0.001118625	
PODNL1	0.001118625	
SLC47A2	0.001118625	
ZNF235	0.001118625	
NHLRC4	0.001118625	
TCP11	0.001118625	
PLA2G4C	0.001118625	
IFT22	0.001118625	
TACR1	0.001118625	
CHP2	0.001118625	
MMP8	0.001118625	
ALLC	0.001118625	
MCOLN3	0.001118625	
RP11-467L13.5	0.001118625	
C1orf116	0.001118625	
ODAM	0.001118625	
LRRC19	0.001118625	
BSND	0.001118625	
PEX5L	0.001118625	
SNORA71C	0.001118625	
ZEB1-AS1	0.001118625	
TIGD1	0.001118625	
MIR5010	0.001118625	
NRN1L	0.001118625	
RGS11	0.001118625	
LYPD6	0.001118625	
VIPR2	0.001118625	
AMZ2P1	0.001118625	
LINC00240	0.001118625	
SYT8	0.001118625	
DCSTAMP	0.001118625	
MS4A12	0.001118625	
XIRP1	0.001118625	
RPL21P28	0.001118625	
ATAT1	0.001118625	
PAX8-AS1	0.001118625	

ADAMTS9-AS2	0.001118625	
KLHL25	0.001118625	
ZNF714	0.001118625	
PROL1	0.001118625	
DNER	0.001118625	
GABRG2	0.001118625	
SLC45A1	0.001118625	
SLC7A10	0.001118625	
AC062029.1	0.001118625	
TTLL6	0.001118625	
NPM1P25	0.001118625	
GDPD2	0.001118625	
IQSEC3	0.001118625	
BSN-AS2	0.001118625	
RP11-356J5.12	0.001118625	
CDH7	0.001118625	
CDKL1	0.001118625	
FGF23	0.001118625	
FAM66C	0.001118625	
MUSTN1	0.001118625	
SLC26A4	0.001118625	
PCDHGA5	0.001118625	
HSP90AB3P	0.001118625	
LPHN3	0.001118625	
GLIPR1	0.001118625	
ADCYAP1R1	0.001118625	
MIR200C	0.001118625	
TNFAIP8L3	0.001118625	
DCST1	0.001118625	
ALOX12B	0.001118625	
CA6	0.001118625	
GAL3ST3	0.001118625	
TAF1L	0.001118625	
PNLDC1	0.001118625	
LINC00471	0.001118625	
CXCL17	0.001118625	
LINC00265	0.001118625	
GLDN	0.001118625	
CDH19	0.001118625	
RP11-434H6.6	0.001118625	
KCNMB3	0.001118625	

KRT83	0.001118625	
C1QL4	0.001118625	
TAAR3	0.001118625	
KCNK13	0.001118625	
SPIN2B	0.001118625	
C5orf56	0.001118625	
SGCZ	0.001118625	
GP5	0.001118625	
HTR7	0.001118625	
CTD-2366F13.1	0.001118625	
FSIP1	0.001118625	
SYT4	0.001118625	
CECR6	0.001118625	
DSG4	0.001118625	
LINC00680	0.001118625	
LINC00578	0.001118625	
FAHD2CP	0.001118625	
PTENP1	0.001118625	
ITPRIPL1	0.001118625	
MIR548AA1	0.001118625	
OLFM4	0.001118625	
RAB39B	0.001118625	
OASL	0.001118625	
CEACAM3	0.001118625	
C5orf38	0.001118625	
ANKRD36	0.001118625	
TMEM106A	0.001118625	
KCNJ14	0.001118625	
RP11-617F23.1	0.001118625	
PMS2P4	0.001118625	
SNORD83A	0.001118625	
DCLK3	0.001118625	
KCNG1	0.001118625	
SPATA8	0.001118625	
GRAMD2	0.001118625	
SVIL-AS1	0.001118625	
RGPD8	0.001118625	
C4orf36	0.001118625	
KCNG3	0.001118625	
CPSF4L	0.001118625	
GREM2	0.001067779	

FUNDC2P2	0.001067779	
PDPN	0.001067779	
CHRNG	0.001067779	
MIR218-2	0.001067779	
CCL26	0.001067779	
AQP8	0.001067779	
CCDC26	0.001067779	
RFX8	0.001067779	
SNAI3	0.001067779	
ACTBL2	0.001067779	
ADAT3	0.001067779	
KY	0.001067779	
IGLV3-9	0.001067779	
LINC00847	0.001067779	
PRR26	0.001067779	
ATP8A2	0.001067779	
C15orf26	0.001067779	
ZPBP2	0.001067779	
BNC2	0.001067779	
ZNF556	0.001067779	
P2RY12	0.001067779	
MIR1539	0.001067779	
ZNF229	0.001067779	
TRIM39-RPP21	0.001067779	
IGLV8-61	0.001067779	
ZSCAN4	0.001067779	
LINC00426	0.001067779	
FAM101A	0.001067779	
C11orf65	0.001067779	
HSPA7	0.001067779	
TNFSF8	0.001067779	
RAB42	0.001067779	
PKD1L2	0.001067779	
ANKRD45	0.001067779	
COL22A1	0.001067779	
RNU4ATAC	0.001067779	
PTPRVP	0.001067779	
RP11-1220K2.2	0.001067779	
PKP1	0.001067779	
NME5	0.001067779	
SNORA70	0.001067779	

PI16	0.001067779	
FAM221A	0.001067779	
CCDC13	0.001067779	
SNORA53	0.001067779	
PCDHGB3	0.001067779	
NIPSNAP3B	0.001067779	
PLA2G3	0.001067779	
TMEM182	0.001067779	
COL21A1	0.001067779	
TMLHE	0.001067779	
MIR23A	0.001067779	
CES5A	0.001067779	
OR2A9P	0.001067779	
SIX6	0.001067779	
TP53AIP1	0.001067779	
PSPHP1	0.001067779	
SNORD62B	0.001067779	
GALR2	0.001067779	
PTRH1	0.001067779	
GMDS-AS1	0.001067779	
RP11-5C23.1	0.001067779	
MTL5	0.001067779	
ZNF487	0.001067779	
MIRLET7I	0.001067779	
HIGD1B	0.001067779	
FREM1	0.001067779	
NTSR1	0.001067779	
WDR17	0.001067779	
ZMAT4	0.001067779	
CFAP69	0.001067779	
FMR1-AS1	0.001067779	
SNORA74B	0.001067779	
HIF1A-AS2	0.001067779	
CTRL	0.001067779	
TACR3	0.001067779	
RTKN2	0.001067779	
HEBP1	0.001067779	
PCAT6	0.001067779	
CRYBA4	0.001067779	
C12orf42	0.001067779	
ESYT3	0.001067779	

ARPIN	0.001067779	
FGF10	0.001067779	
SLC22A14	0.001067779	
VWA2	0.001067779	
BMS1P4	0.001067779	
PPEF1	0.001067779	
DNAAF1	0.001067779	
CACNA1S	0.001067779	
AKR7L	0.001067779	
SMPDL3B	0.001067779	
MUSK	0.001067779	
KLF14	0.001067779	
PGC	0.001067779	
ZNF586	0.001067779	
FAM196A	0.001067779	
AGO1	0.001067779	
PCDHB5	0.001067779	
KTN1-AS1	0.001067779	
FAM19A2	0.001067779	
KLHL10	0.001067779	
ALOX12P2	0.001067779	
RP11-774O3.3	0.001067779	
DZANK1	0.001067779	
KLHL14	0.001067779	
PTPLA	0.001067779	
LRRIQ3	0.001067779	
FAM19A4	0.001067779	
SLC26A11	0.001067779	
CTXN1	0.001067779	
C1orf177	0.001067779	
POLR2J3	0.001067779	
CTSE	0.001067779	
FSCN2	0.001067779	
CMTM1	0.001067779	
MS4A2	0.001067779	
CDSN	0.001067779	
OR1J1	0.001067779	
ARHGEF26-AS1	0.001067779	
SEMA3D	0.001067779	
MCF2L2	0.001067779	
FAM86B1	0.001067779	

KIAA1755	0.001067779	
FAM166A	0.001067779	
SLC24A4	0.001067779	
UCP1	0.001067779	
RP4-769N13.6	0.001067779	
CLUHP3	0.001067779	
NAALAD2	0.001067779	
MB	0.001067779	
LGALS9C	0.001067779	
ZNF385C	0.001067779	
SLC5A7	0.001067779	
ZNF717	0.001067779	
PRSS12	0.001067779	
ZNF699	0.001067779	
TRIM71	0.001067779	
CD160	0.001067779	
CRLF2	0.001067779	
DCAF13P3	0.001067779	
FAM43A	0.001067779	
PGK2	0.001067779	
MIR4658	0.001067779	
ARRDC5	0.001067779	
MAP1LC3B2	0.001067779	
ZNF570	0.001067779	
PMFBP1	0.001067779	
LRFN2	0.001067779	
TRIL	0.001067779	
NEUROD2	0.001067779	
SH2D4B	0.001067779	
DDX43	0.001067779	
MED12L	0.001067779	
SNX15	0.001067779	
TMEM251	0.001067779	
ABO	0.001067779	
NCCRP1	0.001067779	
EVX2	0.001067779	
LOXHD1	0.001067779	
ST3GAL6-AS1	0.001067779	
SCGB1B2P	0.001067779	
GPC5	0.001067779	
PSCA	0.001067779	

RNASE13	0.001067779	
CCDC162P	0.001067779	
HAS1	0.001067779	
PLA2G4D	0.001067779	
PRSS30P	0.001067779	
CELF5	0.001067779	
LRFN5	0.001067779	
DUX4	0.001067779	
RP11-575F12.3	0.001067779	
FLJ37035	0.001067779	
PM20D2	0.001067779	
RASSF6	0.001067779	
SYCE3	0.001067779	
AIM2	0.001067779	
GABRA3	0.001067779	
GABRG1	0.001067779	
MFI2-AS1	0.001067779	
GPR3	0.001067779	
CPA4	0.001067779	
SCGB2B2	0.001067779	
PRRT4	0.001016932	
HAS2-AS1	0.001016932	
TRMT10A	0.001016932	
RIMKLA	0.001016932	
PABPC4L	0.001016932	
CCNB3	0.001016932	
ZNF501	0.001016932	
CLRN1-AS1	0.001016932	
HMGB1P5	0.001016932	
NPAS1	0.001016932	
ZRANB2-AS2	0.001016932	
CCDC39	0.001016932	
PRH2	0.001016932	
GPD1L	0.001016932	
FAM163B	0.001016932	
WDR92	0.001016932	
MIR106B	0.001016932	
NICN1	0.001016932	
SLC6A19	0.001016932	
COX6A1P2	0.001016932	
CMTM2	0.001016932	

CNIH2	0.001016932	
DKKL1	0.001016932	
GBP1P1	0.001016932	
C1orf189	0.001016932	
MIR4665	0.001016932	
TRIM58	0.001016932	
KRT72	0.001016932	
PRLHR	0.001016932	
SLC12A1	0.001016932	
RANBP17	0.001016932	
SLC25A48	0.001016932	
MIR4783	0.001016932	
SRMS	0.001016932	
CEMIP	0.001016932	
CLCNKB	0.001016932	
DIO3	0.001016932	
ZNF578	0.001016932	
FGF16	0.001016932	
SLC23A3	0.001016932	
GHRLOS	0.001016932	
SYT9	0.001016932	
ISLR2	0.001016932	
ZNF169	0.001016932	
FGF22	0.001016932	
GPR84	0.001016932	
RNU12	0.001016932	
MIR3189	0.001016932	
IFITM10	0.001016932	
REG1A	0.001016932	
GPR20	0.001016932	
KLHL31	0.001016932	
MIR193A	0.001016932	
KLHL30	0.001016932	
C1orf111	0.001016932	
FAM160A1	0.001016932	
SLN	0.001016932	
MYBPC1	0.001016932	
CALR3	0.001016932	
PDZD9	0.001016932	
FBXL2	0.001016932	
SLC26A10	0.001016932	

SLC12A5	0.001016932	
EIF1AY	0.001016932	
MIA	0.001016932	
SOX21	0.001016932	
EEF1DP3	0.001016932	
AOC1	0.001016932	
FPGT-TNNI3K	0.001016932	
ZDBF2	0.001016932	
DOC2A	0.001016932	
MIR3649	0.001016932	
SERPINB3	0.001016932	
C16orf93	0.001016932	
TSPO2	0.001016932	
SENP3-EIF4A1	0.001016932	
LHFPL1	0.001016932	
ACBD7	0.001016932	
C10orf111	0.001016932	
EYS	0.001016932	
CPB1	0.001016932	
GOLGA8F	0.001016932	
CBLN2	0.001016932	
SOST	0.001016932	
DNAAF3	0.001016932	
TRIM17	0.001016932	
BPIFB4	0.001016932	
RAB4B-EGLN2	0.001016932	
POM121L8P	0.001016932	
STK33	0.001016932	
CRISPLD1	0.001016932	
RP11-421L21.3	0.001016932	
SLC9A7P1	0.001016932	
C10orf95	0.001016932	
FAM45B	0.001016932	
SPATA17	0.001016932	
GUCD1	0.001016932	
KLK4	0.001016932	
RHCE	0.001016932	
P2RY10	0.001016932	
SHC4	0.001016932	
SLC16A12	0.001016932	
RPA4	0.001016932	

SPATA21	0.001016932	
TNNT2	0.001016932	
EPHA10	0.001016932	
ATP5J2-PTCD1	0.001016932	
KIF27	0.001016932	
OVOL2	0.001016932	
PDSS1	0.001016932	
CRABP1	0.001016932	
S1PR3	0.001016932	
SLC30A8	0.001016932	
ZAR1L	0.001016932	
ZNF324B	0.001016932	
RBM20	0.001016932	
RP11-129M16.4	0.001016932	
PABPC5	0.001016932	
BAI3	0.001016932	
IGKV1-27	0.001016932	
TRPC7	0.001016932	
CDH8	0.001016932	
PRR4	0.001016932	
GPR179	0.001016932	
XKRX	0.001016932	
BTNL8	0.001016932	
FANK1	0.001016932	
MIR659	0.001016932	
CCDC96	0.001016932	
LPO	0.001016932	
CFLAR-AS1	0.001016932	
UGT3A2	0.001016932	
SERF1B	0.001016932	
CFAP221	0.001016932	
ACTL8	0.001016932	
ZNF519	0.001016932	
PLA2G4F	0.001016932	
WDR41	0.001016932	
ARMC4	0.001016932	
UPK1A-AS1	0.001016932	
NEUROG2	0.001016932	
UTY	0.001016932	
TTC34	0.001016932	
UTS2R	0.001016932	

NMS	0.001016932	
HERC2P2	0.001016932	
RAD21-AS1	0.001016932	
ENO4	0.001016932	
RP11-320N7.2	0.001016932	
C6orf25	0.001016932	
RMST	0.001016932	
EIF3FP3	0.001016932	
OVOL1	0.001016932	
FAXC	0.001016932	
C1QTNF9B-AS1	0.001016932	
ATP6V0D2	0.001016932	
OR51E2	0.001016932	
SLC7A5P1	0.001016932	
SHISA6	0.001016932	
SLC26A9	0.001016932	
NLRP11	0.001016932	
DTD2	0.001016932	
LY6G5B	0.001016932	
CDKN2B-AS1	0.001016932	
TRIM34	0.001016932	
MIR641	0.001016932	
CCDC79	0.000966085	
DUOXA1	0.000966085	
DLEC1	0.000966085	
INE1	0.000966085	
LRRTM4	0.000966085	
NTF4	0.000966085	
KIAA1045	0.000966085	
CCDC42	0.000966085	
FCRL6	0.000966085	
NEK10	0.000966085	
DENND5B-AS1	0.000966085	
MIR3186	0.000966085	
SPATA6L	0.000966085	
ZNF763	0.000966085	
VSIG10	0.000966085	
PCDHGB8P	0.000966085	
C9orf171	0.000966085	
ZNF286B	0.000966085	
GJC2	0.000966085	

C3orf36	0.000966085	
WI2-1896O14.1	0.000966085	
EPHA8	0.000966085	
GABRQ	0.000966085	
SGSM1	0.000966085	
RNF138P1	0.000966085	
DKFZP434A062	0.000966085	
COL11A1	0.000966085	
FER1L4	0.000966085	
SHISA2	0.000966085	
CAPN9	0.000966085	
FFAR2	0.000966085	
SNORA67	0.000966085	
TTC16	0.000966085	
KRTAP12-1	0.000966085	
TMEM221	0.000966085	
SYNDIG1	0.000966085	
U3	0.000966085	
SCN3A	0.000966085	
CACNA1I	0.000966085	
KLK13	0.000966085	
URB1-AS1	0.000966085	
CYP2S1	0.000966085	
ZRANB2-AS1	0.000966085	
AKAP2	0.000966085	
TRHDE	0.000966085	
IGSF9B	0.000966085	
VGLL2	0.000966085	
SNORD37	0.000966085	
CTD-2517M22.14	0.000966085	
TEX9	0.000966085	
BAALC	0.000966085	
CHRNA7	0.000966085	
SOGA3	0.000966085	
LGI1	0.000966085	
DRP2	0.000966085	
ALPK1	0.000966085	
TSPAN32	0.000966085	
DKK4	0.000966085	
LANCL2	0.000966085	
GABRA2	0.000966085	

NTN5	0.000966085	
LGALS9B	0.000966085	
ZBTB20-AS1	0.000966085	
DNAJC27-AS1	0.000966085	
SCRT1	0.000966085	
C3orf80	0.000966085	
KCNA7	0.000966085	
MEIS1-AS3	0.000966085	
UNC80	0.000966085	
MGARP	0.000966085	
SNORD77	0.000966085	
PCDHA8	0.000966085	
KCNH6	0.000966085	
KCP	0.000966085	
FLJ26850	0.000966085	
KCNV2	0.000966085	
CCDC62	0.000966085	
RPS6KL1	0.000966085	
FOXN1	0.000966085	
DDX11L2	0.000966085	
KRTAP19-5	0.000966085	
SNORA55	0.000966085	
GLRA1	0.000966085	
HPSE2	0.000966085	
SLC35F1	0.000966085	
SYNDIG1L	0.000966085	
P2RY4	0.000966085	
IL17RD	0.000966085	
WBP11P1	0.000966085	
PRSS27	0.000966085	
NRADDP	0.000966085	
CSPG5	0.000966085	
GAREM	0.000966085	
MIR126	0.000966085	
DSPP	0.000966085	
CTAGE4	0.000966085	
MTMR8	0.000966085	
KRTAP5-7	0.000966085	
MYLK3	0.000966085	
RXFP2	0.000966085	
C6orf163	0.000966085	

CCDC85A	0.000966085	
SV2C	0.000966085	
ZNF93	0.000966085	
COL4A2-AS1	0.000966085	
LRRC55	0.000966085	
CCDC141	0.000966085	
C17orf104	0.000966085	
OPN1SW	0.000966085	
CXorf66	0.000966085	
C11orf86	0.000966085	
HIGD1AP1	0.000966085	
FPR2	0.000966085	
SNORD99	0.000966085	
H3F3C	0.000966085	
DPY19L2P3	0.000966085	
DRD5	0.000966085	
ICA1L	0.000966085	
FNDC8	0.000966085	
FGF14	0.000966085	
FAM151B	0.000966085	
B4GALNT2	0.000966085	
SRP9P1	0.000966085	
TECTB	0.000966085	
GXYLT2	0.000966085	
IGDCC3	0.000966085	
S100Z	0.000966085	
CBWD3	0.000966085	
PCDHA7	0.000966085	
HCG11	0.000966085	
WBP2NL	0.000966085	
BCL2L14	0.000966085	
CLEC5A	0.000966085	
ANXA8	0.000966085	
SLITRK1	0.000966085	
RLBP1	0.000966085	
XXbac-BPG181B23.7	0.000966085	
SERHL	0.000966085	
CXorf21	0.000966085	
KRTAP4-1	0.000966085	
SCGB3A2	0.000966085	
REEP2	0.000966085	

LGALS12	0.000966085	
STK32B	0.000966085	
TBX1	0.000966085	
GOLGA6L9	0.000966085	
DPP10	0.000966085	
FLJ13224	0.000966085	
PYDC1	0.000966085	
GP9	0.000966085	
SRRM4	0.000966085	
SPATA9	0.000966085	
NT5C3A	0.000966085	
FOXD4L1	0.000966085	
SLITRK2	0.000966085	
HCRTR1	0.000966085	
CRB1	0.000966085	
PALM2-AKAP2	0.000966085	
ALDH1L2	0.000966085	
IL19	0.000966085	
RAD51AP2	0.000966085	
ZCCHC12	0.000966085	
UCKL1-AS1	0.000966085	
SMPX	0.000966085	
STRA6	0.000966085	
ST6GALNAC5	0.000966085	
MEPE	0.000966085	
ZFP69	0.000966085	
RP11-9E17.1	0.000966085	
CRB2	0.000966085	
ABHD11-AS1	0.000966085	
PVRIG	0.000966085	
HIST1H2AA	0.000966085	
PFN1P2	0.000966085	
SLC9B1	0.000966085	
SNORD81	0.000966085	
GKN2	0.000966085	
TAS1R2	0.000966085	
TCEAL7	0.000966085	
RRH	0.000966085	
CHIA	0.000966085	
ZNF781	0.000966085	
OR2B6	0.000966085	

FAM43B	0.000966085	
TMEM236	0.000966085	
SPTBN5	0.000966085	
SYT10	0.000966085	
CD1B	0.000966085	
MIR3653	0.000966085	
C10orf105	0.000966085	
HCG4B	0.000966085	
WDR49	0.000966085	
OTOF	0.000966085	
RPL3L	0.000915239	
DNAJC5B	0.000915239	
GPR182	0.000915239	
SNORA70F	0.000915239	
TPTEP1	0.000915239	
ANKRD36B	0.000915239	
PNMT	0.000915239	
ERV3-1	0.000915239	
PANX2	0.000915239	
MIR550A1	0.000915239	
PVALB	0.000915239	
MIMT1	0.000915239	
PCDHB6	0.000915239	
TMEM31	0.000915239	
ART3	0.000915239	
PIK3R6	0.000915239	
SCRG1	0.000915239	
TMEM40	0.000915239	
CABP2	0.000915239	
ZNF831	0.000915239	
DISP2	0.000915239	
ANO4	0.000915239	
MIR4442	0.000915239	
COL6A5	0.000915239	
IL17F	0.000915239	
BARHL2	0.000915239	
TDRD1	0.000915239	
IL9R	0.000915239	
TAS2R38	0.000915239	
WISP3	0.000915239	
MIR3671	0.000915239	

PGLYRP1	0.000915239	
SLITRK3	0.000915239	
ACOT6	0.000915239	
SGOL1-AS1	0.000915239	
ASCL4	0.000915239	
XAGE3	0.000915239	
ZNF749	0.000915239	
LAIR2	0.000915239	
FUT9	0.000915239	
LINC00441	0.000915239	
MIPEPP3	0.000915239	
XIST	0.000915239	
ARHGAP39	0.000915239	
PRMT8	0.000915239	
TIGD4	0.000915239	
BARX2	0.000915239	
PCDHA12	0.000915239	
DSCAM	0.000915239	
MIR202	0.000915239	
SCGB2A1	0.000915239	
C1QTNF4	0.000915239	
ZNF610	0.000915239	
EPHX4	0.000915239	
CSH2	0.000915239	
CSRNP3	0.000915239	
FBLL1	0.000915239	
TM4SF19	0.000915239	
RASL11B	0.000915239	
SP9	0.000915239	
RAB9B	0.000915239	
CD300LB	0.000915239	
MIR4513	0.000915239	
MIR130A	0.000915239	
TLR7	0.000915239	
GDPD4	0.000915239	
HRH2	0.000915239	
KRT39	0.000915239	
MIR933	0.000915239	
LY6G5C	0.000915239	
ZNF429	0.000915239	
PADI1	0.000915239	

MIR4263	0.000915239	
PTX4	0.000915239	
AFAP1-AS1	0.000915239	
MIR4645	0.000915239	
TMSB4XP1	0.000915239	
RGR	0.000915239	
RAB6C	0.000915239	
IRX4	0.000915239	
CNGA4	0.000915239	
LINC00487	0.000915239	
BNC1	0.000915239	
PROP1	0.000915239	
JAKMIP3	0.000915239	
RP1-239B22.5	0.000915239	
GADL1	0.000915239	
ANKMY1	0.000915239	
ADIG	0.000915239	
BMP8A	0.000915239	
PDZRN4	0.000915239	
LINC00238	0.000915239	
TMEM95	0.000915239	
EML5	0.000915239	
SNORD100	0.000915239	
UGT1A7	0.000915239	
DMBX1	0.000915239	
OLFM3	0.000915239	
CTCFL	0.000915239	
PAX4	0.000915239	
HLA-DPB2	0.000915239	
PRDM7	0.000915239	
TMEM125	0.000915239	
LINC00271	0.000915239	
BTN2A3P	0.000915239	
EVX1	0.000915239	
GALNTL6	0.000915239	
TEX22	0.000915239	
TRPM6	0.000915239	
ZNF252P-AS1	0.000915239	
LRRC39	0.000915239	
GSDMA	0.000915239	
SNORD2	0.000915239	

OTUD7A	0.000915239	
TCTE1	0.000915239	
ARL17A	0.000915239	
PPIEL	0.000915239	
LMOD3	0.000915239	
NAV2-AS5	0.000915239	
TMEM17	0.000915239	
FIGNL2	0.000915239	
SNORD66	0.000915239	
NHSL2	0.000915239	
HIST1H2BH	0.000915239	
SYCP2L	0.000915239	
KPNA7	0.000915239	
MTRNR2L1	0.000915239	
SULT4A1	0.000915239	
FHDC1	0.000915239	
MIR618	0.000915239	
MIR670	0.000915239	
TDRD6	0.000915239	
CST2	0.000915239	
DEPDC4	0.000915239	
DDX3Y	0.000915239	
MSANTD3-TMEFF1	0.000915239	
ZNF816-ZNF321P	0.000915239	
PNLIPRP2	0.000915239	
AGAP10	0.000915239	
KIR2DL3	0.000915239	
SLCO5A1	0.000915239	
BARHL1	0.000915239	
PLA2G2E	0.000915239	
CILP2	0.000915239	
GPR150	0.000915239	
AXDND1	0.000915239	
LINC00235	0.000915239	
MYEOV	0.000915239	
ZNF75A	0.000915239	
KCNH8	0.000915239	
MYH6	0.000915239	
C9orf117	0.000915239	
ZNF843	0.000915239	
GABRG3	0.000915239	

NELL1	0.000915239	
SPDYA	0.000915239	
DPY19L2	0.000915239	
GPR173	0.000915239	
MORF4	0.000915239	
SFTA1P	0.000915239	
SLC36A4	0.000915239	
SMTNL2	0.000915239	
KRT18P55	0.000915239	
NKAIN2	0.000915239	
AJAP1	0.000915239	
PCDHB9	0.000915239	
ALMS1P	0.000915239	
MLK7-AS1	0.000915239	
FBXL16	0.000915239	
CCDC144NL-AS1	0.000915239	
HTN1	0.000915239	
KRT12	0.000915239	
CAPZA3	0.000915239	
FAM201A	0.000915239	
CASP12	0.000915239	
GRIK4	0.000915239	
HRK	0.000915239	
RP11-830F9.5	0.000915239	
C9orf84	0.000915239	
FAIM2	0.000915239	
C1QTNF9B	0.000915239	
SLC6A7	0.000915239	
C14orf37	0.000915239	
ACRV1	0.000915239	
GTSF1	0.000915239	
HHATL	0.000915239	
CALB1	0.000915239	
CATSPERG	0.000915239	
PCDHA1	0.000915239	
SLC10A2	0.000915239	
PPM1E	0.000915239	
MMP16	0.000915239	
CDH4	0.000915239	
ANO7P1	0.000915239	
CRISP3	0.000915239	

TMEM191C	0.000915239	
SIGLEC6	0.000915239	
LPAR4	0.000915239	
ASS1P1	0.000915239	
IGF2-AS	0.000915239	
RDM1	0.000915239	
PCDH11X	0.000915239	
TMSB4Y	0.000915239	
LRGUK	0.000915239	
FSTL5	0.000915239	
KRTAP3-1	0.000915239	
TBL1Y	0.000915239	
PCP4	0.000915239	
BEAN1	0.000915239	
SYNPO2L	0.000864392	
RIIAD1	0.000864392	
RNF186	0.000864392	
KIAA1257	0.000864392	
TFDP3	0.000864392	
MPP4	0.000864392	
TMEM217	0.000864392	
ARMC12	0.000864392	
PGM5P2	0.000864392	
PDYN	0.000864392	
CCDC154	0.000864392	
SNORA38	0.000864392	
TMEM144	0.000864392	
ADAM21	0.000864392	
CELA2A	0.000864392	
AQPEP	0.000864392	
CYP46A1	0.000864392	
SSR4P1	0.000864392	
MIR191	0.000864392	
KIAA1024	0.000864392	
SCARNA5	0.000864392	
ZNF684	0.000864392	
YPEL4	0.000864392	
ULK4P2	0.000864392	
SLC22A6	0.000864392	
NOP14-AS1	0.000864392	
EXD1	0.000864392	

TBC1D27	0.000864392	
GTF2IP1	0.000864392	
HEMGN	0.000864392	
CCDC13-AS1	0.000864392	
CHRNA2	0.000864392	
CRYBB2P1	0.000864392	
GRM5-AS1	0.000864392	
BIRC8	0.000864392	
ZNF233	0.000864392	
CSNK1G2-AS1	0.000864392	
RP1L1	0.000864392	
TSPEAR	0.000864392	
IGSF11	0.000864392	
Z83851.4	0.000864392	
PRTG	0.000864392	
CLEC4A	0.000864392	
TTLL2	0.000864392	
GLIPR1L1	0.000864392	
ZFP91-CNTF	0.000864392	
CERS1	0.000864392	
ZNF625	0.000864392	
DKK2	0.000864392	
TVP23B	0.000864392	
PTCRA	0.000864392	
BPIFB1	0.000864392	
CPS1-IT1	0.000864392	
PCDHB12	0.000864392	
DCDC1	0.000864392	
CCAT2	0.000864392	
LRRC24	0.000864392	
PRORSD1P	0.000864392	
METTL24	0.000864392	
C22orf46	0.000864392	
GSTT2	0.000864392	
SAMD13	0.000864392	
HIST2H3D	0.000864392	
GPR174	0.000864392	
SPIC	0.000864392	
SIGLEC17P	0.000864392	
ZNF878	0.000864392	
KRT35	0.000864392	

TRPV5	0.000864392	
ZNF547	0.000864392	
DMRT1	0.000864392	
MIR125B1	0.000864392	
CCR9	0.000864392	
GPA33	0.000864392	
NKPD1	0.000864392	
SLC7A4	0.000864392	
C20orf202	0.000864392	
STEAP1B	0.000864392	
TMEM89	0.000864392	
MT3	0.000864392	
RP11-83A24.2	0.000864392	
MYLK2	0.000864392	
C20orf141	0.000864392	
FEZF2	0.000864392	
RHAG	0.000864392	
HSP90B3P	0.000864392	
ZNF540	0.000864392	
RNF182	0.000864392	
SNORD42B	0.000864392	
ZP2	0.000864392	
SNORA5C	0.000864392	
SRRM5	0.000864392	
CCDC110	0.000864392	
RNF175	0.000864392	
LINC00482	0.000864392	
HSD52	0.000864392	
LRRC15	0.000864392	
ATOH7	0.000864392	
SH3TC2	0.000864392	
CATSPER3	0.000864392	
AOX2P	0.000864392	
PKD2L2	0.000864392	
TMEM130	0.000864392	
MIR4479	0.000864392	
C8orf31	0.000864392	
C19orf18	0.000864392	
FAM153B	0.000864392	
RPL10L	0.000864392	
CCDC87	0.000864392	

PSG3	0.000864392	
C9orf106	0.000864392	
SLC5A2	0.000864392	
KCNH3	0.000864392	
MIR92B	0.000864392	
SNORA66	0.000864392	
KCNV1	0.000864392	
TEKT3	0.000864392	
TMEM178B	0.000864392	
SNORD7	0.000864392	
LINC00925	0.000864392	
CHADL	0.000864392	
ASPHD2	0.000864392	
KLHL1	0.000864392	
CCDC64B	0.000864392	
MUC16	0.000864392	
GUCA2A	0.000864392	
ANKFN1	0.000864392	
ART5	0.000864392	
GBX1	0.000864392	
CALY	0.000864392	
HTN3	0.000864392	
CYP2W1	0.000864392	
ZNF559-ZNF177	0.000864392	
C7orf57	0.000864392	
MIR186	0.000864392	
FKBP6	0.000864392	
C3orf52	0.000864392	
FAM228B	0.000864392	
FBXL21	0.000864392	
SLC7A13	0.000864392	
ST18	0.000864392	
C14orf180	0.000864392	
AC034220.3	0.000864392	
MRVI1-AS1	0.000864392	
ATP2C2	0.000864392	
PRH1-PRR4	0.000864392	
OR51B5	0.000864392	
MIR5008	0.000864392	
GABRA4	0.000864392	
C15orf43	0.000864392	

PPIL6	0.000864392	
ZDHHC15	0.000864392	
KRT2	0.000864392	
HIST1H2BO	0.000864392	
CYTL1	0.000864392	
PKD1P1	0.000864392	
PCDHA11	0.000864392	
TCF15	0.000864392	
WIF1	0.000864392	
LIPF	0.000864392	
NRSN1	0.000864392	
COLGALT2	0.000864392	
HOXD-AS2	0.000864392	
HHIPL2	0.000864392	
RGMB	0.000864392	
MIR3153	0.000864392	
ERVFRD-1	0.000864392	
SHISA8	0.000864392	
SOHLH2	0.000864392	
UCA1	0.000864392	
PCDHB2	0.000864392	
RAB1C	0.000864392	
LINC00523	0.000864392	
PCDHA10	0.000864392	
HYKK	0.000813546	
PDCL2	0.000813546	
NOXO1	0.000813546	
TRIM72	0.000813546	
HTR2C	0.000813546	
C2orf66	0.000813546	
HIST2H2BA	0.000813546	
GNAS-AS1	0.000813546	
LRRTM3	0.000813546	
GTSF1L	0.000813546	
OR2A20P	0.000813546	
ACOXL	0.000813546	
GYPE	0.000813546	
TMEM145	0.000813546	
PSG1	0.000813546	
TRANK1	0.000813546	
GSX2	0.000813546	

KRT6C	0.000813546	
LINC00086	0.000813546	
ZNF474	0.000813546	
WDR63	0.000813546	
EGFL6	0.000813546	
TMEM151B	0.000813546	
C6orf201	0.000813546	
SNAI3-AS1	0.000813546	
SNORD89	0.000813546	
EIF4BP6	0.000813546	
IL20RB	0.000813546	
IL20	0.000813546	
TMSB4XP2	0.000813546	
ZNF860	0.000813546	
FAM163A	0.000813546	
UNC5D	0.000813546	
ADTRP	0.000813546	
C11orf52	0.000813546	
LINC00602	0.000813546	
POU5F2	0.000813546	
PTTG2	0.000813546	
TMEFF2	0.000813546	
HRH3	0.000813546	
RPS2P32	0.000813546	
PSD2	0.000813546	
ZGLP1	0.000813546	
MIR125B2	0.000813546	
MYL4	0.000813546	
NXNL2	0.000813546	
SNTG2	0.000813546	
ADAMTS19	0.000813546	
FEZF1	0.000813546	
RASSF9	0.000813546	
XKR6	0.000813546	
TM9SF1	0.000813546	
CACNG2	0.000813546	
TSPAN2	0.000813546	
RP13-870H17.3	0.000813546	
FAM71C	0.000813546	
PLA2G4E	0.000813546	
RNFT2	0.000813546	

SNORD72	0.000813546	
HMX2	0.000813546	
NKX6-2	0.000813546	
SCARNA16	0.000813546	
CSMD3	0.000813546	
PLAC1	0.000813546	
TMEM155	0.000813546	
ZFP37	0.000813546	
GPR160	0.000813546	
CCDC157	0.000813546	
PCDHB1	0.000813546	
ZNF709	0.000813546	
SLFNL1	0.000813546	
ULK4P3	0.000813546	
LHFPL4	0.000813546	
GNG12-AS1	0.000813546	
TRIM40	0.000813546	
SLC39A2	0.000813546	
AC021218.2	0.000813546	
ZNF750	0.000813546	
C17orf105	0.000813546	
PBX4	0.000813546	
POU6F2	0.000813546	
PSORS1C1	0.000813546	
MC3R	0.000813546	
TFAP2E	0.000813546	
C9orf170	0.000813546	
MIR661	0.000813546	
CDH20	0.000813546	
WBSCR28	0.000813546	
GUSBP11	0.000813546	
HTR6	0.000813546	
DCDC2B	0.000813546	
GLB1L2	0.000813546	
RNU6-1	0.000813546	
C8orf86	0.000813546	
PFN3	0.000813546	
ABCB5	0.000813546	
C2orf71	0.000813546	
KCNH7	0.000813546	
IL21	0.000813546	

SNORA13	0.000813546	
LRRC6	0.000813546	
TMEM71	0.000813546	
IL31RA	0.000813546	
PRSS50	0.000813546	
C16orf96	0.000813546	
PCDHA5	0.000813546	
PROK2	0.000813546	
SYNGR4	0.000813546	
CCDC70	0.000813546	
GALNT13	0.000813546	
NR2E1	0.000813546	
AZU1	0.000813546	
IL36B	0.000813546	
LY86-AS1	0.000813546	
CASQ1	0.000813546	
PCDHA2	0.000813546	
MORC1	0.000813546	
ANO3	0.000813546	
P2RY14	0.000813546	
MIR615	0.000813546	
AKR1E2	0.000813546	
ABCB9	0.000813546	
ZSWIM5	0.000813546	
MLN	0.000813546	
GNAT2	0.000813546	
SERPINI2	0.000813546	
PIGB	0.000813546	
HERC2P4	0.000813546	
GJB6	0.000813546	
ZNF890P	0.000813546	
KIF6	0.000813546	
FLJ22447	0.000813546	
GSTM5	0.000813546	
LINC00535	0.000813546	
MIR4766	0.000813546	
FAM153A	0.000813546	
PCDH11Y	0.000813546	
HSFX1	0.000813546	
FOXB1	0.000813546	
GPR83	0.000813546	

SLC18A1	0.000813546	
GUSBP3	0.000813546	
KATNBL1	0.000813546	
HEPACAM2	0.000813546	
CSTL1	0.000813546	
MOBP	0.000813546	
MIR148A	0.000813546	
RXFP4	0.000813546	
WDR93	0.000813546	
CCIN	0.000813546	
OR51E1	0.000813546	
ZCCHC5	0.000813546	
RTEL1	0.000813546	
DPEP3	0.000813546	
MSL3P1	0.000813546	
PEBP4	0.000813546	
LEMD1	0.000813546	
CDHR1	0.000813546	
GRM4	0.000813546	
CNEP1R1	0.000813546	
CSNK1A1L	0.000813546	
FTMT	0.000813546	
ERICD	0.000813546	
ABCA17P	0.000813546	
ANKRD7	0.000813546	
ENPP5	0.000813546	
BEST3	0.000813546	
RASGEF1C	0.000813546	
TRBV2	0.000813546	
HAR1B	0.000813546	
XKR5	0.000813546	
ZNF215	0.000813546	
HMSD	0.000813546	
C5orf47	0.000813546	
C6orf15	0.000813546	
CNTNAP3	0.000813546	
TESPA1	0.000813546	
LY6G6C	0.000813546	
RAET1E	0.000813546	
LINC00310	0.000813546	
CHD5	0.000762699	

CLNK	0.000762699	
CST6	0.000762699	
DNAH9	0.000762699	
NME9	0.000762699	
SFTPA2	0.000762699	
OTX2-AS1	0.000762699	
KB-1460A1.5	0.000762699	
LINC00200	0.000762699	
DNAI2	0.000762699	
FAT3	0.000762699	
SNORA69	0.000762699	
SIGLEC12	0.000762699	
RSPH10B	0.000762699	
MIR4324	0.000762699	
GHRH	0.000762699	
GABRR3	0.000762699	
MIR99B	0.000762699	
MYOM3	0.000762699	
FGF5	0.000762699	
SNORA5A	0.000762699	
PLK5	0.000762699	
AMZ1	0.000762699	
RNU12-2P	0.000762699	
HEPHL1	0.000762699	
MACROD2	0.000762699	
GCM2	0.000762699	
HCAR1	0.000762699	
PCAT1	0.000762699	
GOLGA8DP	0.000762699	
SNORA51	0.000762699	
LINC00485	0.000762699	
CHRND	0.000762699	
ATP1A4	0.000762699	
SNORD46	0.000762699	
KLK14	0.000762699	
C4orf47	0.000762699	
TNS4	0.000762699	
SNORD82	0.000762699	
LINC00398	0.000762699	
MYH13	0.000762699	
TMIGD2	0.000762699	

SLC9C2	0.000762699	
ST8SIA2	0.000762699	
ZNF514	0.000762699	
CDRT4	0.000762699	
RPSAP52	0.000762699	
MIR3939	0.000762699	
C4orf45	0.000762699	
CASC1	0.000762699	
SLC22A24	0.000762699	
HNRNPA3P1	0.000762699	
KAAG1	0.000762699	
OMD	0.000762699	
ADAMTS18	0.000762699	
MIR1249	0.000762699	
KIAA1456	0.000762699	
GRM6	0.000762699	
KRT82	0.000762699	
NPY2R	0.000762699	
MIR3180-1	0.000762699	
ZNF793	0.000762699	
C1orf127	0.000762699	
FFAR1	0.000762699	
C12orf56	0.000762699	
MIR590	0.000762699	
VIT	0.000762699	
43722	0.000762699	
PANX3	0.000762699	
CLEC4F	0.000762699	
FABP6	0.000762699	
LRRN4	0.000762699	
MAK	0.000762699	
MIR601	0.000762699	
CCDC37	0.000762699	
PCDHA13	0.000762699	
ZFR2	0.000762699	
MGAT4C	0.000762699	
CA3	0.000762699	
CFAP70	0.000762699	
RNF148	0.000762699	
GUSBP5	0.000762699	
ECT2L	0.000762699	

GUSBP2	0.000762699	
CROCCP3	0.000762699	
OR2W3	0.000762699	
GALNT5	0.000762699	
MSANTD1	0.000762699	
SLC4A9	0.000762699	
TEX12	0.000762699	
TMEM8C	0.000762699	
CHRNA9	0.000762699	
MIR5581	0.000762699	
CT62	0.000762699	
YJEFN3	0.000762699	
RPS16P5	0.000762699	
ASIC5	0.000762699	
KRT79	0.000762699	
SLC35F3	0.000762699	
IDI2	0.000762699	
PKHD1L1	0.000762699	
EGFLAM-AS4	0.000762699	
KCTD16	0.000762699	
GALR1	0.000762699	
CCDC73	0.000762699	
ELOVL3	0.000762699	
DNAJC25-GNG10	0.000762699	
SIGLEC15	0.000762699	
HES7	0.000762699	
URGCP-MRPS24	0.000762699	
DPRXP4	0.000762699	
SALL3	0.000762699	
DUPD1	0.000762699	
SLC6A15	0.000762699	
LRRC14B	0.000762699	
SNORA68	0.000762699	
LINC00092	0.000762699	
MAGEB6	0.000762699	
MIR4999	0.000762699	
MIR301A	0.000762699	
MIR146B	0.000762699	
LHX8	0.000762699	
KRT27	0.000762699	
MIR4635	0.000762699	

RNF212	0.000762699	
SNORA2A	0.000762699	
ZNF679	0.000762699	
TMX2-CTNND1	0.000762699	
SCN7A	0.000762699	
NCR2	0.000762699	
C20orf197	0.000762699	
LRRC36	0.000762699	
OR1L8	0.000762699	
C3orf49	0.000762699	
POM121L9P	0.000762699	
NACAP1	0.000762699	
HRASLS5	0.000762699	
LCN1	0.000762699	
MIR4797	0.000762699	
DNAH7	0.000762699	
XKR7	0.000762699	
RP1	0.000762699	
SLC6A5	0.000762699	
RGPD3	0.000762699	
LRRC3B	0.000762699	
SYNPR	0.000762699	
RRN3P1	0.000762699	
ARHGEF33	0.000762699	
A4GNT	0.000762699	
FRMPD2	0.000762699	
SNORA3	0.000762699	
SLITRK4	0.000762699	
SNORA64	0.000762699	
SPAG5-AS1	0.000762699	
KCNT1	0.000762699	
MTRNR2L2	0.000762699	
BCORP1	0.000762699	
RPL13AP17	0.000762699	
MIR30A	0.000762699	
BMP15	0.000762699	
SNORD79	0.000762699	
IGDCC4	0.000762699	
TDH	0.000762699	
WNT8B	0.000762699	
LENEP	0.000762699	

ARPC3P1	0.000762699	
EREG	0.000762699	
CCDC169	0.000711852	
KIRREL3-AS3	0.000711852	
PRRX2	0.000711852	
ST8SIA3	0.000711852	
C12orf74	0.000711852	
GPR62	0.000711852	
MIR33B	0.000711852	
SUMO1P3	0.000711852	
ZPLD1	0.000711852	
TIFAB	0.000711852	
DIP2A-IT1	0.000711852	
ARHGEF38	0.000711852	
TLL2	0.000711852	
DMRTA2	0.000711852	
ARHGAP11B	0.000711852	
PAGE1	0.000711852	
SEMG2	0.000711852	
AMBN	0.000711852	
FAM218A	0.000711852	
OR6B1	0.000711852	
PTF1A	0.000711852	
KRT33A	0.000711852	
CACNG7	0.000711852	
CCDC129	0.000711852	
IDI2-AS1	0.000711852	
43528	0.000711852	
RP11-715J22.6	0.000711852	
GCOM1	0.000711852	
KBTBD13	0.000711852	
ARL17B	0.000711852	
ZNF32-AS1	0.000711852	
FAM179A	0.000711852	
OSTN	0.000711852	
SAG	0.000711852	
GJA10	0.000711852	
ASXL3	0.000711852	
LINC00486	0.000711852	
C21orf91-OT1	0.000711852	
TNP1	0.000711852	

ABRA	0.000711852	
VAX1	0.000711852	
HIGD2B	0.000711852	
ERVV-1	0.000711852	
CD1E	0.000711852	
VSIG8	0.000711852	
CNGA3	0.000711852	
EMX2OS	0.000711852	
FAM184B	0.000711852	
PPP1R14C	0.000711852	
MIR4668	0.000711852	
NEDD8-MDP1	0.000711852	
MYL2	0.000711852	
VWDE	0.000711852	
MIR5188	0.000711852	
LRRC3C	0.000711852	
UBE2F-SCLY	0.000711852	
RPPH1	0.000711852	
SCRT2	0.000711852	
FHAD1	0.000711852	
DNAJC5G	0.000711852	
CNR2	0.000711852	
CPNE9	0.000711852	
LYG2	0.000711852	
CASP14	0.000711852	
LCN10	0.000711852	
NTNG1	0.000711852	
STK32A	0.000711852	
STX19	0.000711852	
RNF133	0.000711852	
HEATR4	0.000711852	
GBAP1	0.000711852	
ANKRD63	0.000711852	
FRMD1	0.000711852	
C6orf222	0.000711852	
BPI	0.000711852	
FUT5	0.000711852	
ARHGAP19-SLIT1	0.000711852	
ZNF695	0.000711852	
UGT8	0.000711852	
KC6	0.000711852	

GUSBP4	0.000711852	
SNORD59A	0.000711852	
TEKT2	0.000711852	
NYAP2	0.000711852	
HRASLS	0.000711852	
NAA11	0.000711852	
PCCA-AS1	0.000711852	
INO80B-WBP1	0.000711852	
C12orf71	0.000711852	
NSFP1	0.000711852	
BSX	0.000711852	
FLJ46284	0.000711852	
P2RX6P	0.000711852	
FBXO15	0.000711852	
NANOS3	0.000711852	
TCERG1L	0.000711852	
CORIN	0.000711852	
BPIFA2	0.000711852	
YIPF7	0.000711852	
SLC13A1	0.000711852	
C1QTNF3-AMACR	0.000711852	
MYO3B	0.000711852	
ZNF295-AS1	0.000711852	
GJB4	0.000711852	
JAZF1-AS1	0.000711852	
C22orf34	0.000711852	
GNAT3	0.000711852	
MIRLET7C	0.000711852	
PRSS56	0.000711852	
CABP5	0.000711852	
GPHA2	0.000711852	
LYZL1	0.000711852	
SPINK4	0.000711852	
PP2D1	0.000711852	
LRRIQ4	0.000711852	
DSCR8	0.000711852	
C10orf107	0.000711852	
DDX25	0.000711852	
PPP1R27	0.000711852	
HSFX2	0.000711852	
CPLX4	0.000711852	

NMUR2	0.000711852	
GPR156	0.000711852	
CKLF-CMTM1	0.000711852	
GALR3	0.000711852	
KIF25	0.000711852	
CNTN6	0.000711852	
AVPR1B	0.000711852	
CACNG8	0.000711852	
ART1	0.000711852	
MYL1	0.000711852	
ASB11	0.000711852	
ELMOD1	0.000711852	
PPP1R17	0.000711852	
NXPH1	0.000711852	
VSTM2A	0.000711852	
TMED7-TICAM2	0.000711852	
CELA2B	0.000711852	
BCRP3	0.000711852	
GPR82	0.000711852	
ANKRD20A5P	0.000711852	
HIST1H2AJ	0.000711852	
HAPLN2	0.000711852	
DNAH10	0.000711852	
TMEM63C	0.000711852	
DNAH8	0.000711852	
SYNE3	0.000711852	
GPR1	0.000711852	
BTBD17	0.000711852	
DIRC3	0.000711852	
VSX1	0.000711852	
SPATA3	0.000711852	
B3GALT5	0.000711852	
MIR93	0.000711852	
PTH2	0.000711852	
CELA3A	0.000711852	
PDHA2	0.000711852	
RASSF10	0.000711852	
SIMC1	0.000711852	
RIMBP3C	0.000661006	
MIR30C1	0.000661006	
MIR5700	0.000661006	

MIR1226	0.000661006	
SNORD69	0.000661006	
CLECL1	0.000661006	
CALHM1	0.000661006	
HTRA4	0.000661006	
LANCL3	0.000661006	
DSC3	0.000661006	
CYP24A1	0.000661006	
PRSS42	0.000661006	
VAV3-AS1	0.000661006	
SNORA37	0.000661006	
CTD-2201E18.3	0.000661006	
ACSM4	0.000661006	
ENPP6	0.000661006	
MIR153-1	0.000661006	
RP11-273G15.2	0.000661006	
CRACR2A	0.000661006	
KLK15	0.000661006	
NHS	0.000661006	
TMPRSS15	0.000661006	
C7orf69	0.000661006	
OPRK1	0.000661006	
PRDM13	0.000661006	
KLRC3	0.000661006	
FAM83C	0.000661006	
MIR4793	0.000661006	
KLHL33	0.000661006	
TBX20	0.000661006	
DGCR9	0.000661006	
FAM180B	0.000661006	
SULT1A4	0.000661006	
CYCSP52	0.000661006	
DBX1	0.000661006	
RLN3	0.000661006	
CLLU1OS	0.000661006	
RBM46	0.000661006	
GPR111	0.000661006	
MEG8	0.000661006	
C1orf140	0.000661006	
TPSD1	0.000661006	
MIR4642	0.000661006	

MOG	0.000661006	
LINC-ROR	0.000661006	
CA1	0.000661006	
DNAJB13	0.000661006	
IRGM	0.000661006	
EFR3B	0.000661006	
DNASE2B	0.000661006	
GRIN3B	0.000661006	
MYH8	0.000661006	
RPSAP15	0.000661006	
C15orf54	0.000661006	
HMX3	0.000661006	
SLC6A14	0.000661006	
EFHB	0.000661006	
KCTD19	0.000661006	
PABPC1L2B	0.000661006	
CYP2G1P	0.000661006	
BHLHA9	0.000661006	
SPIN2A	0.000661006	
CGB7	0.000661006	
FAM57B	0.000661006	
DRGX	0.000661006	
MIR663A	0.000661006	
PGA5	0.000661006	
MIR320A	0.000661006	
MIR589	0.000661006	
PCDHGA1	0.000661006	
UGT2A1	0.000661006	
IRX2	0.000661006	
LINC00864	0.000661006	
PPP1R2P3	0.000661006	
MYO18B	0.000661006	
LINC00239	0.000661006	
EVPLL	0.000661006	
MYH15	0.000661006	
TERC	0.000661006	
LINC00162	0.000661006	
OR2C1	0.000661006	
MIR4739	0.000661006	
PCDH19	0.000661006	
IGFBPL1	0.000661006	

KIR3DX1	0.000661006	
FLJ41941	0.000661006	
PRR15	0.000661006	
GABRA5	0.000661006	
SLC46A2	0.000661006	
STRIP2	0.000661006	
PRSS57	0.000661006	
KIAA0087	0.000661006	
AGPAT4-IT1	0.000661006	
SCN3B	0.000661006	
SHISA9	0.000661006	
LGALS7B	0.000661006	
COL24A1	0.000661006	
SPINK2	0.000661006	
FAM66B	0.000661006	
UMODL1	0.000661006	
ZSCAN5B	0.000661006	
SPEM1	0.000661006	
USP26	0.000661006	
SLC28A3	0.000661006	
SVOP	0.000661006	
HES2	0.000661006	
RHOXF1	0.000661006	
CDK15	0.000661006	
ANKRD31	0.000661006	
XKR4	0.000661006	
ASB5	0.000661006	
TMEM35	0.000661006	
SBK2	0.000661006	
CAPN14	0.000661006	
OPN1LW	0.000661006	
TSIX	0.000661006	
MIR200B	0.000661006	
FBXW4P1	0.000661006	
KCNH5	0.000661006	
VHLL	0.000661006	
TINAG	0.000661006	
SBF2-AS1	0.000661006	
KBTBD12	0.000661006	
AC004538.3	0.000661006	
SCARNA7	0.000661006	

CXorf58	0.000661006	
FAM169B	0.000661006	
SNORA7B	0.000661006	
CYP27C1	0.000661006	
C20orf166-AS1	0.000661006	
LY75-CD302	0.000661006	
DDX53	0.000661006	
MYO1H	0.000661006	
SNORA62	0.000661006	
C19orf81	0.000661006	
OVCH1	0.000661006	
MIR4733	0.000661006	
HERC2P3	0.000661006	
SPAG17	0.000661006	
SLC35G6	0.000661006	
TAS2R40	0.000661006	
MIR200A	0.000661006	
PSG4	0.000661006	
MIR4420	0.000661006	
THSD7B	0.000661006	
DDX4	0.000661006	
OR5M11	0.000661006	
F8A3	0.000661006	
SPINK13	0.000661006	
KLF17	0.000661006	
KRT73	0.000661006	
SLC6A10P	0.000661006	
PPP1R3A	0.000661006	
BMS1P2	0.000661006	
CAPN13	0.000661006	
OR13J1	0.000661006	
LINC00282	0.000661006	
DPCR1	0.000661006	
MUC17	0.000661006	
OR2K2	0.000661006	
TPTE2P1	0.000661006	
STAC2	0.000661006	
AATK-AS1	0.000661006	
CDH12	0.000661006	
C1QL2	0.000661006	
C10orf55	0.000661006	

UBL4B	0.000661006	
RGS13	0.000661006	
MIR5091	0.000610159	
SCN2B	0.000610159	
OR52B2	0.000610159	
STRA8	0.000610159	
SLC17A8	0.000610159	
KLK9	0.000610159	
C15orf53	0.000610159	
CHI3L2	0.000610159	
MIR4639	0.000610159	
KIAA2022	0.000610159	
RPRML	0.000610159	
NODAL	0.000610159	
SNORD93	0.000610159	
POPDC3	0.000610159	
MIR222	0.000610159	
LBX1	0.000610159	
UGT1A5	0.000610159	
FLJ30679	0.000610159	
RNASE7	0.000610159	
DNAH11	0.000610159	
CERS3	0.000610159	
HILS1	0.000610159	
C17orf64	0.000610159	
AGAP11	0.000610159	
TMSB15A	0.000610159	
RNF224	0.000610159	
OR10A3	0.000610159	
CLRN2	0.000610159	
CLEC4C	0.000610159	
SERPINB4	0.000610159	
IMPG1	0.000610159	
SNORA23	0.000610159	
SSTR4	0.000610159	
LINC00488	0.000610159	
C2orf91	0.000610159	
CDKL4	0.000610159	
MIR4268	0.000610159	
MORC2-AS1	0.000610159	
LRTM1	0.000610159	

LINC00303	0.000610159	
LAMP5	0.000610159	
TUSC5	0.000610159	
GPHB5	0.000610159	
MIR608	0.000610159	
TEX29	0.000610159	
GPR21	0.000610159	
ANP32C	0.000610159	
CACNG6	0.000610159	
MEGF11	0.000610159	
SFTA3	0.000610159	
RIC3	0.000610159	
KCNG4	0.000610159	
FMO6P	0.000610159	
DCHS2	0.000610159	
LYPD5	0.000610159	
DEFA6	0.000610159	
USP9Y	0.000610159	
CSAG1	0.000610159	
ERAS	0.000610159	
MIR3609	0.000610159	
IL1F10	0.000610159	
KLHL34	0.000610159	
LHFPL3	0.000610159	
TUBBP5	0.000610159	
CEACAM8	0.000610159	
MIR3940	0.000610159	
MIR320E	0.000610159	
LY6G6D	0.000610159	
CSN3	0.000610159	
PLA2G2F	0.000610159	
MAP7D2	0.000610159	
CCDC140	0.000610159	
LINC00593	0.000610159	
COL18A1-AS1	0.000610159	
UPK3BL	0.000610159	
GPR110	0.000610159	
RIMBP3B	0.000610159	
LRRC49	0.000610159	
KCNQ2	0.000610159	
LINC00332	0.000610159	

MAGEE1	0.000610159	
AMELX	0.000610159	
CPA2	0.000610159	
DAND5	0.000610159	
GGT6	0.000610159	
MIR4449	0.000610159	
TUBA3E	0.000610159	
ANKK1	0.000610159	
ONECUT3	0.000610159	
MIXL1	0.000610159	
RTP2	0.000610159	
FAM159B	0.000610159	
ADAD2	0.000610159	
CDH26	0.000610159	
BMP3	0.000610159	
SLC32A1	0.000610159	
HORMAD1	0.000610159	
VCX	0.000610159	
POU5F1B	0.000610159	
CACNG1	0.000610159	
PCDHB19P	0.000610159	
DNAH2	0.000610159	
TMPPE	0.000610159	
VWA3B	0.000610159	
GPR45	0.000610159	
MST1L	0.000610159	
RTP1	0.000610159	
C9orf131	0.000610159	
PNPLA1	0.000610159	
OR2A25	0.000610159	
MIR5580	0.000610159	
MIR3663	0.000610159	
CLDN8	0.000610159	
ZNF454	0.000610159	
FGF3	0.000610159	
RD3L	0.000610159	
GIF	0.000610159	
KIF19	0.000610159	
PRR5-ARHGAP8	0.000610159	
PRND	0.000610159	
PATE1	0.000610159	

LRRC26	0.000610159	
TXNDC2	0.000610159	
PURG	0.000610159	
TCL6	0.000610159	
SLITRK6	0.000610159	
MIR32	0.000610159	
PRR21	0.000610159	
FFAR3	0.000610159	
PCDHGA6	0.000610159	
MIR140	0.000610159	
KCNB2	0.000610159	
CLEC17A	0.000610159	
TMEM72	0.000610159	
MIR3193	0.000610159	
CTXN3	0.000610159	
TREML2	0.000610159	
GLRA3	0.000610159	
CORO7-PAM16	0.000610159	
MIR4514	0.000610159	
LIPM	0.000610159	
KRT32	0.000610159	
NAT16	0.000610159	
EXTL1	0.000610159	
MIR1203	0.000610159	
PABPC1L2A	0.000610159	
MIR4768	0.000610159	
RP3-394A18.1	0.000610159	
C10orf131	0.000610159	
43800	0.000559313	
GPR15	0.000559313	
FRMPD4	0.000559313	
PGBD3	0.000559313	
C9orf47	0.000559313	
CHRNA6	0.000559313	
MIR4701	0.000559313	
KCNG2	0.000559313	
ADAM32	0.000559313	
FAM83B	0.000559313	
MAGED4B	0.000559313	
G6PC2	0.000559313	
ANXA2P3	0.000559313	

SIAH3	0.000559313	
FAM196B	0.000559313	
BPIFB3	0.000559313	
SNORD38A	0.000559313	
PMS2L2	0.000559313	
L1TD1	0.000559313	
LRRIQ1	0.000559313	
C1orf194	0.000559313	
KERA	0.000559313	
TP53TG5	0.000559313	
HCAR3	0.000559313	
MIR4435-1	0.000559313	
ACPT	0.000559313	
MIR4721	0.000559313	
LYPD6B	0.000559313	
C17orf99	0.000559313	
SPSB4	0.000559313	
RNF223	0.000559313	
LPAR3	0.000559313	
SNORD124	0.000559313	
SLC25A41	0.000559313	
PCDHGC4	0.000559313	
MAPT-AS1	0.000559313	
CLEC2L	0.000559313	
C3orf20	0.000559313	
CHST6	0.000559313	
SEMG1	0.000559313	
MMP21	0.000559313	
MIR3180-2	0.000559313	
SERPINB12	0.000559313	
SNX19P2	0.000559313	
TMPRSS7	0.000559313	
VAX2	0.000559313	
MDS2	0.000559313	
C18orf42	0.000559313	
GALNT9	0.000559313	
DEFA3	0.000559313	
HELT	0.000559313	
GPR115	0.000559313	
MIR363	0.000559313	
MIR4761	0.000559313	

MIR455	0.000559313	
CDKL2	0.000559313	
C11orf21	0.000559313	
MIR30E	0.000559313	
MARK2P9	0.000559313	
FLJ42969	0.000559313	
SPATA13-AS1	0.000559313	
GRAPL	0.000559313	
APCDD1L	0.000559313	
SAMD12-AS1	0.000559313	
SMTNL1	0.000559313	
COL6A6	0.000559313	
LRRC52	0.000559313	
C7orf65	0.000559313	
ZSCAN23	0.000559313	
NPSR1-AS1	0.000559313	
RAX2	0.000559313	
GPR63	0.000559313	
ANXA8L1	0.000559313	
SNORA71D	0.000559313	
BMP10	0.000559313	
KCNK2	0.000559313	
OR1E2	0.000559313	
CLDN20	0.000559313	
MIR3973	0.000559313	
C10orf71	0.000559313	
OR1N2	0.000559313	
NMBR	0.000559313	
INS-IGF2	0.000559313	
MIR592	0.000559313	
HIST1H1T	0.000559313	
DSG3	0.000559313	
MESTIT1	0.000559313	
AKAP14	0.000559313	
MIR4729	0.000559313	
C20orf203	0.000559313	
ANKRD20A11P	0.000559313	
BVES-AS1	0.000559313	
OR2A1	0.000559313	
KIR2DS4	0.000559313	
FABP2	0.000559313	

FER1L5	0.000559313	
PXT1	0.000559313	
CLEC18A	0.000559313	
C5orf60	0.000559313	
BRDT	0.000559313	
MIR4730	0.000559313	
HYDIN	0.000559313	
MAGEA3	0.000559313	
CACNG3	0.000559313	
OPTC	0.000559313	
FAM181A	0.000559313	
FOXR1	0.000559313	
CCDC83	0.000559313	
MIR602	0.000559313	
MIR593	0.000559313	
MIR4659B	0.000559313	
RGS22	0.000559313	
LRRN1	0.000559313	
SNORD1B	0.000559313	
SLX1A-SULT1A3	0.000559313	
C17orf47	0.000559313	
NACA2	0.000559313	
HCAR2	0.000559313	
COL20A1	0.000559313	
PPP3R2	0.000559313	
MIR5696	0.000559313	
LY6G6F	0.000559313	
LINC00577	0.000559313	
CAPSL	0.000559313	
CSHL1	0.000559313	
LINC00548	0.000559313	
JSRP1	0.000559313	
MIR760	0.000559313	
TCP10	0.000559313	
RIPPLY2	0.000559313	
MKRN9P	0.000559313	
LINC00475	0.000559313	
MFSD2B	0.000559313	
GSX1	0.000559313	
ANGPTL7	0.000559313	
ZDHHC8P1	0.000559313	

OR7E91P	0.000559313	
FAM46D	0.000559313	
MIR4767	0.000559313	
GPR31	0.000559313	
PCDHGA7	0.000559313	
MIR5692B	0.000559313	
ZP1	0.000559313	
NLGN4Y	0.000559313	
ACTL7A	0.000559313	
HBM	0.000559313	
PROK1	0.000559313	
RGPD2	0.000559313	
PAPOLB	0.000559313	
TRPM3	0.000559313	
MIR4674	0.000559313	
ARL2-SNX15	0.000559313	
HES3	0.000559313	
BVES	0.000559313	
VSTM2B	0.000559313	
CCDC60	0.000559313	
CDHR4	0.000559313	
TEPP	0.000559313	
GJB5	0.000559313	
TRIM43	0.000559313	
IMPG2	0.000559313	
PIWIL1	0.000559313	
NIPAL4	0.000559313	
TAGLN3	0.000559313	
CETN4P	0.000559313	
CEACAM16	0.000559313	
C1orf64	0.000508466	
FOXD4L6	0.000508466	
ZNF280A	0.000508466	
TMEM212	0.000508466	
NANOS2	0.000508466	
SNORD123	0.000508466	
FLJ16779	0.000508466	
POLR2J2	0.000508466	
CRYGB	0.000508466	
KLRG2	0.000508466	
KRT34	0.000508466	

TAAR5	0.000508466	
MIR765	0.000508466	
SSX4	0.000508466	
CDRT1	0.000508466	
PLEKHD1	0.000508466	
MIR5685	0.000508466	
SCGB1D2	0.000508466	
RIMS4	0.000508466	
ANKRD20A4	0.000508466	
CST5	0.000508466	
MUC15	0.000508466	
MYL7	0.000508466	
ALX3	0.000508466	
SUN5	0.000508466	
RFPL2	0.000508466	
MIR4450	0.000508466	
FAM19A1	0.000508466	
XKR3	0.000508466	
KRTAP5-1	0.000508466	
PPAN-P2RY11	0.000508466	
SHISA7	0.000508466	
PRSS16	0.000508466	
OR6C3	0.000508466	
CES5AP1	0.000508466	
RXFP3	0.000508466	
NDST4	0.000508466	
ARHGAP40	0.000508466	
TMEM72-AS1	0.000508466	
FSIP2	0.000508466	
MIR4470	0.000508466	
PCDH15	0.000508466	
MIR1286	0.000508466	
GPR151	0.000508466	
C10orf82	0.000508466	
KRTAP7-1	0.000508466	
C14orf178	0.000508466	
FAM71F1	0.000508466	
LINC00521	0.000508466	
MIR3176	0.000508466	
LINC00494	0.000508466	
GFRA4	0.000508466	

LACRT	0.000508466	
ZSCAN10	0.000508466	
IGKV2-40	0.000508466	
PART1	0.000508466	
LIPJ	0.000508466	
TAS2R60	0.000508466	
DNAH3	0.000508466	
ALPPL2	0.000508466	
FXYD4	0.000508466	
ATCAY	0.000508466	
HTR1E	0.000508466	
UMOD	0.000508466	
SLC10A4	0.000508466	
CLEC12B	0.000508466	
TRIM74	0.000508466	
DUSP27	0.000508466	
KCNK4	0.000508466	
SNORA84	0.000508466	
MIR4757	0.000508466	
NPHP3-AS1	0.000508466	
FAM83E	0.000508466	
NOX5	0.000508466	
PLP1	0.000508466	
SNORD9	0.000508466	
LINGO3	0.000508466	
LRIT3	0.000508466	
CTXN2	0.000508466	
PGLYRP4	0.000508466	
MIR3180-3	0.000508466	
C10orf67	0.000508466	
TSPAN16	0.000508466	
TMEM229A	0.000508466	
MIR3926-2	0.000508466	
MS4A10	0.000508466	
CLPSL2	0.000508466	
RGS8	0.000508466	
DLEU2L	0.000508466	
TDGF1P3	0.000508466	
TMEM211	0.000508466	
OR8B3	0.000508466	
FAM170A	0.000508466	

ZSWIM2	0.000508466	
GATSL2	0.000508466	
ARL9	0.000508466	
MIR3651	0.000508466	
C12orf50	0.000508466	
NCR3LG1	0.000508466	
LRRC8E	0.000508466	
SNORA24	0.000508466	
POTEKP	0.000508466	
SLC26A8	0.000508466	
SPDYE2	0.000508466	
SEL1L2	0.000508466	
AGBL4	0.000508466	
GJD3	0.000508466	
WNT8A	0.000508466	
KRT42P	0.000508466	
ZNF280B	0.000508466	
OR5B21	0.000508466	
SNORD63	0.000508466	
GSTA5	0.000508466	
ECEL1P2	0.000508466	
SNORA72	0.000508466	
SERF1A	0.000508466	
FMN2	0.000508466	
ANKRD33	0.000508466	
FAM181B	0.000508466	
LMOD2	0.000508466	
GPR22	0.000508466	
PLEKHG4B	0.000508466	
ATP2B3	0.000508466	
NLRP4	0.000508466	
SLC22A12	0.000508466	
TBC1D21	0.000508466	
CTAG2	0.000508466	
HBG1	0.000508466	
LIM2	0.000508466	
HLTF-AS1	0.000508466	
MIR5703	0.000508466	
GLYATL3	0.000508466	
CCNI2	0.000508466	
SLX1B-SULT1A4	0.000508466	

FABP9	0.000508466	
OR8G1	0.000508466	
SOX2-OT	0.000508466	
MIR3908	0.000508466	
LINC00551	0.000508466	
PRM1	0.000508466	
MIR4646	0.000508466	
PNPLA5	0.000508466	
IGSF10	0.000508466	
MIR505	0.000508466	
MIR5587	0.000508466	
SNX31	0.000508466	
TMEM114	0.000457619	
ZNF177	0.000457619	
ESRG	0.000457619	
SELV	0.000457619	
TSNAX-DISC1	0.000457619	
FAM27C	0.000457619	
SMR3A	0.000457619	
SLC17A6	0.000457619	
LPPR5	0.000457619	
GRID2IP	0.000457619	
NDST3	0.000457619	
MIR5006	0.000457619	
INSM2	0.000457619	
PSMA8	0.000457619	
CLPS	0.000457619	
NUP210L	0.000457619	
AQP10	0.000457619	
NRG3	0.000457619	
CCDC166	0.000457619	
MIR4484	0.000457619	
MIR4641	0.000457619	
TMPRSS11E	0.000457619	
OR52N2	0.000457619	
LINC00565	0.000457619	
GJA8	0.000457619	
RBMXL2	0.000457619	
TTC9B	0.000457619	
ADGB	0.000457619	
OR1G1	0.000457619	

MIR3944	0.000457619	
ADAM21P1	0.000457619	
TBC1D3F	0.000457619	
POTEF	0.000457619	
MIR107	0.000457619	
SCGB2A2	0.000457619	
SNORD126	0.000457619	
LINC00319	0.000457619	
TMEM179	0.000457619	
C7orf34	0.000457619	
ARMC3	0.000457619	
CST4	0.000457619	
OR1Q1	0.000457619	
KRT28	0.000457619	
RD3	0.000457619	
GPC6-AS2	0.000457619	
AMPD1	0.000457619	
TBPL2	0.000457619	
LINC00558	0.000457619	
MIR4690	0.000457619	
MIR578	0.000457619	
KRT77	0.000457619	
KLK5	0.000457619	
MIR548D2	0.000457619	
MIR4319	0.000457619	
CTB-36O1.7	0.000457619	
SMR3B	0.000457619	
H2AFB1	0.000457619	
C11orf88	0.000457619	
IRX1	0.000457619	
FOXD4L4	0.000457619	
VWA3A	0.000457619	
IFNL1	0.000457619	
LINC00538	0.000457619	
C2orf61	0.000457619	
PCDHAC1	0.000457619	
LRRC37A6P	0.000457619	
LINC00284	0.000457619	
OR2A42	0.000457619	
SLFN14	0.000457619	
B3GNT6	0.000457619	

DNAI1	0.000457619	
PRSS54	0.000457619	
HMX1	0.000457619	
MMP26	0.000457619	
COL6A4P2	0.000457619	
SH2D7	0.000457619	
KRT16P2	0.000457619	
BEST2	0.000457619	
FABP12	0.000457619	
ZNF365	0.000457619	
MSX2P1	0.000457619	
BPIFC	0.000457619	
ANKRD34B	0.000457619	
MIR27A	0.000457619	
FNDC9	0.000457619	
SNORD88A	0.000457619	
RFPL3	0.000457619	
SNORD36C	0.000457619	
DEFB103B	0.000457619	
TCN1	0.000457619	
MIR4754	0.000457619	
IL25	0.000457619	
MIR4326	0.000457619	
GPR85	0.000457619	
MIR1301	0.000457619	
HTR1F	0.000457619	
TM4SF19-TCTEX1D2	0.000457619	
FAM81A	0.000457619	
SDR9C7	0.000457619	
CLCA1	0.000457619	
SNORA26	0.000457619	
PRIMA1	0.000457619	
CLEC4D	0.000457619	
MIR4285	0.000457619	
NUDT4P1	0.000457619	
MIR429	0.000457619	
PPP1R42	0.000457619	
FBXO40	0.000457619	
MMP20	0.000457619	
KIR2DL1	0.000457619	
ULK4P1	0.000457619	

C15orf38-AP3S2	0.000457619	
OTOGL	0.000457619	
SKINTL	0.000457619	
TGM5	0.000457619	
SLC38A8	0.000457619	
TTLL9	0.000457619	
ANKRD34C	0.000457619	
IL1RAPL2	0.000457619	
MIR3125	0.000457619	
MS4A15	0.000457619	
CD200R1L	0.000457619	
RNF222	0.000457619	
KRT71	0.000457619	
WSCD2	0.000457619	
MIR3132	0.000457619	
CACNA2D3-AS1	0.000457619	
ENDOU	0.000457619	
FGF14-IT1	0.000457619	
TCEAL6	0.000457619	
CELA3B	0.000457619	
ITPK1-AS1	0.000457619	
LINC00479	0.000457619	
LRIT2	0.000457619	
CALHM3	0.000457619	
MT1P3	0.000457619	
MIR4719	0.000457619	
GPR101	0.000457619	
KRTAP16-1	0.000457619	
MS4A6E	0.000457619	
STXBP5L	0.000457619	
LIPN	0.000457619	
FLJ46066	0.000457619	
PKD1L3	0.000457619	
PENK	0.000457619	
MIR137HG	0.000457619	
SNORD105	0.000457619	
MIR1185-2	0.000457619	
TMEM213	0.000457619	
CHRNB3	0.000457619	
LINC00608	0.000457619	
MIR769	0.000457619	

MIR1307	0.000457619	
FCRL1	0.000457619	
IL36A	0.000457619	
CCL1	0.000457619	
CRISP2	0.000457619	
MIR3661	0.000457619	
IQCA1	0.000457619	
CTAGE9	0.000457619	
KRTDAP	0.000457619	
SCARNA23	0.000457619	
ATOH1	0.000457619	
OR4D10	0.000457619	
TMIGD1	0.000457619	
TCAM1P	0.000457619	
ALDH1L1-AS1	0.000457619	
HSD3BP4	0.000457619	
KLKP1	0.000406773	
C8orf34	0.000406773	
SLC27A6	0.000406773	
MIR1293	0.000406773	
KRTAP21-1	0.000406773	
LINC00111	0.000406773	
PSORS1C2	0.000406773	
NTSR2	0.000406773	
FAM103A2P	0.000406773	
CNGA2	0.000406773	
MIR4786	0.000406773	
CHEK2P2	0.000406773	
ATP13A5	0.000406773	
BAGE2	0.000406773	
ASB18	0.000406773	
VTRNA1-3	0.000406773	
C15orf56	0.000406773	
KCNJ18	0.000406773	
RNF151	0.000406773	
NPHP3-ACAD11	0.000406773	
SATL1	0.000406773	
INGX	0.000406773	
SNORD8	0.000406773	
ACSBG2	0.000406773	
OR10J3	0.000406773	

FOXD4L3	0.000406773	
CCDC169-SOHLH2	0.000406773	
GPR123	0.000406773	
COL19A1	0.000406773	
ANKUB1	0.000406773	
SBSN	0.000406773	
LHX5	0.000406773	
SNORD48	0.000406773	
COL18A1-AS2	0.000406773	
SNORA15	0.000406773	
MIR5582	0.000406773	
MIR27B	0.000406773	
MIR4677	0.000406773	
IGFL4	0.000406773	
MIR574	0.000406773	
MIR629	0.000406773	
ARL13A	0.000406773	
SPANXD	0.000406773	
C14orf39	0.000406773	
UCN3	0.000406773	
KLK12	0.000406773	
IFITM5	0.000406773	
PF4V1	0.000406773	
GDF2	0.000406773	
PRKY	0.000406773	
IL9	0.000406773	
KCTD4	0.000406773	
ETV3L	0.000406773	
COL6A4P1	0.000406773	
AIPL1	0.000406773	
SCARNA21	0.000406773	
CALN1	0.000406773	
MIR323B	0.000406773	
LINC00518	0.000406773	
FAM90A2P	0.000406773	
TMPRSS11A	0.000406773	
MRGPRE	0.000406773	
IGBP1P1	0.000406773	
TPRXL	0.000406773	
SNORA46	0.000406773	
RP11-403A3.3	0.000406773	

SSX2B	0.000406773	
LRRC18	0.000406773	
MIR4505	0.000406773	
STYK1	0.000406773	
ATP4A	0.000406773	
LYPD4	0.000406773	
LINC00304	0.000406773	
SNORD62A	0.000406773	
XAGE5	0.000406773	
NF1P2	0.000406773	
ARHGAP8	0.000406773	
KIR3DL3	0.000406773	
MIR3180-4	0.000406773	
MIR9-1	0.000406773	
NXF5	0.000406773	
PAGE2	0.000406773	
NKX2-4	0.000406773	
IQCJ	0.000406773	
HIST2H2AA4	0.000406773	
RAB40AL	0.000406773	
MIR199A2	0.000406773	
DPP6	0.000406773	
SNORA22	0.000406773	
KNCN	0.000406773	
NBEAP1	0.000406773	
OR1A1	0.000406773	
SCARNA8	0.000406773	
MIR221	0.000406773	
PRM2	0.000406773	
FAM132A	0.000406773	
STARD6	0.000406773	
SCARNA14	0.000406773	
SCARNA2	0.000406773	
LINC00589	0.000406773	
LINC00028	0.000406773	
TNFSF12-TNFSF13	0.000406773	
VTRNA1-1	0.000406773	
FLJ43879	0.000406773	
C2orf70	0.000406773	
ZBBX	0.000406773	
AURKAPS1	0.000406773	

KLK8	0.000406773	
SNORA54	0.000406773	
PIRT	0.000406773	
FAM172BP	0.000406773	
MIR657	0.000406773	
KRTAP8-1	0.000406773	
NKAIN1	0.000406773	
NKX6-3	0.000406773	
BEND2	0.000406773	
MT4	0.000406773	
KCNJ13	0.000406773	
SNORA43	0.000406773	
MIR3912	0.000406773	
DNM1P41	0.000406773	
FAM81B	0.000406773	
ZNF157	0.000406773	
RP11-676J12.7	0.000406773	
FCRL2	0.000406773	
SLC36A3	0.000406773	
VWC2L	0.000406773	
OBP2A	0.000406773	
GSC2	0.000406773	
MIR4685	0.000406773	
PRSS41	0.000406773	
KLHL30-AS1	0.000406773	
VCX3B	0.000406773	
NKAIN4	0.000406773	
BLID	0.000406773	
MFSD6L	0.000406773	
SNORA49	0.000406773	
GPR119	0.000406773	
MIR581	0.000406773	
NAV2-AS4	0.000355926	
LINC00520	0.000355926	
SNORD101	0.000355926	
KLK11	0.000355926	
KLK7	0.000355926	
MIR3194	0.000355926	
C11orf85	0.000355926	
OR6A2	0.000355926	
SNORD105B	0.000355926	

MIR3928	0.000355926	
OR8D4	0.000355926	
GDI2P1	0.000355926	
C14orf183	0.000355926	
BRWD1-AS1	0.000355926	
SPANXC	0.000355926	
OR2H2	0.000355926	
OR6B3	0.000355926	
SLURP1	0.000355926	
RNU5D-1	0.000355926	
LYZL6	0.000355926	
FAM150A	0.000355926	
ODF3	0.000355926	
OLIG1	0.000355926	
SLC22A16	0.000355926	
LYZL2	0.000355926	
DAPL1	0.000355926	
ANKRD26P1	0.000355926	
PRAMEF17	0.000355926	
OR10J5	0.000355926	
FAM215A	0.000355926	
MIR4792	0.000355926	
PAGE2B	0.000355926	
SERINC4	0.000355926	
TULP1	0.000355926	
LINC00221	0.000355926	
FRMD6-AS2	0.000355926	
USP17L2	0.000355926	
C4orf17	0.000355926	
MIR567	0.000355926	
SPATA22	0.000355926	
FTHL17	0.000355926	
RNASE8	0.000355926	
LINC00032	0.000355926	
MIR5691	0.000355926	
C3orf30	0.000355926	
SNORA12	0.000355926	
SULT6B1	0.000355926	
C5orf64	0.000355926	
MAGEL2	0.000355926	
MIR365B	0.000355926	

KRT84	0.000355926	
GAGE2A	0.000355926	
NXPH2	0.000355926	
SNORA76	0.000355926	
RNF216-IT1	0.000355926	
MIR374A	0.000355926	
SNORD11	0.000355926	
KHDC1L	0.000355926	
LINC00599	0.000355926	
MIR4669	0.000355926	
KRTAP10-6	0.000355926	
C2orf73	0.000355926	
LINC00460	0.000355926	
SSX4B	0.000355926	
FMR1NB	0.000355926	
SCGB1C1	0.000355926	
NT5C1A	0.000355926	
CDH22	0.000355926	
MIR572	0.000355926	
NYX	0.000355926	
CST9	0.000355926	
TEX13A	0.000355926	
SNORD15B	0.000355926	
HMHB1	0.000355926	
GPR142	0.000355926	
ADARB2-AS1	0.000355926	
43720	0.000355926	
TEX15	0.000355926	
MIR425	0.000355926	
TMPRSS12	0.000355926	
SERPINB7	0.000355926	
OR13C3	0.000355926	
BEND4	0.000355926	
MIR210	0.000355926	
MIR30C2	0.000355926	
ATP12A	0.000355926	
MCHR2	0.000355926	
WFDC10A	0.000355926	
TMEM174	0.000355926	
SCN10A	0.000355926	
OR52N4	0.000355926	

MIR1250	0.000355926	
MIR320B2	0.000355926	
RTL1	0.000355926	
C10orf99	0.000355926	
CATSPER4	0.000355926	
MIR5089	0.000355926	
MIR1180	0.000355926	
LALBA	0.000355926	
BPIFA1	0.000355926	
KCNS1	0.000355926	
MIR3613	0.000355926	
OR4E2	0.000355926	
UNC13C	0.000355926	
C17orf50	0.000355926	
MIR4522	0.000355926	
KRTAP3-3	0.000355926	
TCHHL1	0.000355926	
OR8D1	0.000355926	
KRT78	0.000355926	
ATP4B	0.000355926	
MGC15885	0.000355926	
SORCS1	0.000355926	
RGPD6	0.000355926	
FAM156B	0.000355926	
OR2B2	0.000355926	
RPS10-NUDT3	0.000355926	
ASTL	0.000355926	
GYPA	0.000355926	
FBXO47	0.000355926	
OPN4	0.000355926	
EGFEM1P	0.000355926	
ZFP42	0.000355926	
SNORD91A	0.000355926	
C4orf51	0.000355926	
MIR5690	0.000355926	
MIR5189	0.000355926	
BASP1P1	0.000355926	
SLC1A6	0.000355926	
TMEM88B	0.000355926	
IL17C	0.000355926	
MIR4795	0.000355926	

GKN1	0.000355926	
C6orf195	0.000355926	
PCDH9-AS2	0.000355926	
TEX33	0.000355926	
ZCCHC13	0.000355926	
GSG1L	0.000355926	
MOXD2P	0.000355926	
OTOS	0.000355926	
DMP1	0.000355926	
CLEC18B	0.000355926	
H1FOO	0.000355926	
CNTNAP5	0.000355926	
SLC18A3	0.000355926	
SDC4P	0.000355926	
PRB3	0.000355926	
PROKR1	0.000355926	
MIR7-3HG	0.000355926	
ZBTB8B	0.000355926	
OR13C9	0.00030508	
CDR1	0.00030508	
MIR10B	0.00030508	
PLSCR5	0.00030508	
MIR2276	0.00030508	
DNAJA1P5	0.00030508	
CECR3	0.00030508	
FOXE1	0.00030508	
INSL6	0.00030508	
SKOR2	0.00030508	
MIR3128	0.00030508	
RAET1L	0.00030508	
LINC00184	0.00030508	
C7orf33	0.00030508	
PPY	0.00030508	
C2orf57	0.00030508	
CA7	0.00030508	
SAGE1	0.00030508	
MIR580	0.00030508	
OCSTAMP	0.00030508	
XAGE1B	0.00030508	
TRIML2	0.00030508	
SNORA42	0.00030508	

OR8S1	0.00030508	
BTBD18	0.00030508	
MIR215	0.00030508	
AWAT2	0.00030508	
FOXI3	0.00030508	
MIR483	0.00030508	
LINC00592	0.00030508	
SLC9A9-AS1	0.00030508	
HHLA1	0.00030508	
TCF24	0.00030508	
OR4D9	0.00030508	
LINGO2	0.00030508	
SNORD115-12	0.00030508	
MIR181C	0.00030508	
LINC00534	0.00030508	
MIR874	0.00030508	
TMEM215	0.00030508	
HNRNPCL1	0.00030508	
ABCC12	0.00030508	
MIR2116	0.00030508	
CT45A5	0.00030508	
C4orf22	0.00030508	
FOXD4L5	0.00030508	
OR2V1	0.00030508	
RNF113B	0.00030508	
OR56B4	0.00030508	
IL17REL	0.00030508	
MS4A3	0.00030508	
OR7E156P	0.00030508	
KRT25	0.00030508	
SHANK2-AS1	0.00030508	
XIRP2	0.00030508	
GHSR	0.00030508	
RIMBP2	0.00030508	
OVCH2	0.00030508	
CLEC19A	0.00030508	
PCGEM1	0.00030508	
IFNE	0.00030508	
FAM19A3	0.00030508	
ACCSL	0.00030508	
RNF17	0.00030508	

C11orf42	0.00030508	
TBATA	0.00030508	
OR51I2	0.00030508	
GAGE2E	0.00030508	
AARD	0.00030508	
SAMSN1-AS1	0.00030508	
SLC7A14	0.00030508	
SPRR2D	0.00030508	
CDCP2	0.00030508	
MAGEC3	0.00030508	
MRGPRX2	0.00030508	
RSPH10B2	0.00030508	
PRG3	0.00030508	
WFDC9	0.00030508	
T	0.00030508	
LCE5A	0.00030508	
HTR3B	0.00030508	
LINC00158	0.00030508	
SLC9A4	0.00030508	
MIR4284	0.00030508	
PRSS37	0.00030508	
MIR4633	0.00030508	
SOHLH1	0.00030508	
LRRC38	0.00030508	
FAM181A-AS1	0.00030508	
ARHGEF3-AS1	0.00030508	
RSPO4	0.00030508	
BCRP2	0.00030508	
UCMA	0.00030508	
SLC7A3	0.00030508	
IRG1	0.00030508	
TGIF2LY	0.00030508	
DEFB103A	0.00030508	
MIR3165	0.00030508	
KRTAP10-12	0.00030508	
GRXCR2	0.00030508	
FEV	0.00030508	
FLJ36000	0.00030508	
KRT3	0.00030508	
PSG5	0.00030508	
SFTA2	0.00030508	

TUBB8	0.00030508	
TAAR6	0.00030508	
ZNF80	0.00030508	
OR1E1	0.00030508	
MIR4759	0.00030508	
MIR1205	0.00030508	
MT1JP	0.00030508	
C20orf173	0.00030508	
CNPY1	0.00030508	
PAGE4	0.00030508	
GML	0.00030508	
NDFIP2-AS1	0.00030508	
MYO3A	0.00030508	
ADAMTS20	0.00030508	
LINC00343	0.00030508	
FAM26D	0.00030508	
GABRA6	0.00030508	
MIR2355	0.00030508	
MAGEA9	0.00030508	
KIR3DL2	0.00030508	
PSG7	0.00030508	
SLC35D3	0.00030508	
SSX8	0.00030508	
GRK7	0.00030508	
MIR345	0.00030508	
SVOPL	0.00030508	
SPINT3	0.00030508	
IGSF5	0.00030508	
OR2AG2	0.00030508	
OR51Q1	0.00030508	
MIR3660	0.00030508	
PRSS33	0.00030508	
KCTD8	0.00030508	
MIR365A	0.00030508	
CHURC1-FNTB	0.00030508	
PRR23C	0.00030508	
TRIM6-TRIM34	0.00030508	
OR13D1	0.00030508	
LNX1-AS2	0.00030508	
LINC00327	0.00030508	
SLC25A31	0.00030508	

CRYGN	0.00030508	
MIR199A1	0.000254233	
OR5C1	0.000254233	
VTRNA1-2	0.000254233	
TSPAN19	0.000254233	
GPC5-AS1	0.000254233	
OPN5	0.000254233	
PGPEP1L	0.000254233	
NKAIN3	0.000254233	
LCE1C	0.000254233	
S100G	0.000254233	
MIR556	0.000254233	
DEFB106A	0.000254233	
PRICKLE2-AS2	0.000254233	
CEACAM7	0.000254233	
SPATA4	0.000254233	
MIR4706	0.000254233	
MIR766	0.000254233	
OXGR1	0.000254233	
LY6D	0.000254233	
SPACA4	0.000254233	
KRT26	0.000254233	
CEP57L1P1	0.000254233	
SYCP1	0.000254233	
RGPD4	0.000254233	
FAM66D	0.000254233	
HBBP1	0.000254233	
ADAM30	0.000254233	
FAM71A	0.000254233	
OR10AD1	0.000254233	
WFDC10B	0.000254233	
KCNA10	0.000254233	
CRYGC	0.000254233	
MIR623	0.000254233	
MIR4663	0.000254233	
TMEFF1	0.000254233	
ZFP57	0.000254233	
SORCS3	0.000254233	
CAPNS2	0.000254233	
MIR4716	0.000254233	
TBC1D3	0.000254233	

SNORA77	0.000254233	
C1orf146	0.000254233	
GLRA4	0.000254233	
ANGPTL5	0.000254233	
RNU5E-1	0.000254233	
MIR4518	0.000254233	
GUCY2GP	0.000254233	
MYO16-AS1	0.000254233	
HTR5A	0.000254233	
IL31	0.000254233	
TMPRSS11D	0.000254233	
MIR770	0.000254233	
GUCA1A	0.000254233	
OR5AP2	0.000254233	
RNY4	0.000254233	
C21orf62	0.000254233	
TMSB15B	0.000254233	
FAM66A	0.000254233	
LRRC10	0.000254233	
MIR320C1	0.000254233	
MIR31HG	0.000254233	
BTF3P11	0.000254233	
OR9I1	0.000254233	
NANOGNB	0.000254233	
MAGEE2	0.000254233	
C16orf92	0.000254233	
LNX1-AS1	0.000254233	
TMEM132C	0.000254233	
MIR4515	0.000254233	
LINC00606	0.000254233	
OR10Q1	0.000254233	
OR8A1	0.000254233	
BTG4	0.000254233	
TFAP2D	0.000254233	
MIR5583-1	0.000254233	
KPRP	0.000254233	
SNORD23	0.000254233	
CLDN10-AS1	0.000254233	
MIR4497	0.000254233	
MIR644A	0.000254233	
SLC5A8	0.000254233	

TPTE	0.000254233	
C10orf53	0.000254233	
BRS3	0.000254233	
MIR4673	0.000254233	
ZP4	0.000254233	
DMRTC2	0.000254233	
LCN15	0.000254233	
PRLH	0.000254233	
C4orf26	0.000254233	
OR4N2	0.000254233	
STRC	0.000254233	
MIR548C	0.000254233	
OR2B11	0.000254233	
CER1	0.000254233	
MIR499A	0.000254233	
CLDN24	0.000254233	
MAGEB1	0.000254233	
PSG8	0.000254233	
IQCF6	0.000254233	
KIRREL3-AS2	0.000254233	
CHST5	0.000254233	
PP12613	0.000254233	
SNORA70B	0.000254233	
MIR7-3	0.000254233	
OR1I1	0.000254233	
CLVS2	0.000254233	
THEG	0.000254233	
IFNA5	0.000254233	
DEFA5	0.000254233	
DDX11L9	0.000254233	
KRTAP4-9	0.000254233	
STOML3	0.000254233	
MIR26A1	0.000254233	
NPFFR1	0.000254233	
IFIT1B	0.000254233	
MIR4295	0.000254233	
ATP6V0A4	0.000254233	
SPHKAP	0.000254233	
PTCHD1-AS	0.000254233	
SNORA2B	0.000254233	
SEZ6L	0.000254233	

SLC15A5	0.000254233	
NLRP10	0.000254233	
MIR550A2	0.000254233	
GGNBP1	0.000254233	
MIR583	0.000254233	
PIN1P1	0.000254233	
SPZ1	0.000254233	
MIR3922	0.000254233	
LRTM2	0.000254233	
PPIAL4C	0.000254233	
NPSR1	0.000254233	
WDR87	0.000254233	
TMEM207	0.000254233	
LINC00359	0.000254233	
PABPN1L	0.000254233	
C10orf91	0.000254233	
MIR4714	0.000254233	
SLC6A18	0.000254233	
C9orf50	0.000254233	
MIR3179-3	0.000254233	
HDGFL1	0.000254233	
ZAR1	0.000254233	
OSTCP1	0.000254233	
CLEC3A	0.000254233	
MIR24-2	0.000254233	
TRIML1	0.000254233	
USP17L7	0.000254233	
SNORD11B	0.000254233	
EFCAB3	0.000254233	
FAM41C	0.000254233	
PRICKLE2-AS3	0.000254233	
SPACA3	0.000254233	
IL22RA2	0.000254233	
OR8B8	0.000254233	
MIR1913	0.000254233	
PPEF2	0.000254233	
IZUMO2	0.000203386	
POM121L4P	0.000203386	
OR3A2	0.000203386	
FAM58BP	0.000203386	
DEFB104A	0.000203386	

NHEG1	0.000203386	
C10orf120	0.000203386	
FLJ42393	0.000203386	
INMT-FAM188B	0.000203386	
SNORD71	0.000203386	
MIR4262	0.000203386	
PSG6	0.000203386	
MIR548AC	0.000203386	
TLR8-AS1	0.000203386	
CLLU1	0.000203386	
BANCR	0.000203386	
PHEX-AS1	0.000203386	
KRTAP20-1	0.000203386	
KRT16P3	0.000203386	
LINC00446	0.000203386	
DPYD-AS1	0.000203386	
MIR708	0.000203386	
LINC00052	0.000203386	
MIR34C	0.000203386	
GPX5	0.000203386	
PSG11	0.000203386	
OR4M1	0.000203386	
DAZ2	0.000203386	
GYG2P1	0.000203386	
MIR3155A	0.000203386	
OR52N1	0.000203386	
REG1B	0.000203386	
MIR1284	0.000203386	
TEX26	0.000203386	
PROKR2	0.000203386	
KRTAP4-7	0.000203386	
MIR450A1	0.000203386	
FLJ40288	0.000203386	
LMAN1L	0.000203386	
MIR3162	0.000203386	
CD300LD	0.000203386	
LIX1	0.000203386	
AMTN	0.000203386	
PPIAL4E	0.000203386	
PCDHA9	0.000203386	
DSCAM-AS1	0.000203386	

MIR3199-2	0.000203386	
MIR5192	0.000203386	
MIR4323	0.000203386	
ARL5C	0.000203386	
TMCO5A	0.000203386	
METTL11B	0.000203386	
UOX	0.000203386	
OR11L1	0.000203386	
LINC00379	0.000203386	
MIR23B	0.000203386	
LINC00314	0.000203386	
MIR3151	0.000203386	
NLRP5	0.000203386	
FAM216B	0.000203386	
OR52M1	0.000203386	
TRIM61	0.000203386	
CEACAM18	0.000203386	
MIR4686	0.000203386	
AKNAD1	0.000203386	
TPRG1-AS2	0.000203386	
SPPL2C	0.000203386	
GPR33	0.000203386	
LINC00029	0.000203386	
HIST1H4G	0.000203386	
OR1J4	0.000203386	
KRT75	0.000203386	
FAM187B	0.000203386	
MIR4456	0.000203386	
SNORD67	0.000203386	
PCDH20	0.000203386	
MIR101-2	0.000203386	
MAGI1-AS1	0.000203386	
HSPB2-C11orf52	0.000203386	
PMP2	0.000203386	
TCEB3CL	0.000203386	
LINC00112	0.000203386	
FOXR2	0.000203386	
MIR3197	0.000203386	
ENTHD1	0.000203386	
MIR642A	0.000203386	
KRTAP6-3	0.000203386	

LINC00226	0.000203386	
MIR4472-2	0.000203386	
ABCC5-AS1	0.000203386	
MIR449C	0.000203386	
C2orf80	0.000203386	
FAM138A	0.000203386	
OR2F2	0.000203386	
HNRNPKP3	0.000203386	
LCE1D	0.000203386	
OR56A5	0.000203386	
APOBEC4	0.000203386	
NXNL1	0.000203386	
PBOV1	0.000203386	
OR5H2	0.000203386	
MIR1200	0.000203386	
OR51B6	0.000203386	
WFDC13	0.000203386	
MIR2278	0.000203386	
MIR193B	0.000203386	
MIR1537	0.000203386	
LIPK	0.000203386	
SNORD111B	0.000203386	
MIR4672	0.000203386	
KRT19P2	0.000203386	
OR2L13	0.000203386	
C16orf78	0.000203386	
MIR4711	0.000203386	
DUSP21	0.000203386	
MUC22	0.000203386	
MIR519B	0.000203386	
GAGE1	0.000203386	
SNORD111	0.000203386	
CLRN1	0.000203386	
OR4C45	0.000203386	
CACNG5	0.000203386	
GDF3	0.000203386	
KRT76	0.000203386	
MIR128-1	0.000203386	
XAGE2B	0.000203386	
FAM138E	0.000203386	
C1QTNF8	0.000203386	

KRTAP11-1	0.000203386	
LINC00229	0.000203386	
SNORA80B	0.000203386	
GJD4	0.000203386	
CGB8	0.000203386	
CRYGA	0.000203386	
NPVF	0.000203386	
OR5AK2	0.000203386	
FREM3	0.000203386	
SNORA30	0.000203386	
MIR4632	0.000203386	
DEFB124	0.000203386	
OTOP3	0.000203386	
PPIAL4G	0.000203386	
FAM92B	0.000203386	
PPIAL4B	0.000203386	
C7orf66	0.000203386	
OR6B2	0.000203386	
HMGB4	0.000203386	
FAM138B	0.000203386	
MAS1	0.000203386	
NEUROD4	0.00015254	
MIR4464	0.00015254	
KIF2B	0.00015254	
CT45A1	0.00015254	
OR6K6	0.00015254	
VTRNA2-1	0.00015254	
MIR548A3	0.00015254	
LINC00254	0.00015254	
OR4A16	0.00015254	
CABS1	0.00015254	
FAM71B	0.00015254	
CYMP	0.00015254	
IFNL3	0.00015254	
CLEC18C	0.00015254	
FAM66E	0.00015254	
OR6C6	0.00015254	
MIR3179-1	0.00015254	
GPR78	0.00015254	
SHCBP1L	0.00015254	
MIR541	0.00015254	

IQCF2	0.00015254	
TMPRSS11B	0.00015254	
CLPSL1	0.00015254	
KRTAP4-4	0.00015254	
FER1L6-AS1	0.00015254	
KRT37	0.00015254	
MIR4762	0.00015254	
MIR877	0.00015254	
SPANXN2	0.00015254	
MTUS2-AS1	0.00015254	
RBMXL3	0.00015254	
OR6K2	0.00015254	
BAGE5	0.00015254	
PAGE3	0.00015254	
DHRS7C	0.00015254	
OR8K1	0.00015254	
MIR23C	0.00015254	
MIR138-2	0.00015254	
OR4A47	0.00015254	
PRB4	0.00015254	
PRSS48	0.00015254	
ANKRD20A2	0.00015254	
MIR550A3	0.00015254	
LRRD1	0.00015254	
COTL1P1	0.00015254	
MIR4460	0.00015254	
MIR181B1	0.00015254	
TSPYL6	0.00015254	
ARHGAP26-AS1	0.00015254	
DMRT2	0.00015254	
OR13H1	0.00015254	
SCARNA1	0.00015254	
PIWIL3	0.00015254	
MIR548Y	0.00015254	
OR2T33	0.00015254	
MIR148B	0.00015254	
MIR3945	0.00015254	
GAGE12J	0.00015254	
LINC00477	0.00015254	
REG3G	0.00015254	
ADAM29	0.00015254	

OR10G2	0.00015254	
AMELY	0.00015254	
RSPO1	0.00015254	
ISY1-RAB43	0.00015254	
RAD21L1	0.00015254	
RSPH6A	0.00015254	
DCAF12L1	0.00015254	
MIR4253	0.00015254	
MIR4440	0.00015254	
OR13C2	0.00015254	
MIR190A	0.00015254	
KRTAP19-7	0.00015254	
MIR143	0.00015254	
SNORA40	0.00015254	
LINC00309	0.00015254	
ANKRD20A3	0.00015254	
MIR4744	0.00015254	
SNORD90	0.00015254	
C5orf52	0.00015254	
LARS2-AS1	0.00015254	
LY6G6E	0.00015254	
ABCC13	0.00015254	
POTEH	0.00015254	
OR5M8	0.00015254	
MIR542	0.00015254	
DBX2	0.00015254	
DEFB119	0.00015254	
SNORD41	0.00015254	
DIAPH3-AS1	0.00015254	
OR2AT4	0.00015254	
CELP	0.00015254	
GPRC6A	0.00015254	
TBC1D26	0.00015254	
OR51M1	0.00015254	
C10orf113	0.00015254	
TCL1B	0.00015254	
PPIAL4F	0.00015254	
PRSS38	0.00015254	
GPR152	0.00015254	
BPIFB6	0.00015254	
C8orf74	0.00015254	

EDDM3B	0.00015254	
MIR1208	0.00015254	
AGBL1	0.00015254	
MIR5698	0.00015254	
TSPY1	0.00015254	
MIR194-1	0.00015254	
DEFB116	0.00015254	
RNASE12	0.00015254	
MAGEB10	0.00015254	
IFNL2	0.00015254	
OR2F1	0.00015254	
DMRTC1B	0.00015254	
MIR569	0.00015254	
CST9L	0.00015254	
OR2A2	0.00015254	
C7orf72	0.00015254	
MIR3173	0.00015254	
OR8B12	0.00015254	
SNORA44	0.00015254	
MIR634	0.00015254	
KRTAP13-2	0.00015254	
EIF4E1B	0.00015254	
OR9G4	0.00015254	
CRNN	0.00015254	
SNORA19	0.00015254	
SNORD27	0.00015254	
TDRD5	0.00015254	
OR6S1	0.00015254	
C17orf74	0.00015254	
XAGE1A	0.00015254	
ZC2HC1B	0.00015254	
CTAG1A	0.00015254	
OR5AK4P	0.00015254	
MIR30B	0.00015254	
MS4A13	0.00015254	
LRRC72	0.00015254	
S100A7A	0.00015254	
PPY2	0.00015254	
TAS2R1	0.00015254	
C17orf98	0.00015254	
MIR4727	0.00015254	

MIR4694	0.00015254	
NOX3	0.00015254	
SSX7	0.00015254	
OR4B1	0.00015254	
ZNF321P	0.00015254	
MIR1252	0.000101693	
PRB1	0.000101693	
OR51I1	0.000101693	
LINC00305	0.000101693	
GPR139	0.000101693	
MIR337	0.000101693	
THEGL	0.000101693	
IFNA6	0.000101693	
MIR4660	0.000101693	
MIR3183	0.000101693	
GPR6	0.000101693	
TGM6	0.000101693	
PPIAL4D	0.000101693	
OR6K3	0.000101693	
MIR1272	0.000101693	
LINC00210	0.000101693	
MIR4303	0.000101693	
MIR4312	0.000101693	
DKFZP434L187	0.000101693	
ADAD1	0.000101693	
OR3A1	0.000101693	
SPRR2G	0.000101693	
OR52K1	0.000101693	
MIR4637	0.000101693	
MRGPRX4	0.000101693	
CT45A3	0.000101693	
DNAJB8	0.000101693	
C1orf87	0.000101693	
MIR3130-1	0.000101693	
TSPY2	0.000101693	
MIR5692C2	0.000101693	
KLRF2	0.000101693	
LYZL4	0.000101693	
OR2D2	0.000101693	
MTOR-AS1	0.000101693	
IGHV1OR15-1	0.000101693	

EFCAB9	0.000101693	
PRAMEF12	0.000101693	
MIR1343	0.000101693	
USP29	0.000101693	
ZNF32-AS3	0.000101693	
LZTS1-AS1	0.000101693	
SPATS1	0.000101693	
LELP1	0.000101693	
CHODL-AS1	0.000101693	
MIR3136	0.000101693	
SNORA11B	0.000101693	
NOTO	0.000101693	
MIR4790	0.000101693	
MIR374B	0.000101693	
CXorf49B	0.000101693	
TTC29	0.000101693	
GPR87	0.000101693	
TXNDC8	0.000101693	
C12orf40	0.000101693	
SNORD114-21	0.000101693	
LIPI	0.000101693	
OR4X2	0.000101693	
TMCO5B	0.000101693	
GOLGA6L2	0.000101693	
SNORA36A	0.000101693	
SPAG11B	0.000101693	
FLJ45079	0.000101693	
TAAR9	0.000101693	
CCKAR	0.000101693	
MIR3909	0.000101693	
C2orf83	0.000101693	
IGSF11-AS1	0.000101693	
MIR4474	0.000101693	
C16orf82	0.000101693	
OR4D1	0.000101693	
SLC35F4	0.000101693	
SNORD121A	0.000101693	
MIR3178	0.000101693	
ANKRD20A1	0.000101693	
DNAJB8-AS1	0.000101693	
OR5K2	0.000101693	

C17orf77	0.000101693	
PSG10P	0.000101693	
OR52E2	0.000101693	
MIR4308	0.000101693	
OR2A14	0.000101693	
CRISP1	0.000101693	
C16orf90	0.000101693	
OR6P1	0.000101693	
VN1R2	0.000101693	
MIR33A	0.000101693	
OR5AU1	0.000101693	
TFAMP1	0.000101693	
LINC00330	0.000101693	
PRR23B	0.000101693	
FOXB2	0.000101693	
ZNF705D	0.000101693	
ATP6V1G3	0.000101693	
MIR5693	0.000101693	
DEFB126	0.000101693	
RBMY2FP	0.000101693	
MIR5093	0.000101693	
MIR3925	0.000101693	
OR11H6	0.000101693	
LINC00507	0.000101693	
KIAA1024L	0.000101693	
MIR548F3	0.000101693	
PPBPP2	0.000101693	
EPGN	0.000101693	
FTH1P18	0.000101693	
KRTAP1-5	0.000101693	
SNORA8	0.000101693	
SNORD127	0.000101693	
KRTAP6-1	0.000101693	
MIR4765	0.000101693	
MIR152	0.000101693	
OR52E4	0.000101693	
HTR3E	0.000101693	
OR1A2	0.000101693	
MIR138-1	0.000101693	
GAS2L2	0.000101693	
CCDC105	0.000101693	

METTL21C	0.000101693	
MIR526B	0.000101693	
OR1D4	0.000101693	
MIR598	0.0000508	
HYALP1	0.0000508	
TSPY10	0.0000508	
MIR4695	0.0000508	
OR5K1	0.0000508	
GPR32	0.0000508	
SNORD116-1	0.0000508	
F8A2	0.0000508	
LINC00457	0.0000508	
OR7G3	0.0000508	
OR10C1	0.0000508	
OTOR	0.0000508	
SNORD56B	0.0000508	
IFNA21	0.0000508	
LRRC30	0.0000508	
MIR5196	0.0000508	
C15orf32	0.0000508	
MIR761	0.0000508	
KRTAP4-6	0.0000508	
MIR5095	0.0000508	
KRTAP1-3	0.0000508	
MAGEA9B	0.0000508	
OR4F15	0.0000508	
MIR559	0.0000508	
MIA-RAB4B	0.0000508	
GLT6D1	0.0000508	
OPALIN	0.0000508	
C2orf27B	0.0000508	
OR4C13	0.0000508	
KRT24	0.0000508	
ATXN8OS	0.0000508	
DAOA-AS1	0.0000508	
PCA3	0.0000508	
MIR7-2	0.0000508	
CARTPT	0.0000508	
DMRTC1	0.0000508	
MAGED4	0.0000508	
SNORD5	0.0000508	

LINC00051	0.0000508	
TBC1D28	0.0000508	
OR56B1	0.0000508	
NKX2-6	0.0000508	
MIR4654	0.0000508	
BPIFA3	0.0000508	
OR51A7	0.0000508	
DYX1C1-CCPG1	0.0000508	
RGS21	0.0000508	
OR11G2	0.0000508	
MMD2	0.0000508	
OR52L1	0.0000508	
LINC00297	0.0000508	
CXorf49	0.0000508	
MIR648	0.0000508	
KRTAP17-1	0.0000508	
SSX6	0.0000508	
LINC00423	0.0000508	
IFNW1	0.0000508	
MYL10	0.0000508	
OR51G2	0.0000508	
OR8H3	0.0000508	
KCNK16	0.0000508	
FAM9C	0.0000508	
PRR9	0.0000508	
TECRL	0.0000508	
MYADML	0.0000508	
KRTAP12-3	0.0000508	
SNORD114-22	0.0000508	
MIR548W	0.0000508	
WFDC5	0.0000508	
IFNK	0.0000508	
OR9A4	0.0000508	
GOT1L1	0.0000508	
HMGB3P1	0.0000508	
MIR4532	0.0000508	
FIGLA	0.0000508	
MIR3122	0.0000508	
SAMD7	0.0000508	
OR5B12	0.0000508	
MIR892A	0.0000508	

OR10J1	0.0000508	
OR11A1	0.0000508	
OR2V2	0.0000508	
MIR4753	0.0000508	
SERPINB10	0.0000508	
OR4F13P	0.0000508	
TMPRSS11F	0.0000508	
MIR181B2	0.0000508	
MIR4748	0.0000508	
TDRG1	0.0000508	
OR10V2P	0.0000508	
MIR555	0.0000508	
C8orf87	0.0000508	
OR1L3	0.0000508	
GOLGA8G	0.0000508	
KCNIP4-IT1	0.0000508	
ASB10	0.0000508	
MIR378E	0.0000508	
MIR3126	0	
LINC00301	0	
MIR421	0	
OR4D6	0	
MIR4500	0	
ODF4	0	
MIR4429	0	
NOBOX	0	
MIR942	0	
CST11	0	
SPAG11A	0	
PCNAP1	0	
MIR4314	0	
MIR4728	0	
MIR298	0	
MIR34B	0	
MIR1278	0	
SNORD92	0	
C6orf118	0	
OR5AR1	0	
MIR4469	0	
DEFB123	0	
CT47A6	0	

FAM24A	0	
POTEA	0	
GPR26	0	
RPTN	0	
FSBP	0	
FLJ31662	0	
TPTE2P6	0	
BSPH1	0	
MIR4652	0	
