# Supplementary material for: Identification of Key Components in Colon Adenocarcinoma Using Transcriptome to Interactome Multilayer Framework
Source: Sci Rep. 2020 Mar 19;10:4991. doi: 10.1038/s41598-020-59605-z (PMC7081269; doi:10.1038/s41598-020-59605-z)
Supplement: Supplementary file 2 — supplementary information 2. [file 41598_2020_59605_MOESM2_ESM.pdf]

Identification of key components in colon adenocarcinoma using transcriptome to interactome multilayer framework

Ehsan Pournoor[1], Zaynab Mousavian[2], Abbas Nowzari Dalini[2], Ali Masoudi-Nejad[1*]

[1] Laboratory of Systems Biology and Bioinformatics (LBB), Institute of Biochemistry and Biophysics, University of Tehran, Tehran, Iran

[2] School of Mathematics, Statistics, and Computer Science, College of Science, University of Tehran, Tehran, Iran

***Corresponding Author**

Ali Masoudi-Nejad, Ph.D.
Laboratory of Systems Biology and Bioinformatics (LBB)
Institute of Biochemistry and Biophysics
University of Tehran, Tehran, Iran
E-mail: amasoudin@ut.ac.ir
WWW: http://LBB.ut.ac.ir
Tel: +98-21-6695-9256
Fax: +98-21-6640-4680

The modules overlap with COAD-related genes. Color density is demonstrating degree of overlap.

module seed	RW_score	# of module genes	# of colon genes in module	percentage	pValue	FDR B&H	FDR B&Y	Bonferroni
GABPA	1	44	6	0.136364				
MAX	0.99445772	370	89	0.240541				
CTCF	0.98947476	64	12	0.1875				
SP1	0.97772919	239	99	0.414226	2.67E-33	3.54E-30	3.24E-29	1.41E-29
MYC	0.95754309	41	3	0.073171				
EGR1	0.85874816	250	116	0.464	3.34E-44	3.31E-41	3.07E-40	1.99E-40
USF1	0.8058677	153	94	0.614379	5.5E-48	3E-44	2.7E-43	3E-44
TFAP2A	0.7116998	381	93	0.244094				
YY1	0.67407332	140	51	0.364286	4.20E-15	7.65E-13	6.72E-12	1.53E-11

E2F1	0.55910917	156	39	0.25	6.14E-07	3.27E-05	2.24E-04	1.19E-07
MXI1	0.5235674	63	19	0.301587	3.69E-05	1.95E-03	1.51E-02	4.82E-02
JUN	0.51105914	140	64	0.457143	2.98E-25	3.84E-22	3.39E-21	1.15E-21

Modules genes

module seed	module genes
GABPA	AICDA, TSNAX, POLRMT, GUK1, IMPDH1, GMPS, AK1, ENTPD4, CANT1, AK5, GOLGA3, ENTPD1, DUT, NT5E, NT5C2, IMPDH2, C1D, NT5M, AK2, ITPA, ADA, MORC3, ENTPD6, TK2, TFB1M, DTYMK, PNP, TFB2M, TYMP, CDA, ENTPD2, ACP2, NT5C1B, USP16, AK7, NT5C, DCTD, TYMS, ENTPD3, GABPA, TK1, ENTPD5, TFAM, AK4
MAX	RPL35, BAG2, EEF1B2P3, RPL27A, TUBB4A, HIST1H2BM, LAS1L, IGF2BP1, TBL2, HSPA8, PCBP2, PAK1IP1, RPL18A, HIST1H1D, NLRP1, RPS14, RPL3, HNRNPM, GTF3C4, GTPBP4, DEDD2, DHX30, IKBKG, CASP7, RUVBL1, DICER1, MRPS2, MAP3K7, GNB2L1, EIF3C, RPL15, TNFRSF1A, MRPS22, ELAVL1, RP11-51O6.1, DCTN1, RPS18, CD4, TRAF3, CASP6, MYBBP1A, CFLAR, YWHAE, RPL36, RPL31, TUBA1A, KRT18, GEMIN4, GNAI2, VCP, DDB1, RPL9, RPS12, ACTG1, RPL17, HNRNPA1, TUBA3C, RPS7, RPL12, EPRS, RPL5, TUBB, GAPDH, KPNB1, SLC25A3, HSPA5, RPS20, RPL22, TNPO1, BRAF, RPS15A, EEF1A2, NAT10, TRAF6, WDR18, RPLP2, TIMM50, RPL37, BIRC3, RPL39, ACTB, EIF3K, TEX10, WDR77, SLC25A6, AKT2, RPL8, NFKBIB, PHB2, TRAF1, RPS2, RBM28, ENO1, MAP3K1, TRAF2, HSPA9, RPL14, DDX5, LCK, HNRNPC, HNRNPU, DNAJA1, MAP3K3, CLTC, IKBKE, ARRB1, RPL13, PYCARD, FBXL13, NCL, NHP2L1, RPS5, XIAP, RPS17, RPS9, PHF5A, HNRNPL, ANXA6, KRR1, MAP3K5, RPS27A, MCF2L, EIF4A2, RPL28, RPS3A, UBB, EIF2AK2, RPS24, C18orf32, RPL18, MAX, AKT1, SRPK1, SF3B2, SNHG6, EIF3B, RPL18AP3, RPL29, BCL2L1, HSP90AB1, MME, EIF4A3, TRADD, RPS4Y2, RPL7A, HNRNPA2B1, U2AF1, EIF3E, PWP1, MRPS23, RPL10, RPL23, TANK, DDX21, BIRC2, TOP1, EIF3D, LRPPRC, GMEB1, RPSA, ILF3, RPS4Y1, LUC7L2, TCP1, MATR3, UBC, CASP4, RPL36A, HNRNPD, FMR1, SYNE1, CASP1, CALM2, HSPD1, RYBP, SF3B1, RPS27L, RPS19, NFKBIA, MRPS15, SF3B3, PABPC3, PTCD3, NFKBIE, RPL6, GTF3C1, PSEN2, SLC25A5, EEF1A1, SCYL2, MOV10, IRAK1, TNFRSF1B, RPL27, POLR1C, TXLNG, HNRNPH2, ILF2, RPL37A, RPL19, DDX47, DHCR7, TAB2, PSEN1, TRPC4AP, MAPT, CASP3, CALM1, RPS16, RPL39P3, MAP2K1, HSP90AA1, MRPS16, RPS25, DHX36, IGF2BP3, RPL24, NFKB2, RPS26, RELB, NOD1, POLR2H, RPS27, FXR2, FBL, CASP9, RAN, TBK1, MRPS5, RPL23A, RPS21, UBA52, RPL30, CASP2, EIF3F, YWHAH, RPL21, RPS10, TCOF1, ANXA2, PABPC1, SYNCRIP, ANXA1, SART3, HSPA1L, RIPK1, RPN1, RPL26, DDX20, ZNF326, RIPK2, CUL1, HTT, PELP1, RPLP1, CTB-63M22.1, DSP, PPP2R2B, BID, RPL41, RP11-466H18.1, DLX4, RSL1D1, TAB1, SRSF10, CASP10, DHX9, GNL3, CALM3, NPM1, CAD, DDX18, LRRC59, C1QBP, RPS23, MYL6, ARRB2, HSPA1A, NLRC4, PRPF3, DIMT1, IKBKB, PCNA, EIF3I, CHUK, RPS6, RBM10, IMMT, HNRNPF, RPL38, RPL11, TNFAIP3, ACTG2, U2AF2, RPLP0, DDX3X, SNRNP200, SSRP1, MRPS27, COPA, TUBB2B, DDOST, RPS28, RPL7P9, BCL10, RP11-234A1.1, VDAC1, RPL4, RB1, UPF1, SKP1, RPL13AP20, MAP3K8, SRP14, RPL3P4, MAP3K14, NOLC1, NACA, RUVBL2, TUBA3D, RPS15, BRIX1, RPL7, H2AFX, PHB, CDC37, RPL32, RAF1, TUBB1, RPS3, DHX15, GEMIN2, ATAD3B, PRKCZ, SFPQ, DEDD, FADD, GCN1L1, RPL34, YWHAZ, EEF1B2, NOP58, H1FX, RPL10A, PRKDC, PRMT5, RIPK3, RPS4X, YWHAB, CASP5, NAP1L1, ATP5C1, RPS13, FXR1, LYAR, RPL13A, YBX1, EIF3M, FLG2, EBNA1BP2, POLR1A, RPL35A, RPS8, RPS11, CASP8, TUFM, VIM, EEF2, RPS29, HNRNPH1
CTCF	DPF2, HDAC2, SMARCC2, CHD3, MTA2, ARID4B, ALKBH2, SMARCB1, ESPL1, RBBP7, PHF21A, ARID1A, PDS5B, ACTB, TAF9, CHD4, SYMPK, HNRNPC, SMARCA5, CPSF2, HSPB9, STAG2, PDS5A, MBD3, EFTUD2, SMC3, BRMS1, SMC1A, WDR5, SMARCD2, RBBP4, MBD3L1, BRMS1L, SAP18, SIN3A, TAF12, RAD21,

	GATAD2B, RELB, RAN, SMARCD3, SAP30, SMARCC1, WAPAL, ING1, ACTL6A, TAF6, POLR2A, STAG1, RBP1, MBD2, HDAC1, KDM1A, BAZ1A, RCOR1, SMARCD1, SMARCA4, SMARCE1, MTA1, RBBP5, TAF1, CTCF, SMARCA2, SIN3B
SP1	CSNK2A2, KLF5, DPF2, BCL2, SMAD3, HDAC2, SMARCC2, PIAS1, XRCC6, CHD3, DNMT3B, MTA2, CYP3A7, SAP130, GTF2H1, TAF5, SNW1, TDG, ARID4B, CDK7, THRB, CREBBP, LEF1, ETS2, SMARCB1, RARA, ARID4A, GTF2E2, MYBL2, RBBP7, KRT16, RXRB, PHF21A, SP4, ARID1A, SUMO2, MAPK3, PIAS3, RARG, CDKN1A, ACTB, RUNX3, CCND1, TAF11, TAF9, CHD4, PIAS2, RBPJ, BRCA2, LCAT, RUNX1, CCNC, SYMPK, PPARA, CBX5, COL2A1, SMAD2, KAT2A, SKI, ERCC3, SMARCA5, CSNK2A1, CDK8, SNIP1, LPL, CPSF2, HIPK2, EED, MBD3, SP3, NCOR2, UBE2I, H3F3A, MAPK1, GTF2A1, AKT1, BRMS1, BARD1, MAPK8, WDR5, AR, GTF2F2, CTNNB1, NRIP1, SMARCD2, MAPK14, SMAD7, GATAD2A, RBBP4, ARID1B, RAD51, SP1, NCOA2, PGR, HDAC3, TOP2B, BRMS1L, ZBTB16, TGM4, NCOA6, SAP18, HIF1A, TRIM24, BCL3, BSG, DACH1, CDK9, SIN3A, TAF12, CCNE1, MED21, HMG20B, CASP3, CHAF1A, DAXX, ZHX1, DNMT1, ETS1, TAF8, GTF2F1, GATAD2B, ZBTB7A, SATB1, RXRG, SUMO1, MSX1, RELB, GTF2E1, DNMT3A, RAN, TGFBR2, KAT2B, PML, SMARCD3, HIST3H3, SUV39H1, CTBP1, HDAC9, TAF4, HTT, ZNF451, RARB, SUDS3, CBX1, MED1, RBL2, CCNA2, SAP30, SMARCC1, SMAD1, CBS, THRA, ING1, ACTL6A, VEGFA, TAF6, NCOA1, SMAD4, POLR2A, ATF7IP, GTF2B, CDH1, NR0B2, PCNA, RBP1, NR1H2, TRIM28, FHL2, TAF10, MBD2, HDAC1, SUPT16H, MBD1, CDKN2A, PADI3, KDM1A, BAZ1A, MDM2, PPARD, CYP27A1, RB1, TGFBR1, RCOR1, SMARCD1, ARID3A, SMARCA4, SMARCE1, MTA1, PIAS4, CBX3, GRIN1, CDK4, PARP1, SP100, SETDB1, RBBP5, HSF1, TP73, HIST4H4, ESR2, HDAC5, TAF1, MAOB, ZMYM2, BAZ1B, NKX2-1, TAF2, NCOA3, NCOR1, MYB, PSMC5, SKIL, UBE2K, MEF2C, PRPF40A, MAPK9, PRKDC, NR4A1, EZH2, BRCA1, BCL6, VDR, UTRN, TAF7, MECP2, SIRT1, SMARCA2, MYOD1, RBL1, SIN3B, LOR, HDAC4, TRRAP, TLE1, CASP8, MECOM, MUC4, CYP17A1, NR1H3
MYC	SIKE1, PPP2R4, PPP4C, DOCK5, CCT2, PDCD10, TUBB, STK25, PPP2R1B, PPP2R1A, CCT7, PPT1, CTTNBP2NL, MAP3K3, STRN4, ABCB7, CCT3, TCP1, CTTNBP2, PPP2CA, IGBP1, TBK1, CCT6A, MOB4, CCT5, FGFR1OP2, PPP2R2B, MYC, TRAF3IP3, CCT8, ANKHD1, STRN3, TUBA8, CCT4, TUBA1B, STRN, PPP2CB, SARS, PPP2R2D, STK24, PPP2R2C
EGR1	ID2, CSNK2A2, STUB1, ACVR1, BCL2, SMAD3, NAB1, HDAC2, SMARCC2, PIAS1, XRCC6, CHD3, CDK6, MTA2, YWHAE, GTF2H1, CHEK1, SNW1, ZNF8, CDK7, THRB, MAPK6, CREBBP, LEF1, ETS2, SMARCB1, RARA, XRCC5, RASD2, MYBL2, SRC, RBBP7, BMPR1B, RXRB, PHF21A, WT1, SP4, PPARGC1A, SMAD6, ARID1A, FOXM1, SUMO2, MAPK3, RARG, CDKN1A, RNF111, ACTB, RUNX3, RUNX2, CCND1, TAF9, CHD4, PIAS2, PAK1, RBPJ, RUNX1, CCNC, SYMPK, PPARA, CBX5, COL2A1, SMAD2, KAT2A, SKI, ERCC3, SMARCA5, CSNK2A1, CDK8, SNIP1, GSK3B, CPSF2, SMURF2, TERT, MBD3, SMURF1, SP3, NCOR2, ZEB2, SPEN, UBE2I, EFTUD2, MAPK1, UBB, GLI3, AKT1, BRMS1, MAPK8, TGFB1, WDR5, STRAP, CTNNB1, BIRC5, RAB38, NRIP1, SMARCD2, MAPK14, SMAD7, RBBP4, UBC, ARID1B, RAD51, NOTCH1, NCOA2, PITX1, CDK1, PGR, HDAC3, TOP2B, ZBTB16, NCOA6, SAP18, HIF1A, TRIM24, BCL3, NFKBIA, RET, CDK9, SIN3A, TAF12, HDAC7, MED21, CASP3, CHAF1A, DAXX, HSP90AA1, DNMT1, ETS1, GTF2F1, GATAD2B, ZBTB7A, SATB1, RXRG, SUMO1, RELB, CDK2, GTF2E1, DNMT3A, RAN, RASL12, TGFBR2, FN1, KAT2B, SQSTM1, PML, SMARCD3, HIST3H3, CTBP1, HDAC9, TAF4, ABCA2, HTT, SMAD5, RARB, SMAD9, MED1, TGIF1, CCNA2, SAP30, SMARCC1, SMAD1, CBS, THRA, RELA, CTBP2, ING1, ACTL6A, VEGFA, TAF6, FLI1, NCOA1, SMAD4, POLR2A, GTF2B, CDH1, NR0B2, IKBKB, PCNA, CHUK, TOP2A, USF2, NR1H2, FHL2, MBD2, HDAC1, SUPT16H, MBD1, PSMD11, CDKN2A, CCNB1, COPS5, KDM1A, MDM2, CCND2, PPARD, TOB1, HSPA4, RB1, TGFBR1, RCOR1, SMARCD1, CIITA, SMARCA4, CCND3, SMARCE1, MTA1, PIAS4, CDK4, PARP1, SETDB1, RBBP5, HSF1, DVL1, TP73, HIST4H4, ESR2, HDAC5, TAF1, MAOB, DAB2, ERBB2IP, NCOA3, NCOR1, MYB, SKIL, MEF2C, PPP1CA, MAPK9, ERBB2, PRKDC, NR4A1, EZH2, SNRNP70, BRCA1, BCL6, VDR, CDKN1B, MECP2, SIRT1, SMARCA2, MYOD1, EGR1, RBL1, SIN3B, HDAC4, TRRAP, TLE1, CASP8, MECOM, AXIN1, NR1H3
USF1	CSNK2A2, BCL2, SMAD3, HDAC2, SMARCC2, PIAS1, XRCC6, CHD3, PSG2, CDK6, PTMA, SNW1, TDG, CDK7, THRB, MAPK6, ABL1, CREBBP, LEF1, ETS2, SMARCB1, RARA, XRCC5, STAT3, SRC, RBBP7, NR3C1, RXRB, WT1, PPARGC1A, MAPK3, PIAS3, RARG, CDKN1A, ACTB, RUNX2, CCND1, TAF9, RUNX1, PPARA, TYR, USF1, SMAD2, SKI, CSNK2A1, GSK3B, TERT, WRN, HIPK2, SP3, NCOR2, UBE2I, MAPK1, AKT1, FOXO3, MAPK8, AR,

	CTNNB1, NRIP1, TOP1, MAPK14, SMAD7, RBBP4, FOXO1, UBC, NCOA2, CEBPA, CDK1, PGR, HDAC3, EGFR, ZBTB16, NCOA6, HIF1A, TRIM24, NFKBIA, SIN3A, RUNX1T1, DAXX, HSP90AA1, ETS1, RXRG, SUMO1, RELB, CDK2, RXRA, STAT5B, KAT2B, YWHAH, PML, CTBP1, RARB, MED1, MITF, TGIF1, CCNA2, SMARCC1, SMAD1, THRA, RELA, ING1, VEGFA, EP300, NCOA1, SMAD4, POLR2A, GTF2B, CDH1, NR0B2, IKBKB, PCNA, CHUK, TOP2A, NR1H2, FHL2, HDAC1, TBP, CCNB1, MDM2, PPARD, RB1, SMARCA4, SMARCE1, PIAS4, CDK4, PARP1, TP53, HSF1, TP73, ESR2, ESR1, TAF1, CEBPB, NCOA3, NCOR1, MYB, SKIL, MEF2C, STAT5A, MAPK9, PRKDC, NR4A1, EZH2, BRCA1, BCL6, VDR, SIRT1, SMARCA2, MYOD1, RBL1, HDAC4, APC, NR1H3
TFAP2A	RPL35, BAG2, EEF1B2P3, RPL27A, TUBB4A, HIST1H2BM, LAS1L, CLTA, IGF2BP1, TBL2, HSPA8, PCBP2, PAK1IP1, RPL18A, HIST1H1D, RPS14, RPL3, HNRNPM, GTF3C4, GTPBP4, DEDD2, DHX30, IKBKG, FAS, CASP7, RUVBL1, DICER1, MRPS2, MAP3K7, GNB2L1, EIF3C, RPL15, TNFRSF1A, MRPS22, ELAVL1, RP11-51O6.1, DCTN1, RPS18, CD4, TRAF3, CASP6, MYBBP1A, CFLAR, YWHAE, RPL36, FLNA, RPL31, TUBA1A, KRT18, GEMIN4, GNAI2, VCP, DDB1, RPL9, RPS12, ACTG1, RPL17, HNRNPA1, KCNIP3, TUBA3C, RPS7, RPL12, EPRS, RPL5, TUBB, GAPDH, KPNB1, SLC25A3, HSPA5, RPS20, RPL22, TNPO1, BRAF, RPS15A, NAT10, TRAF6, SLC25A21, WDR18, RPLP2, TIMM50, RPL37, BIRC3, HIP1, RPL39, ACTB, EIF3K, TEX10, WDR77, SLC25A6, AKT2, RPL8, NFKBIB, PHB2, TRAF1, RPS2, HIP1R, RBM28, EMG1, ENO1, MAP3K1, TRAF2, HSPA9, RPL14, DDX5, LCK, HNRNPC, HNRNPU, DNAJA1, MAP3K3, CLTC, IKBKE, ARRB1, RPL13, FBXL13, NCL, NHP2L1, RPS5, XIAP, RPS17, RPS9, PHF5A, HNRNPL, ANXA6, KRR1, MAP3K5, RPS27A, TFAP2A, MCF2L, EIF4A2, RPL28, RPS3A, UBB, EIF2AK2, RPS24, C18orf32, RPL18, AKT1, SRPK1, SF3B2, SNHG6, IKBKAP, EIF3B, RPL18AP3, RPL29, BCL2L1, HSP90AB1, MME, EIF4A3, TRADD, CGB, RPS4Y2, RPL7A, HNRNPA2B1, U2AF1, EIF3E, CTNNB1, PWP1, PRKCI, MRPS23, RPL10, RPL23, TANK, DDX21, BIRC2, TOP1, EIF3D, LRPPRC, RPSA, ILF3, RPS4Y1, LUC7L2, TCP1, MATR3, UBC, CASP4, RPL36A, HNRNPD, FMR1, SYNE1, CASP1, CALM2, HSPD1, EGFR, SF3B1, FARSB, RPS27L, RPS19, NFKBIA, MRPS15, SF3B3, PABPC3, PTCD3, NFKBIE, RPL6, GTF3C1, PSEN2, SLC25A5, EEF1A1, SCYL2, MOV10, IRAK1, TNFRSF1B, RPL27, POLR1C, HNRNPH2, ILF2, RPL37A, RPL19, DDX47, DHCR7, TAB2, PSEN1, MAPT, CASP3, CALM1, RPS16, RPL39P3, MAP2K1, HSP90AA1, LDLRAP1, MRPS16, APP, RPS25, DHX36, IGF2BP3, RPL24, RPS26, RELB, NOD1, POLR2H, RPS27, FXR2, FBL, CASP9, TGM2, RAN, TBK1, MRPS5, RPL23A, RPS21, UBA52, RPL30, CASP2, EIF3F, YWHAH, RPL21, RPS10, TCOF1, ANXA2, PABPC1, SYNCRIP, ANXA1, SART3, HSPA1L, RIPK1, RPN1, RPL26, DDX20, ZNF326, RIPK2, CUL1, HTT, PELP1, RPLP1, CTB-63M22.1, DSP, RPL41, RP11-466H18.1, DLX4, RSL1D1, TAB1, SRSF10, CASP10, DHX9, GNL3, CALM3, NPM1, CAD, DDX18, LRRC59, C1QBP, AKT3, RPS23, MYL6, ARRB2, HSPA1A, CDH1, PRPF3, DIMT1, IKBKB, PCNA, EIF3I, CHUK, RPS6, RBM10, IMMT, HNRNPF, RPL38, RPL11, TNFAIP3, ACTG2, U2AF2, RPLP0, DDX3X, SNRNP200, SSRP1, MRPS27, COPA, TUBB2B, DDOST, RPS28, RPL7P9, BCL10, RP11-234A1.1, VDAC1, RPL4, RB1, UPF1, RPL13AP20, MAP3K8, SRP14, RPL3P4, MAP3K14, NOLC1, EPN2, CFTR, NACA, RUVBL2, TUBA3D, RPS15, BRIX1, RPL7, H2AFX, PHB, CDC37, RPL32, RAF1, TUBB1, PRKCQ, RPS3, DHX15, GEMIN2, ATAD3B, PRKCZ, DEDD, FADD, GCN1L1, RPL34, YWHAZ, EEF1B2, CLTB, NOP58, H1FX, RPL10A, PRKDC, PRMT5, RIPK3, RPS4X, YWHAB, NAP1L1, ATP5C1, IRS1, RPS13, FXR1, LYAR, MYOD1, RPL13A, YBX1, EIF3M, FLG2, EBNA1BP2, POLR1A, RPL35A, RPS8, RPS11, CASP8, TUFM, VIM, EEF2, RPS29, HNRNPH1
YY1	CSNK2A2, SMAD3, HDAC2, SMARCC2, PIAS1, DHX30, CHD3, MTA2, SNW1, ARID4B, CDK7, THRB, CREBBP, SMARCB1, RARA, ARID4A, RBBP7, RXRB, HMGA1, PHF21A, HSPA5, ARID1A, SUMO2, MAPK3, RARG, CDKN1A, ACTB, CCND1, TAF9, CHD4, RBPJ, RUNX1, SYMPK, PPARA, SMAD2, SMARCA5, CSNK2A1, CPSF2, MBD3, NCOR2, SPEN, H3F3A, EFTUD2, MAPK1, AKT1, BRMS1, MAPK8, WDR5, NRIP1, SMARCD2, MAPK14, RBBP4, YY1, ARID1B, PGR, HDAC3, TOP2B, BRMS1L, ZBTB16, NCOA6, SAP18, HIF1A, NFKBIA, INO80, CDK9, SIN3A, TAF12, HDAC7, RUNX1T1, MED21, DAXX, HSP90AA1, GTF2F1, GATAD2B, SUMO1, RELB, CDK2, RXRA, RAN, IFNB1, KAT2B, YWHAH, PML, SMARCD3, SUV39H1, CTBP1, HDAC9, NR2E3, RARB, MED1, TGIF1, SAP30, SMARCC1, SMAD1, THRA, RELA, MXD1, ING1, ACTL6A, TAF6, NCOA1, SMAD4, POLR2A, GTF2B, RBP1, MBD2, HDAC1, SUPT16H, KDM1A, MDM2, PPARD, RB1, RCOR1, SMARCD1, SMARCA4, SMARCE1, MTA1, PARP1, RBBP5, HSF1, HIST4H4, ESR2, HDAC5, TAF1, BAZ1B, NCOA3, NCOR1, SKIL, MEF2C, NR4A1, BRCA1, BCL6, VDR, MECP2, SMARCA2, MYOD1, RBL1, SIN3B, HDAC4, ATF2

E2F1	CENPL, BUB1B, LIN9, KIF11, NUSAP1, TFDP2, ASPM, SHCBP1, CCNB2, CDK6, RACGAP1, MCM6, MAD2L1, UBE2C, C17orf53, CDC20, NCAPG, WDR76, CHEK1, RAD54L, CDK7, MCM4, DEPDC1B, CDC45, TACC3, KIF4A, CDC7, MYBL2, MCM5, FOXM1, MCM2, TFDP1, PCLAF, PARPBP, CCND1, HJURP, RFC1, PRC1, E2F8, GTSE1, DEPDC1, CENPF, SKA3, CDC25C, SKP2, SPC25, HELLS, CDKN3, CDCA5, NEK2, KNTC1, STIL, ORC4, CKAP2L, SKA1, POLQ, RFC4, RPA2, BIRC5, KIF23, CENPM, RPA1, PLK4, E2F1, RAD51, TPX2, CDK1, CEP55, SPDL1, CLSPN, UBTF, RAD51AP1, CCNE1, KIFC1, CCNF, MCM7, FANCI, SGO2, CDT1, CENPE, BUB1, CDK2, CCNA1, RBBP8, KIF2C, ORC6, TTK, ANLN, EME1, CHAF1B, KIF20A, TCF19, TICRR, PKMYT1, RBL2, ATAD5, CCNA2, ORC5, ASF1B, PLK1, NUF2, GINS1, ARHGAP11A, DBF4B, MCM3, TOP2A, CDCA2, RRM2, NCAPG2, ORC1, SGO1, CDKN2A, MCM10, CCNB1, ORC3, ZWINT, MELK, RB1, WDR62, CDC6, DBF4, CENPK, XRCC2, KIF18B, MKI67, KIF15, CDK4, CENPA, NDC80, KIF18A, HMMR, TAF1, CENPI, LMNB1, UBE2T, CIP2A, KIF14, EZH2, BRCA1, TRIP13, ECT2, EXO1, CDKN1B, RBL1, PTTG1, PRR11, WDHD1, TROAP, DLGAP5, CDCA8, FANCD2, ORC2, CDCA3, APC, AXIN1, CTNNB1,
MXI1	HDAC2, SMARCC2, CHD3, MTA2, SAP130, ARID4B, SMARCB1, ARID4A, RBBP7, PHF21A, HSPA5, ARID1A, ACTB, TAF9, CHD4, SETD1A, SYMPK, HCFC1, SMARCA5, CPSF2, MBD3, OGT, EFTUD2, BRMS1, WDR5, SMARCD2, RBBP4, HDAC3, BRMS1L, SAP18, SIN3A, TAF12, HSP90AA1, GATAD2B, RELB, RAN, SMARCD3, ING2, HIST3H3, ASH2L, SUDS3, SAP30, SMARCC1, MXD1, ING1, ACTL6A, TAF6, RBP1, MBD2, HDAC1, KDM1A, HSPA4, RCOR1, SMARCD1, SMARCA4, SMARCE1, MTA1, RBBP5, HSF1, TAF1, MXI1, SMARCA2, SIN3B
JUN	BCL2, SMAD3, HDAC2, PIAS1, DHX30, XRCC6, CHD3, MTA2, TP63, SNW1, TDG, THRB, CREBBP, SMARCB1, RARA, TSHB, AFG3L1P, MOK, RBBP7, RXRB, PPARGC1A, ARID1A, SUMO2, MAPK3, RARG, CDKN1A, NR1H4, CCND1, TAF9, CHD4, RUNX1, CCNC, SYMPK, PPARA, HCFC1, ESRRA, SMAD2, SMARCA5, CSNK2A1, CDK8, CPSF2, HIPK2, MBD3, NCOR2, SPEN, UBE2I, EFTUD2, MAPK1, AKT1, BRMS1, GATA2, MAPK8, WDR5, TRIP4, NRIP1, SMARCD2, MAPK14, SMAD7, RBBP4, NCOA2, PGR, HDAC3, ZBTB16, NCOA6, SAP18, HIF1A, TRIM24, BCL3, SIN3A, TAF12, MED21, DAXX, ETS1, GTF2F1, GATAD2B, RXRG, SUMO1, CDK2, RXRA, HHEX, RAN, KAT2B, YWHAH, PML, SMARCD3, NR1I3, CTBP1, RARB, MED1, TGIF1, SAP30, SMARCC1, THRA, ING1, ACTL6A, TAF6, NCOA1, SMAD4, POLR2A, GTF2B, NR0B2, NR1H2, TAF10, MBD2, HDAC1, KDM1A, MDM2, PPARD, RB1, SMARCD1, SMARCA4, MTA1, NR1I2, CDK4, PARP1, JUN, RBBP5, TP73, ESR2, HDAC5, TAF1, NKX2-1, NCOA3, NCOR1, PSMC5, SKIL, MEF2C, MAPK9, NR4A1, POU1F1, BRCA1, BCL6, VDR, SNRK, SMARCA2, MYOD1, RBL1, SIN3B, HDAC4, NR1H3
