# Supplementary material for: Identification of Key Components in Colon Adenocarcinoma Using Transcriptome to Interactome Multilayer Framework
Source: Sci Rep. 2020 Mar 19;10:4991. doi: 10.1038/s41598-020-59605-z (PMC7081269; doi:10.1038/s41598-020-59605-z)
Supplement: Supplementary file 3 — supplementary information 3. [file 41598_2020_59605_MOESM3_ESM.pdf]

Identification of key components in colon adenocarcinoma using transcriptome to interactome multilayer framework

Ehsan Pournoor[1], Zaynab Mousavian[2], Abbas Nowzari Dalini[2], Ali Masoudi-Nejad[1*]

[1] Laboratory of Systems Biology and Bioinformatics (LBB), Institute of Biochemistry and Biophysics, University of Tehran, Tehran, Iran

[2] School of Mathematics, Statistics, and Computer Science, College of Science, University of Tehran, Tehran, Iran

***Corresponding Author**

Ali Masoudi-Nejad, Ph.D.
Laboratory of Systems Biology and Bioinformatics (LBB)
Institute of Biochemistry and Biophysics
University of Tehran, Tehran, Iran
E-mail: amasoudin@ut.ac.ir
WWW: http://LBB.ut.ac.ir
Tel: +98-21-6695-9256
Fax: +98-21-6640-4680

Biological processes related to module USF1

Name	pValue	FDR B&H	Genes from Input	Genes in Annotation
positive regulation of gene expression	4.99E-94	2.534E-90	123	1890
positive regulation of macromolecule biosynthetic process	1.72E-89	4.356E-86	118	1777
positive regulation of nitrogen compound metabolic process	6.89E-88	1.167E-84	120	1944
positive regulation of RNA metabolic process	2.1E-87	2.328E-84	113	1593
positive regulation of RNA biosynthetic process	2.29E-87	2.328E-84	112	1546
positive regulation of cellular biosynthetic process	5.4E-87	4.569E-84	119	1921
positive regulation of transcription, DNA-templated	1.97E-86	1.25E-83	111	1528
positive regulation of nucleic acid-templated transcription	1.97E-86	1.25E-83	111	1528
positive regulation of biosynthetic process	5.54E-86	3.125E-83	119	1959

regulation of transcription by RNA polymerase II	1.25E-85	6.348E-83	118	1916
positive regulation of nucleobase-containing compound metabolic process	6.25E-85	2.888E-82	116	1832
positive regulation of transcription by RNA polymerase II	7.32E-78	3.098E-75	95	1094
response to endogenous stimulus	8.01E-72	3.13E-69	105	1740
cellular response to organic cyclic compound	1.75E-71	6.348E-69	74	561
negative regulation of gene expression	2.06E-69	6.975E-67	101	1627
response to organic cyclic compound	2.48E-68	7.861E-66	87	1045
negative regulation of transcription, DNA-templated	3.2E-65	9.568E-63	90	1261
cellular response to endogenous stimulus	1.05E-64	2.966E-62	90	1278
negative regulation of nucleic acid-templated transcription	5.85E-64	1.564E-61	90	1303
response to lipid	1.16E-63	2.935E-61	83	1023
negative regulation of cellular macromolecule biosynthetic process	1.24E-63	3.004E-61	93	1452
negative regulation of RNA biosynthetic process	1.61E-63	3.719E-61	90	1318
negative regulation of RNA metabolic process	1.94E-63	4.293E-61	91	1366
negative regulation of transcription by RNA polymerase II	2.38E-62	5.03E-60	76	806
negative regulation of macromolecule biosynthetic process	2.6E-61	5.276E-59	93	1540
negative regulation of nucleobase-containing compound metabolic process	7.12E-61	1.391E-58	92	1508
negative regulation of nitrogen compound metabolic process	4.42E-60	8.325E-58	94	1640
negative regulation of cellular biosynthetic process	1.09E-59	1.974E-57	93	1605
negative regulation of biosynthetic process	5.77E-59	1.011E-56	93	1635
cellular response to lipid	1.44E-57	2.431E-55	65	572
response to steroid hormone	2.55E-57	4.185E-55	65	577
intracellular receptor signaling pathway	8.54E-57	1.356E-54	53	299
regulation of cell proliferation	8.17E-56	1.258E-53	91	1666
response to hormone	1.73E-55	2.578E-53	77	1033
cellular response to hormone stimulus	1.84E-55	2.669E-53	67	677
cellular response to steroid hormone stimulus	8.89E-53	1.255E-50	50	290
hormone-mediated signaling pathway	1.17E-51	1.608E-49	46	229

steroid hormone mediated signaling pathway	9.93E-51	1.328E-48	43	189
regulation of cell death	2.54E-50	3.304E-48	86	1650
regulation of apoptotic process	8.86E-50	1.125E-47	83	1519
regulation of programmed cell death	2.14E-49	2.649E-47	83	1536
programmed cell death	9.85E-49	1.191E-46	90	1952
apoptotic process	3.53E-48	4.175E-46	89	1923
regulation of cell differentiation	4.73E-47	5.459E-45	84	1699
positive regulation of developmental process	5.58E-43	6.294E-41	73	1323
response to oxygen-containing compound	3.36E-41	3.714E-39	77	1614
negative regulation of cell death	8.03E-41	8.681E-39	64	1001
cellular response to oxygen-containing compound	1.07E-39	1.132E-37	63	1001
chromosome organization	1.7E-39	1.763E-37	67	1189
regulation of multicellular organismal development	3.02E-39	3.068E-37	80	1893

Pathways related to module USF1

Term	P-value	Adj. P-value	Z-score
Androgen receptor signaling pathway WP138	6.79E-58	2.17E-55	-1.38815
TGF-beta Signaling Pathway WP366	7.66E-38	1.23E-35	-1.2601
Integrated Breast Cancer Pathway WP1984	1.72E-37	1.84E-35	-0.99879
Nuclear Receptors WP170	6.06E-31	4.85E-29	-1.53161
Adipogenesis WP236	8.28E-30	5.3E-28	-1.04883
Cell Cycle WP179	1.46E-27	7.77E-26	-1.11229
AGE/RAGE pathway WP2324	2.48E-25	1.13E-23	-1.9584
Retinoblastoma Gene in Cancer WP2446	2.01E-24	8.06E-23	-1.66274
Pancreatic adenocarcinoma pathway WP4263	3.32E-24	1.18E-22	-1.59432
Non-small cell lung cancer WP4255	1.44E-23	4.62E-22	-1.53125
Breast cancer pathway WP4262	2.32E-23	6.76E-22	-0.90715
RAC1/PAK1/p38/MMP2 Pathway WP3303	2.65E-23	7.06E-22	-1.39478
Oncostatin M Signaling Pathway WP2374	5.77E-22	1.35E-20	-1.85675
Circadian rhythm related genes WP3594	5.89E-22	1.35E-20	-0.88343
TGF-beta Receptor Signaling WP560	1.01E-21	2.16E-20	-2.33059
VEGFA-VEGFR2 Signaling Pathway WP3888	1.54E-21	3.09E-20	-0.79477

DNA Damage Response (only ATM dependent) WP710	1.04E-20	1.97E-19	-1.06194
Signaling Pathways in Glioblastoma WP2261	4.49E-20	7.98E-19	-1.49679
TNF related weak inducer of apoptosis (TWEAK) Signaling Pathway WP2036	5.64E-20	9.49E-19	-2.38891
Brain-Derived Neurotrophic Factor (BDNF) signaling pathway WP2380	8.93E-20	1.43E-18	-0.86385
Chromosomal and microsatellite instability in colorectal cancer WP4216	2.24E-19	3.41E-18	-1.18043
Energy Metabolism WP1541	3.53E-19	5.13E-18	-1.95291
Leptin signaling pathway WP2034	4.51E-19	6.28E-18	-1.01867
Hepatitis C and Hepatocellular Carcinoma WP3646	6.88E-19	9.17E-18	-1.7872
IL-4 Signaling Pathway WP395	3.2E-18	4.1E-17	-1.68254
RANKL/RANK (Receptor activator of NFKB (ligand)) Signaling Pathway WP2018	4.26E-18	5.25E-17	-1.67297
miRNA regulation of prostate cancer signaling pathways WP3981	8.01E-18	9.49E-17	-2.34011
ErbB Signaling Pathway WP673	9.84E-18	1.12E-16	-1.27747
Aryl Hydrocarbon Receptor WP2586	1.5E-17	1.65E-16	-1.90374
Thymic Stromal LymphoPoietin (TSLP) Signaling Pathway WP2203	2.06E-17	2.2E-16	-2.11
Wnt/beta-catenin Signaling Pathway in Leukemia WP3658	2.55E-17	2.64E-16	-2.43499
Nuclear Receptors Meta-Pathway WP2882	3.15E-17	3.15E-16	-0.67084
DNA Damage Response WP707	1.11E-16	1.08E-15	-1.09236
miRNA Regulation of DNA Damage Response WP1530	2.12E-16	1.94E-15	-1.33999
Non-genomic actions of 1,25 dihydroxyvitamin D3 WP4341	2.12E-16	1.94E-15	-0.88709
Vitamin D in inflammatory diseases WP4482	3.05E-16	2.71E-15	-2.36581
Corticotropin-releasing hormone signaling pathway WP2355	4.19E-16	3.62E-15	-1.07055
Integrated Cancer Pathway WP1971	4.43E-16	3.64E-15	-2.03412
Interleukin-11 Signaling Pathway WP2332	4.43E-16	3.64E-15	-2.02649
Kit receptor signaling pathway WP304	5.47E-16	4.38E-15	-1.65463
Prolactin Signaling Pathway WP2037	5.87E-16	4.58E-15	-0.9357
Notch Signaling Pathway WP61	8.77E-16	6.68E-15	-1.14032
Endometrial cancer WP4155	1.38E-15	1.03E-14	-1.7599
Pathways Affected in Adenoid Cystic Carcinoma WP3651	2.14E-15	1.55E-14	-1.42828
Sudden Infant Death Syndrome (SIDS) Susceptibility Pathways WP706	4.65E-15	3.31E-14	-0.58427
Bladder Cancer WP2828	6.97E-15	4.75E-14	-1.95278
ATM Signaling Pathway WP2516	6.97E-15	4.75E-14	-1.91724
Tumor suppressor activity of SMARCB1 WP4204	1.97E-14	1.31E-13	-1.78338

B Cell Receptor Signaling Pathway WP23	2.08E-14	1.36E-13	-0.75042
DNA IR-damage and cellular response via ATR WP4016	3.72E-14	2.38E-13	-0.8831
G1 to S cell cycle control WP45	6.05E-14	3.8E-13	-1.61565
Senescence and Autophagy in Cancer WP615	6.49E-14	3.99E-13	-0.95358
EGF/EGFR Signaling Pathway WP437	1.19E-13	7.2E-13	-0.53637
Wnt Signaling Pathway WP363	1.68E-13	9.98E-13	-1.83598
PI3K-Akt Signaling Pathway WP4172	1.72E-13	9.98E-13	-0.24785
TGF-B Signaling in Thyroid Cells for Epithelial-Mesenchymal Transition WP3859	4E-13	2.29E-12	-2.3592
Epithelial to mesenchymal transition in colorectal cancer WP4239	1.42E-12	7.95E-12	-0.66744
Angiopoietin Like Protein 8 Regulatory Pathway WP3915	1.62E-12	8.96E-12	-0.63819
Mammary gland development pathway - Pregnancy and lactation (Stage 3 of 4) WP2817	1.71E-12	9.11E-12	-1.83891
Ovarian Infertility Genes WP34	1.71E-12	9.11E-12	-1.14995
MicroRNAs in cardiomyocyte hypertrophy WP1544	1.83E-12	9.59E-12	-0.79427
Pregnane X Receptor pathway WP2876	2.33E-12	1.2E-11	-0.98923
Transcription factor regulation in adipogenesis WP3599	2.85E-12	1.45E-11	-1.69832
Photodynamic therapy-induced AP-1 survival signaling. WP3611	4.03E-12	2.02E-11	-1.38946
Estrogen signaling pathway WP712	4.34E-12	2.14E-11	-2.15313
Angiogenesis WP1539	6.47E-12	3.14E-11	-2.14234
IL-7 Signaling Pathway WP205	9.45E-12	4.45E-11	-2.09069
Hypothesized Pathways in Pathogenesis of Cardiovascular Disease WP3668	9.45E-12	4.45E-11	-1.90012
Cardiac Hypertrophic Response WP2795	1.11E-11	5.08E-11	-1.56925
IL-1 signaling pathway WP195	1.11E-11	5.08E-11	-1.35505
IL-5 Signaling Pathway WP127	1.58E-11	7.13E-11	-1.65307
Viral Acute Myocarditis WP4298	4.2E-11	1.87E-10	-0.79993
TP53 Network WP1742	6.23E-11	2.73E-10	-2.19836
IL17 signaling pathway WP2112	6.63E-11	2.87E-10	-1.48489
Association Between Physico-Chemical Features and Toxicity Associated Pathways WP3680	7.46E-11	3.18E-10	-1.36752
Initiation of transcription and translation elongation at the HIV-1 LTR WP3414	8.78E-11	3.65E-10	-1.69924
White fat cell differentiation WP4149	8.78E-11	3.65E-10	-1.56995
ESC Pluripotency Pathways WP3931	9.03E-11	3.7E-10	-0.40437
Structural Pathway of Interleukin 1 (IL-1) WP2637	1.12E-10	4.45E-10	-1.45307
IL-3 Signaling Pathway WP286	1.12E-10	4.45E-10	-1.3729
Resistin as a regulator of inflammation WP4481	1.15E-10	4.45E-10	-1.33785
Prion disease pathway WP3995	1.15E-10	4.45E-10	-1.0271

TNF alpha Signaling Pathway WP231	1.15E-10	4.45E-10	-0.69617
Neovascularisation processes WP4331	1.42E-10	5.41E-10	-1.03572
EBV LMP1 signaling WP262	2.95E-10	1.11E-09	-1.86105
The effect of progerin on the involved genes in Hutchinson-Gilford Progeria Syndrome WP4320	3.12E-10	1.16E-09	-1.17053
Neural Crest Differentiation WP2064	3.22E-10	1.18E-09	-0.48573
DNA IR-Double Strand Breaks (DSBs) and cellular response via ATM WP3959	3.33E-10	1.21E-09	-1.34669
Wnt Signaling Pathway and Pluripotency WP399	3.58E-10	1.29E-09	-0.36542
H19 action Rb-E2F1 signaling and CDK-Beta-catenin activity WP3969	5.19E-10	1.84E-09	-2.2473
MAPK Signaling Pathway WP382	7.16E-10	2.52E-09	-0.08942
EPO Receptor Signaling WP581	7.78E-10	2.71E-09	-1.64486
IL-2 Signaling Pathway WP49	9.24E-10	3.18E-09	-1.45303
MECP2 and Associated Rett Syndrome WP3584	1.02E-09	3.46E-09	-1.16823
IL-6 signaling pathway WP364	1.13E-09	3.8E-09	-1.43158
T-Cell antigen Receptor (TCR) Signaling Pathway WP69	1.73E-09	5.72E-09	-0.4729
Spinal Cord Injury WP2431	1.73E-09	5.72E-09	-0.3165
Human Thyroid Stimulating Hormone (TSH) signaling pathway WP2032	1.81E-09	5.9E-09	-1.2875
Canonical and Non-Canonical TGF-B signaling WP3874	2.1E-09	6.78E-09	-1.7125
Extracellular vesicle-mediated signaling in recipient cells WP2870	2.35E-09	7.51E-09	-1.49038
LncRNA involvement in canonical Wnt signaling and colorectal cancer WP4258	2.66E-09	8.44E-09	-0.6975
Constitutive Androstane Receptor Pathway WP2875	3.83E-09	1.2E-08	-1.32074
Focal Adhesion WP306	4.21E-09	1.31E-08	0.049368
Nuclear Receptors in Lipid Metabolism and Toxicity WP299	4.83E-09	1.49E-08	-0.72825
Copper homeostasis WP3286	5.52E-09	1.68E-08	-1.15765
Hematopoietic Stem Cell Gene Regulation by GABP alpha/beta Complex WP3657	6.44E-09	1.95E-08	-1.99658
Toll-like Receptor Signaling Pathway WP75	6.55E-09	1.96E-08	-0.1142
NAD metabolism, sirtuins and aging WP3630	1.09E-08	3.24E-08	-1.66747
miRNAs involvement in the immune response in sepsis WP4329	1.13E-08	3.33E-08	-1.05244
Endoderm Differentiation WP2853	1.15E-08	3.33E-08	-0.29147
MET in type 1 papillary renal cell carcinoma WP4205	1.56E-08	4.48E-08	-1.08317
Apoptosis WP254	1.6E-08	4.57E-08	-0.31107
TCA Cycle Nutrient Utilization and Invasiveness of Ovarian Cancer WP2868	1.64E-08	4.64E-08	-2.32705
PDGF Pathway WP2526	1.67E-08	4.7E-08	-0.58394

RIG-I-like Receptor Signaling WP3865	1.78E-08	4.96E-08	-0.31872
Ras Signaling WP4223	1.83E-08	5.06E-08	-0.02111
Wnt Signaling WP428	1.91E-08	5.24E-08	-0.14818
ncRNAs involved in Wnt signaling in hepatocellular carcinoma WP4336	1.97E-08	5.35E-08	-0.52055
T-Cell antigen Receptor (TCR) pathway during Staphylococcus aureus infection WP3863	2.33E-08	6.25E-08	-0.85587
Fas Ligand (FasL) pathway and Stress induction of Heat Shock Proteins (HSP) regulation WP314	2.88E-08	7.68E-08	-1.17015
Mammary gland development pathway - Puberty (Stage 2 of 4) WP2814	3.01E-08	7.88E-08	-2.0202
Osteopontin Signaling WP1434	3.01E-08	7.88E-08	-0.73936
Nonalcoholic fatty liver disease WP4396	3.08E-08	8E-08	-0.00155
Vitamin A and Carotenoid Metabolism WP716	3.42E-08	8.82E-08	-0.59524
PPAR Alpha Pathway WP2878	3.69E-08	9.43E-08	-0.52153
Insulin Signaling WP481	4.27E-08	1.08E-07	0.05166
Follicle Stimulating Hormone (FSH) signaling pathway WP2035	4.71E-08	1.19E-07	-1.27684
Chemokine signaling pathway WP3929	5.51E-08	1.38E-07	0.033734
Regulation of Microtubule Cytoskeleton WP2038	5.57E-08	1.38E-07	-0.58563
Ebola Virus Pathway on Host WP4217	5.77E-08	1.42E-07	-0.11217
Nanoparticle-mediated activation of receptor signaling WP2643	5.96E-08	1.45E-07	-0.61686
miRNAs involved in DNA damage response WP1545	6.93E-08	1.68E-07	-1.22587
Focal Adhesion-PI3K-Akt-mTOR-signaling pathway WP3932	9.02E-08	2.17E-07	0.404282
Regulation of toll-like receptor signaling pathway WP1449	1.17E-07	2.79E-07	0.399797
IL-9 Signaling Pathway WP22	1.41E-07	3.34E-07	-1.92703
Thermogenesis WP4321	1.45E-07	3.42E-07	0.142225
Apoptosis-related network due to altered Notch3 in ovarian cancer WP2864	1.53E-07	3.58E-07	-0.96772
Vitamin D Receptor Pathway WP2877	1.59E-07	3.7E-07	0.295795
Signal transduction through IL1R WP4496	1.7E-07	3.91E-07	-0.84583
Transcription co-factors SKI and SKIL protein partners WP4533	1.94E-07	4.43E-07	-0.7189
Caloric restriction and aging WP4191	2.25E-07	5.11E-07	-1.78629
Photodynamic therapy-induced NF-kB survival signaling WP3617	2.45E-07	5.53E-07	-0.17248
Cell Differentiation - Index expanded WP2023	2.62E-07	5.86E-07	-1.21472
BMP Signaling Pathway in Eyelid Development WP3927	3.47E-07	7.71E-07	-1.38166
Supression of HMGB1 mediated inflammation by THBD WP4479	4.03E-07	8.77E-07	-1.3613

NLR Proteins WP288	4.03E-07	8.77E-07	-1.27114
Role Altered Glycolysation of MUC1 in Tumour Microenvironment WP4480	4.03E-07	8.77E-07	-1.20507
Prader-Willi and Angelman Syndrome WP3998	4.12E-07	8.92E-07	0.177824
Endochondral Ossification WP474	5.76E-07	1.24E-06	-0.61292
PI3K/AKT/mTOR - VitD3 Signalling WP4141	5.82E-07	1.24E-06	-0.24783
Heart Development WP1591	1.01E-06	2.14E-06	-0.99933
Notch Signaling WP268	1.16E-06	2.44E-06	-0.25757
Mechanoregulation and pathology of YAP/TAZ via Hippo and non-Hippo mechanisms WP4534	1.51E-06	3.15E-06	-0.8022
MAPK and NFkB Signalling Pathways Inhibited by Yersinia YopJ WP3849	1.55E-06	3.23E-06	-1.86153
Rett syndrome causing genes WP4312	1.71E-06	3.53E-06	-0.43425
Mesodermal Commitment Pathway WP2857	1.99E-06	4.07E-06	-0.03012
Estrogen Receptor Pathway WP2881	2.23E-06	4.55E-06	-1.402
Cannabinoid receptor signaling WP3869	2.51E-06	5.06E-06	-1.14641
PDGFR-beta pathway WP3972	2.51E-06	5.06E-06	-0.13945
Ethanol effects on histone modifications WP3996	3.55E-06	7.01E-06	-1.00051
Factors and pathways affecting insulin-like growth factor (IGF1)-Akt signaling WP3850	3.55E-06	7.01E-06	-0.84625
Toll-like Receptor Signaling WP3858	3.55E-06	7.01E-06	-0.24241
Pathways in clear cell renal cell carcinoma WP4018	4E-06	7.86E-06	0.456269
Mammary gland development pathway - Embryonic development (Stage 1 of 4) WP2813	4.21E-06	8.21E-06	-1.40529
Alpha 6 Beta 4 signaling pathway WP244	4.9E-06	9.5E-06	-0.98329
ID signaling pathway WP53	5.58E-06	1.07E-05	-1.74847
SREBF and miR33 in cholesterol and lipid homeostasis WP2011	5.58E-06	1.07E-05	-0.57047
Signaling of Hepatocyte Growth Factor Receptor WP313	5.71E-06	1.08E-05	-0.55253
Type 2 papillary renal cell carcinoma WP4241	5.71E-06	1.08E-05	-0.03668
Apoptosis Modulation and Signaling WP1772	6.32E-06	1.19E-05	-0.02364
Regulation of Wnt/B-catenin Signaling by Small Molecule Compounds WP3664	7.25E-06	1.36E-05	-0.38314
LncRNA-mediated mechanisms of therapeutic resistance WP3672	8.63E-06	1.6E-05	-2.25491
Somatroph axis (GH) and its relationship to dietary restriction and aging WP4186	8.63E-06	1.6E-05	-1.08507
PPAR signaling pathway WP3942	1.23E-05	2.27E-05	0.346826
Nucleotide-binding Oligomerization Domain (NOD) pathway WP1433	1.47E-05	2.69E-05	-0.08196
Non-homologous end joining WP438	1.5E-05	2.73E-05	-0.48908
Eukaryotic Transcription Initiation WP405	1.87E-05	3.38E-05	-0.70688
Aryl Hydrocarbon Receptor Pathway WP2873	2.61E-05	4.67E-05	-0.45563

NRF2-ARE regulation WP4357	2.6E-05	4.67E-05	-0.40328
Differentiation of white and brown adipocyte WP2895	3.68E-05	6.46E-05	-0.51268
Signal Transduction of S1P Receptor WP26	3.68E-05	6.46E-05	-0.13381
Physiological and Pathological Hypertrophy of the Heart WP1528	3.68E-05	6.46E-05	0.252065
Mammary gland development pathway - Involution (Stage 4 of 4) WP2815	5.06E-05	8.81E-05	-0.99366
Vitamin D Metabolism WP1531	5.06E-05	8.81E-05	-0.35066
Hair Follicle Development: Cytodifferentiation (Part 3 of 3) WP2840	5.51E-05	9.53E-05	-0.05258
Interferon type I signaling pathways WP585	5.74E-05	9.87E-05	-0.65674
Hematopoietic Stem Cell Differentiation WP2849	6.27E-05	0.000107	-0.56289
PI3K-AKT-mTOR signaling pathway and therapeutic opportunities WP3844	7.73E-05	0.000132	0.376088
Gastric Cancer Network 2 WP2363	8.82E-05	0.000149	-0.6988
Bone Morphogenic Protein (BMP) Signalling and Regulation WP1425	9.18E-05	0.000155	-1.19157
Ectoderm Differentiation WP2858	9.38E-05	0.000157	0.512085
Pathways Regulating Hippo Signaling WP4540	0.000107	0.000179	0.900496
BMP2-WNT4-FOXO1 Pathway in Human Primary Endometrial Stromal Cell Differentiation WP3876	0.000119	0.000196	-1.10237
Cell Differentiation - Index WP2029	0.000119	0.000196	-1.02855
Fibrin Complement Receptor 3 Signaling Pathway WP4136	0.000179	0.000293	-0.54982
Oxysterols derived from cholesterol WP4545	0.000187	0.000303	-0.01308
AMP-activated Protein Kinase (AMPK) Signaling WP1403	0.000186	0.000303	0.148091
Amyotrophic lateral sclerosis (ALS) WP2447	0.000199	0.000321	-0.61634
Primary Focal Segmental Glomerulosclerosis FSGS WP2572	0.000227	0.000365	0.889931
Interactome of polycomb repressive complex 2 (PRC2) WP2916	0.000229	0.000366	-1.09758
Amplification and Expansion of Oncogenic Pathways as Metastatic Traits WP3678	0.000276	0.000439	-0.77274
4-hydroxytamoxifen, Dexamethasone, and Retinoic Acids Regulation of p27 Expression WP3879	0.000329	0.000522	-0.01302
Extracellular vesicles in the crosstalk of cardiac cells WP4300	0.000389	0.000607	-0.96757
Farnesoid X Receptor Pathway WP2879	0.000389	0.000607	-0.63753
Serotonin Receptor 4/6/7 and NR3C Signaling WP734	0.000389	0.000607	-0.153
Hereditary leiomyomatosis and renal cell carcinoma pathway WP4206	0.000455	0.000707	-1.05688
Synaptic signaling pathways associated with autism spectrum disorder WP4539	0.000577	0.000892	0.140718

Regulation of Apoptosis by Parathyroid Hormone-related Protein WP3872	0.000608	0.00093	0.036579
Type II diabetes mellitus WP1584	0.000608	0.00093	0.468421
Pathogenic Escherichia coli infection WP2272	0.000829	0.001264	0.68078
Metastatic brain tumor WP2249	0.000855	0.00129	-1.80987
Robo4 and VEGF Signaling Pathways Crosstalk WP3943	0.000855	0.00129	-0.29549
Regulation of Actin Cytoskeleton WP51	0.001052	0.00158	1.046304
MicroRNA for Targeting Cancer Growth and Vascularization in Glioblastoma WP3593	0.001191	0.00178	-1.55558
TLR4 Signaling and Tolerance WP3851	0.00125	0.00186	0.131825
MAPK Cascade WP422	0.001386	0.002025	-0.78037
Lung fibrosis WP3624	0.001382	0.002025	-0.38759
T-Cell Receptor and Co-stimulatory Signaling WP2583	0.001386	0.002025	0.027993
miRs in Muscle Cell Differentiation WP2012	0.001386	0.002025	0.527642
TFs Regulate miRNAs related to cardiac hypertrophy WP1559	0.00158	0.002297	-0.21045
Tgif disruption of Shh signaling WP3674	0.002021	0.002926	0.057214
Liver X Receptor Pathway WP2874	0.002513	0.003623	-1.21403
Wnt Signaling in Kidney Disease WP4150	0.002605	0.003738	0.510947
Melatonin metabolism and effects WP3298	0.002819	0.004009	-0.57243
Photodynamic therapy-induced HIF-1 survival signaling WP3614	0.002819	0.004009	0.780879
PTF1A related regulatory pathway WP4147	0.003056	0.004327	0.313903
Oxidative Damage WP3941	0.003525	0.004969	0.476329
Bile Acids synthesis and enterohepatic circulation WP4389	0.003649	0.005099	-0.95677
Nanoparticle triggered regulated necrosis WP2513	0.003649	0.005099	-0.18365
Alzheimers Disease WP2059	0.003789	0.005271	0.528119
Common Pathways Underlying Drug Addiction WP2636	0.00405	0.005587	-0.59238
DNA Replication WP466	0.00405	0.005587	-0.13529
MFAP5-mediated ovarian cancer cell motility and invasiveness WP3301	0.004291	0.005818	-1.14184
Development of pulmonary dendritic cells and macrophage subsets WP3892	0.004291	0.005818	-0.37586
Transcriptional cascade regulating adipogenesis WP4211	0.004291	0.005818	0.501842
ncRNAs involved in STAT3 signaling in hepatocellular carcinoma WP4337	0.004291	0.005818	0.665378
ATM Signaling Network in Development and Disease WP3878	0.004924	0.006648	-0.00263
ERK Pathway in Huntington's Disease WP3853	0.004981	0.006697	-0.47782
NO/cGMP/PKG mediated Neuroprotection WP4008	0.005564	0.00745	0.038662

Role of Osx and miRNAs in tooth development WP3971	0.005719	0.007593	0.304077
Phytochemical activity on NRF2 transcriptional activation WP3	0.005719	0.007593	0.939596
Ciliary landscape WP4352	0.006423	0.008494	0.786559
NAD+ metabolism WP3644	0.006503	0.008564	-0.62138
Leptin Insulin Overlap WP3935	0.007333	0.009617	-1.06117
Integrin-mediated Cell Adhesion WP185	0.007575	0.009894	0.489038
Cardiac Progenitor Differentiation WP2406	0.007775	0.010114	0.07242
Inhibition of exosome biogenesis and secretion by Manumycin A in CRPC cells WP4301	0.008209	0.010635	-0.85464
Imatinib and Chronic Myeloid Leukemia WP3640	0.010093	0.01297	-0.82429
Serotonin Receptor 2 and ELK-SRF/GATA4 signaling WP732	0.010093	0.01297	0.563135
NAD+ biosynthetic pathways WP3645	0.012149	0.01555	-0.39282
Hippo-Merlin Signaling Dysregulation WP4541	0.013641	0.017391	0.441343
miRNA regulation of p53 pathway in prostate cancer WP3982	0.014371	0.018177	0.888314
Photodynamic therapy-induced NFE2L2 (NRF2) survival signaling WP3612	0.014371	0.018177	1.154977
Sterol Regulatory Element-Binding Proteins (SREBP) signalling WP1982	0.015918	0.020055	1.159492
Canonical and Non-canonical Notch signaling WP3845	0.018006	0.022595	-0.12046
Arrhythmogenic Right Ventricular Cardiomyopathy WP2118	0.01916	0.02395	0.977427
Gastric Cancer Network 1 WP2361	0.020621	0.025676	-0.2796
Fluoropyrimidine Activity WP1601	0.026291	0.032609	-0.02237
p38 MAPK Signaling Pathway WP400	0.027796	0.03421	-0.66592
BDNF-TrkB Signaling WP3676	0.027796	0.03421	0.035882
miR-517 relationship with ARCN1 and USP1 WP3596	0.037673	0.046189	0.821232

Biological processes related to module E2F1

Name	pValue	FDR B&H	Genes from Input	Genes in Annotation
cell cycle	5.18E-116	8.534E-113	135	1766
cell cycle process	6.81E-116	8.534E-113	127	1385
mitotic cell cycle	5.07E-110	4.235E-107	114	1016
mitotic cell cycle process	2.65E-109	1.662E-106	111	931
mitotic cell cycle phase transition	9.107E-76	4.564E-73	76	529
cell cycle phase transition	4.117E-74	1.72E-71	76	555

nuclear division	1.24E-62	4.439E-60	70	599
organelle fission	8.912E-61	2.792E-58	70	636
cell division	2.896E-59	8.064E-57	70	668
chromosome segregation	2.808E-58	7.036E-56	56	333
regulation of cell cycle	9.771E-57	2.226E-54	78	1008
sister chromatid segregation	6.855E-55	1.432E-52	48	225
nuclear chromosome segregation	1.99E-54	3.836E-52	51	283
chromosome organization	1.081E-53	1.935E-51	80	1189
regulation of cell cycle process	6.949E-53	1.161E-50	63	598
G1/S transition of mitotic cell cycle	3.591E-51	5.624E-49	47	247
cell cycle G1/S phase transition	2.658E-50	3.918E-48	47	257
regulation of mitotic cell cycle	1.161E-43	1.616E-41	53	508
regulation of mitotic cell cycle phase transition	2.17E-41	2.862E-39	44	318
regulation of cell cycle phase transition	3.419E-40	4.284E-38	44	338
mitotic sister chromatid segregation	1.697E-39	2.025E-37	33	136
DNA metabolic process	2.555E-39	2.911E-37	63	992
DNA replication	2.289E-38	2.494E-36	43	346
cell cycle checkpoint	6.756E-37	7.054E-35	37	234
microtubule cytoskeleton organization	4.129E-34	4.139E-32	44	464
negative regulation of cell cycle	2.563E-33	2.47E-31	44	484
positive regulation of cell cycle process	2.053E-32	1.906E-30	35	259
sister chromatid cohesion	2.966E-32	2.654E-30	28	127
negative regulation of cell cycle process	3.36E-32	2.903E-30	34	240
negative regulation of mitotic cell cycle	9.146E-32	7.64E-30	33	225
DNA-dependent DNA replication	1.524E-31	1.232E-29	28	134
cellular response to DNA damage stimulus	2.585E-31	2.024E-29	51	800
microtubule-based process	6.411E-31	4.868E-29	47	658
mitotic cell cycle checkpoint	1.444E-30	1.064E-28	29	162
positive regulation of cell cycle	3.315E-30	2.374E-28	37	352
negative regulation of mitotic cell cycle phase transition	2.579E-29	1.793E-27	28	159
regulation of cell division	2.648E-29	1.793E-27	34	291
DNA replication initiation	6.037E-29	3.981E-27	18	35
cell cycle G2/M phase transition	7.321E-29	4.704E-27	30	205
negative regulation of cell cycle phase transition	1.854E-28	1.162E-26	28	170
G2/M transition of mitotic cell cycle	5.479E-28	3.349E-26	29	197
regulation of chromosome segregation	6.302E-27	3.76E-25	22	87
regulation of nuclear division	1.605E-26	9.352E-25	27	177
regulation of mitotic nuclear division	1.113E-25	6.34E-24	25	149
DNA integrity checkpoint	3.375E-23	1.879E-21	24	164
regulation of sister chromatid segregation	1.342E-22	7.313E-21	18	68
DNA conformation change	9.879E-20	5.267E-18	26	285

regulation of mitotic sister chromatid separation	1.212E-19	6.329E-18	15	52
mitotic sister chromatid separation	2.308E-19	1.18E-17	15	54
regulation of cell cycle G2/M phase transition	2.709E-19	1.358E-17	16	68
chromosome separation	5.778E-19	2.839E-17	16	71
regulation of mitotic sister chromatid segregation	7.711E-19	3.716E-17	15	58
cytoskeleton organization	1.211E-18	5.724E-17	45	1164
regulation of mitotic metaphase/anaphase transition	2.426E-18	1.126E-16	14	49
mitotic spindle organization	2.962E-18	1.35E-16	17	95
regulation of metaphase/anaphase transition of cell cycle	3.346E-18	1.497E-16	14	50
DNA repair	4.207E-18	1.85E-16	31	516
metaphase/anaphase transition of mitotic cell cycle	4.579E-18	1.979E-16	14	51
metaphase/anaphase transition of cell cycle	6.222E-18	2.643E-16	14	52
regulation of microtubule cytoskeleton organization	9.784E-18	4.086E-16	19	143
regulation of organelle organization	1.529E-17	6.28E-16	44	1186
regulation of microtubule-based process	1.682E-17	6.799E-16	20	171
spindle organization	1.897E-17	7.544E-16	19	148
mitotic DNA integrity checkpoint	2.462E-17	9.64E-16	17	107
DNA damage checkpoint	4.063E-17	1.566E-15	19	154
regulation of G2/M transition of mitotic cell cycle	7.425E-17	2.819E-15	14	61
regulation of G1/S transition of mitotic cell cycle	2.89E-16	1.081E-14	18	146
regulation of chromosome organization	7.605E-16	2.803E-14	23	301
regulation of cell cycle G1/S phase transition	9.542E-16	3.418E-14	18	156
positive regulation of mitotic cell cycle	9.547E-16	3.418E-14	17	132
negative regulation of cell division	1.673E-15	5.904E-14	14	75
spindle checkpoint	1.762E-15	6.134E-14	12	45
positive regulation of mitotic cell cycle phase transition	2.796E-15	9.6E-14	13	61
protein-DNA complex assembly	3.706E-15	1.255E-13	20	225
microtubule organizing center organization	4.522E-15	1.511E-13	16	121
negative regulation of mitotic sister chromatid separation	5.004E-15	1.65E-13	11	36
establishment of chromosome localization	5.476E-15	1.765E-13	13	64
negative regulation of mitotic nuclear division	5.493E-15	1.765E-13	12	49
attachment of spindle microtubules to kinetochore	6.017E-15	1.909E-13	10	26
signal transduction by p53 class mediator	6.283E-15	1.968E-13	21	263
chromosome localization	6.796E-15	2.102E-13	13	65
regulation of transcription involved in G1/S transition of mitotic cell cycle	9.489E-15	2.9E-13	10	27
negative regulation of mitotic sister chromatid segregation	9.88E-15	2.983E-13	11	38
meiotic cell cycle	1.007E-14	3.006E-13	20	237

positive regulation of cell cycle phase transition	1.035E-14	3.052E-13	13	67
regulation of cell cycle arrest	1.588E-14	4.628E-13	15	108
cell cycle arrest	2.221E-14	6.397E-13	20	247
negative regulation of sister chromatid segregation	2.539E-14	7.231E-13	11	41
protein-DNA complex subunit organization	3.253E-14	9.16E-13	20	252
regulation of cyclin-dependent protein serine/threonine kinase activity	4.539E-14	1.255E-12	14	94
negative regulation of chromosome segregation	4.557E-14	1.255E-12	11	43
regulation of transferase activity	7.439E-14	2.026E-12	36	1006
metaphase plate congression	7.927E-14	2.136E-12	11	45
negative regulation of G1/S transition of mitotic cell cycle	8.257E-14	2.201E-12	14	98
negative regulation of nuclear division	9.517E-14	2.493E-12	12	61
regulation of cyclin-dependent protein kinase activity	9.549E-14	2.493E-12	14	99
mitotic DNA damage checkpoint	1.271E-13	3.249E-12	14	101
negative regulation of cell cycle G1/S phase transition	1.271E-13	3.249E-12	14	101
DNA packaging	1.288E-13	3.26E-12	18	206
DNA biosynthetic process	1.95E-13	4.791E-12	18	211
negative regulation of mitotic metaphase/anaphase transition	1.95E-13	4.791E-12	10	35
mitotic spindle checkpoint	1.95E-13	4.791E-12	10	35
proteasome-mediated ubiquitin-dependent protein catabolic process	2.271E-13	5.527E-12	23	393
negative regulation of metaphase/anaphase transition of cell cycle	2.681E-13	6.46E-12	10	36
mitotic metaphase plate congression	3.647E-13	8.705E-12	10	37
regulation of DNA metabolic process	5.155E-13	1.219E-11	22	368
meiotic cell cycle process	6.633E-13	1.553E-11	17	195
double-strand break repair via homologous recombination	8.789E-13	2.039E-11	13	93
mitotic G1/S transition checkpoint	9.216E-13	2.1E-11	12	73
mitotic G1 DNA damage checkpoint	9.216E-13	2.1E-11	12	73
proteasomal protein catabolic process	9.989E-13	2.255E-11	23	422
recombinational repair	1.013E-12	2.266E-11	13	94
DNA recombination	1.049E-12	2.327E-11	19	267
G1 DNA damage checkpoint	1.092E-12	2.401E-11	12	74
cytokinesis	1.325E-12	2.887E-11	15	145
mitotic spindle assembly checkpoint	2.772E-12	5.988E-11	9	31
centrosome cycle	2.872E-12	6.152E-11	12	80
spindle assembly checkpoint	3.829E-12	8.132E-11	9	32
protein-containing complex assembly	4.007E-12	8.439E-11	46	1825
positive regulation of cell cycle arrest	6.052E-12	1.264E-10	12	85

DNA damage response, signal transduction by p53 class mediator	6.262E-12	1.297E-10	13	108
double-strand break repair	8.949E-12	1.838E-10	16	196
regulation of proteolysis involved in cellular protein catabolic process	1.064E-11	2.168E-10	18	267
positive regulation of cell division	1.22E-11	2.465E-10	14	140
mitotic cytokinesis	1.249E-11	2.504E-10	9	36
centromere complex assembly	1.821E-11	3.622E-10	10	53
signal transduction in response to DNA damage	3.021E-11	5.961E-10	13	122
DNA synthesis involved in DNA repair	3.085E-11	6.04E-10	11	75
regulation of cellular protein catabolic process	3.533E-11	6.862E-10	18	287
regulation of proteasomal ubiquitin-dependent protein catabolic process	4.462E-11	8.601E-10	14	154
microtubule cytoskeleton organization involved in mitosis	4.702E-11	8.926E-10	10	58
mitotic spindle assembly	4.702E-11	8.926E-10	10	58
negative regulation of proteolysis involved in cellular protein catabolic process	4.795E-11	9.035E-10	11	78
DNA unwinding involved in DNA replication	6.408E-11	1.198E-09	6	10
negative regulation of proteasomal ubiquitin-dependent protein catabolic process	9.415E-11	1.735E-09	10	62
DNA damage response, signal transduction by p53 class mediator resulting in cell cycle arrest	9.415E-11	1.735E-09	10	62
negative regulation of cellular protein catabolic process	1.097E-10	1.989E-09	11	84
cytoskeleton-dependent cytokinesis	1.103E-10	1.989E-09	9	45
signal transduction involved in mitotic G1 DNA damage checkpoint	1.111E-10	1.989E-09	10	63
intracellular signal transduction involved in G1 DNA damage checkpoint	1.111E-10	1.989E-09	10	63
signal transduction involved in mitotic DNA integrity checkpoint	1.308E-10	2.291E-09	10	64
signal transduction involved in mitotic cell cycle checkpoint	1.308E-10	2.291E-09	10	64
signal transduction involved in mitotic DNA damage checkpoint	1.308E-10	2.291E-09	10	64
ubiquitin-dependent protein catabolic process	1.42E-10	2.472E-09	24	590
signal transduction involved in DNA integrity checkpoint	1.795E-10	3.082E-09	10	66
signal transduction involved in DNA damage checkpoint	1.795E-10	3.082E-09	10	66
modification-dependent protein catabolic process	1.868E-10	3.184E-09	24	598
signal transduction involved in cell cycle checkpoint	2.095E-10	3.548E-09	10	67
modification-dependent macromolecule catabolic process	2.445E-10	4.112E-09	24	606

regulation of attachment of spindle microtubules to kinetochore	2.781E-10	4.646E-09	6	12
negative regulation of chromosome organization	2.802E-10	4.651E-09	12	117
negative regulation of proteasomal protein catabolic process	2.832E-10	4.669E-09	10	69
spindle assembly	3.375E-10	5.528E-09	11	93
regulation of kinase activity	3.654E-10	5.946E-09	28	842
regulation of proteasomal protein catabolic process	6.855E-10	1.108E-08	14	189
DNA duplex unwinding	7.562E-10	1.215E-08	10	76
G2 DNA damage checkpoint	7.999E-10	1.277E-08	8	38
regulation of protein kinase activity	1.411E-09	2.238E-08	26	775
proteolysis involved in cellular protein catabolic process	1.434E-09	2.249E-08	24	662
DNA geometric change	1.436E-09	2.249E-08	10	81
reproductive process	1.738E-09	2.705E-08	37	1503
reproduction	1.802E-09	2.788E-08	37	1505
regulation of protein catabolic process	1.951E-09	2.999E-08	19	414
cellular protein catabolic process	3.627E-09	5.542E-08	24	694
chromatin organization	5.882E-09	8.933E-08	25	770
protein phosphorylation	5.942E-09	8.97E-08	42	1952
regulation of double-strand break repair via homologous recombination	7.779E-09	1.167E-07	6	19
positive regulation of transferase activity	7.947E-09	1.185E-07	23	665
regulation of cytoskeleton organization	8.185E-09	1.214E-07	19	452
organelle localization	1.037E-08	1.528E-07	20	510
positive regulation of chromosome segregation	1.104E-08	1.617E-07	6	20
regulation of protein modification process	1.169E-08	1.703E-07	40	1841
negative regulation of protein catabolic process	1.338E-08	1.938E-07	11	131
chromosome condensation	1.396E-08	2.011E-07	7	35
anaphase-promoting complex-dependent catabolic process	1.598E-08	2.289E-07	9	77
negative regulation of organelle organization	2.49E-08	3.545E-07	16	337
regulation of double-strand break repair	2.566E-08	3.633E-07	7	38
establishment of organelle localization	2.94E-08	4.139E-07	18	438
protein catabolic process	3.595E-08	5.033E-07	25	844
positive regulation of proteolysis involved in cellular protein catabolic process	3.864E-08	5.379E-07	12	180
protein localization to chromosome	4.093E-08	5.667E-07	8	61
protein localization to chromosome, centromeric region	4.715E-08	6.493E-07	5	13
positive regulation of protein catabolic process	5.993E-08	8.207E-07	14	268
strand displacement	6.289E-08	8.565E-07	6	26
regulation of ubiquitin-protein transferase activity	6.714E-08	9.095E-07	10	120

regulation of centrosome cycle	7.471E-08	0.000001007	7	44
positive regulation of nuclear division	7.705E-08	0.000001033	8	66
regulation of phosphorylation	8.283E-08	0.000001104	34	1510
regulation of ligase activity	8.494E-08	0.000001126	10	123
mitotic recombination	8.785E-08	0.000001152	7	45
regulation of DNA-dependent DNA replication	8.785E-08	0.000001152	7	45
positive regulation of cellular protein catabolic process	8.826E-08	0.000001152	12	194
microtubule-based movement	9.338E-08	0.000001212	13	235
chromatin remodeling at centromere	1.029E-07	0.000001329	7	46
chromatin assembly	1.206E-07	0.00000155	11	162
chromatin remodeling	1.366E-07	0.000001747	11	164
positive regulation of DNA metabolic process	1.448E-07	0.000001842	12	203
regulation of exit from mitosis	1.568E-07	0.000001975	5	16
DNA replication checkpoint	1.568E-07	0.000001975	5	16
negative regulation of G2/M transition of mitotic cell cycle	1.578E-07	0.000001977	6	30

Pathways related to module E2F1

Term	P-value	Adj. P-value	Z-score
G1 to S cell cycle control WP45	3.94E-47	1.8E-45	-1.95642
Retinoblastoma Gene in Cancer WP2446	5.59E-48	3.83E-46	-1.74017
DNA Replication WP466	2.4E-38	8.22E-37	-2.09373
Cell Cycle WP179	19.79E-32	2.71E-61	-1.15315
miRNA Regulation of DNA Damage Response WP1530	1.77E-24	4.85E-23	-1.72269
DNA Damage Response WP707	3.81E-23	8.69E-22	-1.32103
Gastric Cancer Network 1 WP2361	1.39E-16	2.39E-15	-1.83397
DNA IR-damage and cellular response via ATR WP4016	9.81E-22	1.92E-20	-1.27862
miRNAs involved in DNA damage response WP1545	7.63E-08	6.54E-07	-3.38894
ATM Signaling Pathway WP2516	1.88E-11	2.87E-10	-2.23923
Integrated Cancer Pathway WP1971	4.76E-11	6.52E-10	-2.23108
Regulation of sister chromatid separation at the metaphase-anaphase transition WP4240	9.66E-10	1.1E-08	-2.3819
H19 action Rb-E2F1 signaling and CDK-Beta-catenin activity WP3969	5.12E-08	4.68E-07	-2.84809
ID signaling pathway WP53	1.1E-07	8.89E-07	-2.87544
DNA IR-Double Strand Breaks (DSBs) and cellular response via ATM WP3959	1.02E-08	9.99E-08	-2.07864
Signaling Pathways in Glioblastoma WP2261	8.24E-10	1.03E-08	-1.59912
DNA Mismatch Repair WP531	3.78E-05	0.000185	-2.89841

Gastric Cancer Network 2 WP2363	1.28E-07	9.75E-07	-1.73472
Tumor suppressor activity of SMARCB1 WP4204	3.91E-06	2.43E-05	-2.13364
Human Thyroid Stimulating Hormone (TSH) signaling pathway WP2032	8.13E-07	5.3E-06	-1.74211
Bladder Cancer WP2828	1.43E-05	7.53E-05	-2.12744
Prader-Willi and Angelman Syndrome WP3998	4.7E-07	3.22E-06	-1.59626
Wnt Signaling Pathway WP363	5.24E-05	0.000231	-2.18808
Photodynamic therapy-induced AP-1 survival signaling. WP3611	4.32E-05	0.000197	-1.95428
Integrated Breast Cancer Pathway WP1984	2.44E-09	2.57E-08	-0.91326
ATR Signaling WP3875	0.002099	0.005981	-2.88868
Pancreatic adenocarcinoma pathway WP4263	6.19E-06	3.69E-05	-1.40291
Non-small cell lung cancer WP4255	1.26E-05	6.92E-05	-1.38824
Wnt/beta-catenin Signaling Pathway in Leukemia WP3658	0.001061	0.00338	-2.25473
Regulation of Wnt/B-catenin Signaling by Small Molecule Compounds WP3664	0.000292	0.001143	-1.89391
Aryl Hydrocarbon Receptor WP2586	0.00045	0.001668	-1.83167
Apoptosis-related network due to altered Notch3 in ovarian cancer WP2864	0.000775	0.002589	-1.81971
Extracellular vesicle-mediated signaling in recipient cells WP2870	0.001619	0.004822	-1.99363
Hepatitis C and Hepatocellular Carcinoma WP3646	0.000574	0.002017	-1.69736
ATM Signaling Network in Development and Disease WP3878	0.000414	0.001574	-1.60388
Breast cancer pathway WP4262	3.66E-07	2.64E-06	-0.83277
miRNA regulation of prostate cancer signaling pathways WP3981	0.002139	0.005981	-1.97921
Homologous recombination WP186	0.004457	0.011972	-2.24022
Spinal Cord Injury WP2431	3.92E-05	0.000185	-1.13298
Endometrial cancer WP4155	0.001484	0.004517	-1.74127
Ciliary landscape WP4352	7.8E-06	4.45E-05	-0.95319
LncRNA involvement in canonical Wnt signaling and colorectal cancer WP4258	9.46E-05	0.000393	-1.14694
Association Between Physico-Chemical Features and Toxicity Associated Pathways WP3680	0.001763	0.00514	-1.61192
DNA Damage Response (only ATM dependent) WP710	2.49E-05	0.000127	-0.95617
Metastatic brain tumor WP2249	0.045902	0.088572	-3.28654
Imatinib and Chronic Myeloid Leukemia WP3640	0.010475	0.025626	-2.1871
PPAR Alpha Pathway WP2878	0.001061	0.00338	-1.32492
Chromosomal and microsatellite instability in colorectal cancer WP4216	0.000265	0.001069	-1.07313
IL-7 Signaling Pathway WP205	0.016124	0.036213	-2.13899
TGF-beta Signaling Pathway WP366	8.01E-05	0.000343	-0.93161
Regulation of Microtubule Cytoskeleton WP2038	0.005528	0.014024	-1.48329

ncRNAs involved in Wnt signaling in hepatocellular carcinoma WP4336	0.000567	0.002017	-1.03027
Androgen receptor signaling pathway WP138	0.000698	0.002391	-1.02597
Monoamine Transport WP727	0.02573	0.054231	-1.72221
MECP2 and Associated Rett Syndrome WP3584	0.012582	0.029806	-1.42398
Wnt Signaling Pathway and Pluripotency WP399	0.001228	0.003823	-0.87218
Mammary gland development pathway - Puberty (Stage 2 of 4) WP2814	0.096815	0.163749	-2.35963
BMP2-WNT4-FOXO1 Pathway in Human Primary Endometrial Stromal Cell Differentiation WP3876	0.096815	0.163749	-2.26591
Fas Ligand (FasL) pathway and Stress induction of Heat Shock Proteins (HSP) regulation WP314	0.04247	0.083121	-1.63055
Mammary gland development pathway - Involution (Stage 4 of 4) WP2815	0.075335	0.137611	-1.99158
The effect of progerin on the involved genes in Hutchinson-Gilford Progeria Syndrome WP4320	0.033688	0.066888	-1.3696
Extracellular vesicles in the crosstalk of cardiac cells WP4300	0.1383	0.212616	-2.32261
Mammary gland development pathway - Embryonic development (Stage 1 of 4) WP2813	0.110859	0.182985	-2.04964
Senescence and Autophagy in Cancer WP615	0.00926	0.023066	-0.91906
Interactome of polycomb repressive complex 2 (PRC2) WP2916	0.1178	0.192126	-1.93099
Endoderm Differentiation WP2853	0.005004	0.013183	-0.7774
Ovarian Infertility Genes WP34	0.02573	0.054231	-1.10234
Copper homeostasis WP3286	0.062215	0.117122	-1.44121
AMP-activated Protein Kinase (AMPK) Signaling WP1403	0.016757	0.037028	-0.97432
Role of Osx and miRNAs in tooth development WP3971	0.110859	0.182985	-1.79301
Adipogenesis WP236	0.003546	0.009717	-0.68844
TP53 Network WP1742	0.1383	0.212616	-1.96252
Wnt Signaling WP428	0.012619	0.029806	-0.8459
Photodynamic therapy-induced NF-kB survival signaling WP3617	0.030402	0.06125	-1.05821
Cell Differentiation - Index expanded WP2023	0.1383	0.212616	-1.67817
Primary Focal Segmental Glomerulosclerosis FSGS WP2572	0.018756	0.040787	-0.74231
RAC1/PAK1/p38/MMP2 Pathway WP3303	0.016119	0.036213	-0.70315
PTF1A related regulatory pathway WP4147	0.082551	0.148809	-1.15568
Vitamin D Receptor Pathway WP2877	0.014159	0.032877	-0.63668
Apoptosis WP254	0.028019	0.04767	-0.75476
Genotoxicity pathway WP4286	0.08676	0.154364	-0.99205
Mesodermal Commitment Pathway WP2857	0.028204	0.05767	-0.66679
Mammary gland development pathway - Pregnancy and lactation (Stage 3 of 4) WP2817	0.221802	0.31007	-1.56374
Oncostatin M Signaling Pathway WP2374	0.091492	0.158663	-0.96842
Signal Transduction of S1P Receptor WP26	0.177891	0.262055	-1.27888

Pathways Affected in Adenoid Cystic Carcinoma WP3651	0.091492	0.158663	-0.88971
miRs in Muscle Cell Differentiation WP2012	0.203276	0.293145	-1.24171
Fluoropyrimidine Activity WP1601	0.227881	0.315351	-1.32549
Nucleotide Metabolism WP404	0.1383	0.212616	-0.95993
Interleukin-11 Signaling Pathway WP2332	0.291721	0.364314	-1.51692
Hippo-Merlin Signaling Dysregulation WP4541	0.067644	0.125232	-0.66155
Eukaryotic Transcription Initiation WP405	0.286141	0.364314	-1.33065
TNF related weak inducer of apoptosis (TWEAK) Signaling Pathway WP2036	0.280518	0.364314	-1.25422
Wnt Signaling in Kidney Disease WP4150	0.245839	0.333464	-1.12688
Heart Development WP1591	0.291721	0.364314	-1.2702
PI3K-AKT-mTOR signaling pathway and therapeutic opportunities WP3844	0.209499	0.295891	-0.90427
PI3K-Akt Signaling Pathway WP4172	0.005302	0.013706	-0.26886
Apoptosis Modulation and Signaling WP1772	0.15858	0.236146	-0.71798
Hedgehog Signaling Pathway WP4249	0.291721	0.364314	-1.0632
ESC Pluripotency Pathways WP3931	0.062408	0.117122	-0.45374
Mechanoregulation and pathology of YAP/TAZ via Hippo and non-Hippo mechanisms WP4534	0.308203	0.375577	-1.06493
Aryl Hydrocarbon Receptor Pathway WP2873	0.302752	0.373667	-1.04692
Cardiac Hypertrophic Response WP2795	0.350314	0.406721	-1.18498
IL-4 Signaling Pathway WP395	0.345193	0.406721	-1.16322
ErbB Signaling Pathway WP673	0.15858	0.236146	-0.65555
MicroRNAs in cardiomyocyte hypertrophy WP1544	0.139675	0.212616	-0.58067
TGF-beta Receptor Signaling WP560	0.345193	0.406721	-1.05096
Oxidative Damage WP3941	0.269138	0.354537	-0.73044
Parkinsons Disease Pathway WP2371	0.257579	0.342605	-0.66964
Epithelial to mesenchymal transition in colorectal cancer WP4239	0.127996	0.2063	-0.42931
Photodynamic therapy-induced HIF-1 survival signaling WP3614	0.251732	0.33811	-0.6318
T-Cell antigen Receptor (TCR) pathway during Staphylococcus aureus infection WP3863	0.385066	0.435984	-0.83159
Neural Crest Differentiation WP2064	0.186327	0.271562	-0.43461
Histone Modifications WP2369	0.408752	0.459008	-0.66818
Notch Signaling Pathway WP61	0.380217	0.434081	-0.42853
Pathogenic Escherichia coli infection WP2272	0.350314	0.406721	-0.35961
Hair Follicle Development: Cytodifferentiation (Part 3 of 3) WP2840	0.494767	0.52545	-0.4139
Ectoderm Differentiation WP2858	0.292515	0.364314	-0.2306
Leptin signaling pathway WP2034	0.449125	0.492241	-0.25343
Arrhythmogenic Right Ventricular Cardiomyopathy WP2118	0.440397	0.486567	-0.19719
Circadian rhythm related genes WP3594	0.207344	0.295891	-0.09198

Parkin-Ubiquitin Proteasomal System pathway WP2359	0.422526	0.470618	-0.16721
Corticotropin-releasing hormone signaling pathway WP2355	0.518058	0.541785	-0.2125
Nuclear Receptors Meta-Pathway WP2882	0.238573	0.326845	-0.09253
Viral Acute Myocarditis WP4298	0.482705	0.520714	-0.06568
TNF alpha Signaling Pathway WP231	0.514251	0.541785	-0.0586
Ebola Virus Pathway on Host WP4217	0.637019	0.65128	-0.07712
Pyrimidine metabolism WP4022	0.486757	0.520982	-0.04053
Brain-Derived Neurotrophic Factor (BDNF) signaling pathway WP2380	0.309783	0.375577	-0.00572
Focal Adhesion-PI3K-Akt-mTOR-signaling pathway WP3932	0.908447	0.908447	0.443174
EGF/EGFR Signaling Pathway WP437	0.360968	0.415568	0.08187
Regulation of Actin Cytoskeleton WP51	0.692414	0.697506	0.26548
Pathways Regulating Hippo Signaling WP4540	0.536649	0.556977	0.219076
Regulation of toll-like receptor signaling pathway WP1449	0.664535	0.67438	0.412587
Focal Adhesion WP306	0.45851	0.498538	0.269419
Sudden Infant Death Syndrome (SIDS) Susceptibility Pathways WP706	0.349697	0.406721	0.235717
VEGFA-VEGFR2 Signaling Pathway WP3888	0.551604	0.568194	0.771737

Differentially expressed genes inside detected modules

Gene	DEG type	LogFC	adj-pValue
PGR	Down-Regulated	-1.288619423	0.000139653
ID2	Down-Regulated	-1.836407727	8.98E-19
NCOR1	Down-Regulated	-1.084189195	2.53E-11
SATB1	Down-Regulated	-1.68716642	2.74E-13
SMAD6	Down-Regulated	-1.072472542	1.51E-08
LCAT	Down-Regulated	-3.518705534	3.36E-21
BMPR1B	Down-Regulated	-3.324356581	1.51E-12
DACH1	Down-Regulated	-2.941596682	1.86E-22
CYP27A1	Down-Regulated	-1.431140141	9.61E-08
CYP3A7	Down-Regulated	-2.496712954	1.06E-07
RBP1	Down-Regulated	-1.768003079	2.35E-06
RBL1	Up-Regulated	1.014366371	5.67E-08
DNMT3A	Up-Regulated	1.004744659	8.14E-09
CCNE1	Up-Regulated	2.485713795	4.60E-12
MCM5	Up-Regulated	1.179651652	8.91E-10
MCM3	Up-Regulated	1.1829497	7.75E-13
MYBL2	Up-Regulated	3.366162287	4.71E-15
LPL	Up-Regulated	2.564688298	2.30E-11

**KIF4A**	Up-Regulated	3.46022583	2.16E-19
**MCM4**	Up-Regulated	1.254759224	5.22E-11
**CDC6**	Up-Regulated	2.823756142	3.31E-16
**CDC45**	Up-Regulated	3.023467931	9.99E-18
**MSX1**	Up-Regulated	1.702071201	4.09E-09
**FANCD2**	Up-Regulated	1.835386688	2.46E-12
**MCM10**	Up-Regulated	2.811145539	1.09E-14
**KIAA1524**	Up-Regulated	1.658207654	1.37E-08
**TACC3**	Up-Regulated	1.600147466	3.71E-14
**RRM2**	Up-Regulated	2.958730291	3.58E-17
**CLSPN**	Up-Regulated	2.744472028	5.54E-14
**NEK2**	Up-Regulated	3.794772824	2.94E-17
**PITX1**	Up-Regulated	4.116529206	1.51E-08
**RAD54L**	Up-Regulated	2.975783196	3.17E-15
**MAD2L1**	Up-Regulated	1.903569312	2.74E-12
**MCM6**	Up-Regulated	1.566041172	4.18E-14
**TRIP13**	Up-Regulated	2.720090194	1.50E-17
**CENPM**	Up-Regulated	3.005053878	1.70E-16
**CDCA5**	Up-Regulated	2.982161002	4.98E-20
**SGOL1**	Up-Regulated	2.549120805	1.39E-13
**KIF14**	Up-Regulated	2.856494403	6.88E-15
**RFC4**	Up-Regulated	1.338716444	1.10E-13
**CDC20**	Up-Regulated	3.698680682	1.28E-18
**CDC7**	Up-Regulated	1.566401659	9.61E-09
**ASF1B**	Up-Regulated	2.355889148	2.72E-14
**CENPF**	Up-Regulated	3.396339807	2.85E-18
**KIF20A**	Up-Regulated	3.544024625	7.47E-19
**WDR62**	Up-Regulated	2.043077932	6.08E-17
**KIFC1**	Up-Regulated	3.162279207	6.56E-19
**ANLN**	Up-Regulated	3.532045905	3.62E-17
**ORC1**	Up-Regulated	2.767666338	3.39E-15
**MKI67**	Up-Regulated	3.207312944	6.28E-18
**RASD2**	Up-Regulated	2.827344587	2.91E-21
**BUB1B**	Up-Regulated	3.238297613	7.41E-17
**NDC80**	Up-Regulated	2.841439877	1.09E-17
**PKMYT1**	Up-Regulated	2.750215929	2.16E-18
**RASL12**	Up-Regulated	2.211393022	5.53E-18
**UBE2C**	Up-Regulated	3.575832895	3.13E-20
**GINS1**	Up-Regulated	2.258573549	3.09E-13
**KIF18A**	Up-Regulated	2.957636763	8.44E-17
**FANCI**	Up-Regulated	1.927119177	9.01E-16

**CCNB2**	Up-Regulated	3.327590987	3.13E-18
**KIF11**	Up-Regulated	1.774523124	1.29E-12
**EXO1**	Up-Regulated	3.318159572	2.31E-18
**MELK**	Up-Regulated	3.779165688	6.50E-20
**CENPE**	Up-Regulated	2.794047142	2.33E-16
**WDR76**	Up-Regulated	1.82012816	1.80E-11
**E2F8**	Up-Regulated	3.418611918	9.28E-14
**RACGAP1**	Up-Regulated	2.110779789	1.87E-17
**CENPK**	Up-Regulated	2.286001876	3.11E-12
**CHAF1B**	Up-Regulated	1.974420739	1.54E-11
**LIN9**	Up-Regulated	1.259847049	1.04E-09
**KIAA0101**	Up-Regulated	1.902506584	5.24E-12
**STIL**	Up-Regulated	2.081514911	3.15E-14
**GTSE1**	Up-Regulated	2.920017462	2.33E-16
**NUSAP1**	Up-Regulated	1.732518214	2.14E-10
**CDCA2**	Up-Regulated	2.740626794	5.20E-13
**KNTC1**	Up-Regulated	1.401213122	8.83E-09
**WDHD1**	Up-Regulated	1.657857458	4.34E-13
**DEPDC1B**	Up-Regulated	2.279392965	7.25E-09
**CENPA**	Up-Regulated	3.500461762	7.00E-21
**KIF23**	Up-Regulated	2.889605545	1.22E-15
**SHCBP1**	Up-Regulated	2.853512474	1.31E-13
**CDCA8**	Up-Regulated	2.84696446	1.03E-19
**DLGAP5**	Up-Regulated	3.306022236	1.71E-18
**ECT2**	Up-Regulated	2.140418248	7.96E-14
**CEP55**	Up-Regulated	2.450846554	2.30E-14
**SGOL2**	Up-Regulated	2.131104064	2.87E-14
**CCNF**	Up-Regulated	1.818340643	1.10E-14
**CDCA3**	Up-Regulated	2.653297249	1.99E-19
**KIF15**	Up-Regulated	2.716216445	1.08E-13
**HJURP**	Up-Regulated	3.550946039	1.55E-18
**NUF2**	Up-Regulated	3.538153644	8.98E-19
**KIF18B**	Up-Regulated	3.450941656	5.34E-18
**DEPDC1**	Up-Regulated	3.821788099	1.61E-16
**TCF19**	Up-Regulated	2.364587011	7.40E-15
**C17orf53**	Up-Regulated	1.262210124	1.57E-08
**PARPBP**	Up-Regulated	2.241293469	1.31E-13
**CDT1**	Up-Regulated	2.984575601	5.45E-17
**NCAPG**	Up-Regulated	3.647949073	4.01E-18
**CENPI**	Up-Regulated	2.670425519	2.30E-14
**CENPL**	Up-Regulated	2.014698742	4.53E-17

**TTK**	Up-Regulated	3.3000251	3.82E-16
**ASPM**	Up-Regulated	3.546057109	1.05E-16
**TROAP**	Up-Regulated	3.538567538	2.11E-20
**RAD51AP1**	Up-Regulated	1.957861143	1.39E-11
**HELLS**	Up-Regulated	1.760709041	1.08E-09
**UBE2T**	Up-Regulated	2.968487787	6.16E-20
**SKA3**	Up-Regulated	3.166741929	3.26E-17
**SKA1**	Up-Regulated	3.979793771	1.46E-19
**POLQ**	Up-Regulated	2.627697259	1.05E-13
**PRR11**	Up-Regulated	1.984129659	5.77E-13
**ZWINT**	Up-Regulated	2.102120309	4.73E-14
**DBF4B**	Up-Regulated	1.107609957	4.91E-09
**ATAD5**	Up-Regulated	1.147833059	3.03E-07
**TICRR**	Up-Regulated	3.204105177	6.38E-17
**CKAP2L**	Up-Regulated	3.16171032	3.67E-15
**SPC25**	Up-Regulated	3.010985429	1.22E-17
**SPDL1**	Up-Regulated	1.436094997	1.12E-13
